# Establishing haptic texture attribute space and predicting haptic attributes from image features using 1D-CNN

**DOI:** 10.1038/s41598-023-38929-6

**Published:** 2023-07-19

**Authors:** Waseem Hassan, Joolekha Bibi Joolee, Seokhee Jeon

**Affiliations:** Department of Computer Science and Engineering, Kyung Hee University, Yongin-si, Gyeonggi-do South Korea

**Keywords:** Physiology, Psychology, Engineering

## Abstract

The current study strives to provide a haptic attribute space where texture surfaces are located based on their haptic attributes. The main aim of the haptic attribute space is to come up with a standardized model for representing and identifying haptic textures analogous to the RGB model for colors. To this end, a four dimensional haptic attribute space is established by conducting a psychophysical experiment where human participants rate 100 real-life texture surfaces according to their haptic attributes. The four dimensions of the haptic attribute space are rough-smooth, flat-bumpy, sticky-slippery, and hard-soft. The generalization and scalability of the haptic attribute space is achieved by training a 1D-CNN model for predicting attributes of haptic textures. The 1D-CNN is trained using the attribute data from psychophysical experiments and image features collected from the images of real textures. The prediction power granted by the 1D-CNN renders scalability to the haptic attribute space. The prediction accuracy of the proposed 1D-CNN model is compared against other machine learning and deep learning algorithms. The results show that the proposed method outperforms the other models on MAE and RMSE metrics.

## Introduction

The initial medium that humans use for attaining information about textures is the visual sense^1,2^. The appearance of a texture can provide us with enough information to be able to successfully identify its physical attributes in most cases^3^. In order to gain in depth information about the said texture, humans rely on the sense of touch^4,5^. Interaction with a texture to reveal its haptic attributes is a trait intrinsic to human beings. In daily life interactions, human beings use these two senses to identify haptic attributes of textures all around them. Recently, researchers have pointed out that interaction sounds can play a role in haptic identification^6^. However, it only stands for certain haptic attributes and do not play a role as important as vision or haptics in texture perception^7,8^.

Visual texture perception can be readily described by the RGB model (or CMYK in case of pigments). The RGB model is a physical-level descriptor for identifying visual texture of an object^9–11^. In the same sense, surface topography of a texture can be termed as the haptic equivalent of the RGB model for visual texture. As humans, we label various combinations of RGB as colors to make them understandable and more relatable for the general public. However, we do not have a similar representation system for describing and understanding haptic texture or surface topography of an object. Haptic texture perception of an object is the result of interaction, and in the absence of interaction, any efforts made in explaining surface topography to a layman would be hard to understand and mostly futile.

The motivation for this study lies in addressing two significant gaps in the understanding of haptic texture perception: the lack of a universal rendering system and standardized perceptual dimensions. Unlike visual perception, which benefits from common frameworks such as the RGB model, haptic perception lacks an accessible, standardized mechanism. Moreover, the absence of a common language to describe and understand haptic texture or surface topography presents challenges in both academic research and practical applications. This disparity limits our ability to communicate, understand, and experiment with haptic textures effectively. By proposing the Haptic Attribute Space (HAS), we aim to establish a framework akin to a swatch book for the visual sense, where haptic textures can be readily identified and arranged based on their haptic attributes (roughness, hardness, etc). Such a system would make haptic textures more comprehensible to both the general public and professionals working with haptic texture modeling. In the contemporary world, consumers buy online products without being able to touch them. Consumers are less likely to buy online products that have a strong tactile aspect^12^. A standardized system where haptic attributes of products are made available would allow the end user to make more informed decisions. By establishing the Haptic Attribute Space (HAS) and a method to infer haptic properties from visual data, we aim to democratize the understanding of haptic textures, making them more accessible and applicable across various domains.

Recently, different researchers have attempted to explore/identify haptic attributes of real and virtual textures via their physical or virtual properties. To this end, Wu et al.^13^ and Mun et al.^14^ created textures with perceptually differentiable haptic attributes by manipulating properties of virtual textures. Similarly, Ujitoko et al.^15^ were able to generate vibrotactile signals to render different textures based on their images or haptic attributes. The studies in^16,17^ used the SynTouch BioTac sensor to collect physical interaction signals from objects and use them for predicting haptic attributes of textures. Another study attempted to establish one such space (authoring space) where textures were located based on their haptic attributes^18^. The space was created from a combination of physical interaction signals (contact acceleration patterns) with texture and haptic attributes (hardness, roughness) of texture. A total of 25 texture surfaces were used in this study. In the authoring space, new haptic textures could be rendered (by interpolation) based on arbitrary attribute values within the convex hull of the authoring space textures. This space was primarily used for haptic texture authoring but it did contain haptic textures populated based on their haptic attributes. However, the aforementioned studies had a few shortcomings if they were to be considered for a standardized haptic attribute space. First, the dataset in some studies was too small to begin with and statistically satisfactory results could not have been achieved with it. Second, scalability in terms of adding new real textures into the space was not straightforward. In certain studies the whole space would have to be remade in case of a new entry. Third, the authoring space in^18^ was intended to create virtual textures inheriting properties of real textures, while our current goal is to establish a space which can accommodate and identify new real textures based on their haptic attributes. Last, all of the aforementioned studies needed physical interaction with a surface in order to incorporate them into the space.

In the present system, the main aim is to facilitate and expedite the process of haptic attribute extraction by avoiding the requirement of physical interaction for every new texture, which introduces a bottleneck into the process. To this end, the current study attempts to extract haptic attributes of texture from image features, without the need for physical interaction. The use of image features for haptic attribute extraction in combination with the structure of the attribute space facilitates the rapid scalability of the overall attribute space in terms of addition of new textures into the space.

The current study aims to provide a Haptic Attribute Space (HAS) where haptic textures are defined by their haptic attribute values. The HAS is a four dimensional space where the dimensions are haptic attributes of textures, i.e., rough-smooth, flat-bumpy, sticky-slippery, and hard-soft. The study comprises of two parts. The first part of the study deals with establishment of the HAS from a dataset of 100 texture surfaces. The HAS is established by conducting psychophysical experiments with human subjects. They select a list of attributes that could define the haptic properties of the surfaces in the dataset, and then rate all the 100 surfaces in the datasets against those attributes. The four attribute pairs chosen as a result of this exercise become the axes of the HAS. The second part deals with generalizing the HAS to new textures by using images of new textures and predicting their attribute values. It is proclaimed that there lies an area of intersection between visual and haptic texture perception^19–21^, and this study aims to exploit that area of intersection. This study introduces a multi-scale 1D-CNN model to predict haptic attribute values of new textures from their image features. The 1D-CNN model is trained using the data from psychophysical experiments and image features of the 100 surfaces dataset. The aim of the 1D-CNN model is to make it possible to assign attribute values to newly seen and/or physically absent texture surfaces.

### Contributions

The major contributions of the paper are listed below.

- Collecting a dataset of 100 unique texture surfaces and establishing their perceptual space.
- Establishing a four dimensional Haptic Attribute Space from the dataset of 100 texture surfaces. The HAS describes the real textures based on their haptic attribute values.
- Designing a multi-scale 1D-CNN model to predict haptic attributes of textures based on their image features.

### Expected outcomes

The HAS allows a textured surface to be defined by its haptic attributes as rated by human subjects. For example, how smooth or how bumpy a surface feels. A surface with a higher value of smoothness would intuitively mean a smoother surface. It would help in categorizing and defining textures based on their haptic attributes. In general, humans compare textures by going back and forth exploring all the textures while concentrating on different aspects of perception each time. Oftentimes, the characteristics should be memorized in the form of multiple perceptual scales and, keeping them consistent is not an easy task. The HAS describes textures numerically, which makes it intuitive to rank various textures in terms of certain properties or as a combination of several. Given that new textures can be effortlessly placed into the HAS without the need to remake the whole space, it is possible to scale the HAS using the 1D-CNN.

The 1D-CNN used for predicting haptic attributes of textures can be used to haptically describe arbitrary textures from their images. It is important to acknowledge that human users maintain a certain degree of standard deviation in their haptic ratings for the same textures. Therefore, if the prediction error of the algorithm remains less than the standard deviation observed in human ratings, the error could be deemed perceptually insignificant.

From a practical standpoint, the predicting capability opens several avenues of applications for the proposed system. The HAS could be utilized to ease the e-commerce experience. In the fashion and textile industry, this approach could enable designers and consumers to better understand the feel of fabrics and materials from online images, assisting in design and purchasing decisions. Such a system would let users make informed decisions while shopping online. Another possible application of the HAS could be in the haptic mapping of remote environments. One of the benefits of the proposed system is that surfaces can be haptically labeled from images, without needing physical interaction with the actual surface. Once the images of a remote environment are available, its contents could be segmented and each segment could be haptically labeled using the proposed 1D-CNN model. For individuals with visual impairments, this research could significantly enhance accessibility technologies, making tactile maps, braille displays, and haptic feedback devices more detailed and useful. In fields where physical interaction may be harmful, like archaeology or art preservation, our method could provide additional haptic information without risking damage.

## Related works

### Haptic texture attributes

Haptic interaction with textured surfaces can occur in two ways, i.e., tool-mediated interaction, or bare-handed interaction. In bare-handed interaction, a user directly touches the textured surface using their fingers. In tool-based interaction, a user interacts with a textured surface through a rigid probe, and the interaction vibrations pass through the tool onto the user’s hand. Researchers have used both these techniques to discover the underlying perceptual dimensions of haptic textures. In case of bare-handed interactions, pioneering work was done by Yoshida et al.^22^. Their experiments resulted in four major perceptual dimensions, namely, hard-soft, heavy-light, cold-warm, and rough-smooth. Two of these dimensions (rough-smooth, hard-soft) were corroborated by Hollins et al.^23^ by using bipolar haptic attributes. Other studies^24–26^ concluded that haptic texture consisted of three main perceptual dimensions, i.e., rough-smooth, hard-soft, and cold-warm. Okamoto et al.^24^ presented a comprehensive study and provided a strong case for including friction as a major perceptual dimension. They also provided reasonable arguments for differentiation between macro and micro roughnesses as perceptual dimensions. Currently, a total of five attributes are considered as the major perceptual dimensions for haptic perception of texture. These dimensions are micro-roughness, macro-roughness, friction, stiffness, and warmth. In tool mediated interactions, Lamotte et al.^27^ used active tapping as a method of interaction and reported that texture perception mainly varied on the hardness-softness dimension. They reported that softness can be more easily discriminated when participants used active tapping as a mode of interaction. Similarly, other researchers uncovered the rough-smooth dimension using tool mediated interactions^28,29^.

Most attempts at describing haptic attributes deal with perceptual dimensions. The relation between physical attributes of texture and the perceptual dimensions is not clearly defined. Therefore, researchers have focused on the relation between haptic attributes of texture (roughness, stiffness, etc) and the perceptual dimensions. The haptic dimensions and attributes of texture have been successfully identified^22,24,28,30^. However, the goal of these studies was the dimensions/attributes only and no further investigation was done.

### Haptic texture classification

Texture classification using image features has remained the focus of several researches over the years. One of the pioneering efforts was presented by Haralick et al.^31^. They introduced the Grey Level Co-occurrence Matrix (GLCM) from which image features were calculated and used for texture classification. Various texture classification algorithms have been presented with ever improving prediction accuracy. These algorithms include but are not limited to filter bank features^32^, binarized statistical image features^33^, local binary pattern features^34,35^, color maps^36^, neural networks^37,38^, etc. All the above mentioned algorithms and many more such texture classification algorithms reported high accuracy on various texture datasets. However, these are purely based on image features and as such could not be applied to haptic texture classification.

A recent method of collecting sensorized data from haptic interaction with textures has been used for haptic texture classification. Customized interaction tools with various sensors are used to collect information from texture surfaces. Various features are calculated from this collected information. In this regard, Stresse et al.^39–41^ used a custom built pen-like tool to interact with textures and collect acceleration signals, sound, frictional force, and images. These data are then used to collect various features that are used for haptic texture and material classification. Similarly, Romano et al.^42^ used normal force, friction, scanning velocity and acceleration resulting from tool exploration. Kerzel et al. used a single force sensor to record the signals during lateral and vertical motions of the tool and trained a neural network using these data for compliance and texture identification^43^. Recently, Lima et al. used raw data from an inertial measurement unit (IMU) and deep barometer for texture classification using machine learning techniques^44^.

In essence, the aforementioned techniques use physical vibrations from interaction with surfaces or mechanical properties of textures to classify haptic textures. It can be argued that such data could provide a high accuracy in haptic texture classification tasks. However, the process of collecting information from real textures every single time can be a tedious and time consuming process. One of the bottlenecks of such methods is the requirement of a physical surface for classification, as the signals are collected by interacting with a real surface, therefore, generalization or scalability of the system can be hindered. Using the proposed system, haptic attributes/properties of textures can be classified or identified based on their images only. Thus eliminating the requirement of a real texture, and making the process of haptic attribute identification more robust and usable.

### Visual and haptic texture

Humans rely on both visual and haptic information when interacting with an object. Both the modalities contribute towards the identification of the object. Information about shape, color, location, etc., is mostly provided by the visual sense, while, the haptic sense aids in attaining richer texture information^21,45^. Contrary to popular belief, Lederman et al. and Heller showed, in separate studies, that vision and haptics perform equally well in texture perception tasks^19,45^. In fact, it was argued that texture perception is intrinsically a bimodal (visual and haptic) phenomenon and perception degrades if observed through either of the individual modalities. Humans judge haptic and visual cues of real textures similarly, and the two modalities depict congruent perceptual characterizations^46,47^. Functional Magnetic Resonance Imaging (fMRI) evidence shows that haptic and visual perception of texture activate same areas within the medial ventral temporal cortex of the brain^48^. Similarly, the fMRI based studies by Eck et al. indicated a crossmodal interaction in the somatosensory and visual cortices when humans process texture information^21^. Either of the visual or haptic sense can attain a dominating role in terms of texture perception depending on the nature of the task and the amount of variability available to either of the modality. To this end, Ernst et al. modeled the human nervous system responses using a maximum-likelihood integrator which accepted visual and haptic cues as inputs and estimated the role of each modality in perception^20^.

**Figure 1:**
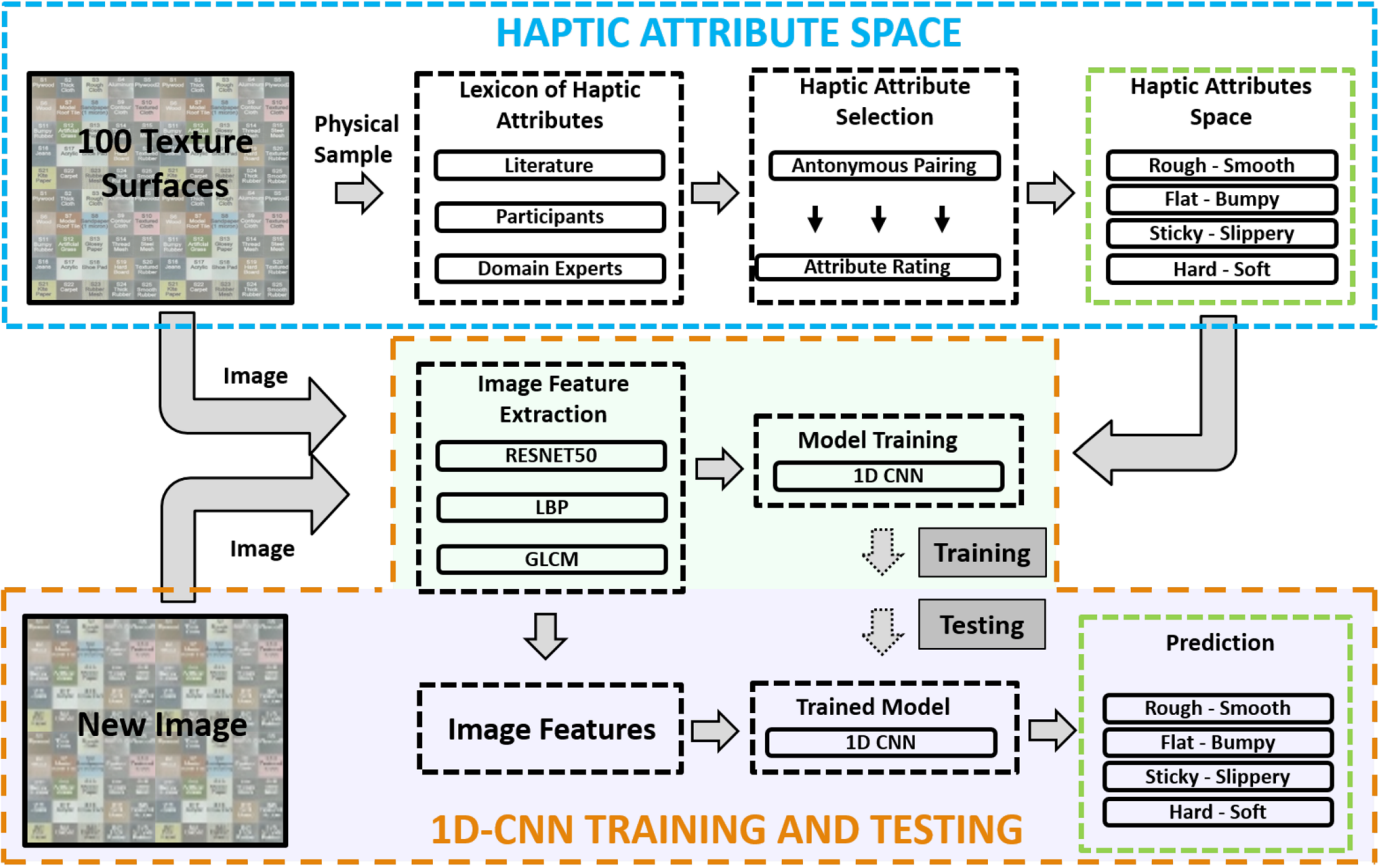
A block diagram of the overall framework. The top row details the steps required to establish the HAS. The next two rows show the training and testing methodology of the 1D-CNN.

The aforementioned studies provide evidence that the visual and haptic sense operate in a harmonious manner and that there remains an overlap between their functionalities. The current study aims to capitalize this overlap of haptic and visual senses and to use it for predicting haptic attributes of textures using their images.

## Overview

Figure 1 presents the overall system and the interaction of individual components. The constituent components of the system and their relationship will be briefly defined in this section.

The main aim of the current study is to provide a standardized space where texture surfaces are located based on perceptually meaningful haptic attributes. This is achieved by accurately predicting the haptic attributes of texture surfaces from their images and subsequently locating the haptic textures in terms of quantifiable haptic characteristics in a haptic attribute space. The overall study can be divided into two major parts, i.e., establishing the haptic attribute space, and establishing a relationship between the haptic attributes and the image feature space.

The study starts with the collection of 100 different texture surfaces (see “Texture dataset” section) which are rated by human users to establish the haptic perceptual space (see “Haptic perceptual space” section) and the haptic attribute space. The haptic perceptual space is a 3D space achieved from the multidimensional scaling (MDS) of similarity ratings from human subjects. The haptic attribute space is established based on user ratings of texture surfaces according to different perceptual properties. The haptic attribute space is a four dimensional space where each dimension defines a perceptual characteristic of the 100 haptic textures used in this study. All the textures are located in this four dimensional space according to user ratings.

The next step is to establish an image feature space (see “Image feature space” section) where each surface is defined by its image features. A combination of various algorithms is used to extract meaningful textural features from the 100 texture surfaces used in this study.

A relationship between the haptic attribute space and the image feature space is established using a multi-scale 1D convolutional neural network (1D-CNN) (see “1D-CNN” section). The 1D-CNN takes the image features extracted from texture surfaces as input, and predicts the corresponding haptic attributes. The haptic attribute space established as a result of this exercise can potentially be scaled by populating it with new unseen textures.

## Haptic attribute space

The Haptic Attribute Space (HAS) is a four dimensional space where haptic textures are located based on the differences in their physical characteristics and properties. Each axis of HAS represents a particular attribute that defines the textural properties of surfaces. Psychophysical experiments are conducted on a dataset of versatile textures to establish the HAS space. In one experiment, human subjects rated all the textures according to various attributes that define the haptic properties of textures. These ratings were used to establish the four dimensional HAS space. In another experiment, the human subjects rated the texture surfaces based on their perceived dissimilarities. The result of the second experiment created a three dimensional perceptual space where texture surfaces are placed based on their dissimilarities. The perceptual space highlights that the variance in the 100 texture surfaces can be satisfactorily explained using only three dimensions, whereas, the HAS has four dimensions. The four dimensions of HAS explain the perceptual attributes of textures, and these are prone to perceptual correlations. The attribute pairs are regressed into the perceptual space in “Haptic attribute regression into perceptual space” where they exhibit non-orthogonal intersection angles to show their correlation. The details of the texture dataset, the psychophysical experiments and their purposes are provided in the following sections.

**Figure 2:**
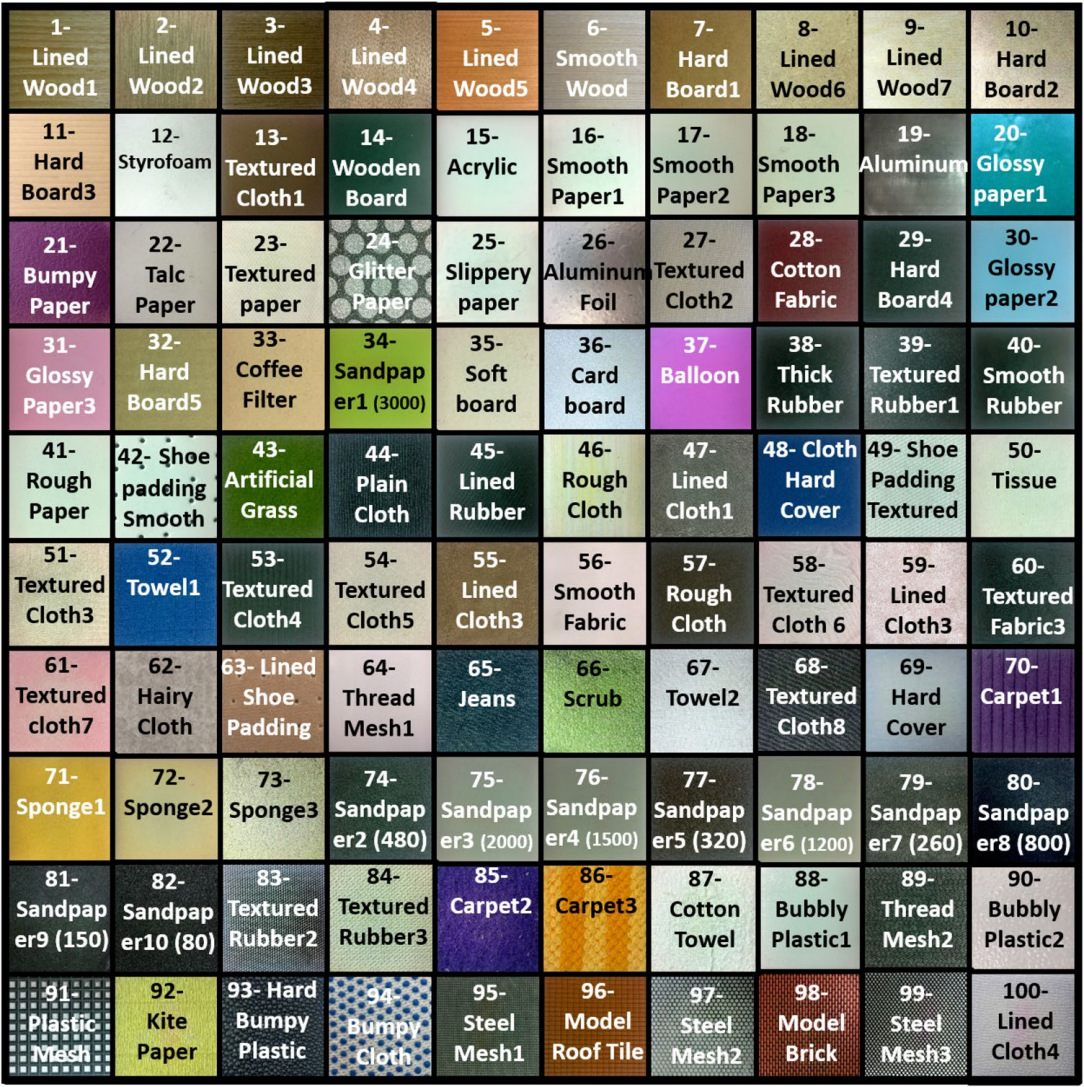
The 100 texture surfaces used in this study.

### Texture dataset

A total of 100 different texture surfaces were collected to be used as stimuli in both psychophysical experiments. An effort was made to collect texture surfaces in such a way that they captured a wide variety of daily life haptic interactions. The 100 texture surfaces are provided in Fig. 2 and the dataset is provided as a Supplementary Material. The textures in the dataset can be subjectively categorized into varied categories based on material or textural/surface properties. The material categories are wood, rubber, plastic, fabric, leather, sandpaper, paper, sponge, and metal. Each of these categories contains multiple texture surfaces, i.e., flat surfaces with varying degrees of smoothness, metallic and fabric meshes, fabric with visible threads, flat fabrics, wooden surfaces with different textures, and many more. Some of the textures in these subjective categories are similar in haptic perception, while others are completely different. There are surfaces present with similar textural properties across different categories.

All the texture surfaces were cut to a size of 100 × 100 mm and mounted on hard acryl plates of the same size. This was done to provide a uniform base for all the surfaces, otherwise, the stiffness of the underlying table-top could influence their perception. Some of the metallic and thick wooden texture surfaces were not mounted on top of acryl plates, as the participants could not perceive the effect of the underlying tabletop through these surfaces.

**Figure 3:**
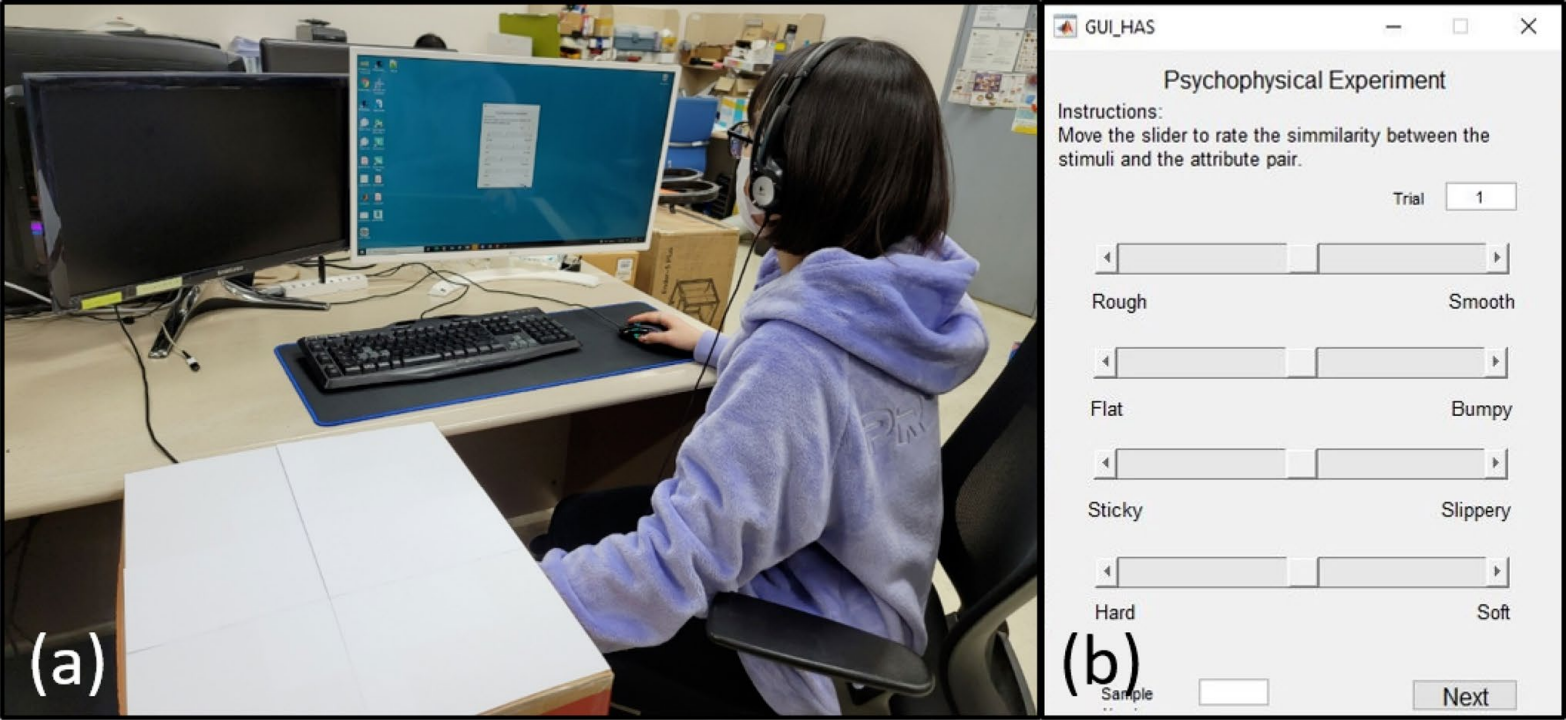
(**a**) The experimental setup for the haptic attribute rating experiment. (**b**) The GUI for haptic attribute rating experiment.

**Figure 4:**
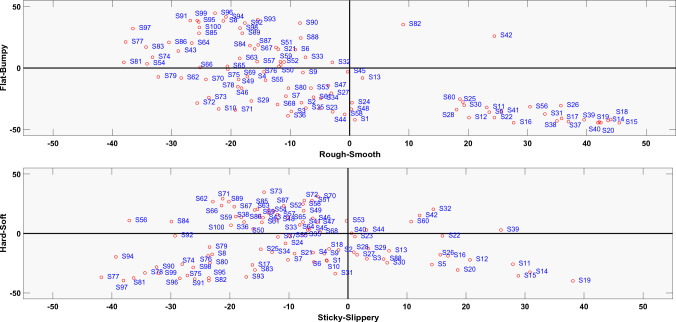
The four dimensional haptic attribute space is shown as two 2-dimensional spaces. The texture surfaces are scattered around in the haptic attribute space.

### Haptic texture attribute space

The main aim of this experiment was to identify the prominent haptic attributes that can be used to explain the textural properties of the 100 surfaces. The identification of these prominent haptic attributes was carried out using the semantic differential method. It was a multi-step process. First, a comprehensive list of attributes/adjectives was compiled that could potentially describe texture surface properties. Second, the list was narrowed down to 8 attributes (4 attribute pairs) based on participant responses. Third, the 100 texture surfaces were rated according to these four attribute pairs. The four attribute-pairs formed the four axes of the four dimensional HAS where all the surfaces were located according to their corresponding attribute values.

#### Participants and stimuli

The 100 texture surfaces detailed in “Texture dataset” section were used as stimuli in this experiment. A total of 26 participants took part in the experiment (19 male and 7 female) with an average age of 28 (ranging from 25 to 34).

#### Procedure

The participants sat in a chair in front of a table. They wore headphones playing white noise at a volume that blocked interaction and environment noises but experiment instructions could be heard. The texture surfaces were provided one at a time. The participants were allowed to use their preferred exploratory methods for interacting with the textures. The surfaces were placed under a cardboard box with a small opening for the participant’s hand at one end and another larger opening for the experimenter to replace the surfaces. The participants were not able to see any of the surfaces throughout the experiment. The experimental setup is presented in Fig. 3a.

The first part of the experiment was to collect a comprehensive list of haptic attributes. A total of 60 haptic attributes were collected in total. Some of these came from literature^14,49–51^, while the others were selected by human participants keeping in mind the type of textures present in the dataset. The full list of attributes is provided in Table 1.

**Table 1:** The list of attributes provided to participants for the attribute rating experiment.

Abrasive	Granular	Bald	Bouncy	**Flat**	Glassy
**Hard**	Cold	Grating	Warm	Pointy	Fizzy
**Sticky**	Sharp	Wavy	Wooden	Hatched	**Smooth**
Jarred	Patterned	Solid	Mild	Silky	Malleable
Prickly	Metallic	Refined	Angular	Rigid	**Rough**
Jagged	Irritating	**Slippery**	Mushy	Slick	Furry
Grainy	Pleasant	**Bumpy**	Spongy	Bubbly	Thick
Fine	**Soft**	Blur	Slow	Fast	Deep
Shallow	Thin	Heavy	Blunt	Light	Dark
Bright	Vague	Distinct	Sparse	Dense	Even

The four selected attribute pairs are in boldface font.

The participants were asked to select attributes that they felt could describe the haptic properties of the texture surfaces. The decision was either a 1 for yes or a 0 for no. This process was conducted to filter out the particular attributes that were dominant among the 100 surfaces used in this experiment.

A total of 11 attributes (selected by at least 50% of the participants) were short listed for the next experiment. Among these 11 attributes, four pairs of attributes with an antonymous relation were selected as they could represent opposite extremes of the same property. The four pairs were rough-smooth, flat-bumpy, sticky-slippery, and hard-soft. The three attributes that did not form antonymous pairs were pleasant, even, and abrasive. It must be noted that hard-soft in this experiment referred to texture and not compliance of the surface. All the surfaces were mounted on acryl plates to minimize the compliance bias. These four pairs were used in the next part of the experiment.

In the next part of the experiment (by same participants), participants rated all the texture surfaces according to the four attribute pairs selected in the first part. A GUI (graphical user interface), as shown in Fig. 3b, displayed each of the four attribute pairs on opposite sides of a slider. The slider had no scale marks and spanned a length of 127 mm^49^ on the screen. The participants were asked to perceive the surfaces and slide the marker in the direction of the attribute. The slider values were mapped from zero to hundred and averaged across all participants.

#### Analysis and results

The four attribute pairs acquired as a result of this experiment were used to establish the HAS. Each attribute pair represents a unique dimension. The four-dimensional HAS is shown in Fig. 4 in the form of two two-dimensional plots. The data for all the participants is averaged. The axes of the plots are marked with attribute pairs. Originally the participant responses were rated between zero and 100 with 50 being the center point, however, in Fig. 4 the attribute values are centered around zero. The shifting of the ratings does not have any affect on their perception. It has been done to provide an easier visual understanding of the plots.

### Haptic perceptual space

The haptic perceptual space is a multi dimensional space where surfaces are scattered based on their perceptual similarities. Perceptually similar surfaces are grouped together while perceptually different surfaces are located away from one another. The similarities among surfaces are determined by human participants using a cluster sorting psychophysical experiment.

**Figure 5:**
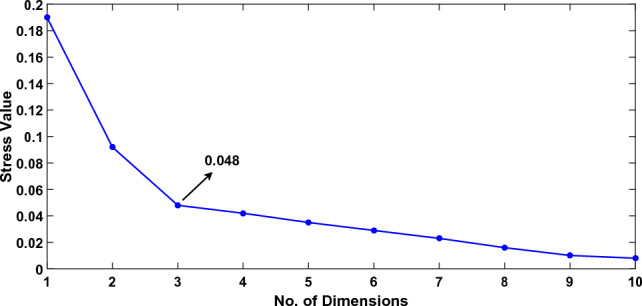
The Kruskal stress value for the first ten dimensions of the perceptual space.

#### Participants and stimuli

The 100 texture surfaces detailed in “Texture dataset” section were used in this experiment. The 26 participants who took part in the previous experiment also took part in this experiment.

#### Procedure

The participants sat in a chair in front of a table. They wore headphones playing white noise to block out interaction and environmental noise. The participants were blindfolded to avoid visual bias.

This experiment was in the form of a cluster sorting task. The participants were asked to group perceptually similar textures into a predefined number of groups. They were free to use their preferred method of texture exploration. Every participant conducted a total of five trials where the total number of groups were 3, 6, 9, 12, and 15. The order of the trials was counter-balanced using Latin square.

One surface was provided at a time which was assigned to a group by the participant. The next surface, if perceptually similar, could be assigned to the same group, otherwise, it could be placed in a new group. After assigning all the surfaces to the predefined number of groups, participants were asked to reevaluate the groups for errors. The five trials on average took 160 min per participant excluding break times.

#### Analysis and results

Data from the experiment were in the form of groups made across various trials. Scores to a pair of surfaces in the same group were assigned based on the total number of groups in that trial. Scores for the surfaces across all trials were subsequently added together to get a similarity score. For example, a pair of surfaces were grouped together in the trials with total groups at 6, 9, and 15. The similarity score for this pair would be 6 + 9 + 15 = 30. This method ensures that the surfaces that were grouped together across more trails receive a higher similarity score. The data across all participants were averaged and used to form a similarity matrix. The similarity matrix was converted into a dissimilarity matrix and scaled from zero to one.

**Figure 6:**
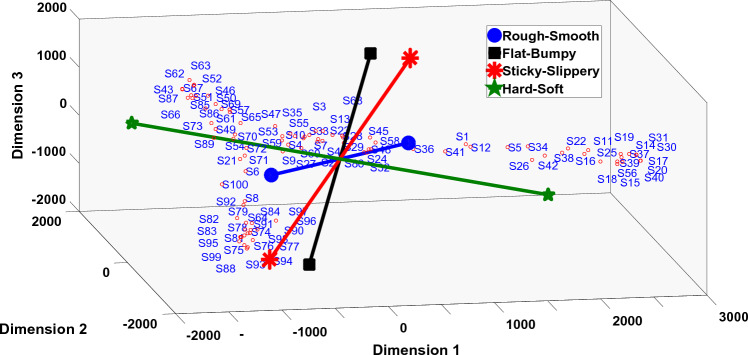
The four attribute pairs are regressed into the three dimensional perceptual space. The length of the attribute pair shows the goodness of fit.

Multi-dimensional scaling (MDS) analysis was performed on the dissimilarity data to establish the perceptual space. The number of dimensions for MDS was determined by running a Kruskal stress test on the MDS data. The stress test results in Fig. 5 show a stress value of 0.048 at the third dimension which is considered as fair according to^52^, therefore, a three dimensional space is sufficient to describe the variation across the data in our dataset. The perceptual space is provided in Fig. 6. The dimensions obtained from the MDS analysis do not portray any physical meaning. These are the result of an optimization algorithm used to locate the given surfaces at optimum distances to maintain their dissimilarity ratings.

### Haptic attribute regression into perceptual space

In order to make sense of the dimensions of MDS in terms of attributes of a surface, the four attribute pairs from “Haptic attribute space” section are regressed into the perceptual space. Multi-linear regression was performed where the perceptual space dimensions were the response variables, whereas, the attribute scores were used as the predictor variables. Figure 6 shows the four attribute pairs regressed into the perceptual space. The length of each attribute pair shows the goodness of fit of the regression model.

The HAS is the combination of the four attribute pairs, and ideally, one would want the four dimensions to be perpendicular to one another. However, Fig. 6 shows that the attribute pairs are not perpendicular. This analysis further emphasizes the fact that haptic perception is not a linear phenomenon. Furthermore, it shows that haptic attributes of texture are dependent on one another up to varying degrees. This nonlinearity is carried and contained in the HAS dimensions. The angles of the four attribute pairs with respect to origin are provided in Table 2.

The HAS can be constructed in two different ways. First is by projecting the points in perceptual space onto the regressed attribute pairs. The projections form the four dimensions of the HAS. This method preserves the ordinal relationship between the points. The second method is to directly use the attribute rating values provided by the users to form the dimensions of the HAS. In the current study, the second method was followed. The motivation behind the current study was to introduce standardized dimensions where textures can be located and differentiated based on their differences. Therefore, in addition to the ordinal differences, the numeral differences were considered important to be preserved.

**Table 2:** The 3D angles for the four attribute pairs regressed into the perceptual space.

Attribute pair	Elevation	Azimuth
Rough–smooth	324.48	99.93
Flat–bumpy	70.66	52.0
Sticky–slippery	228.96	47.77
Hard–soft	345.58	338.09

## Image feature space

The main aim of the current study is to establish a system that enables us to identify the haptic attributes of a texture only by examining its image (visual texture). To this end, images of the textures in our dataset were used to collect meaningful features that will be used in training the 1D-CNN model. A large variety of image feature extraction techniques are available in the literature. In the current study, we decided to use an amalgam of classical and deep learning based image features. Three image feature extraction techniques were used, i.e., Gray Level Co-occurrence Matrix (GLCM)^31^, Local Binary Pattern (LBP)^34^, and ResNet50^53^. A wide variety of other feature extraction techniques were tested before settling down on the aforementioned three methods. The three methods chosen in this study contain a complementary modus operandi. LBP captures the local spatial patterns in an image, GLCM has the ability to capture local and intermediate level features, while, ResNet50 progressively extracts higher level deep features from the input images. A combination of these three features covers diverse aspects of an image and the resulting image features show high discrimination ability. The details of the image capturing setup and image features are provided in the following sections.

### Image capturing setup

All the images used for training were captured using a standardized and uniform procedure to guard against any scaling or resolution bias. It is important to capture the finer details of the surface with clarity and in high enough resolution so that the algorithm can extract meaningful features from the images. All images were captured using dp2 Quattro SIGMA digital camera and saved in high quality RAW format (14-bit lossless compression). The camera was mounted on top of the surfaces using a tripod stand. The distance between the camera lens and the surfaces was kept constant at 300 mm. The images were taken in standard room lighting, however, special care was taken to guard against any shadows. Color does not affect the haptic perception, therefore, all the images were converted into grayscale before using them in training to remove/reduce color bias.

### Gray level co-occurrence matrix

In^31^, Grey Level Co-occurrence Matrix (GLCM) based texture feature descriptor was proposed for surface classification. Recently, for haptic texture assignment in^54^, GLCM was utilized as one of the texture features because of its higher performance in this area. Motivated by this, we employed the GLCM, which considers the spatial relationships between two pixels at a time in the surface texture image. First, the surface images are resized into 1568 × 1568. Then, the GLCM method is applied to this resized surface image, which produces a matrix of 8 × 8. Then, this matrix is flattened to generate a feature vector of size 1 × 64.

### Local binary pattern

Local pixel information from an image can be calculated by using Local Binary Pattern (LBP). The LBP is performed by comparing the pixel values of an image by thresholding a circular neighborhood area^34^. In this work, we applied the LBP method on the surface image to calculate the local spatial patterns. First, the resized input images are divided into multiple cells with sizes 224 × 224. Then, the LBP operation is performed on each cell, which generates a feature vector with the size 1 × 59. Subsequently, the feature vectors obtained from each cell are combined to produce the final feature vector with size 1 × 2891 using the LBP.

### ResNet50

The ResNet model was presented in^53^ for the image classification task, which is trained on the ImageNet dataset. The network achieved state-of-the-art performance in image recognition due to having residual learning support. In our work, we used the pre-trained ResNet-50 model to capture the higher level deep features from the surface images. At first, the surface images are resized into 224 × 224 in order to match the input of the ResNet-50 model. Then, the processed surface images are fed into the pre-trained ResNet-50 model, which gives us the feature vector with size 1 × 1000 containing the deep spatial information of the surface texture.

After capturing surface features by employing the GLCM, LBP and ResNet-50, we concatenate the features and produce a feature vector with size 1 × 3955, which is then used as input to the multi-scale 1D-CNN.

**Figure 7:**
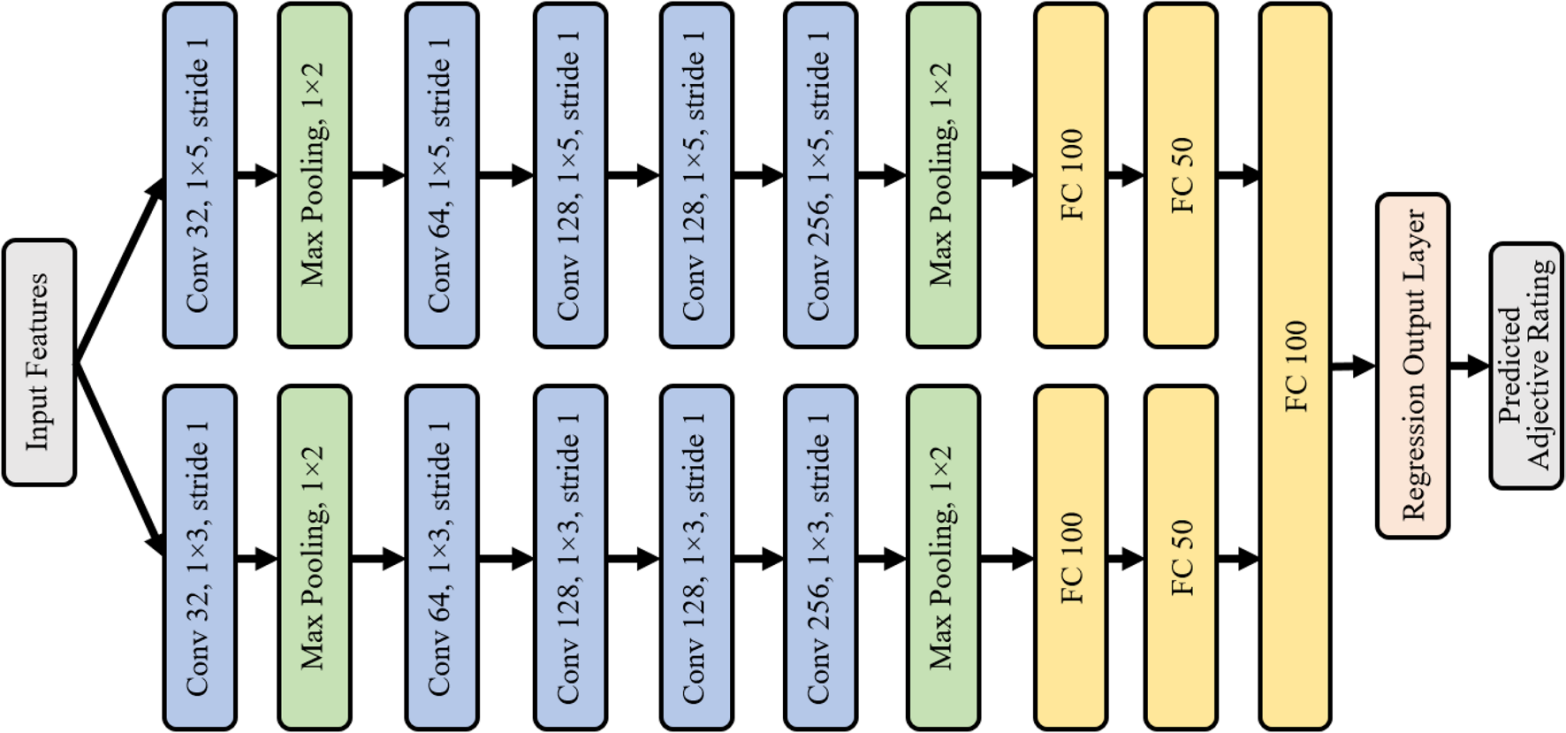
The structure of the proposed multi-scale 1D-CNN.

## 1D-CNN

Recently, deep learning-based approaches have become popular for haptics applications, i.e., tactile understanding^55^, texture signal generation from surface images^37^, perceptual similarity learning from haptic textures^56^ and so on. In light of the aforementioned work’s success, in this paper, we design a multi-scale 1D-CNN to establish a relationship between haptic attributes of surface texture and its image features. The 1D-CNN model was preferred over 2D-CNNs because 1D contains significantly lesser parameters. A higher number of parameters can increase training time and may result in overfitting of the model when training data is limited. The proposed infrastructure contains two strands of 1D-CNN models, where each model captures details at different scales and thus captures the macro and micro level information separately. The infrastructure of the 1D-CNN used here is similar to that of a conventional CNN. The difference is the use of the input data and trainable kernels of the one-dimensional (1D) vector. As a consequence, during the training phase, the forward propagation and backpropagation procedures are modified. The structure of the proposed multi-scale 1D-CNN is presented in Fig. 7. The model takes the image features captured using the previously discussed methods. Then, we train the model with respect to the given haptic attribute values. Ultimately, the model is able to predict the haptic attribute values for the given surface image features. The details of the proposed multi-scale 1D-CNN are as follows.

The network contains two sub-1D CNNs. Each 1D-CNN has five 1D convolutional layers, two 1D max-pooling layers, and two fully connected layers. The convolutional layers are in charge of extracting the features, while the max-pooling layers reduce the dimensionality of each feature map. In the convolutional layers, different numbers of kernels are applied with different scales. Therefore, local spatial information in different scales is captured. In the convolution operation, we operated 1 × 3, and 1 × 5 sizes of kernels, while the max-pooling process is performed on 1 × 2 blocks. Additionally, we utilize multiple kernels to obtain the diverse aspects from each scale of local information in convolutional layers. For instance, the first convolution layer operates 32 kernels, the second convolution layer uses 64 kernels, the third and fourth involve 128 kernels, while the fifth convolution layer operates 256 kernels. More specifically, in a 1D convolution layer, the computation is performed as follows.

1 $$\begin{aligned} g_{i}=f(w_{i}^{T}a_{n}+b_{i}) \end{aligned}$$

2 $$\begin{aligned} f(z)=\left\{ \begin{array}{ll} z; & \quad if \; z > 0 \\ 0; & \quad otherwise, \end{array}\right. \end{aligned}$$

where g$_\text {i}$ is the calculation result of the ith filter, a$_\text {n}$ is the input data of size 1 × N, w$_\text {i}$ is the ith convolutional kernel vector with size 1 × N, b$_\text {i}$ is the bias of the ith filter and the ReLU nonlinear activation function is denoted as f.

Each sub-1D CNN model ends with two fully connected (FC) layers having 100 and 50 neurons, respectively. Finally, another FC layer with 100 nodes is engaged to concatenate the features achieved through different 1D CNN modules. The loss function in this study is the mean-square error (MSE), while the activation function is the Sigmoid function. To train the model, the ADAM optimizer is used.

In most cases, the number of samples is significantly less than the number of features when it comes to sampling costs in real-world applications. Due to the lack of texture samples, the complex model can easily lead to overfitting problems without considering the model’s generalization ability. In other words, the fitted model can correctly predict the adjective rating for the training data, but the test set’s prediction results are poor. Therefore, to balance prediction performance and speed up the deep network training process, the rectified linear unit (ReLU) and batch normalization (BN) techniques are used after the convolution operation in this study.

## Evaluation

The main goal of the proposed system is to predict accurate and reliable haptic attributes of textures based on their images. It is important to reliably predict these attributes and that the errors in haptic attribute value prediction stay below human perception. It is safe to assume that an absolute error rate of less than the mean standard deviation between participant responses can be considered perceptually insignificant. In this section, a numerical evaluation of the proposed model is carried out using the Leave-One-Out Cross-Validation (LOOCV). The evaluation assesses the prediction capability of the system in terms of how well it can predict haptic attribute values for unseen textures.

The proposed model was also compared against other prominent classical and neural network algorithms in terms of prediction accuracy. Linear regression and Support Vector Regression were chosen among the classical algorithms. These are two of the most commonly used regression techniques. In case of neural network models, an Artificial neural network and state-of-the-art 1D CNN by Taye et al.^57^ were selected.

### Leave-one-out cross validation

Cross validation is an evaluation method used to verify the estimation capability of a trained model on unseen data. It tests the generalization ability of a trained model on a dataset that was not used in training the model. One of the most common forms of cross validation is the *k*-fold cross validation, where the dataset is split into *k* subsets. A fixed number of subsets are used for training and the remaining are used for testing. The process is repeated until all the subsets are used for testing. LOOCV is a special case of the *k*-fold cross validation where *k* = 1. The number of subsets is equal to the number of instances in the dataset. In every cycle, all the instances are used for training barring one which is used for testing. The process is repeated until all the instances in the dataset have been used as test instances. LOOCV provides an accurate and unbiased evaluation of a model as every item in the dataset is used for testing. LOOCV was chosen to perform an exhaustive evaluation of the proposed model. LOOCV can be computationally expensive for large datasets, but the dataset used in this study is not large by machine learning standards.

**Figure 8:**
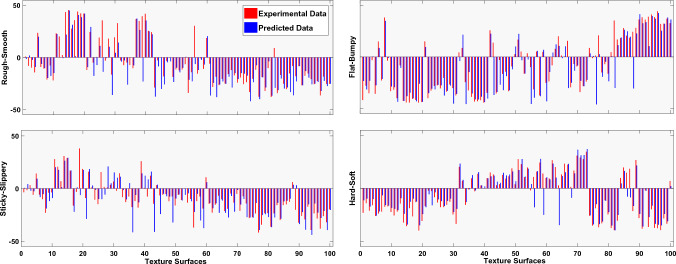
The attribute values from the psychophysical experiment are plotted alongside the attribute values predicted by the 1D-CNN.

The dataset described in “Texture dataset” section was used in LOOCV. The model was trained using 99 of the textures in the dataset, and the remaining one was used as a test set. The same process was repeated until all the textures were used as test sets. The point by point prediction results from LOOCV for the proposed model are provided in Fig. 8. Using LOOCV as an evaluation metric reduces the need to test the model on surfaces outside of the original. It must be noted that in every iteration the 100th instance is an unseen surface for the model as if the testing was being done with textures outside the original dataset.

**Figure 9:**
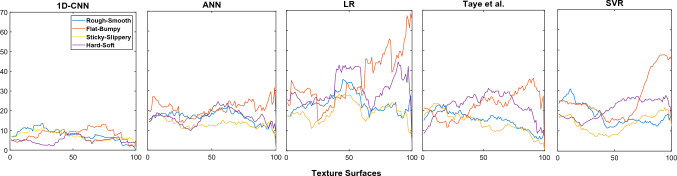
The mean absolute error (MAE) values for the proposed and four other algorithms.

Figure 8 shows the predicted value and the value assigned by human subjects to each of the texture surfaces. The predicted and experimental ratings are provided in the Supplementary Material for reference.  It can be seen that in most cases the prediction result is very close to the haptic value assigned by human subjects. In order to gain a better understanding of the prediction results, Mean Absolute Error (MAE) was calculated with a window size of 20, as shown in Fig. 9. MAE for all 100 surfaces is provided in Table 3. The MAE provides a more direct and intuitive summary of the prediction results. It can be seen that the max MAE (for an average of 20 surfaces) of 11.2 is achieved by Rough-Smooth towards the beginning of the curve. MAE for the other three attributes mostly stays below this value. It should be noted that MAE provided the absolute value of error and, thus, an error of 10 means 10 out of 100.

### Error comparison

In this section, the proposed algorithm is compared against other popular and state-of-the-art algorithms. These algorithms are linear regression, support vector regression, artificial neural net, and state-of-the-art 1D CNN^57^. Note that, linear regression and support vector regression algorithms are used from the Scikit-learn machine learning library. On the other hand, ANN is designed with two fully connected (FC) layers and a regression output layer. The FC layers have 200 and 100 nodes, respectively. To train the ANN, an ADAM optimizer is used along with the MSE loss function. The same LOOCV evaluation strategy was used for all four algorithms to keep the comparison fair. Table 4 shows the root mean square error (RMSE) for the four algorithms and the proposed algorithm. The Root Mean Square Error (RMSE) is measured as follows.

3 $$\begin{aligned} RMSE = \sqrt{\frac{1}{N}\sum _{i=1}^{N}(y_{i}-\bar{y_{i}})^{2}} \end{aligned}$$

where $\overline{y}_\text {i}$ represents the actual rating of the ith sample, y$_\text {i}$ denotes the predicted rating and N is the total samples. This experiment shows that linear regression has an RMSE of 29.9, 57.05, 25.04, and 42.18 for Rough-Smooth (R-S), Flat-Bumpy (F-B), Sticky-Slippery (S-S) and Hard-Soft (H-S), respectively. On the other hand, ANN has an RMSE of 20.41, 30.52, 16.74 and 20.29 for R-S, F-B, S-S, and H-S, respectively. However, SVR shows better RMSE for F-B and S-S compared to the other existing algorithms.

**Table 3:** The mean absolute error (MAE) values for the proposed system and four other algorithms.

	R-S	F-B	S-S	H-S
Linear regression	23.11	32.43	19.17	30.21
Support vector regression	17.81	23.85	12.94	20.28
Artificial neural network	16.96	20.62	13.31	16.59
1D CNN (Taye et al.^57^)	15.57	22.16	14.43	21.44
Proposed 1D-CNN	**8.13**	**8.47**	**7.12**	**5.15**

The values are written for each of the four attribute pairs.

Bold represent the best results.

**Table 4:** The root mean square error (RMSE) values for the proposed system and four other algorithms.

	R-S	F-B	S-S	H-S
Linear regression	29.9	57.05	25.04	42.18
Support vector regression	22.78	26.38	15.97	21.46
Artificial neural network	20.41	30.52	16.74	20.29
1D CNN (Taye et al.^57^)	20.79	27.70	19.70	26.59
Proposed 1D-CNN	**13.39**	**14.30**	**9.59**	**7.91**

The values are written for each of the four attribute pairs.

Bold represent the best results.

From Table 4 it can be observed that in most cases, the deep learning algorithm (i.e., 1D CNN) proposed in^57^ failed to produce lower RMSE (see Table 4) than the simple machine learning algorithms (i.e., ANN). This is because the 1D CNN^57^ applies kernels with larger window sizes; therefore, this 1D CNN fails to capture the local features adequately. Besides, the number of kernels applied to the 1D CNN^57^ is also fewer compared to the proposed multi-scale 1D-CNN. Capturing the local spatial information in different scales as well as utilizing a large number of kernels helps to boost the performance of our model. Hence, the proposed algorithm has significantly lower RMSE values as compared to the other four algorithms.

**Table 5:** The RMSE of each individual feature in comparison with the concatenated features.

	R-S	F-B	S-S	H-S
GLCM	17.91	14.51	15.21	10.81
LBP	18.92	19.16	16.91	11.50
ResNet-50	18.62	15.26	19.00	10.40
Concatenated features	**13.39**	**14.30**	**9.59**	**7.91**

Bold represent the best results.

### Individual feature error

In the aforementioned comparisons, the proposed model was trained using a concatenated 1D vector comprising ResNet-50, LBP, and GLCM features. In this subsection, the three features were individually used to train the 1D-CNN model and predict the output. This exercise was conducted to figure out the individual accuracy of each feature and whether a single feature could perform better than the concatenated version.

Table 5 shows that the model trained with feature concatenation provided the highest accuracy as compared to the individual features. This result was expected, as the ResNet-50 captures higher level spatial information, while LBP and GLCM focus on micro level spatial arrangement of texture. The concatenated features had the advantage of using both higher level and micro level information for predicting the attributes of textures and thus performed better than the individual features.

## Discussion

Figure 9 shows that different attribute pairs perform differently for certain texture surfaces. The MAE plot of the 1D-CNN model shows that the R-S attribute pair has the highest MAE value among all others for the first 25 textures, and the F-B attribute pair performs the worst for textures 50 to 85. Similarly, the best attribute pair in MAE plot of 1D-CNN for the first 50 textures turns out to be H-S, while for the last 50 textures, three attribute pairs (R-S, S-S, H-S) predict with similar accuracy.

As mentioned earlier, the prediction error for the R-S attribute pair is relatively higher for the first 25 texture surfaces. A majority of these textures are polished surfaces, as seen in Fig. 2. It is probable that the camera used in this study could not capture images detailed enough to fully encapsulate the micro geometry of the surfaces. The prediction values for the F-B attribute pair for textures 74 to 83 have a very high error rate. It can be seen from Fig. 2 that all these surfaces are sandpapers with different grit ratings (80 to 3000). The algorithm predicted their attribute values based on the image features. However, it is highly likely that the participants judged these surfaces as sandpapers only without going into too much detail about the texture itself. This phenomenon is called as pre-judgment, where a participant judges the haptic qualities of a texture based on their past experience rather than the textures available at the time. Pre-judgment is defined in greater detail in^58^. The fact that the MAE is shown in terms of a moving window of 20 surfaces may have resulted in the outliers affecting the average. MAE for all the 100 surfaces is available in Table 3, and it can be seen that the highest MAE is 8.47 for F-B.

The exact value for human JND (Just noticeable difference) for haptic attributes of real textures is unknown. However, earlier research^59^ showed that perceptual similarity boundaries extend a fair distance beyond a given surface in the perceptual space. In order to check how much of an error is negligible in perceptual attributes, we calculated the standard deviation of haptic attributes across all participants. The standard deviation for each texture was calculated separately and then averaged across all 100 textures to get one single standard deviation value for all textures across all participants. The mean standard deviation for R-S was 15.14, F-B was 13.48, S-S was 16.90, and H-S was 16.77. It can be assumed that a prediction error less than the standard deviation can be considered as perceptually similar. The highest MAE, as mentioned earlier, was 8.47 in this study which is less than the mean standard deviation, therefore, the overall prediction error can be considered perceptually negligible.

In Fig. 4 the four quadrants in each plot represent specific types of texture surfaces. For instance, in the first plot of HAS (Rough-Smooth and Flat-Bumpy), the first quadrant has smooth and bumpy surfaces, the second quadrant represents rough and bumpy surfaces, the third quadrant contains rough and flat surfaces, and the fourth quadrant is populated with smooth and flat surfaces. It is intuitive to assume that the rough and bumpy, and smooth and flat are densely populated as these attributes often occur simultaneously. The rough and flat is also well populated, however, most of the surfaces are close to the origin. This shows that some flat surfaces were perceived as mildly rough, for example, high grit sandpapers or some wooden surfaces. The least populated quadrant is the smooth and bumpy one. The current dataset contained very few textures that could represent these two attributes. The two surfaces in this quadrant are the ones with clearly perceivable bumps on an otherwise smooth surface.

A similar pattern can be seen in the third and fourth dimensional plots of HAS in Fig. 4. The first quadrant contains slippery and soft surfaces and is the least populated. The second quadrant has sticky and soft surfaces, the third one contains hard and sticky surfaces, and the fourth quadrant contains slippery and hard surfaces. The second and third quadrants are the most populous quadrants which means that a high number of surfaces were perceived as more sticky as compared to slippery. It can be seen that in the second quadrant, the surfaces do not reach extreme values and are rather situated more towards the origin. It can be argued that we do not encounter such surfaces in most of our daily life interactions. Some examples can be organic surfaces (chewing gum, clay, etc) or silicone, which are not a part of this texture dataset. The third quadrant consists of sticky and hard surfaces. The extremes in this quadrant are some sandpapers and metallic meshes. The fourth quadrant contains slippery and hard surfaces. This quadrant incorporates metals or polished hardwood surfaces in the extreme.

The dataset used in this study primarily consists of common office and household materials, with mostly uniform textures. However, in real-life scenarios, we encounter a wide range of surfaces that are not represented in the current study. These could include organic surfaces, oily or wet surfaces, and surfaces with artificial patterns, among others. As a result, it can be said that the current library covers only a portion of the overall haptic space of textures. It is important to consider this when evaluating new surfaces; if we test a surface that belongs to the same portion of the haptic space as the dataset, the haptic attributes will be predicted accurately. However, if we test a surface that lies far away from the dataset in the haptic space of textures, the predicted haptic attributes may not be perceptually correct for that particular surface.

Expanding on the aforementioned *subset of the overall haptic space*, a natural question would be to ask if the given 100 textures sufficiently represent the overall convex hull that covers this specific subset. Does the perceptual space of the 100-texture dataset offer a comprehensive representation, or would it evolve upon the addition of more similar textures? To investigate this, a short analysis of building a stepwise MDS (10 textures at a time) was carried out. The MDS from the initial two batches of 10 textures exhibited randomly scattered textures. The shape of the perceptual appeared after the next two batches, i.e., a total of 40 textures. The remaining textures only helped in filling in the shape, leading to negligible changes to the overall perceptual space. Moreover, a subset of the 100 textures, i.e., 84 textures, was used in another study^59^ to establish a perceptual space. The perceptual space was clustered into groups of perceptually similar textures. These clusters were enclosed in convex hulls, and it was noticed that adjacent convex hulls frequently overlapped. The overlapping perceptual convex hulls indicate that participants found it difficult to perceptually distinguish the textures. Therefore, it can be reasonably inferred that the 100-texture perceptual space is considerably dense and complete.

Incorporating new textures into the HAS is based on their image features. This emphasizes the image capturing setup and the quality of the image being captured. It is a well-known fact that better quality images lead to better image features. The algorithm (1D-CNN) can also predict haptic attributes with a lower error if the image features are well collected and correctly capture the micro and macro texture information of a surface. It is of utmost importance that the image is captured without shadows, the texture should be clearly visible (not blurred), and with high resolution.

## Conclusions

In this paper, we established a four-dimensional Haptic Attribute Space (HAS) from psychophysical experiments. The axes of the 4D HAS are haptic attributes of texture that were chosen by participants to best represent the 100 textures used in this study. The 100 textures are then scattered in the 4D HAS according to their corresponding attribute values. In order to populate the HAS with new textures, a multi-scale 1D-CNN was trained to predict haptic attributes of texture based on their image features. The HAS in combination with the multi-scale 1D-CNN provides a universal space where all textures can be represented based on their attribute values. This provides an intuitive way to classify or identify textures based on their images, without the need to physically interact with them.

The current study captures image features from images entirely containing textures. In order to deploy the advantages of the proposed system in the real or virtual world, the overall scene needs to be segmented into distinct texture regions. These texture segments can then be fed into the proposed system to predict the haptic attributes of the various elements in the environment (Supplementary Information).

## Supplementary Information

Supplementary Information 2.

Supplementary Information 1.

## Data Availability

The image dataset and the adjective ratings used during the current study are included in this published article and its supplementary information files. Code is available from the first or corresponding author upon reasonable request.
