# Supplementary figures and images for: Establishing haptic texture attribute space and predicting haptic attributes from image features using 1D-CNN
Source: Sci Rep. 2023 Jul 19;13:11684. doi: 10.1038/s41598-023-38929-6 (PMC10356925; doi:10.1038/s41598-023-38929-6)

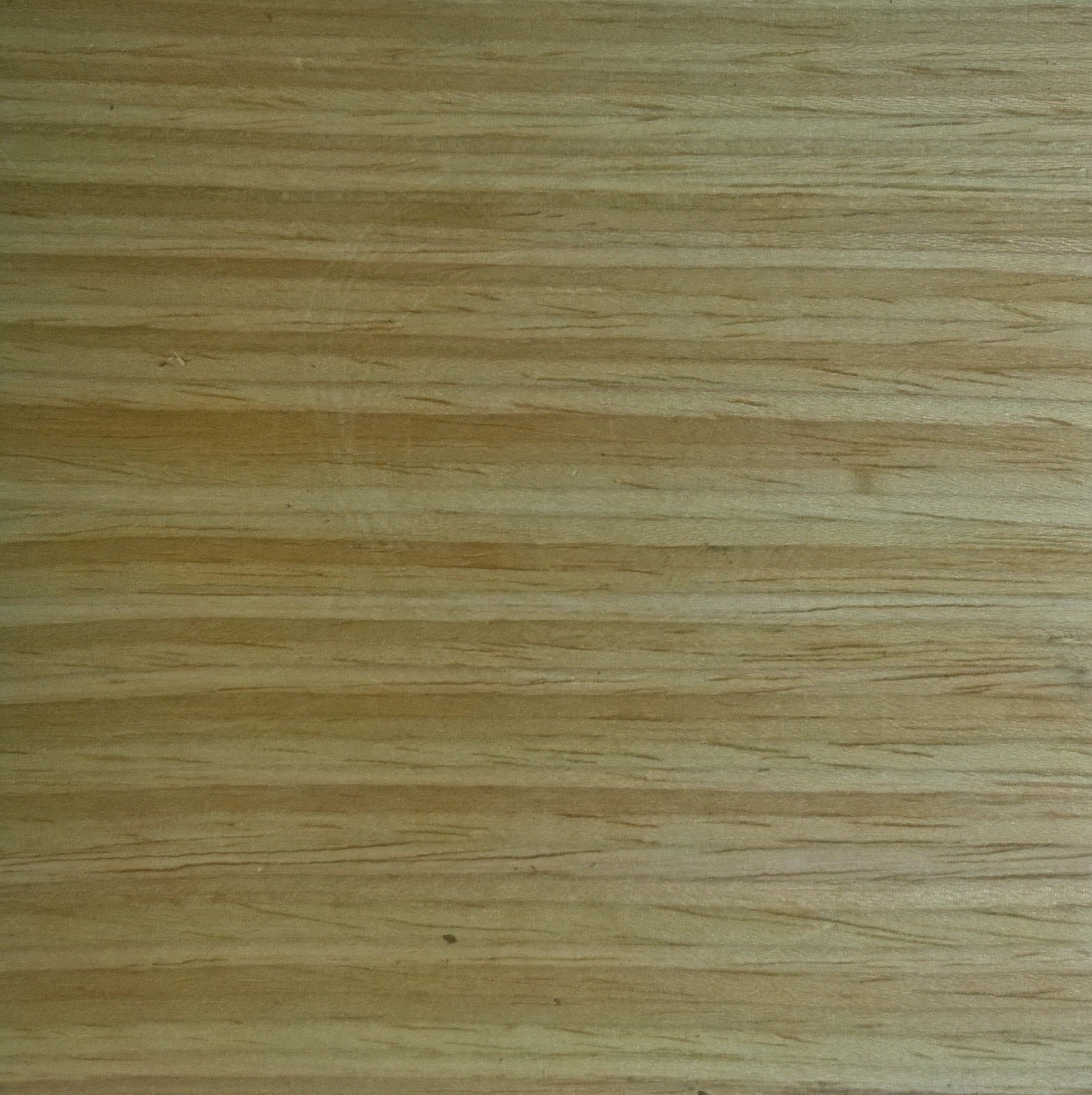

Supplement: Supplementary file 1 — Supplementary Information 2. [file 41598_2023_38929_MOESM1_ESM.zip › 1.jpg]

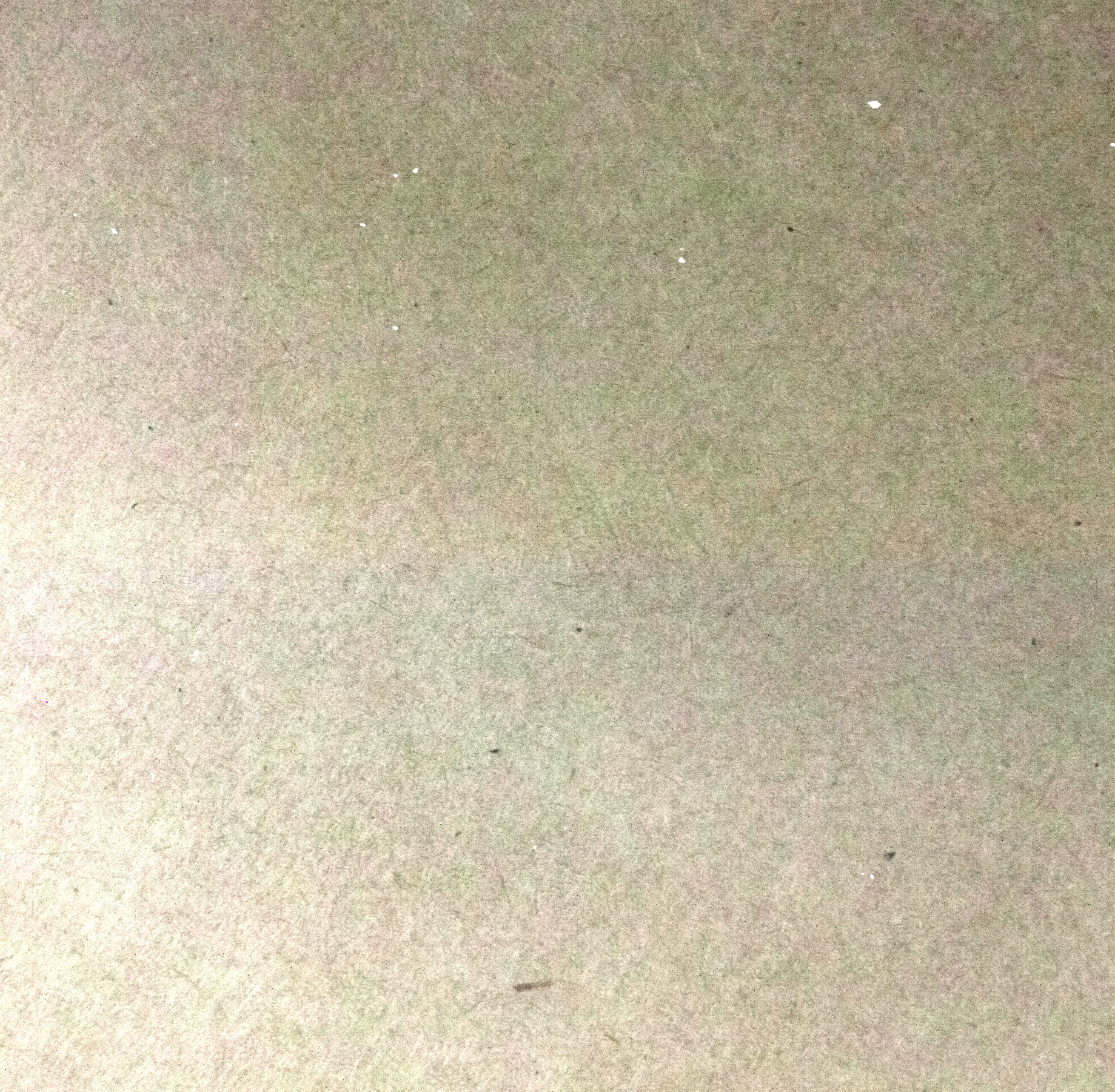

Supplement: Supplementary file 1 — Supplementary Information 2. [file 41598_2023_38929_MOESM1_ESM.zip › 10.JPG]

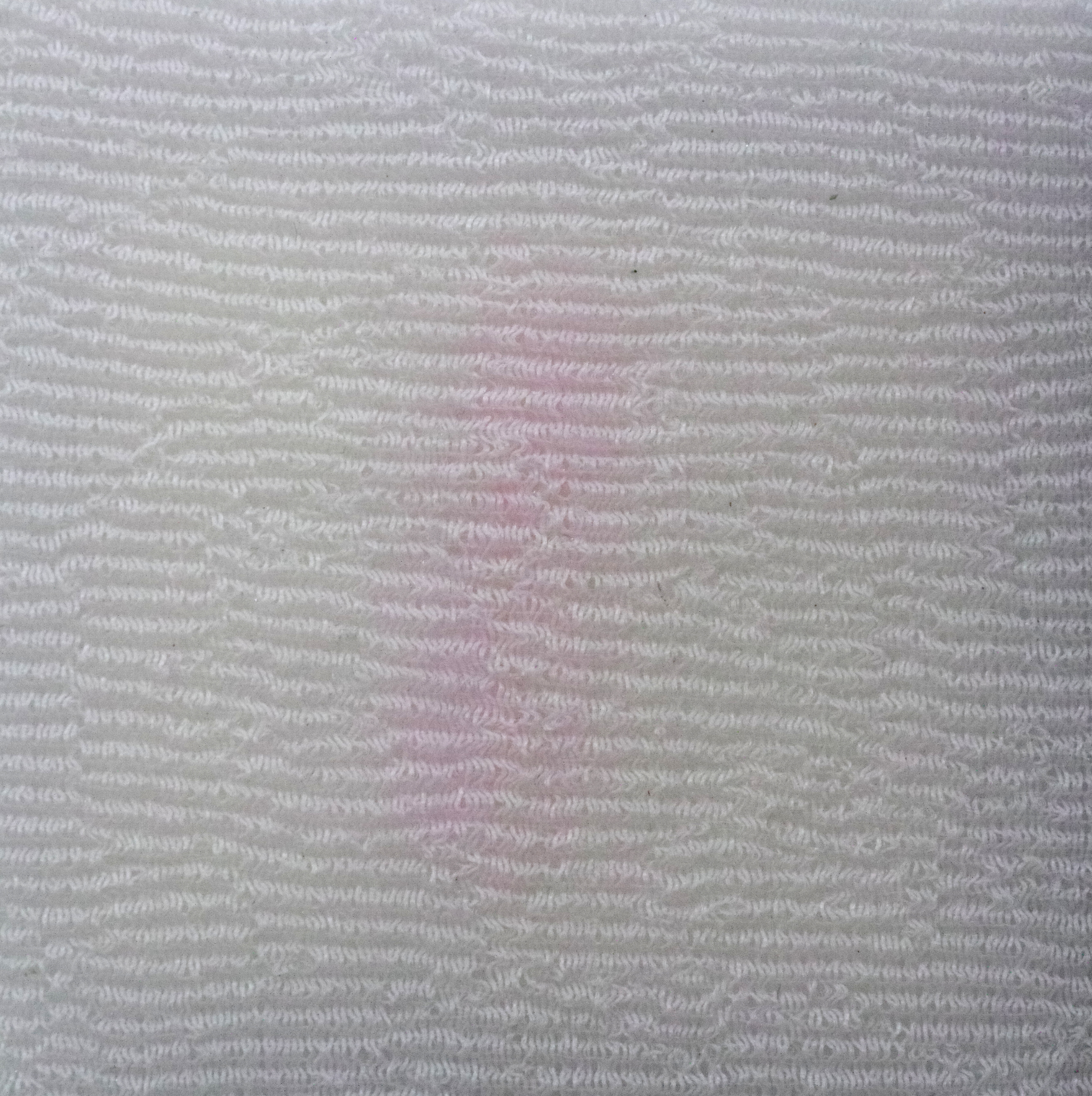

Supplement: Supplementary file 1 — Supplementary Information 2. [file 41598_2023_38929_MOESM1_ESM.zip › 100.JPG]

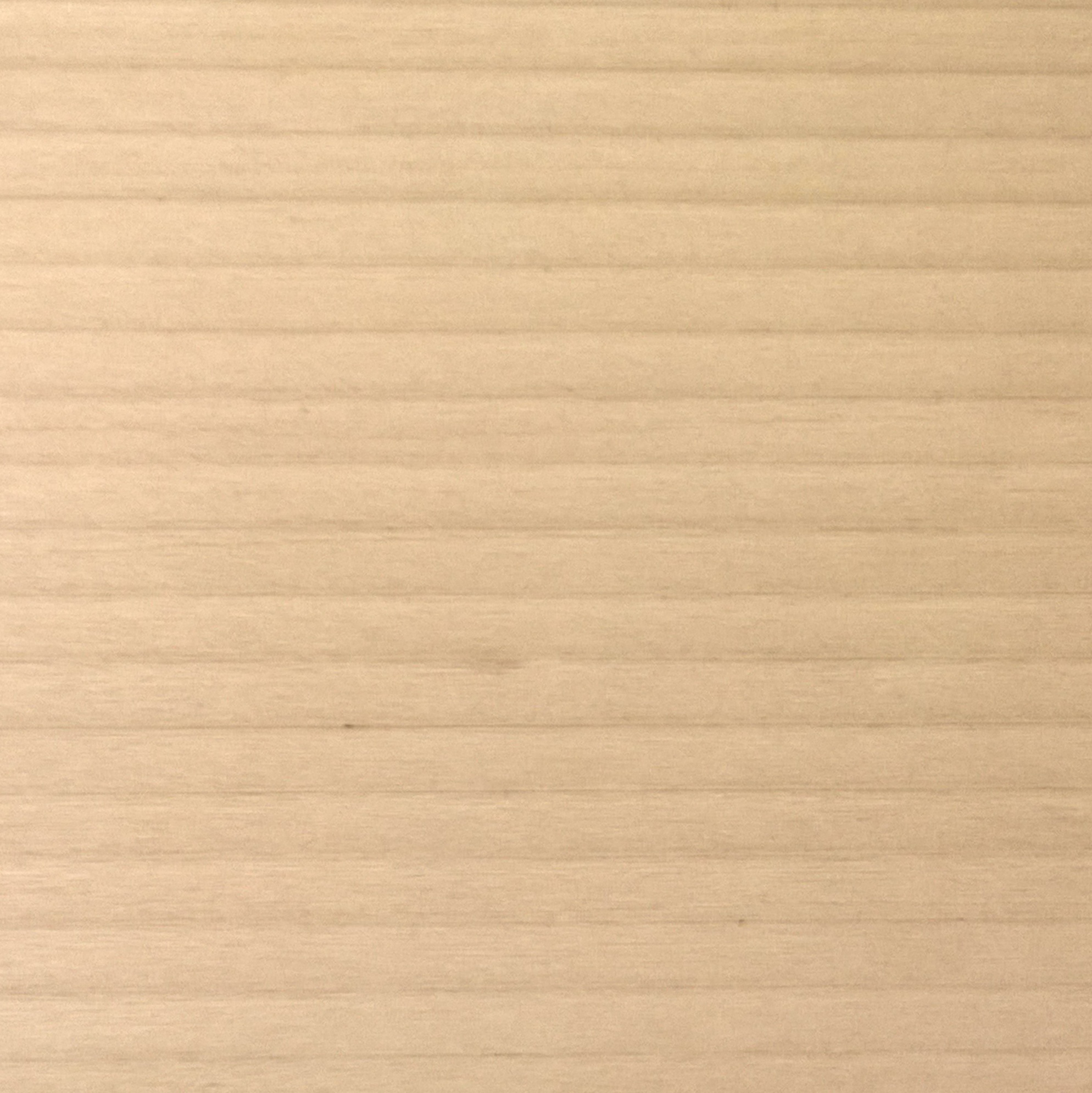

Supplement: Supplementary file 1 — Supplementary Information 2. [file 41598_2023_38929_MOESM1_ESM.zip › 11.jpg]

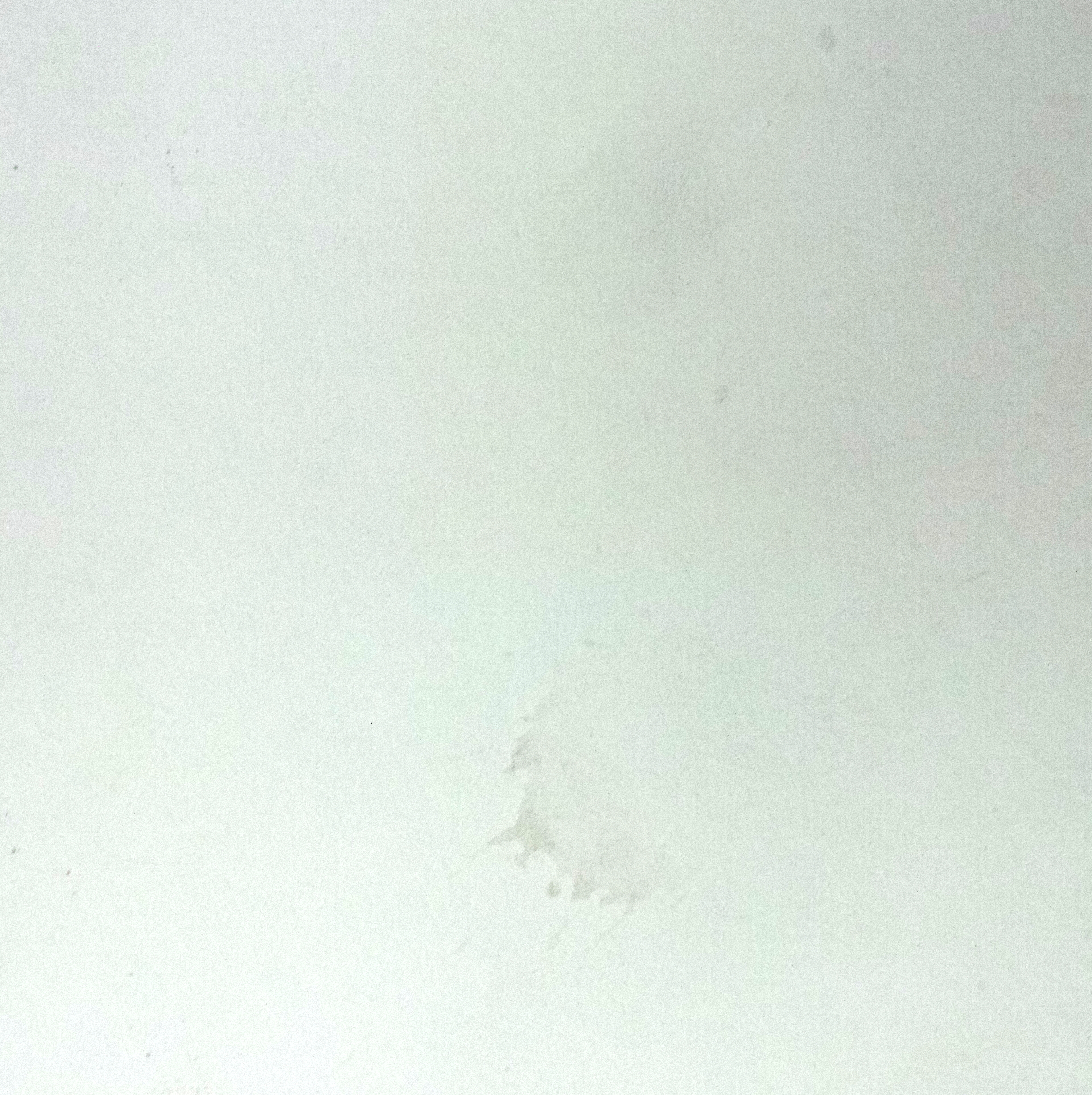

Supplement: Supplementary file 1 — Supplementary Information 2. [file 41598_2023_38929_MOESM1_ESM.zip › 12.JPG]

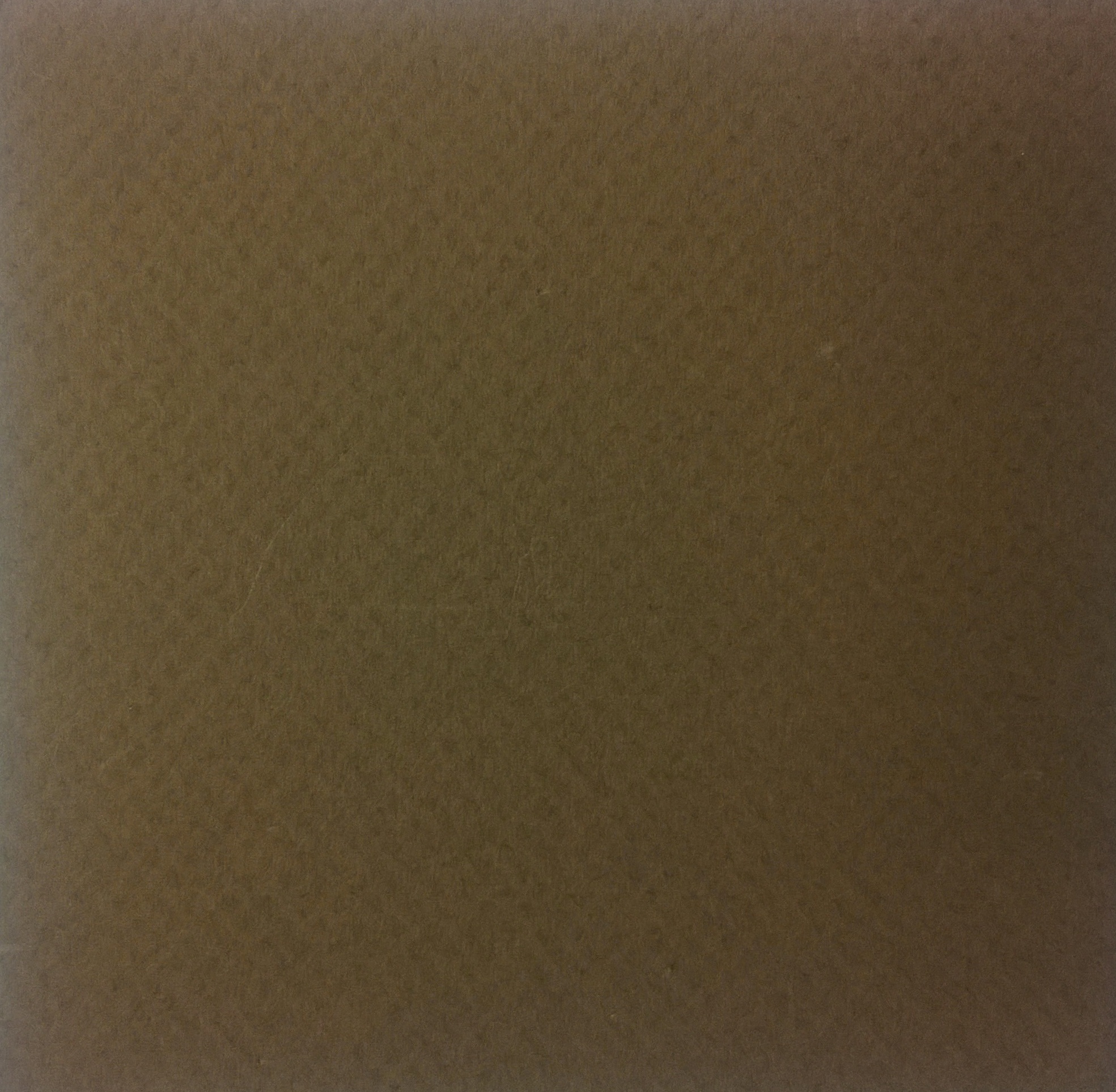

Supplement: Supplementary file 1 — Supplementary Information 2. [file 41598_2023_38929_MOESM1_ESM.zip › 13.jpg]

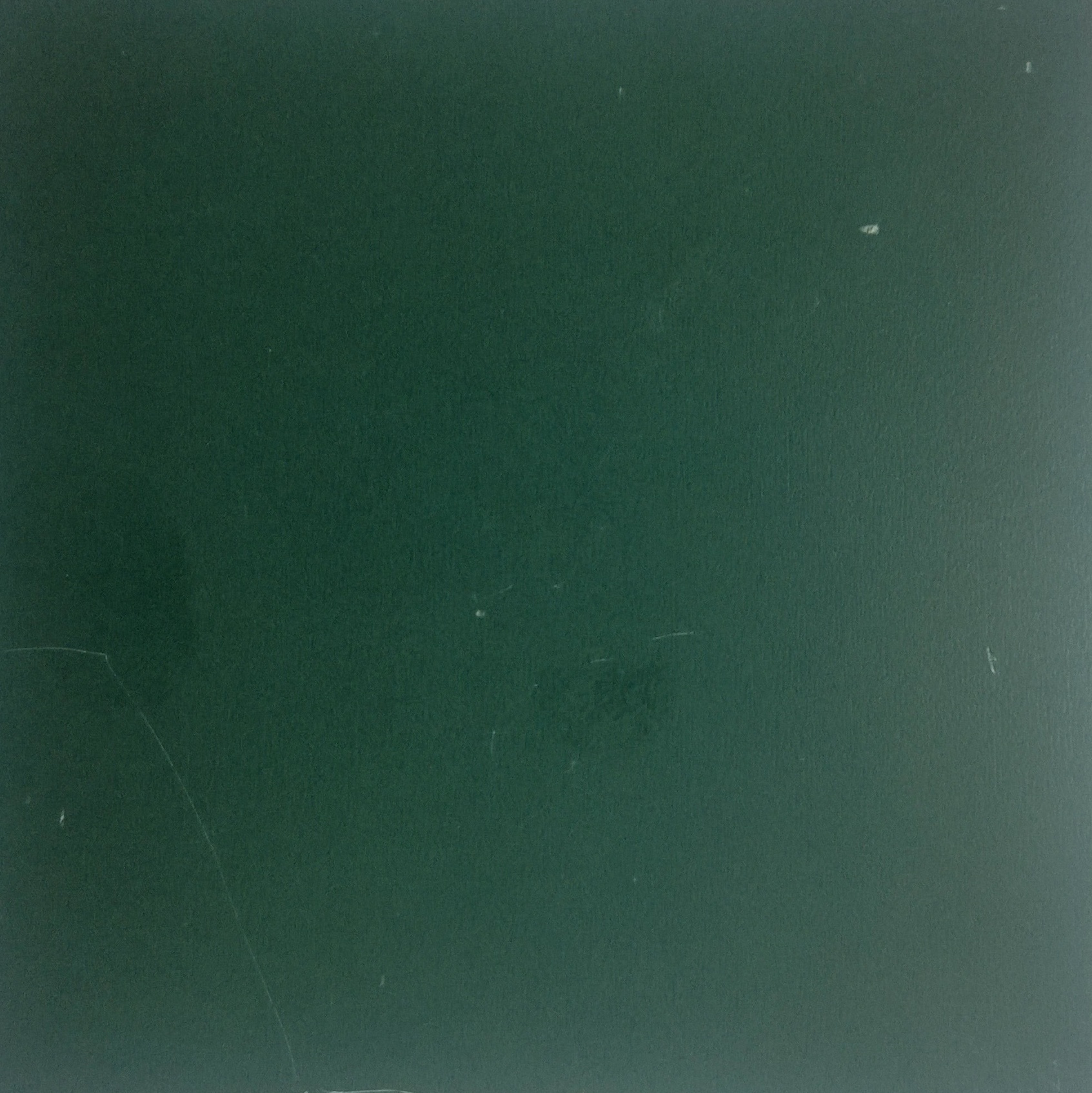

Supplement: Supplementary file 1 — Supplementary Information 2. [file 41598_2023_38929_MOESM1_ESM.zip › 14.jpg]

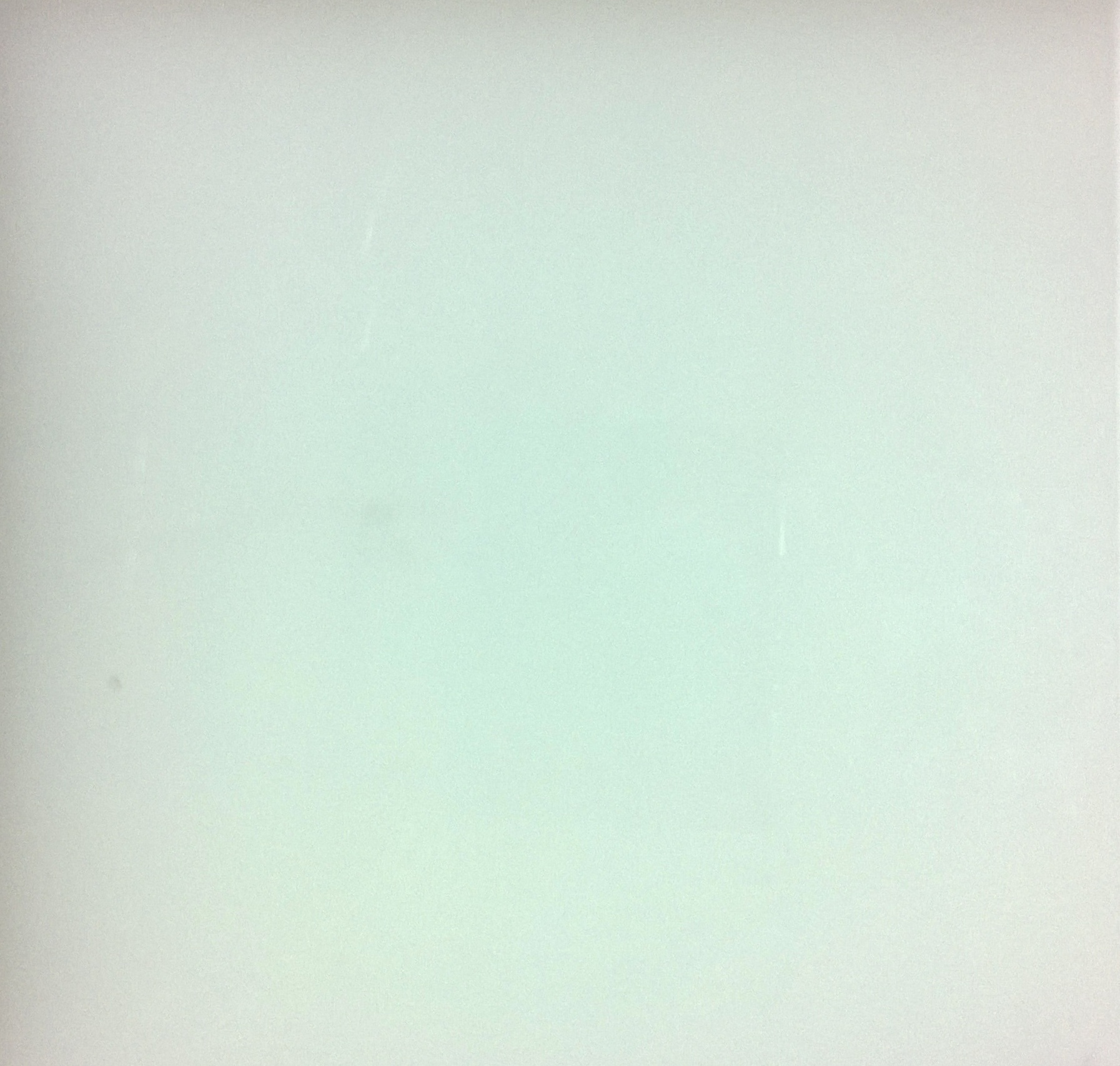

Supplement: Supplementary file 1 — Supplementary Information 2. [file 41598_2023_38929_MOESM1_ESM.zip › 15.jpg]

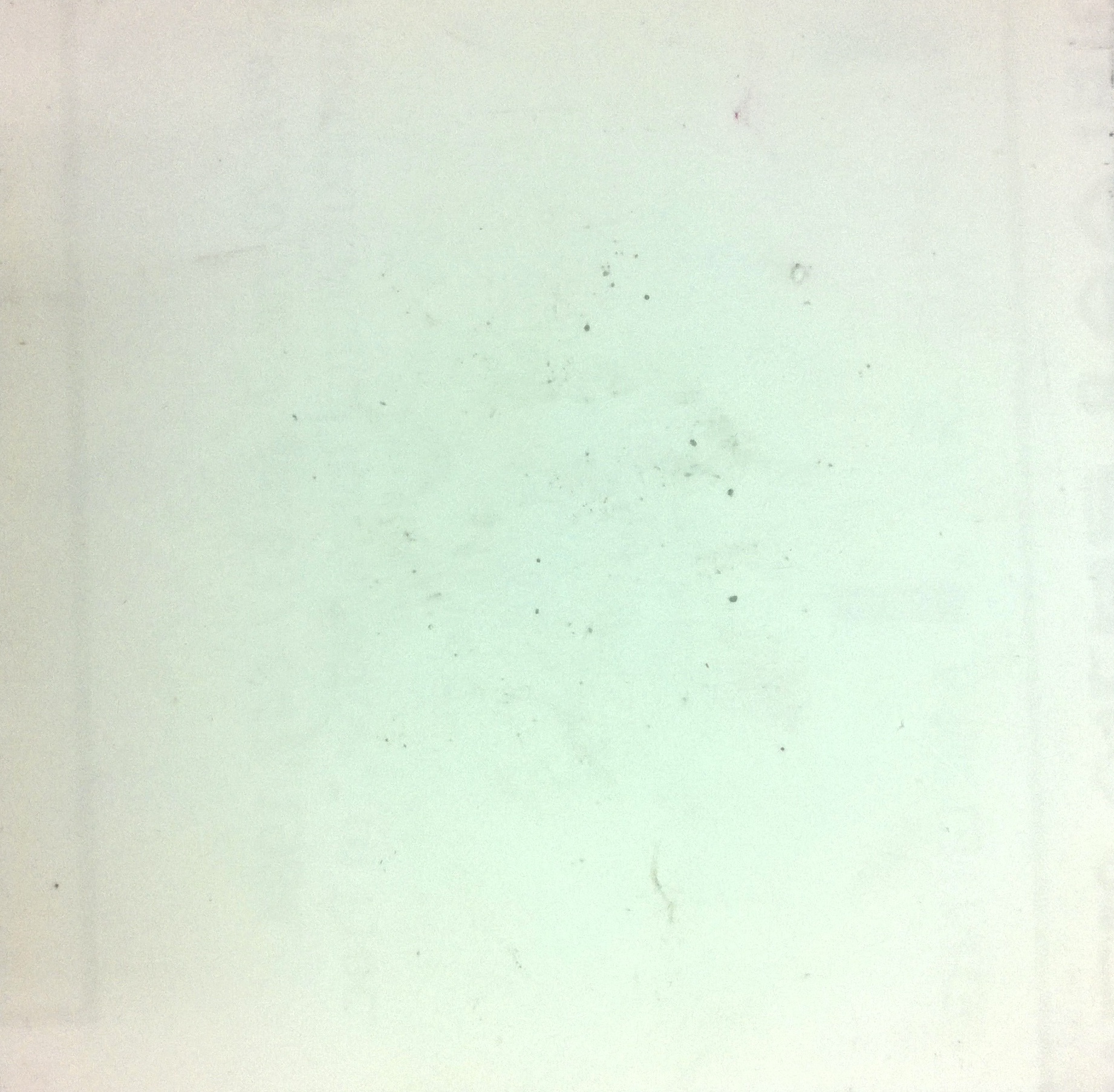

Supplement: Supplementary file 1 — Supplementary Information 2. [file 41598_2023_38929_MOESM1_ESM.zip › 16.jpg]

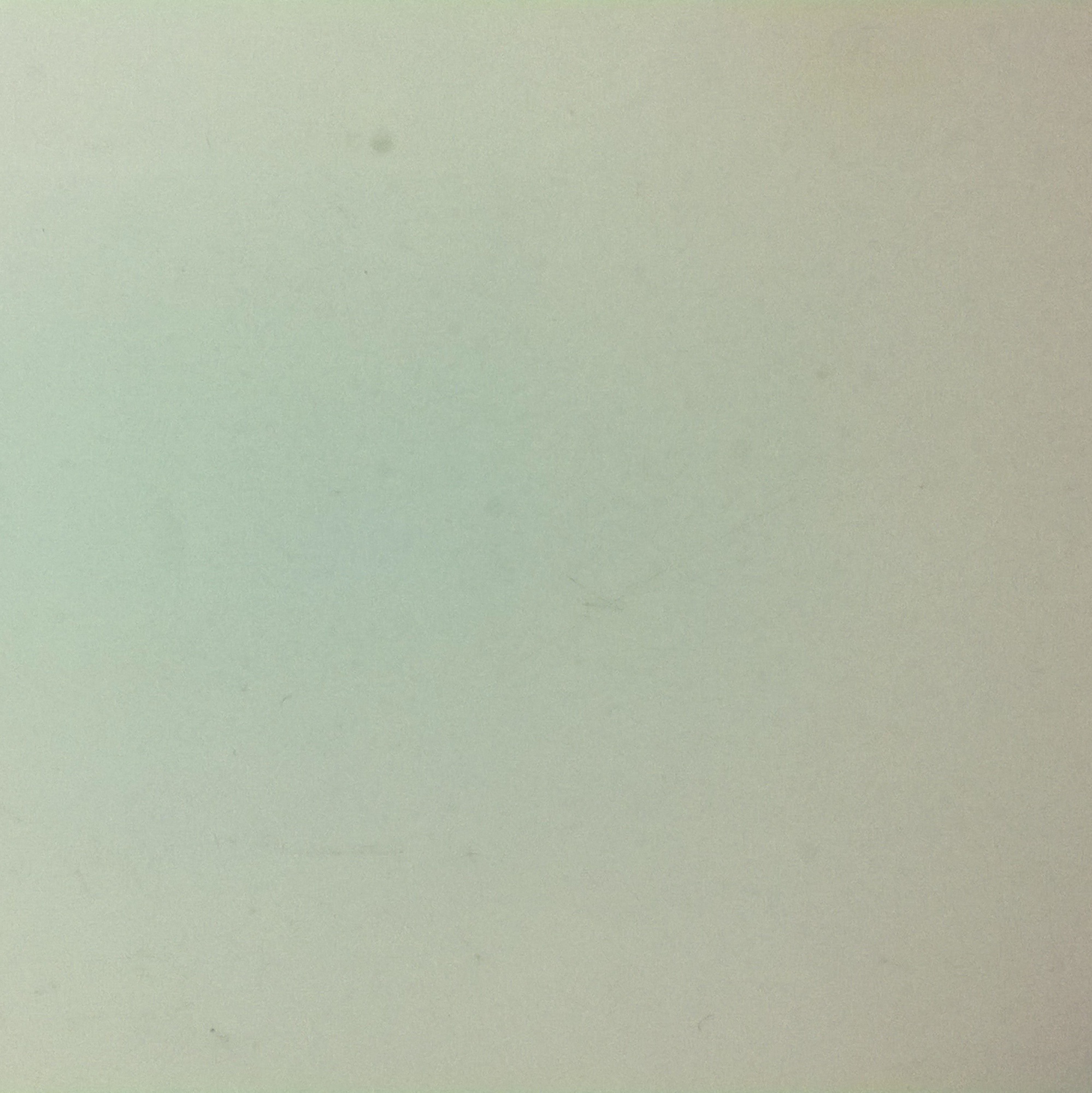

Supplement: Supplementary file 1 — Supplementary Information 2. [file 41598_2023_38929_MOESM1_ESM.zip › 17.jpg]

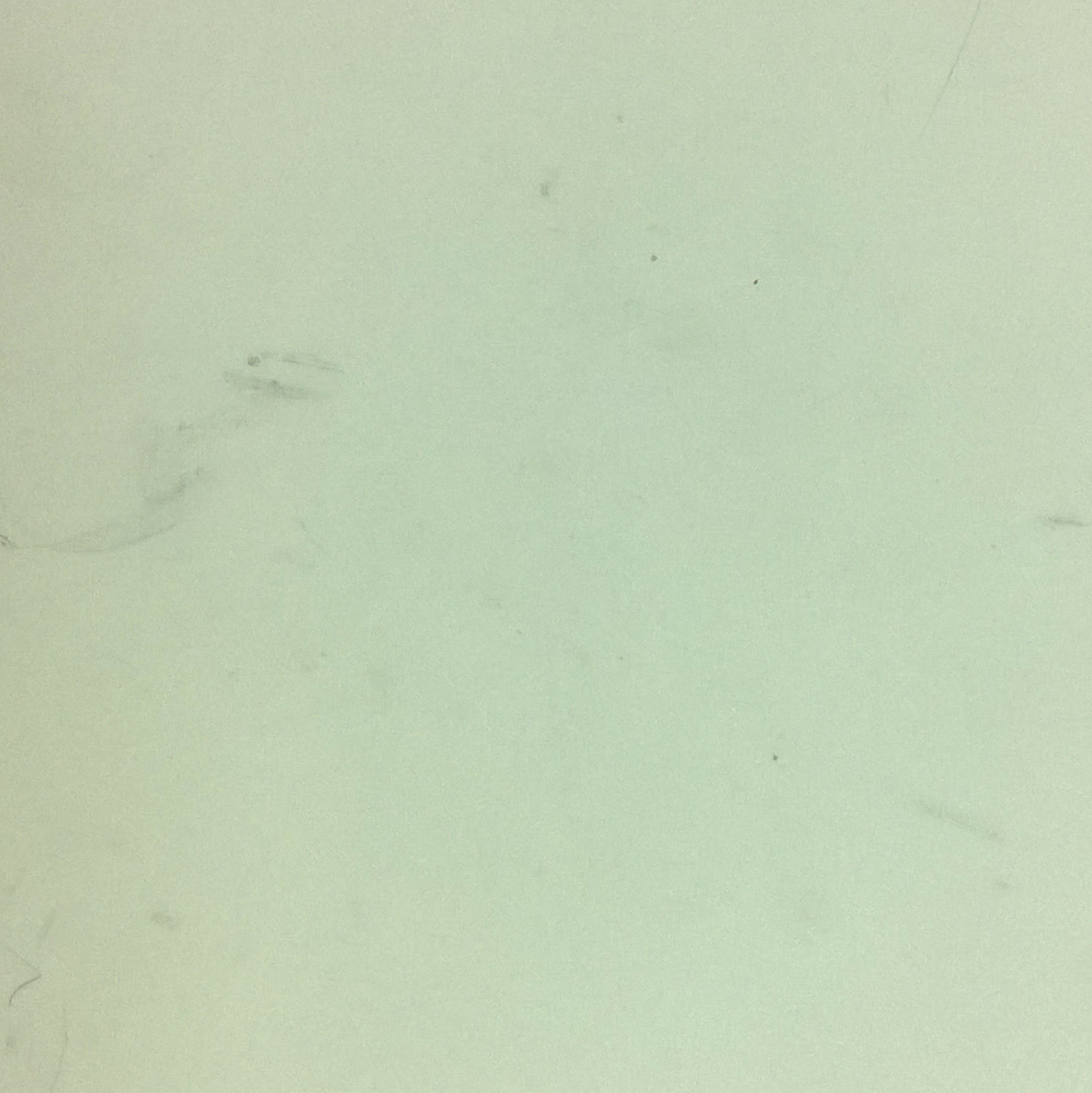

Supplement: Supplementary file 1 — Supplementary Information 2. [file 41598_2023_38929_MOESM1_ESM.zip › 18.jpg]

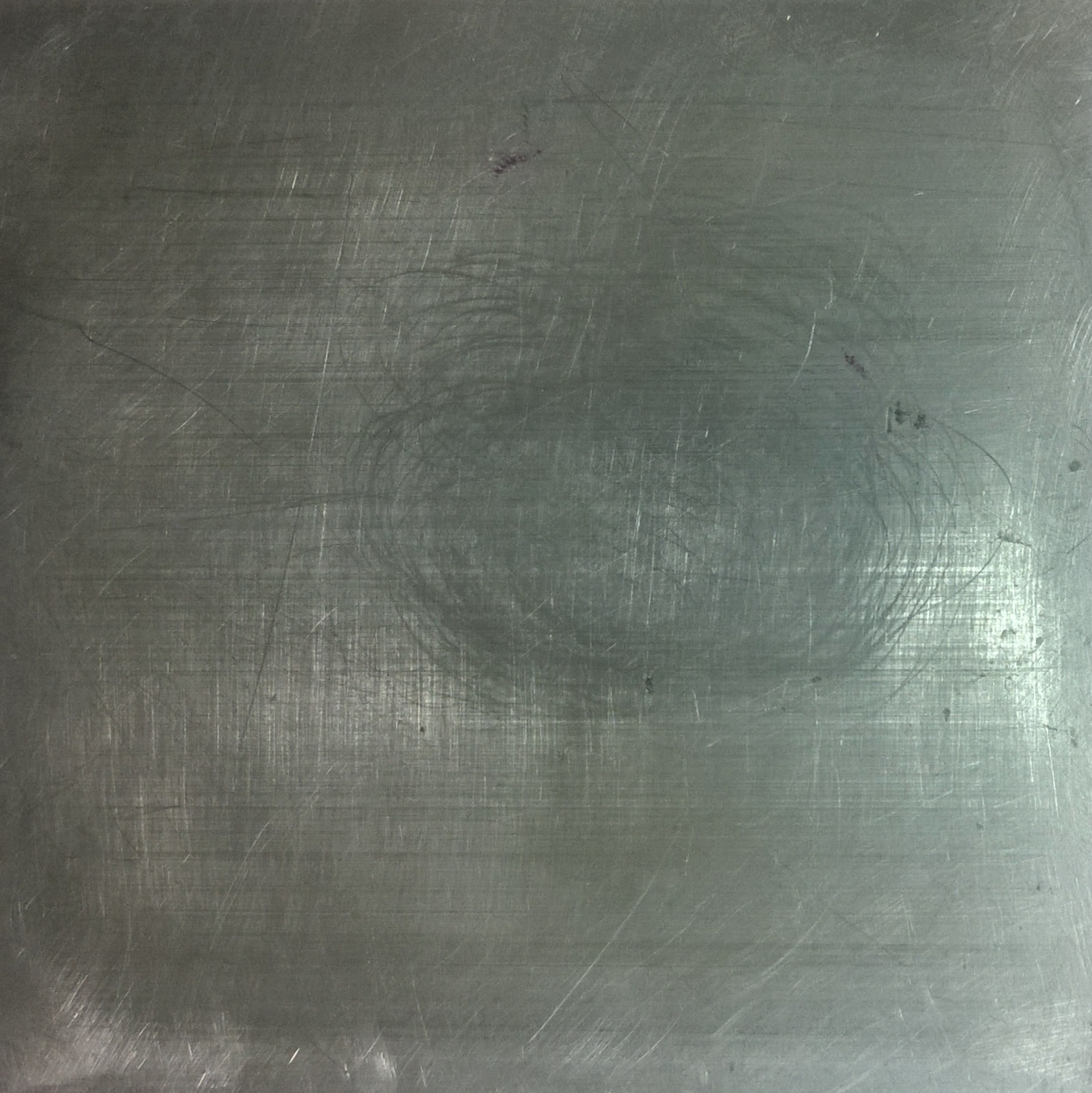

Supplement: Supplementary file 1 — Supplementary Information 2. [file 41598_2023_38929_MOESM1_ESM.zip › 19.jpg]

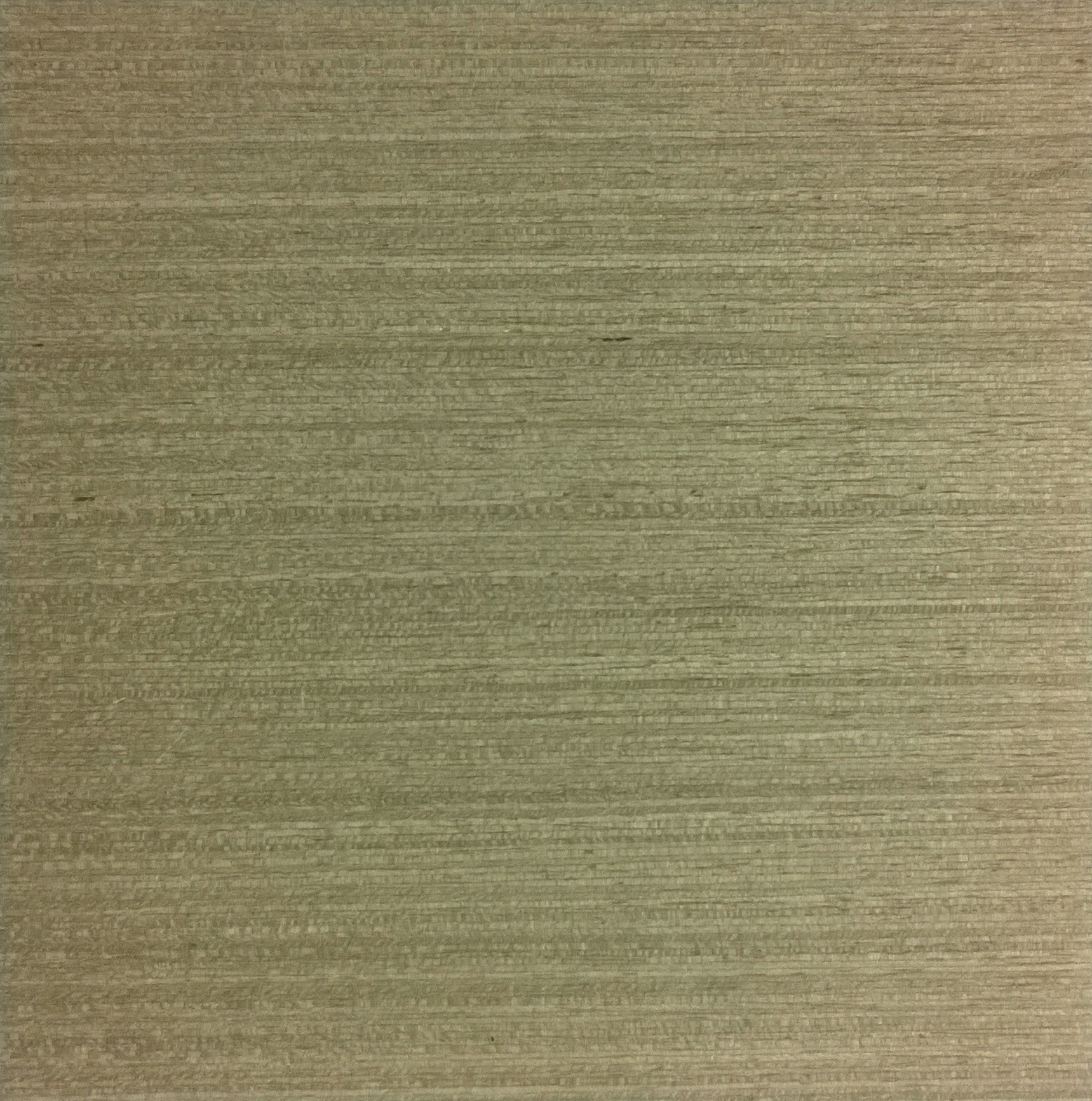

Supplement: Supplementary file 1 — Supplementary Information 2. [file 41598_2023_38929_MOESM1_ESM.zip › 2.jpg]

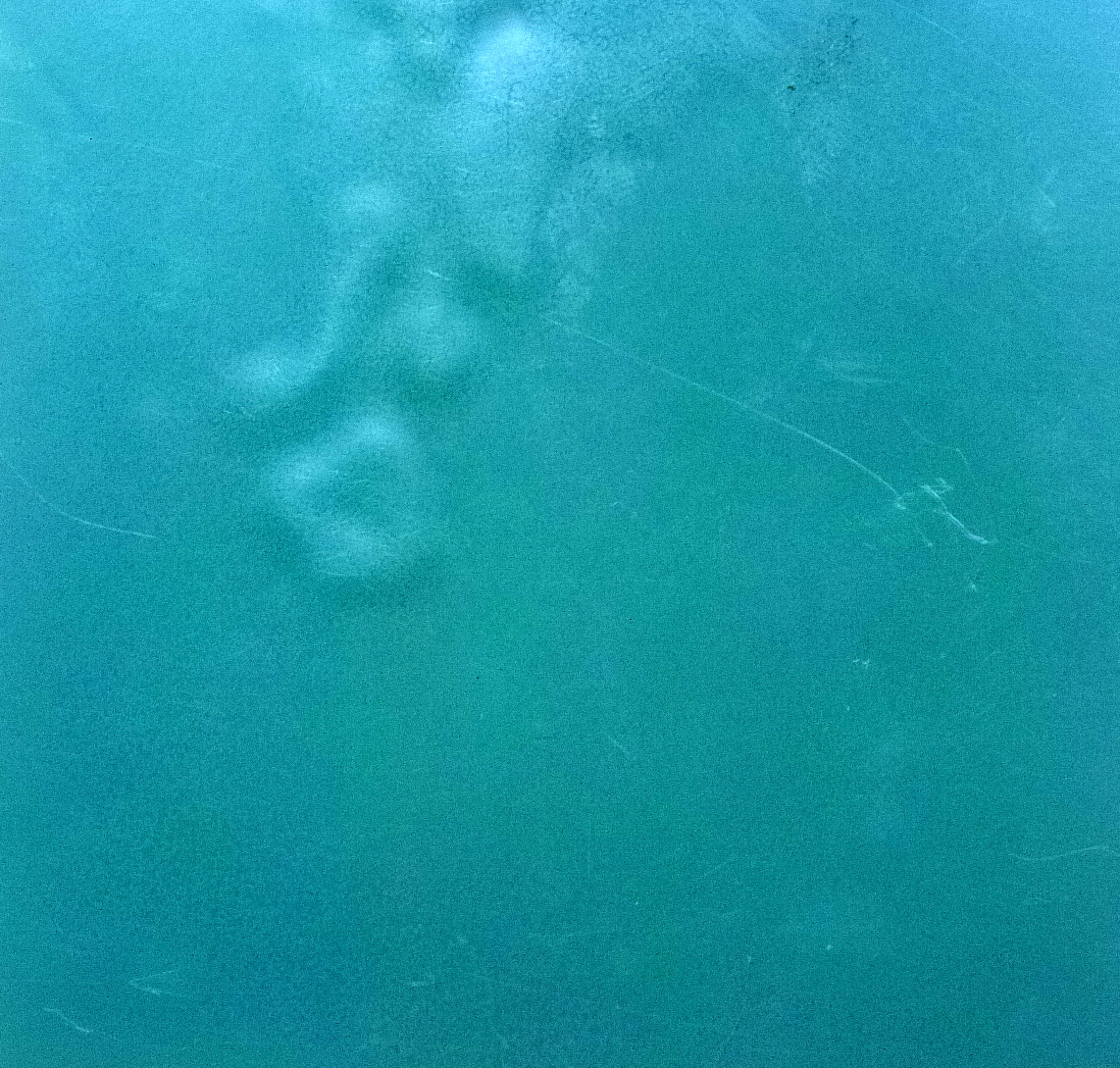

Supplement: Supplementary file 1 — Supplementary Information 2. [file 41598_2023_38929_MOESM1_ESM.zip › 20.JPG]

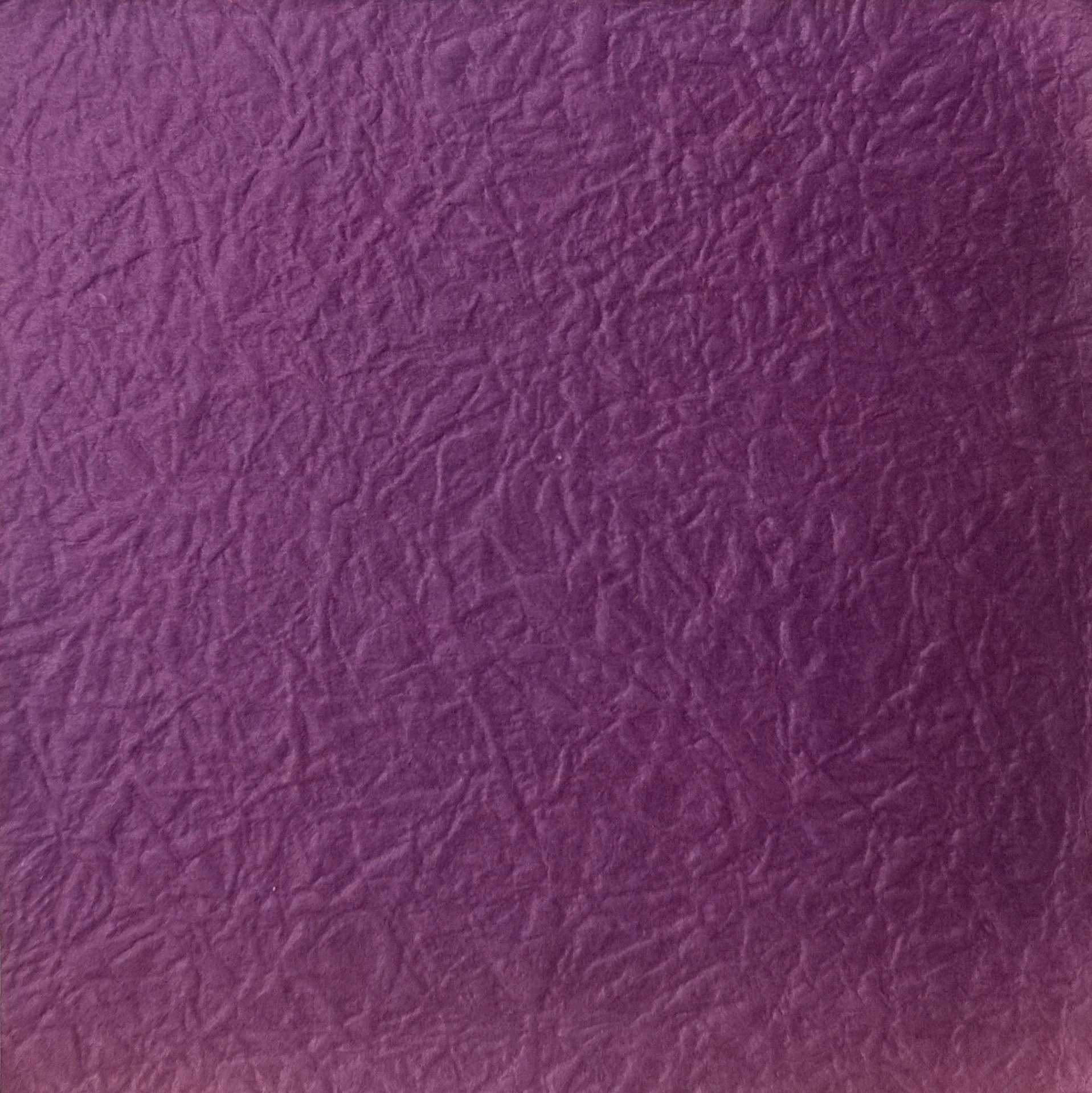

Supplement: Supplementary file 1 — Supplementary Information 2. [file 41598_2023_38929_MOESM1_ESM.zip › 21.jpg]

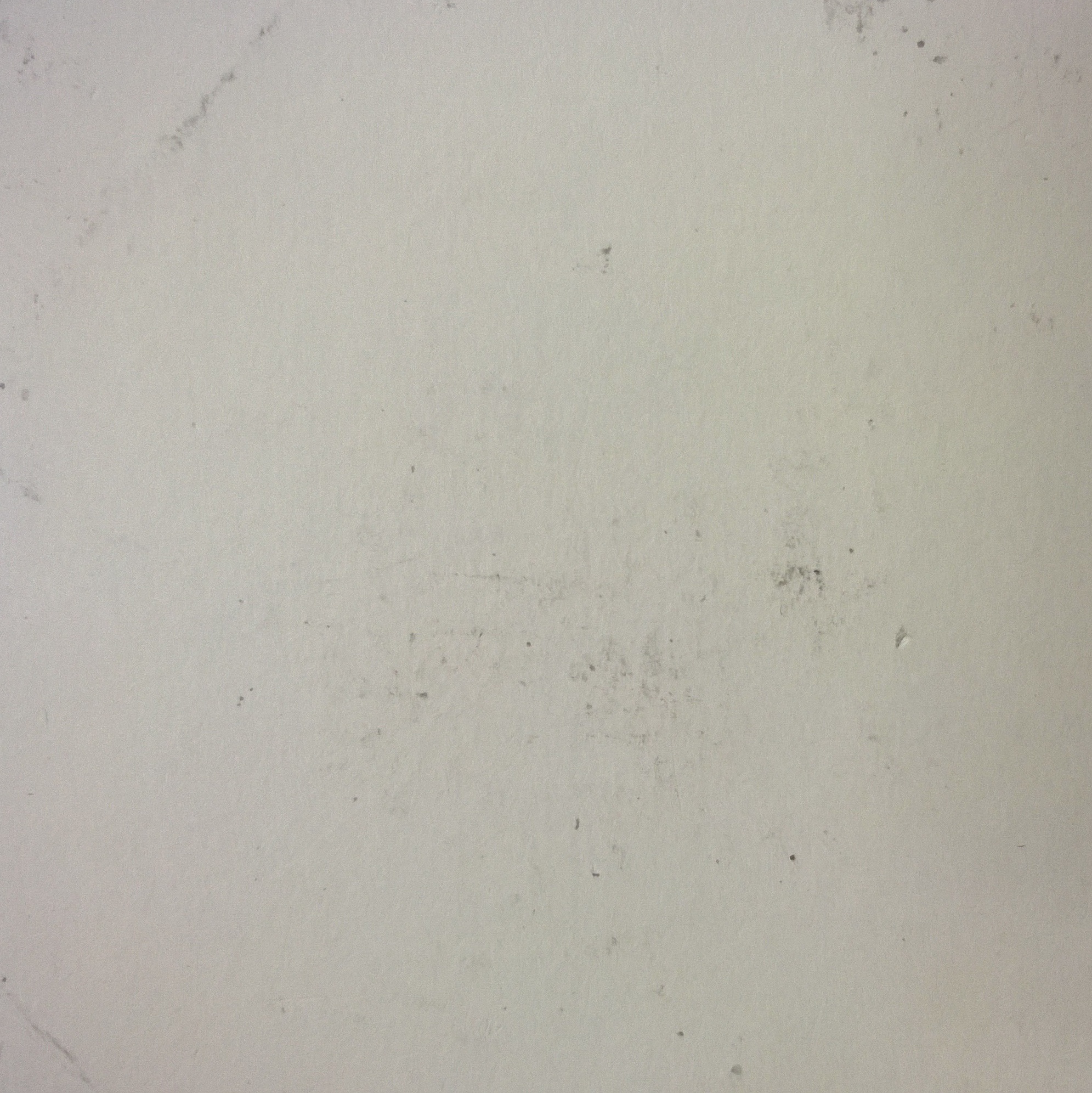

Supplement: Supplementary file 1 — Supplementary Information 2. [file 41598_2023_38929_MOESM1_ESM.zip › 22.jpg]

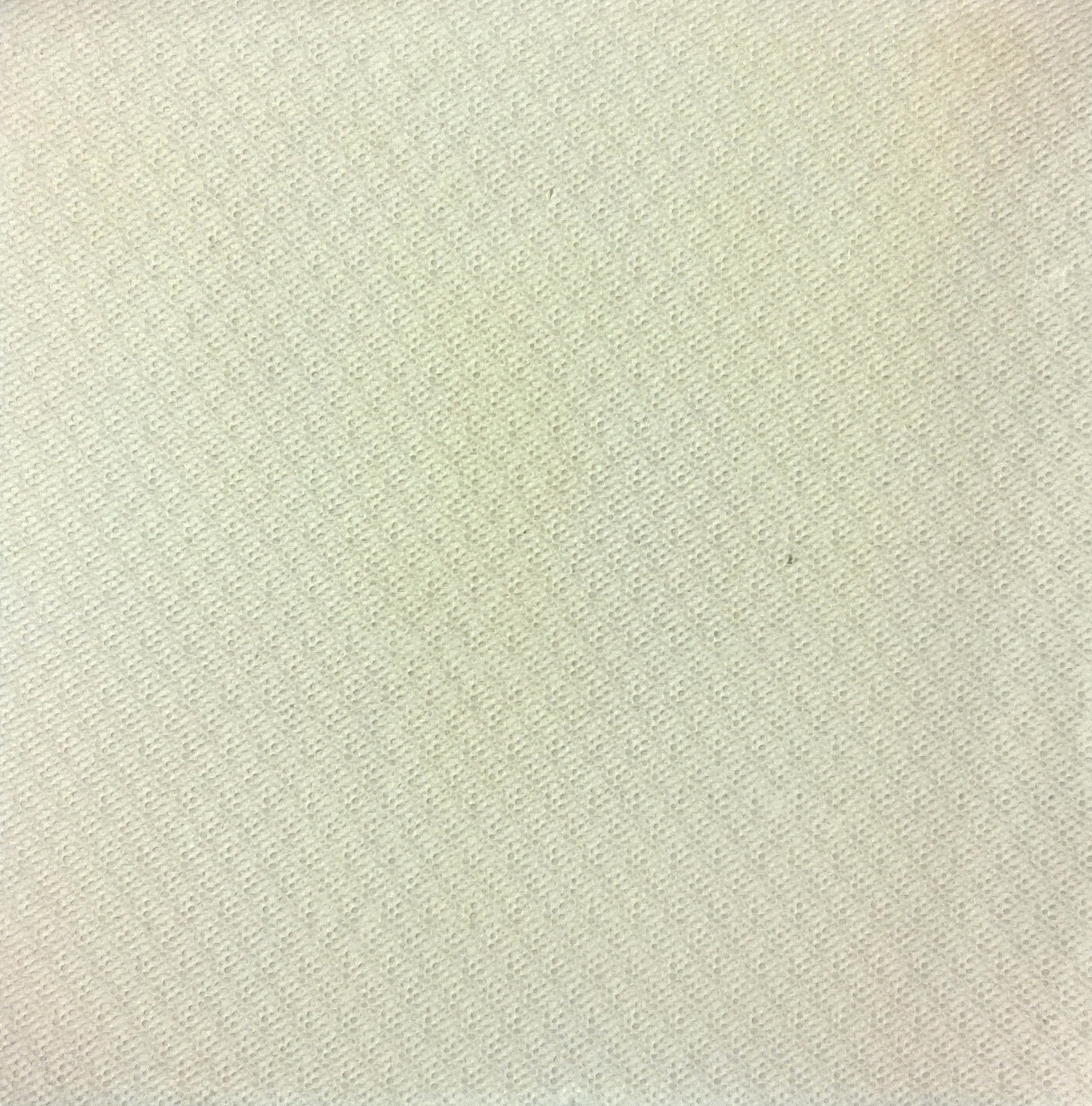

Supplement: Supplementary file 1 — Supplementary Information 2. [file 41598_2023_38929_MOESM1_ESM.zip › 23.jpg]

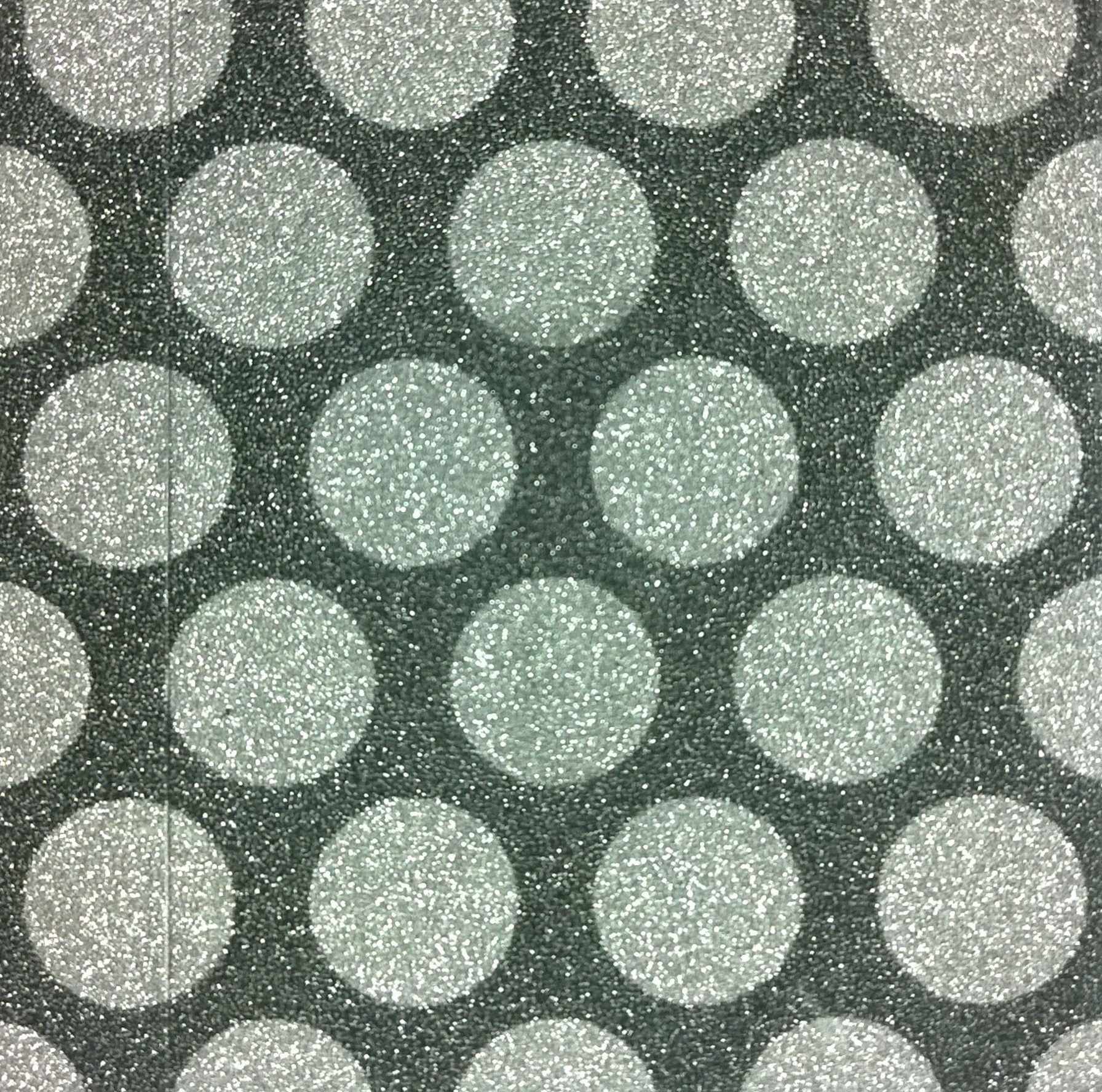

Supplement: Supplementary file 1 — Supplementary Information 2. [file 41598_2023_38929_MOESM1_ESM.zip › 24.jpg]

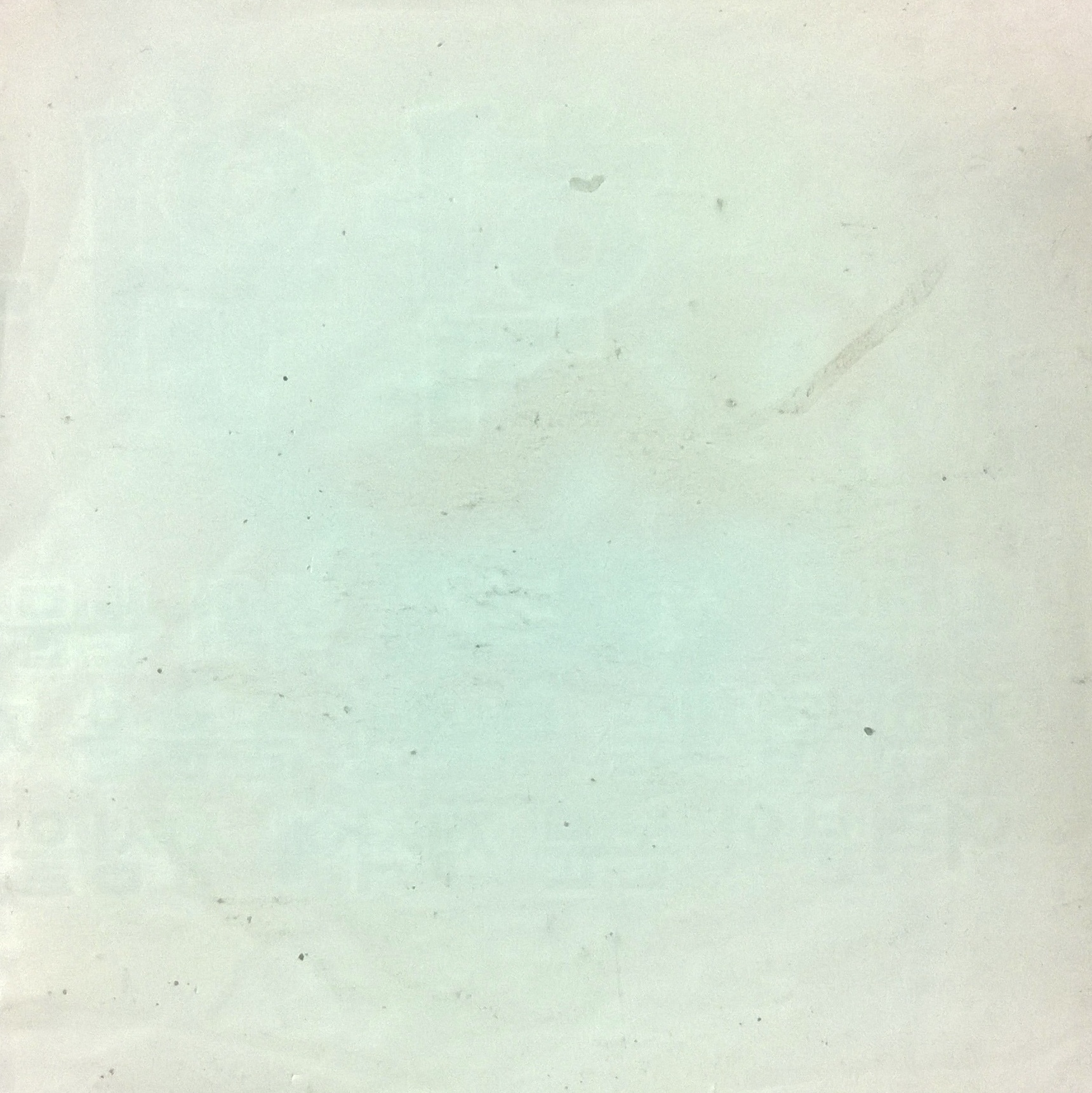

Supplement: Supplementary file 1 — Supplementary Information 2. [file 41598_2023_38929_MOESM1_ESM.zip › 25.jpg]

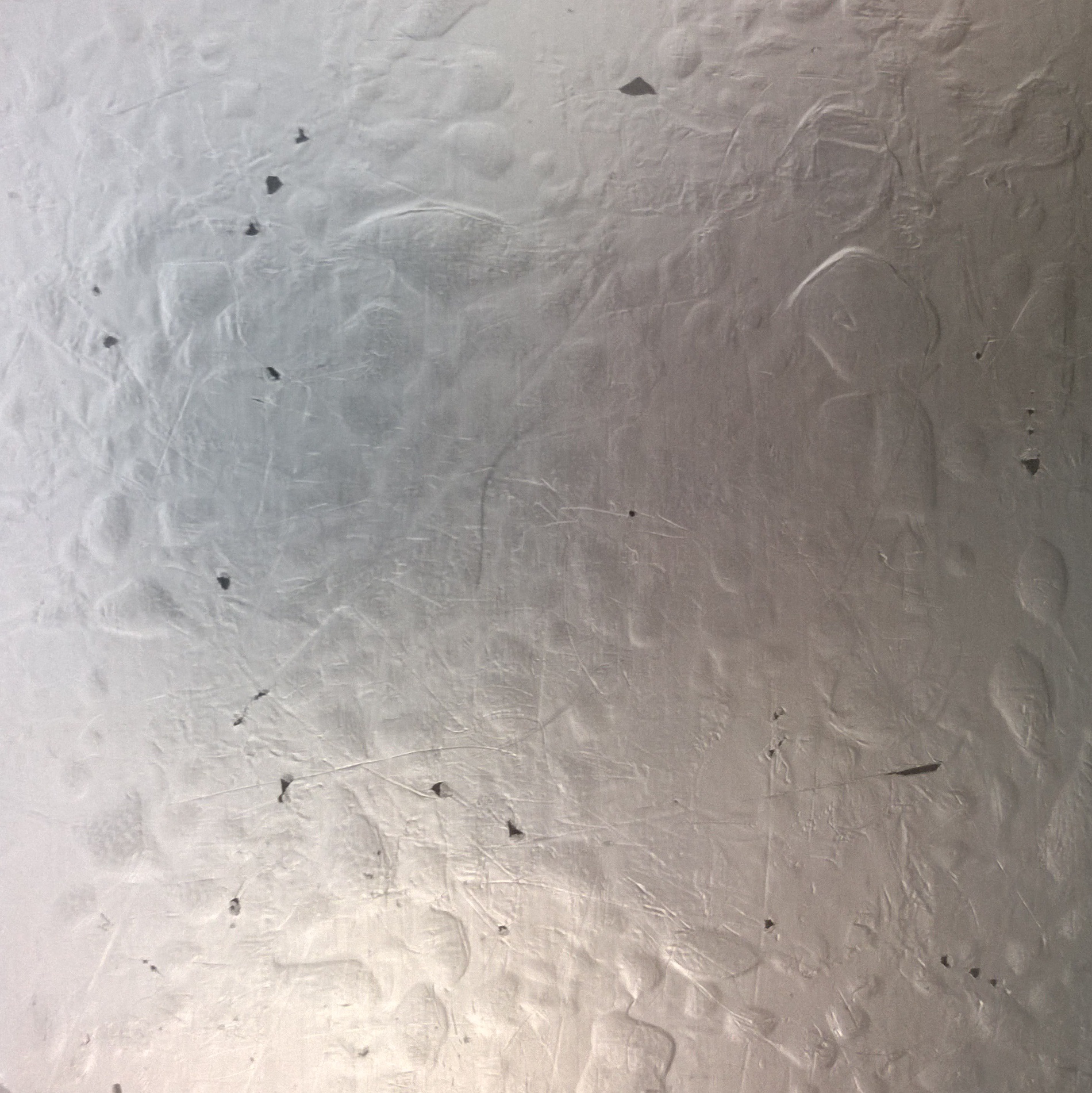

Supplement: Supplementary file 1 — Supplementary Information 2. [file 41598_2023_38929_MOESM1_ESM.zip › 26.jpg]

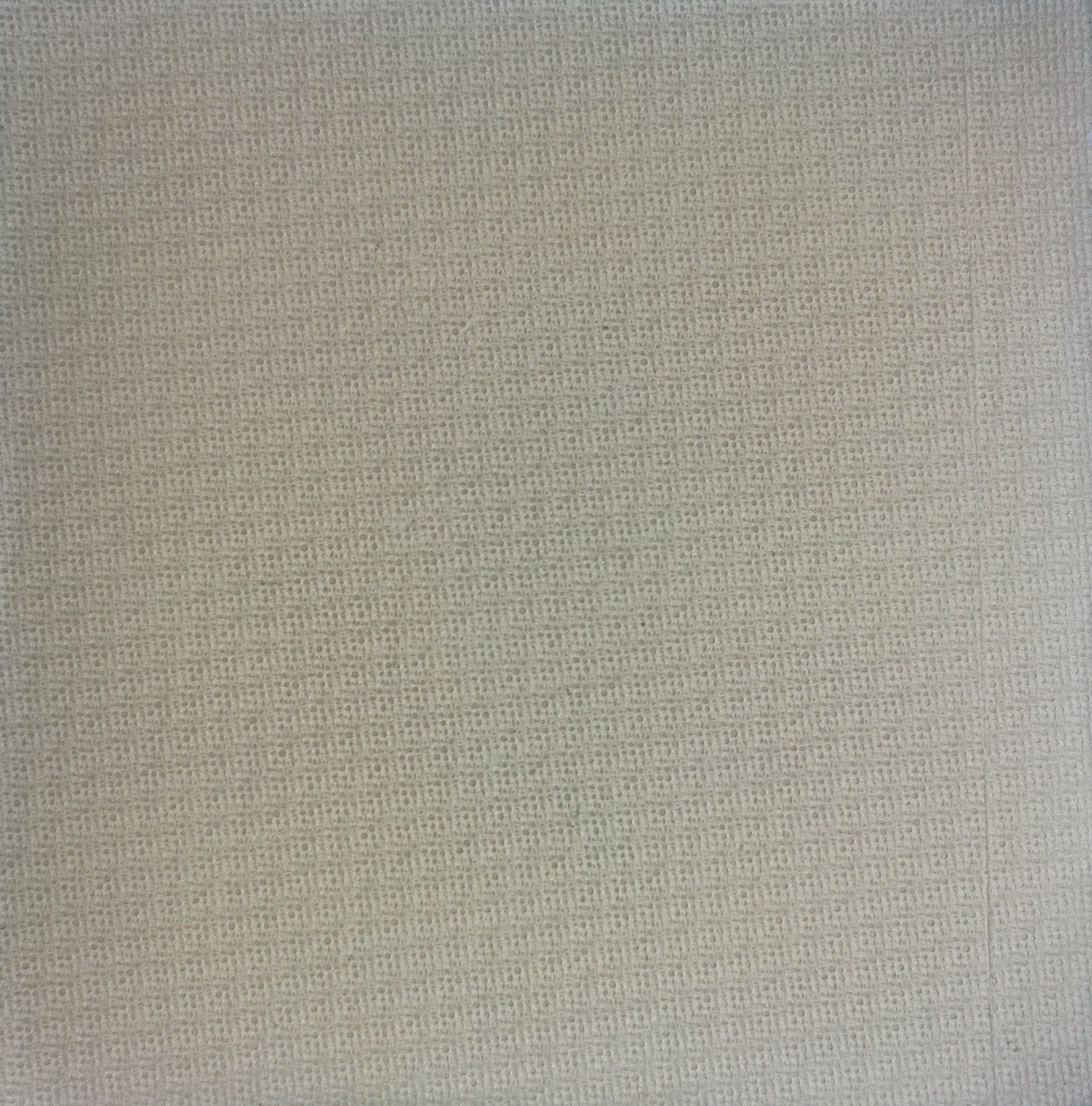

Supplement: Supplementary file 1 — Supplementary Information 2. [file 41598_2023_38929_MOESM1_ESM.zip › 27.jpg]

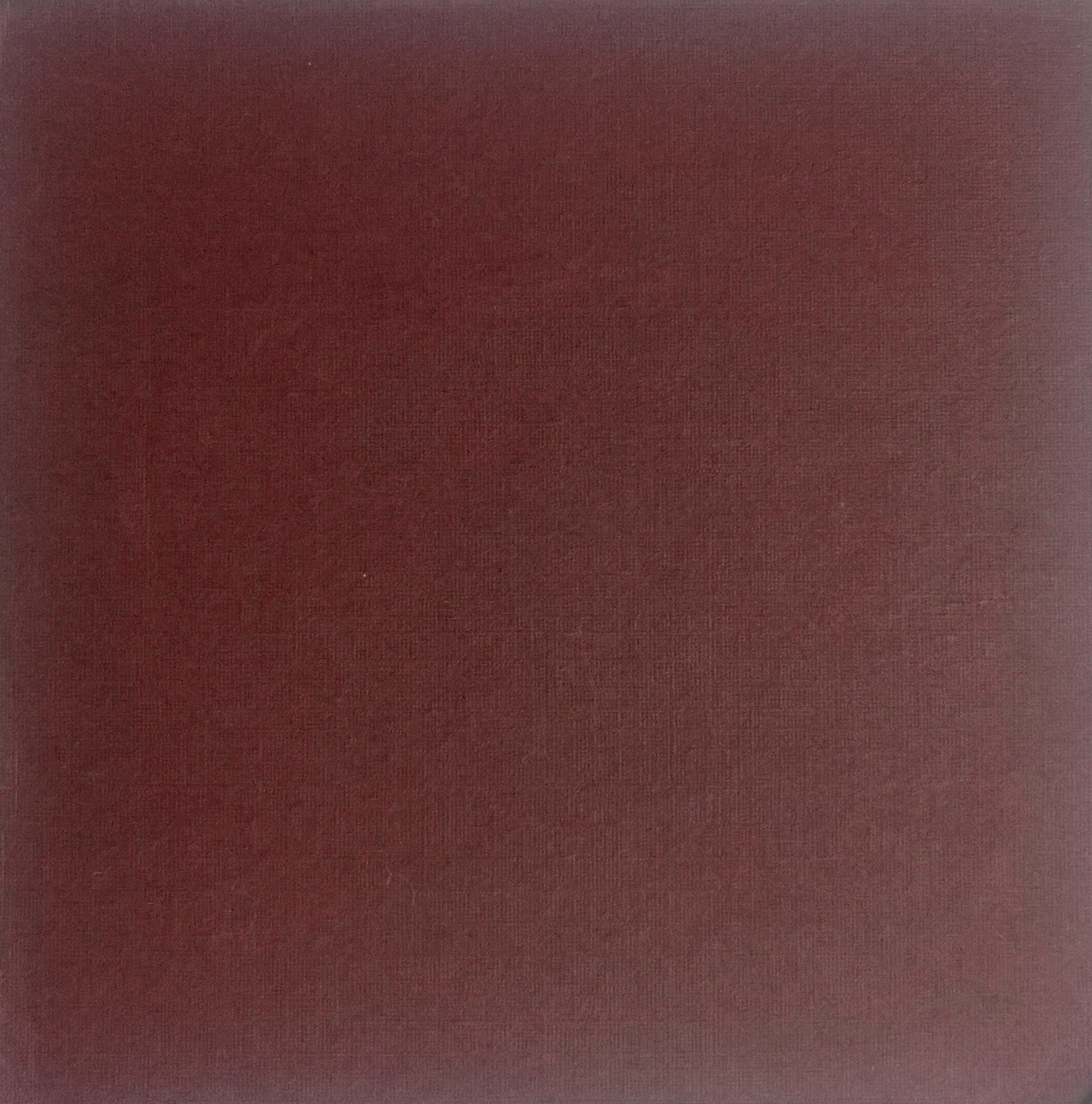

Supplement: Supplementary file 1 — Supplementary Information 2. [file 41598_2023_38929_MOESM1_ESM.zip › 28.jpg]

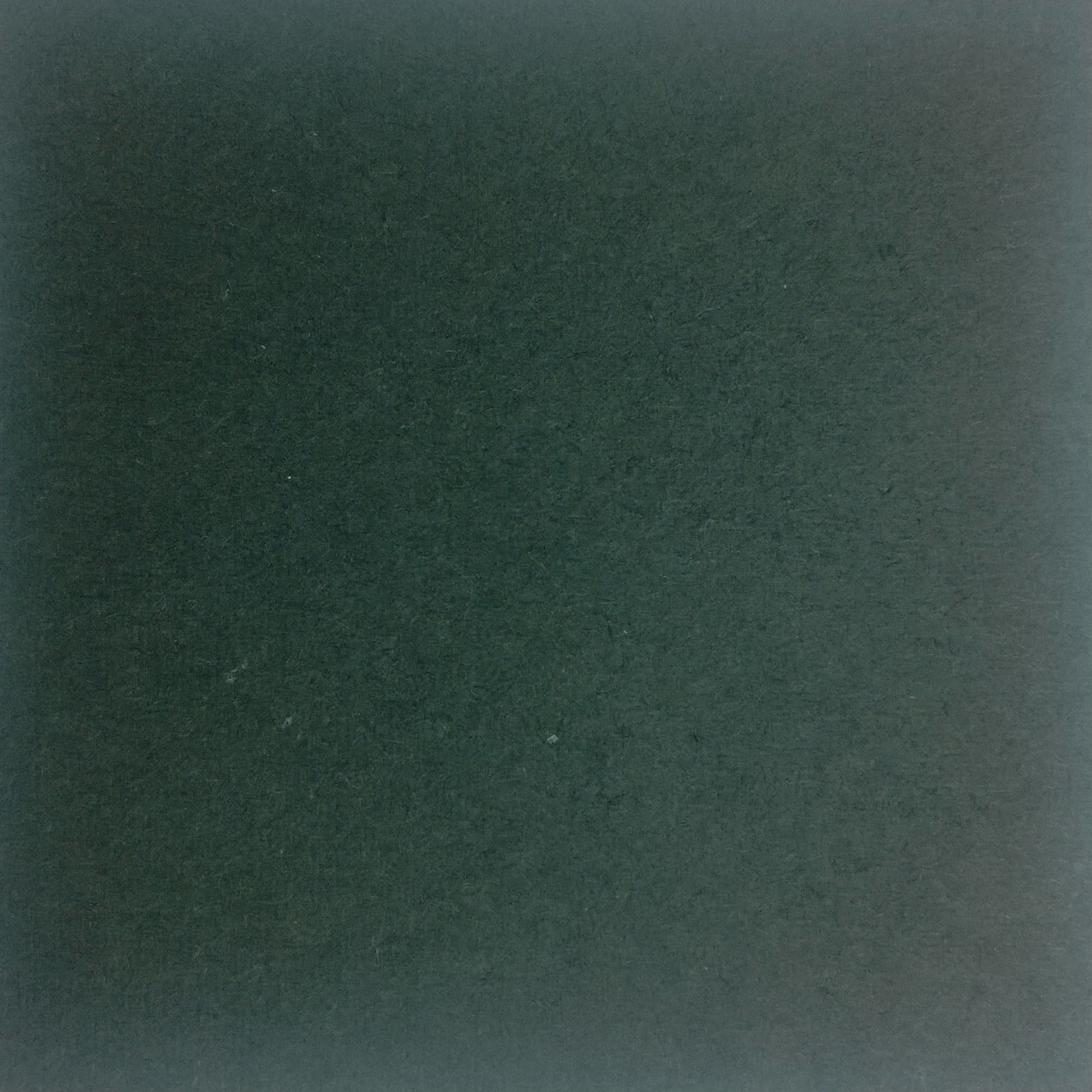

Supplement: Supplementary file 1 — Supplementary Information 2. [file 41598_2023_38929_MOESM1_ESM.zip › 29.jpg]

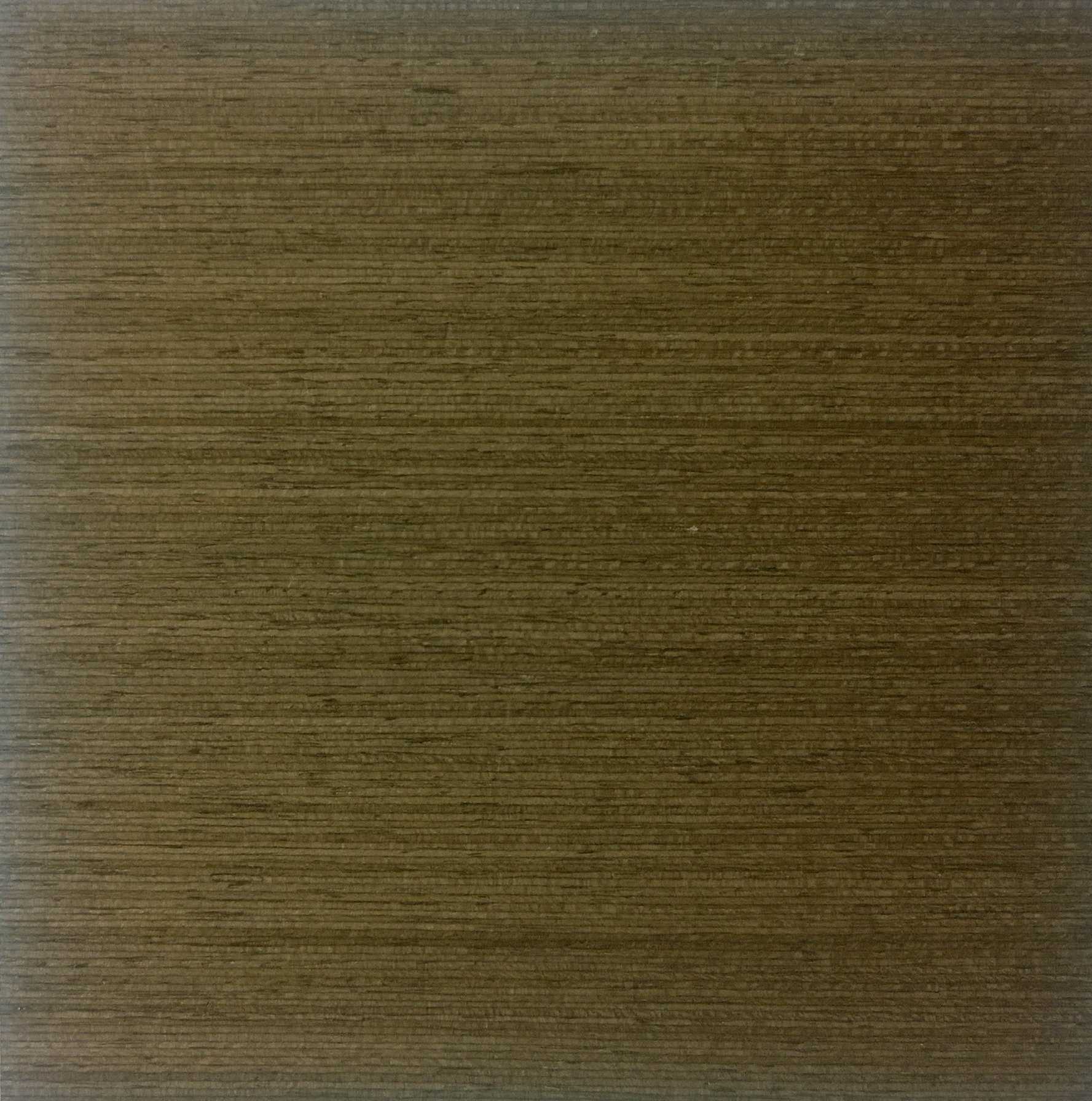

Supplement: Supplementary file 1 — Supplementary Information 2. [file 41598_2023_38929_MOESM1_ESM.zip › 3.jpg]

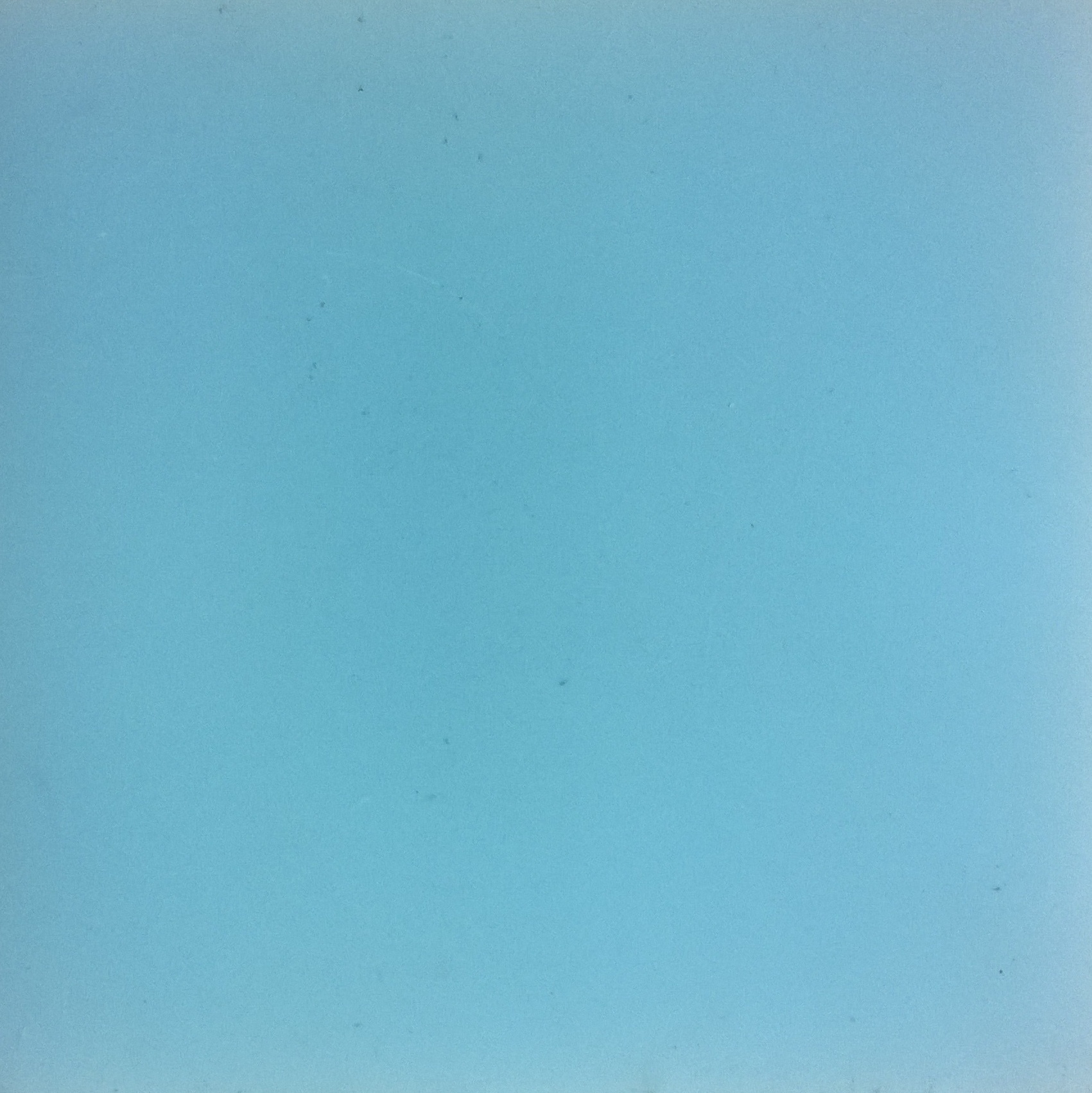

Supplement: Supplementary file 1 — Supplementary Information 2. [file 41598_2023_38929_MOESM1_ESM.zip › 30.jpg]

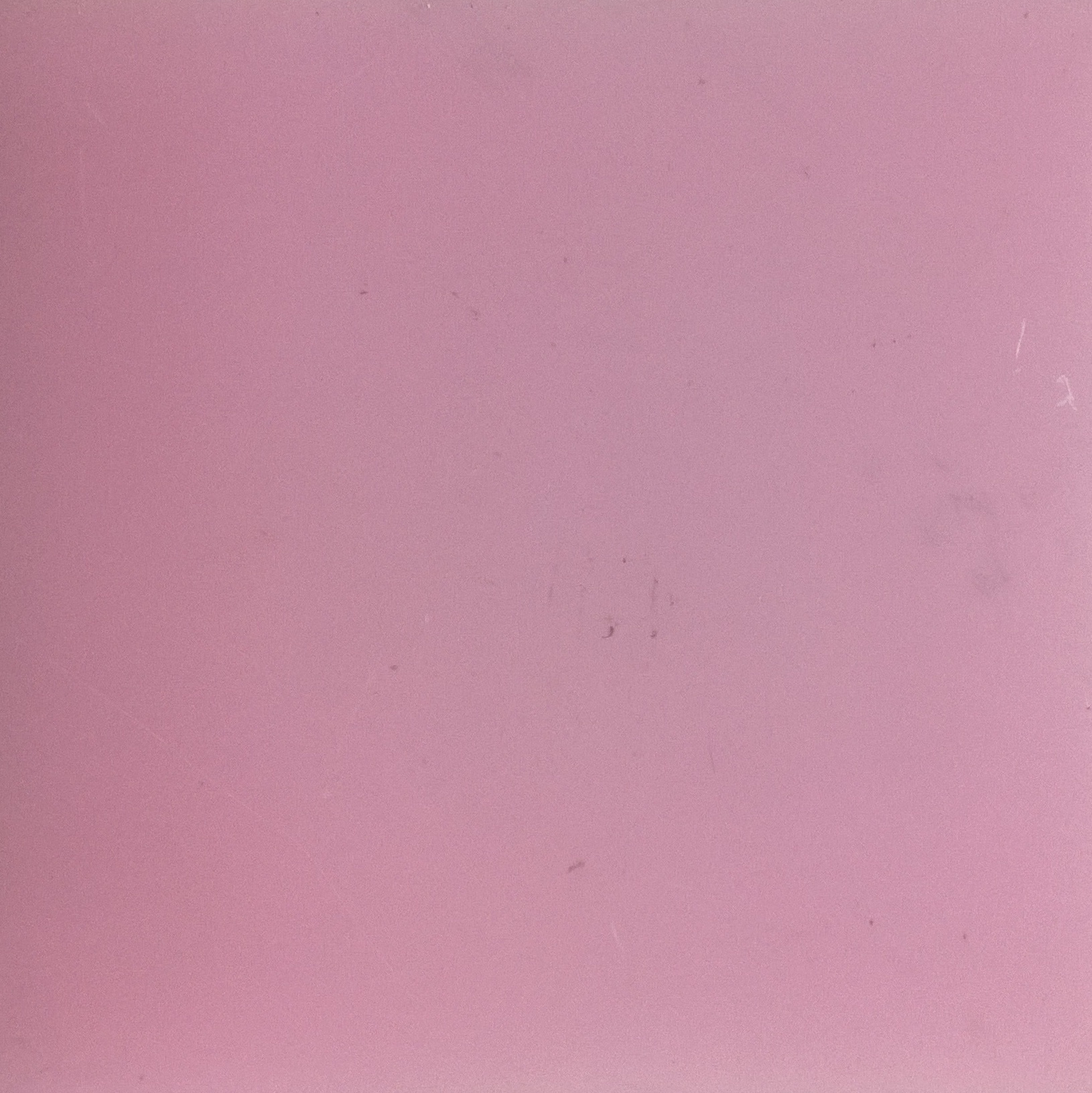

Supplement: Supplementary file 1 — Supplementary Information 2. [file 41598_2023_38929_MOESM1_ESM.zip › 31.jpg]

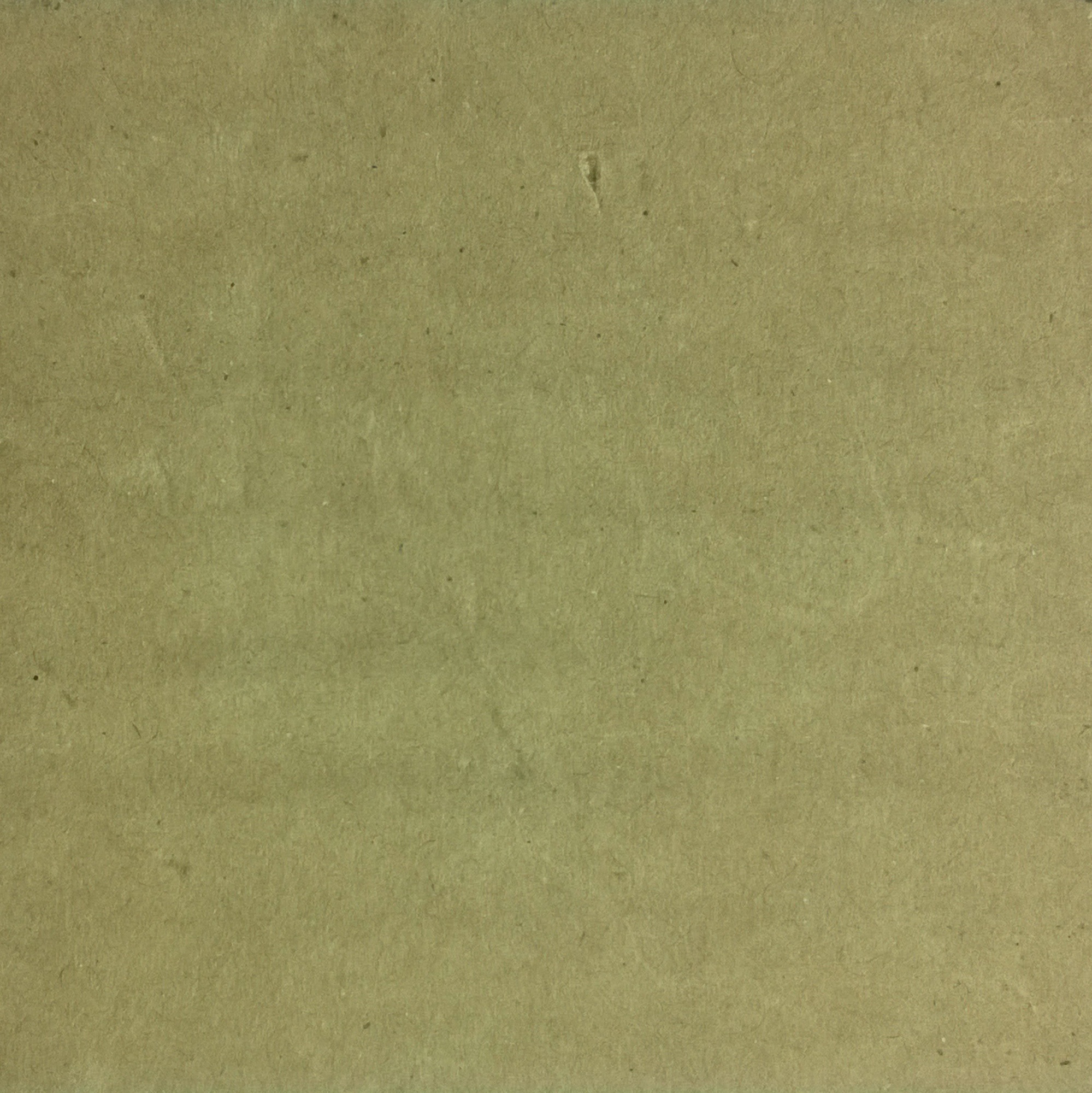

Supplement: Supplementary file 1 — Supplementary Information 2. [file 41598_2023_38929_MOESM1_ESM.zip › 32.jpg]

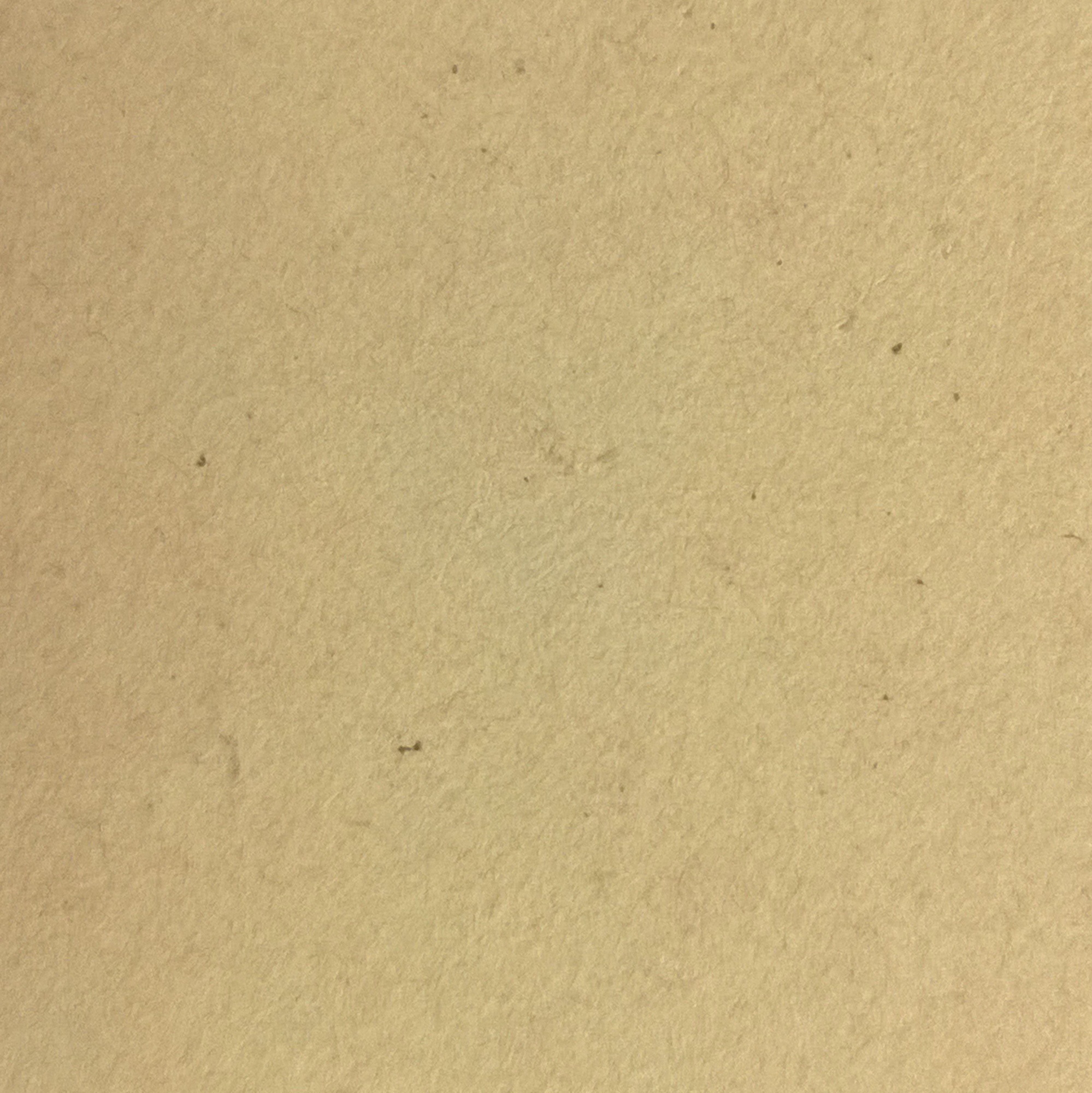

Supplement: Supplementary file 1 — Supplementary Information 2. [file 41598_2023_38929_MOESM1_ESM.zip › 33.jpg]

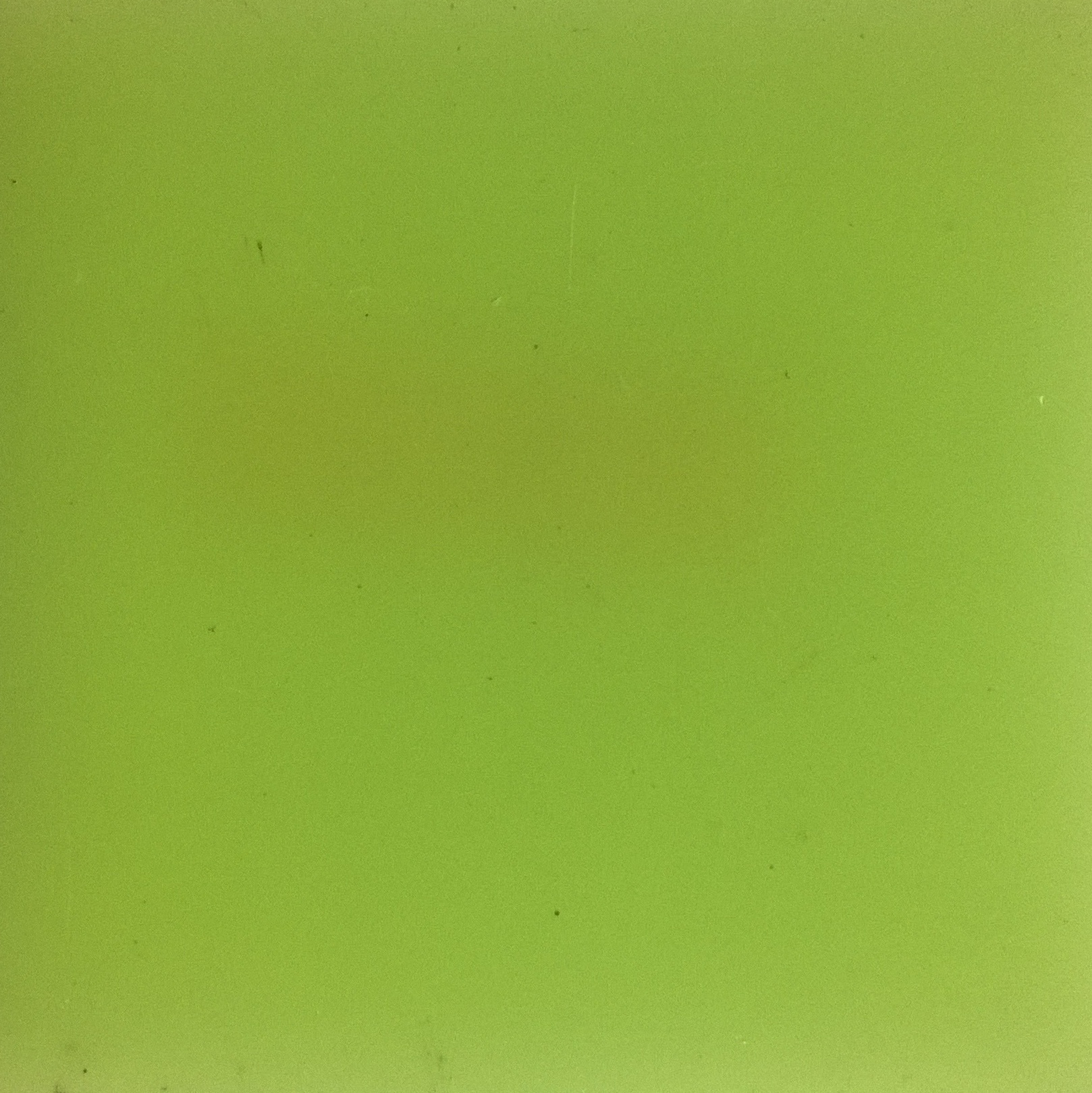

Supplement: Supplementary file 1 — Supplementary Information 2. [file 41598_2023_38929_MOESM1_ESM.zip › 34.jpg]

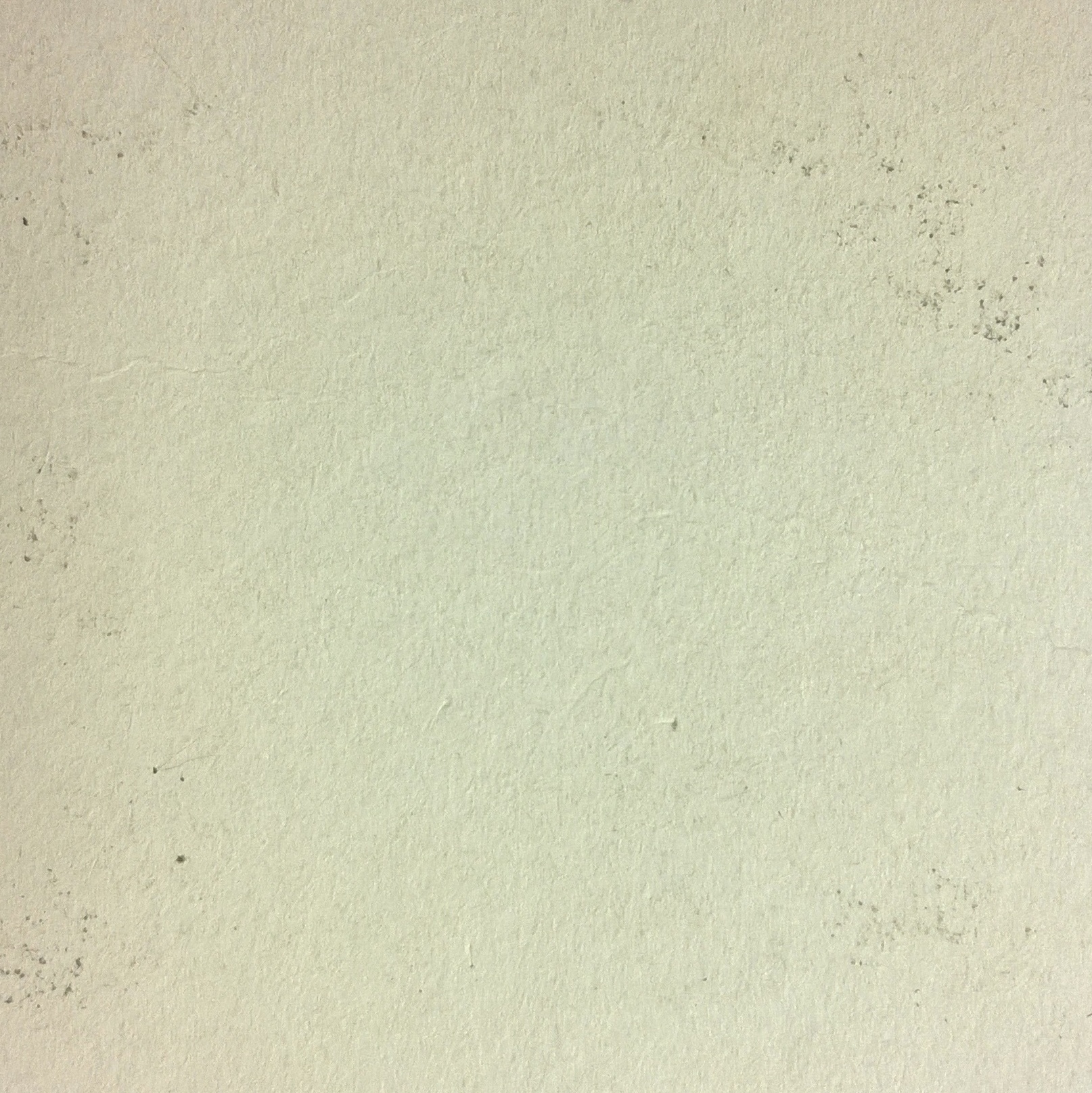

Supplement: Supplementary file 1 — Supplementary Information 2. [file 41598_2023_38929_MOESM1_ESM.zip › 35.jpg]

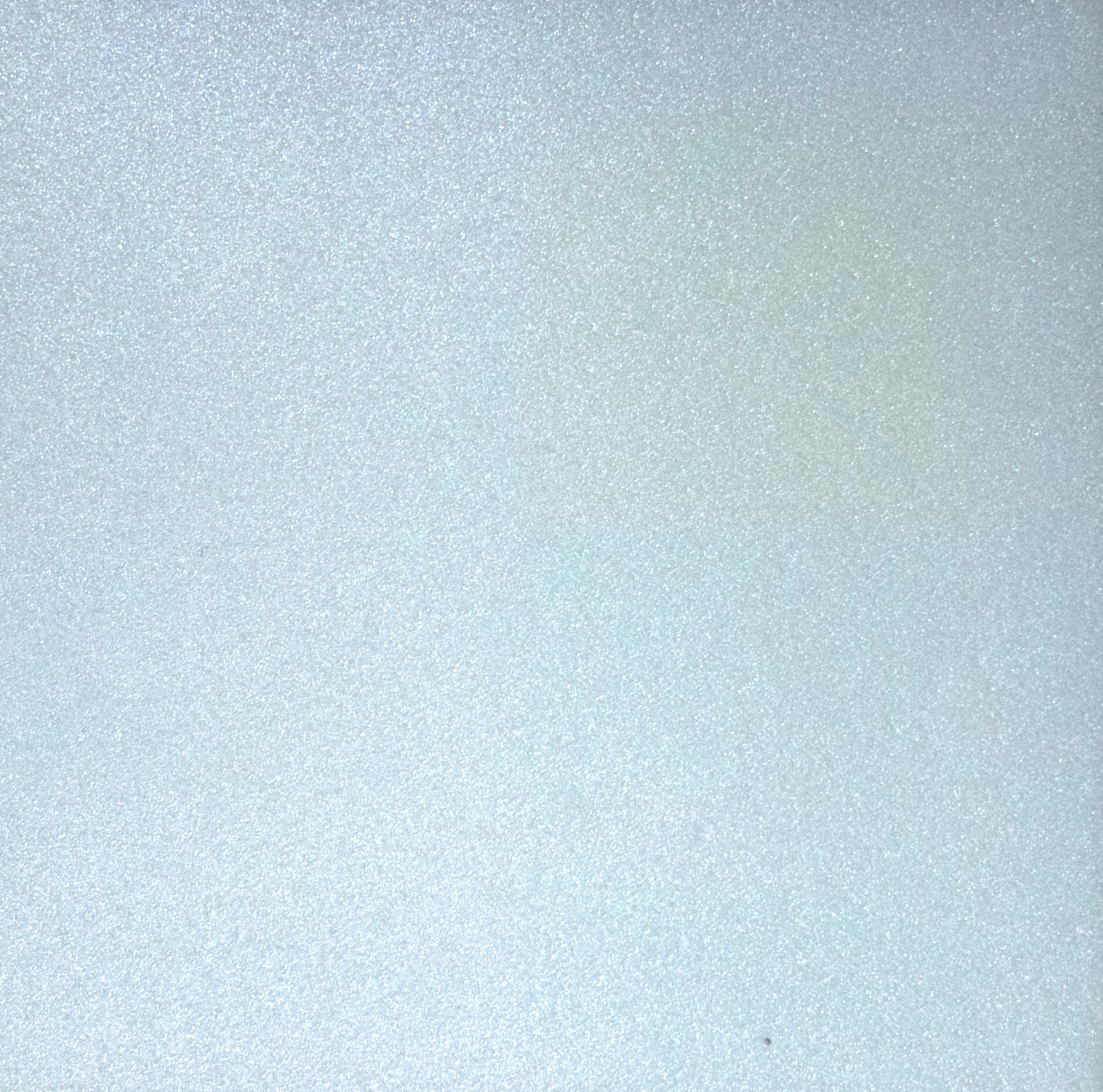

Supplement: Supplementary file 1 — Supplementary Information 2. [file 41598_2023_38929_MOESM1_ESM.zip › 36.JPG]

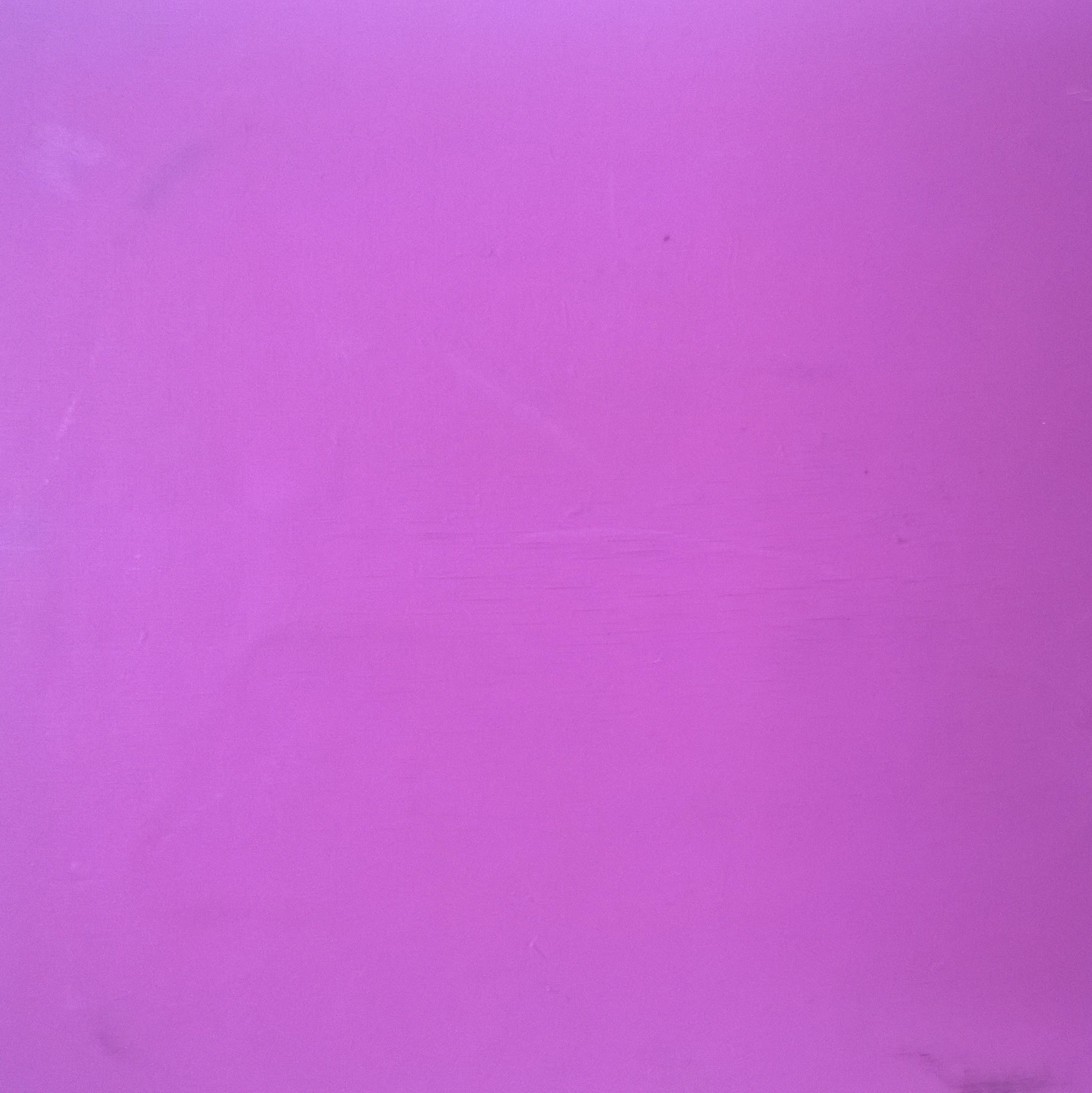

Supplement: Supplementary file 1 — Supplementary Information 2. [file 41598_2023_38929_MOESM1_ESM.zip › 37.jpg]

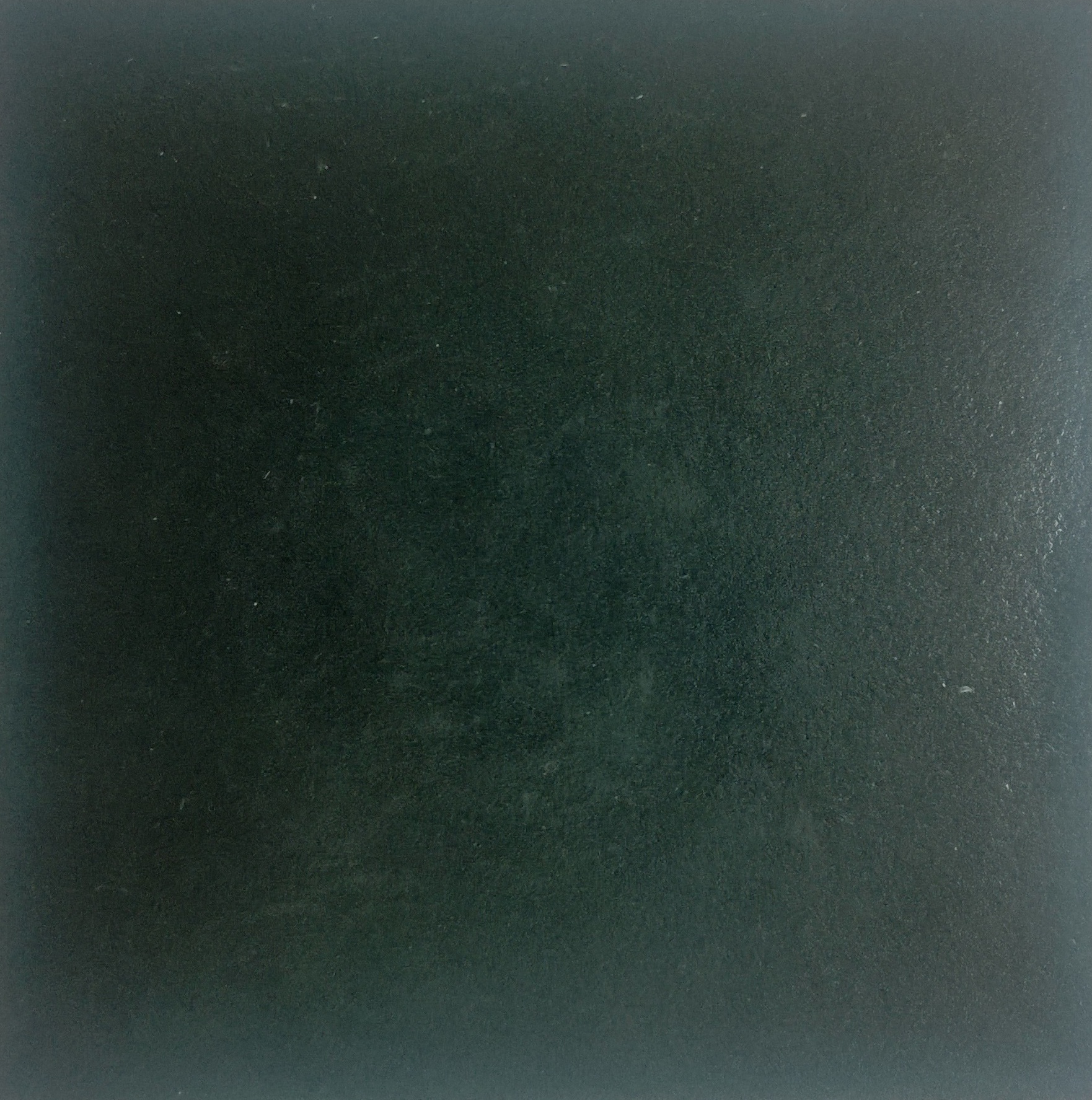

Supplement: Supplementary file 1 — Supplementary Information 2. [file 41598_2023_38929_MOESM1_ESM.zip › 38.jpg]

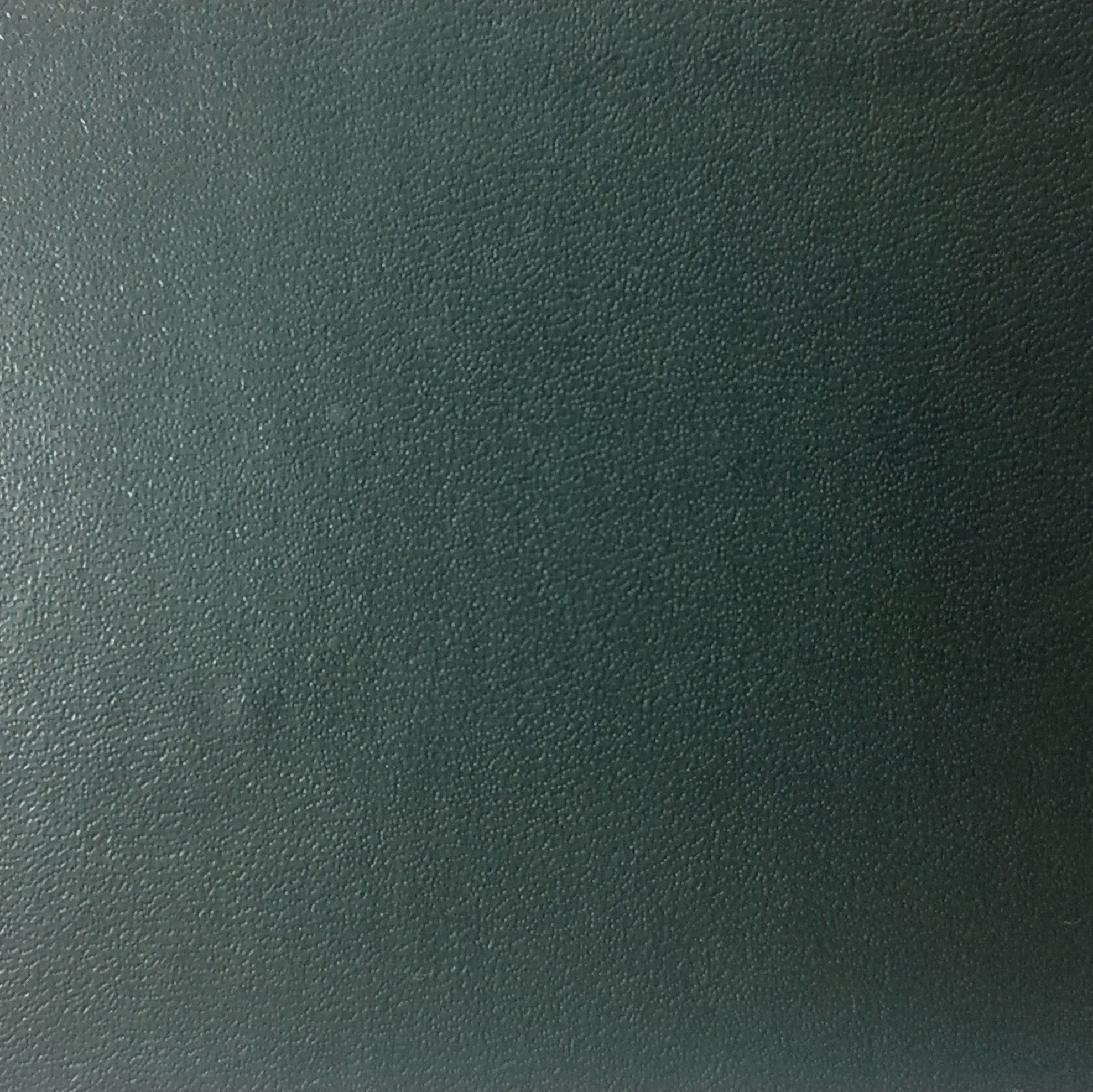

Supplement: Supplementary file 1 — Supplementary Information 2. [file 41598_2023_38929_MOESM1_ESM.zip › 39.jpg]

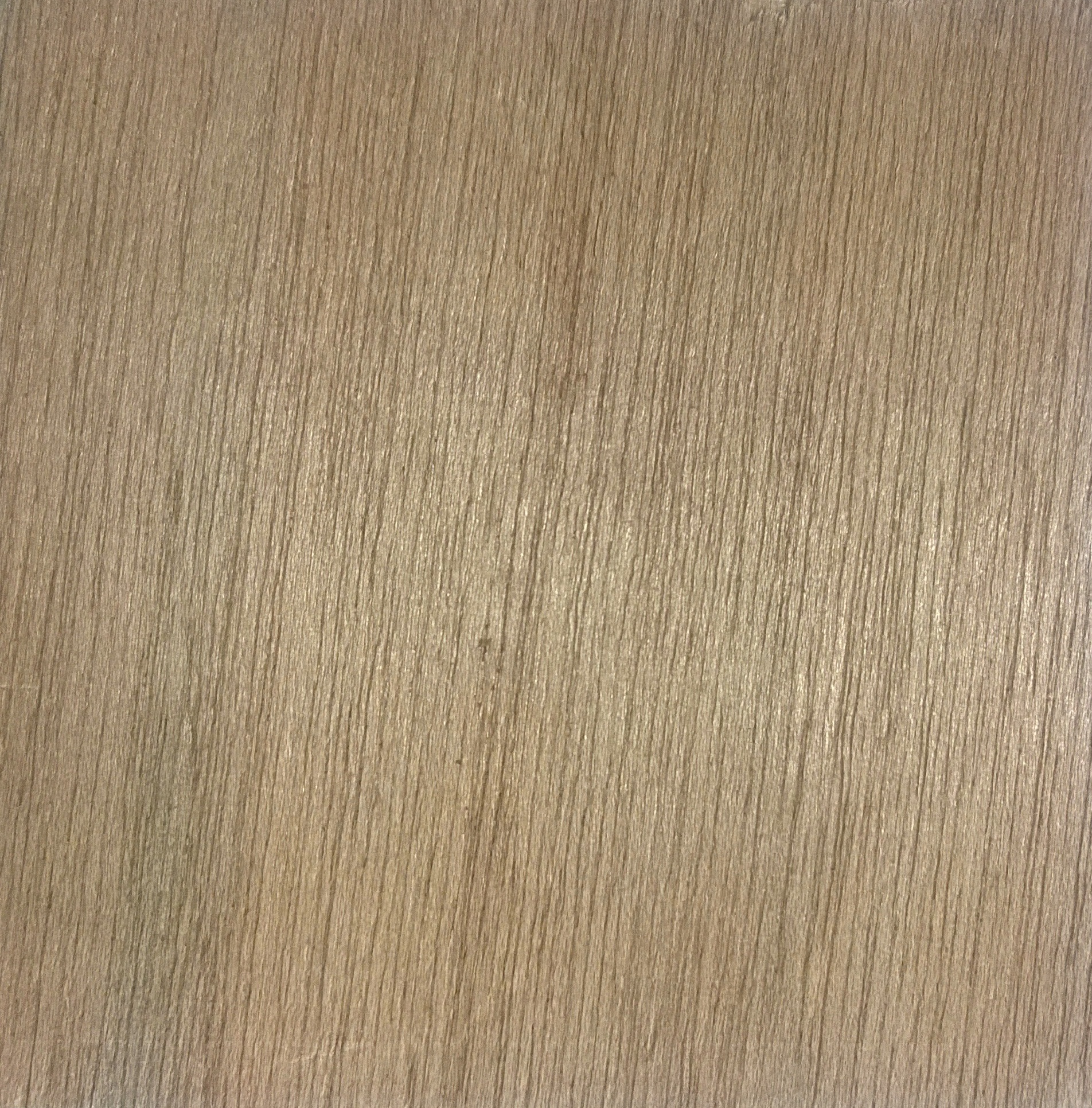

Supplement: Supplementary file 1 — Supplementary Information 2. [file 41598_2023_38929_MOESM1_ESM.zip › 4.jpg]

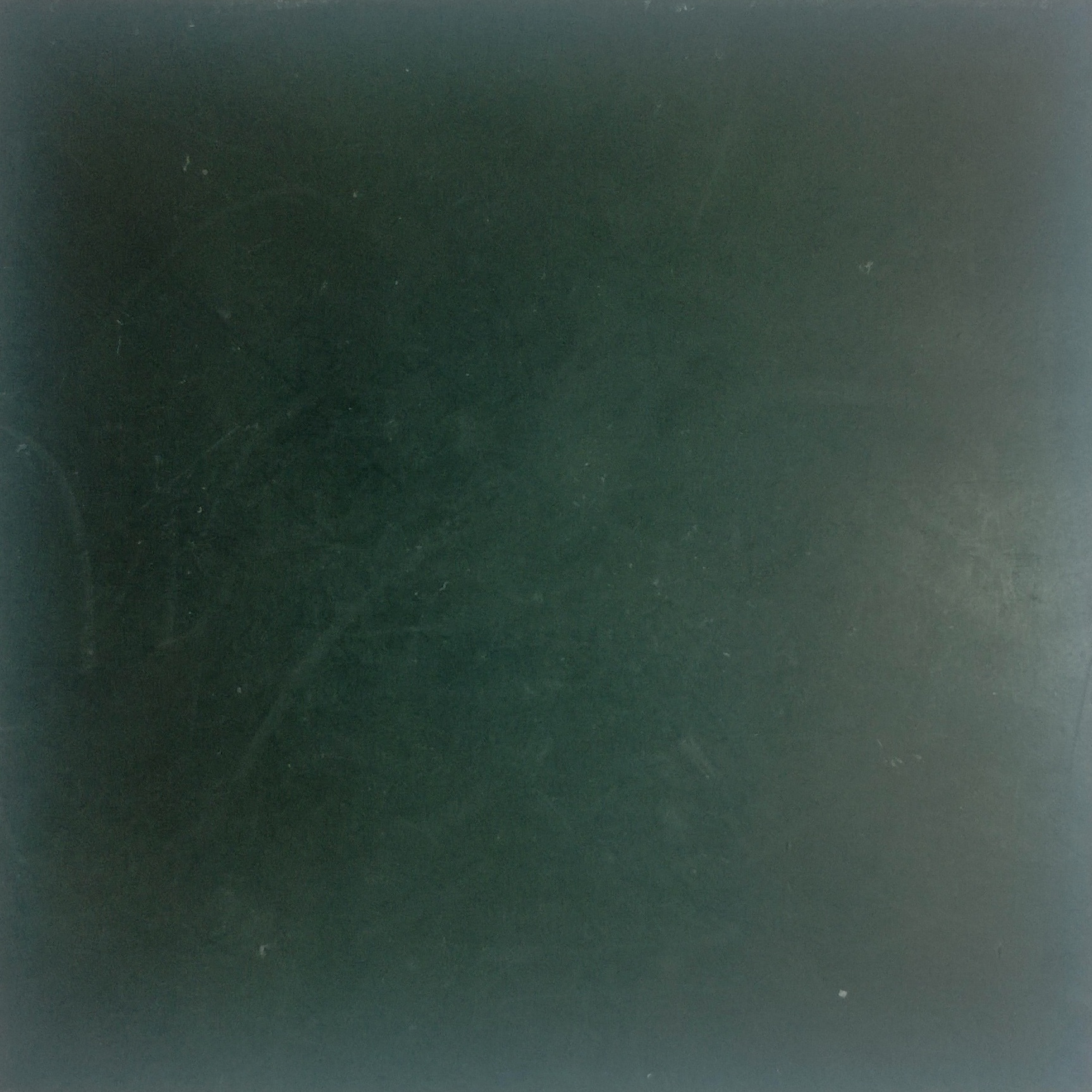

Supplement: Supplementary file 1 — Supplementary Information 2. [file 41598_2023_38929_MOESM1_ESM.zip › 40.jpg]

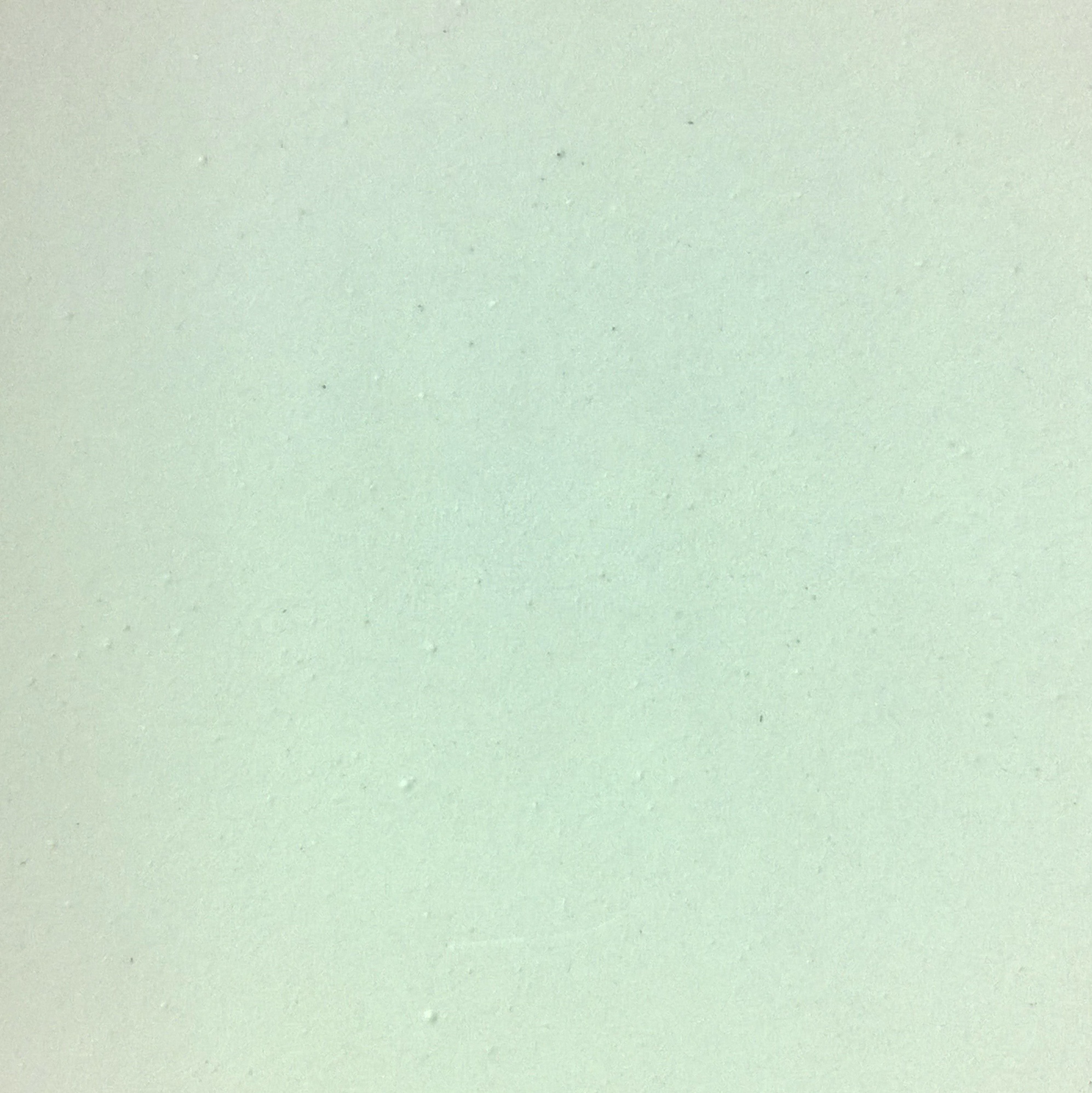

Supplement: Supplementary file 1 — Supplementary Information 2. [file 41598_2023_38929_MOESM1_ESM.zip › 41.jpg]

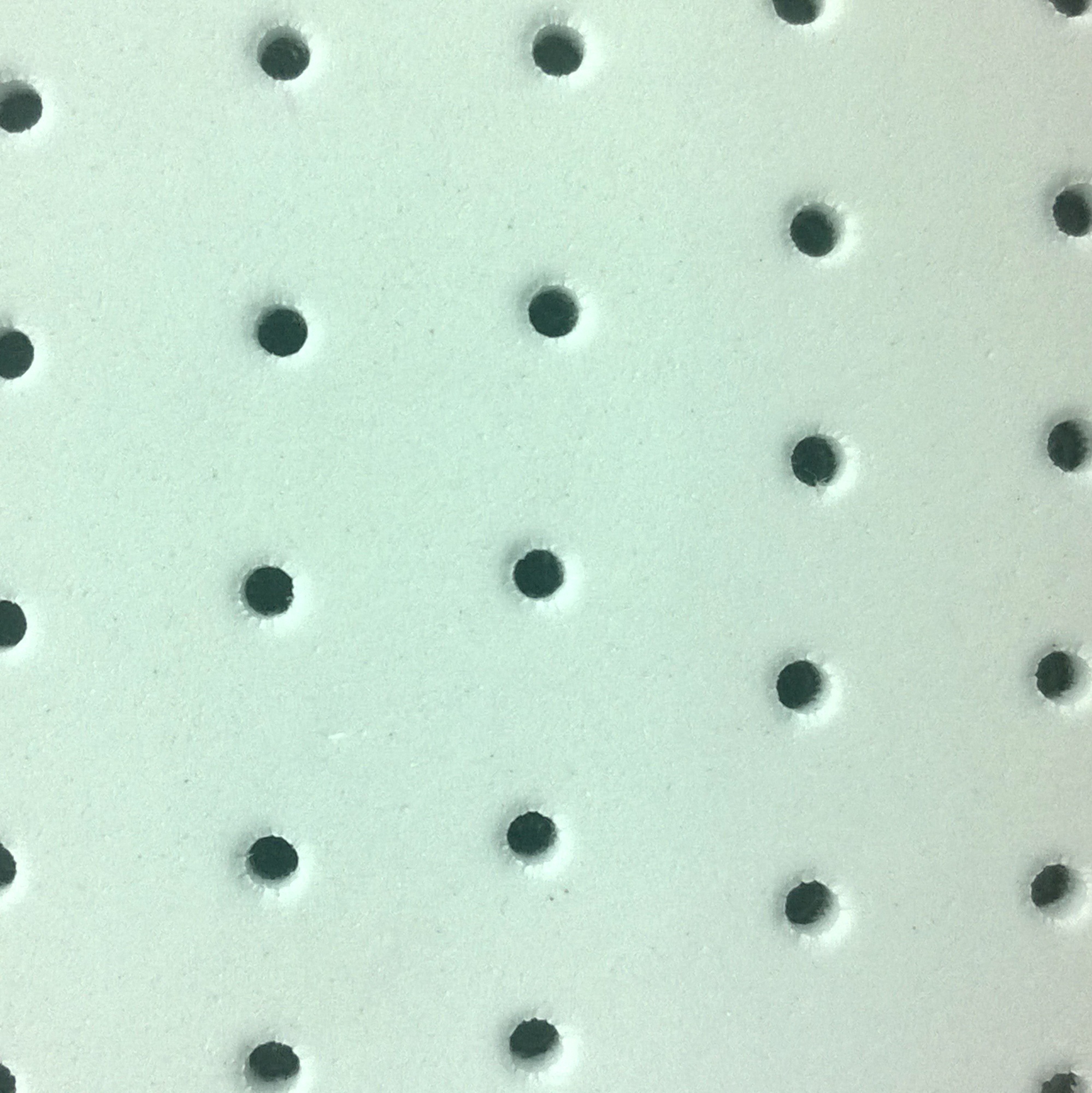

Supplement: Supplementary file 1 — Supplementary Information 2. [file 41598_2023_38929_MOESM1_ESM.zip › 42.jpg]

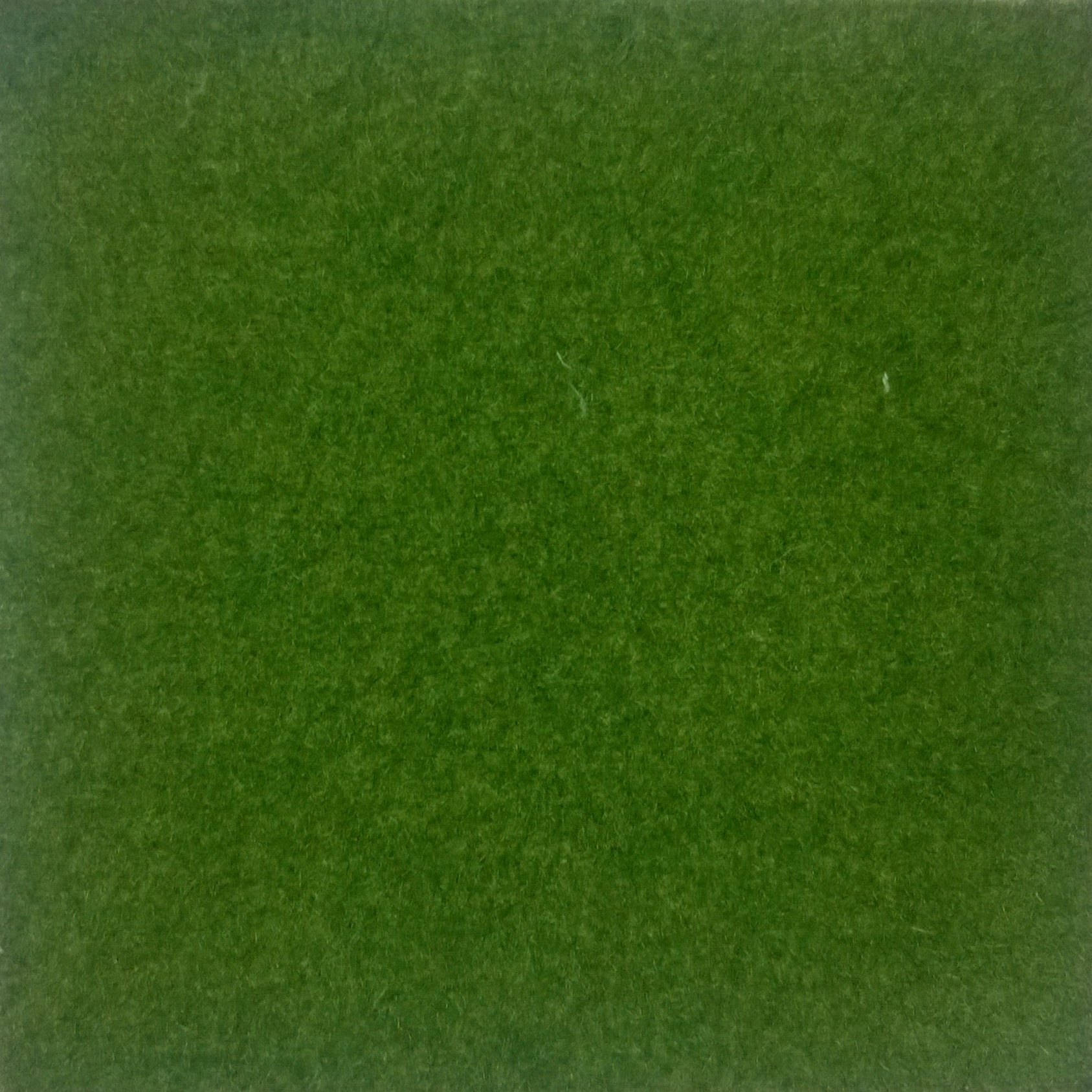

Supplement: Supplementary file 1 — Supplementary Information 2. [file 41598_2023_38929_MOESM1_ESM.zip › 43.jpg]

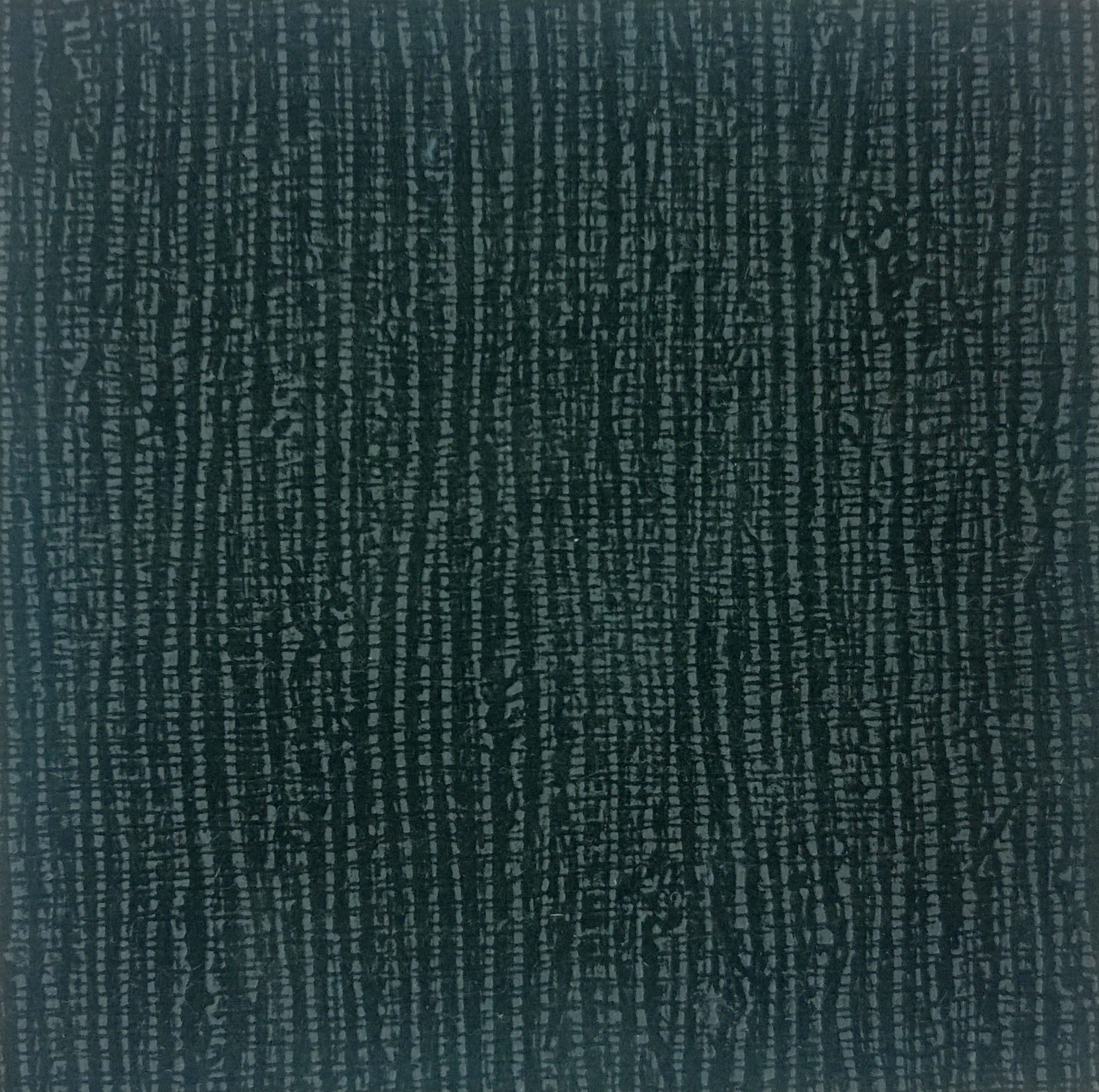

Supplement: Supplementary file 1 — Supplementary Information 2. [file 41598_2023_38929_MOESM1_ESM.zip › 44.jpg]

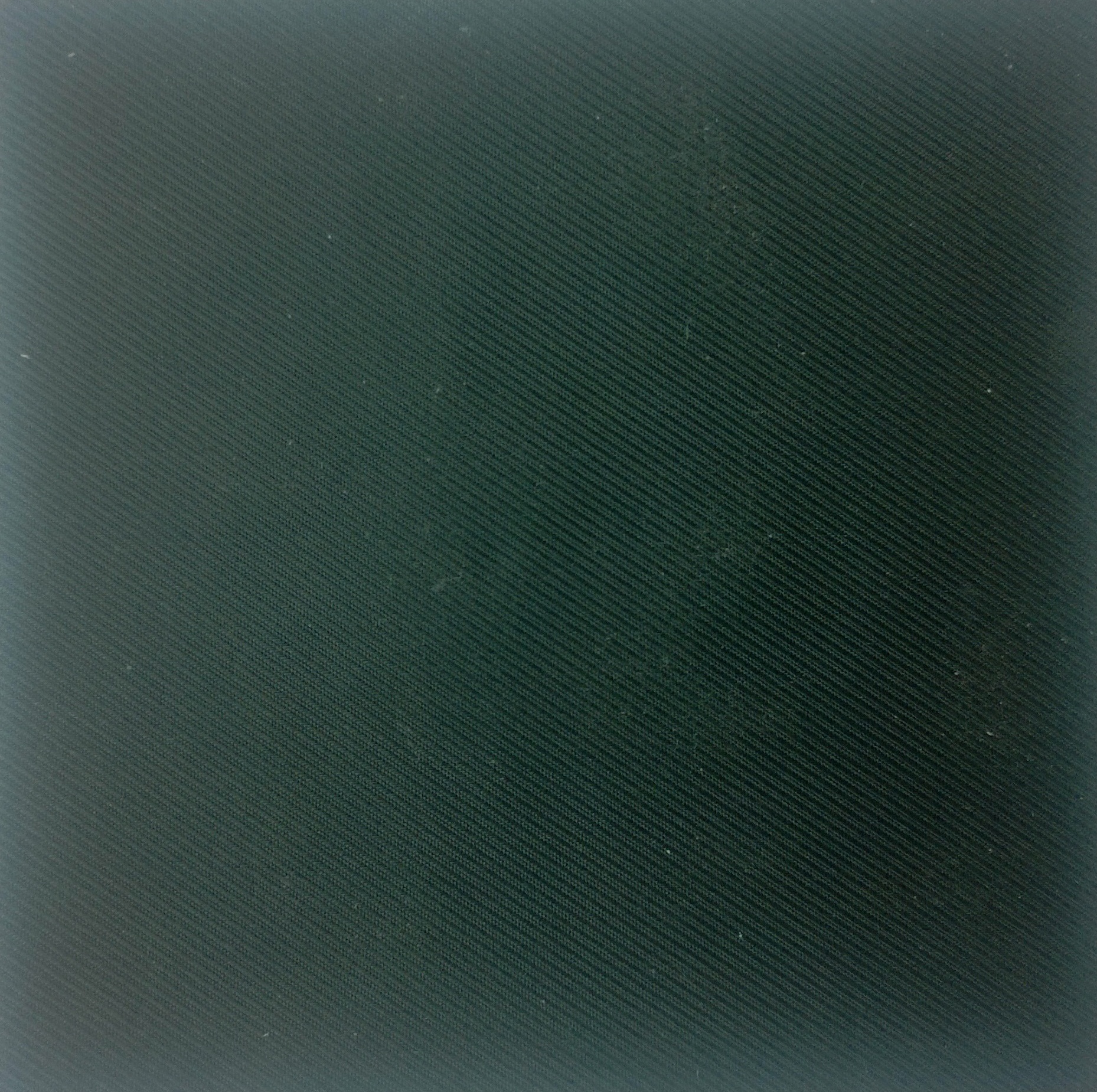

Supplement: Supplementary file 1 — Supplementary Information 2. [file 41598_2023_38929_MOESM1_ESM.zip › 45.jpg]

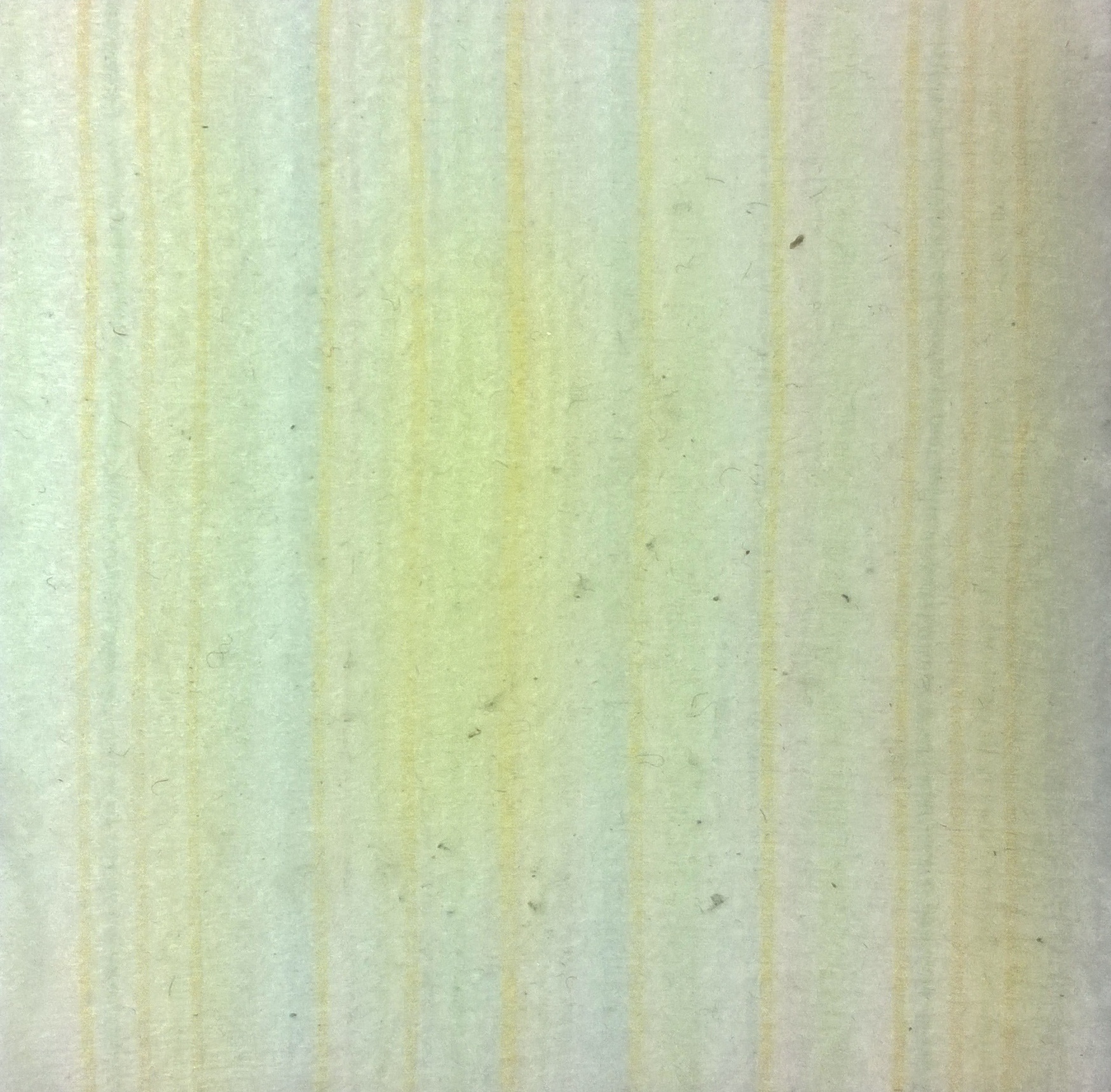

Supplement: Supplementary file 1 — Supplementary Information 2. [file 41598_2023_38929_MOESM1_ESM.zip › 46.jpg]

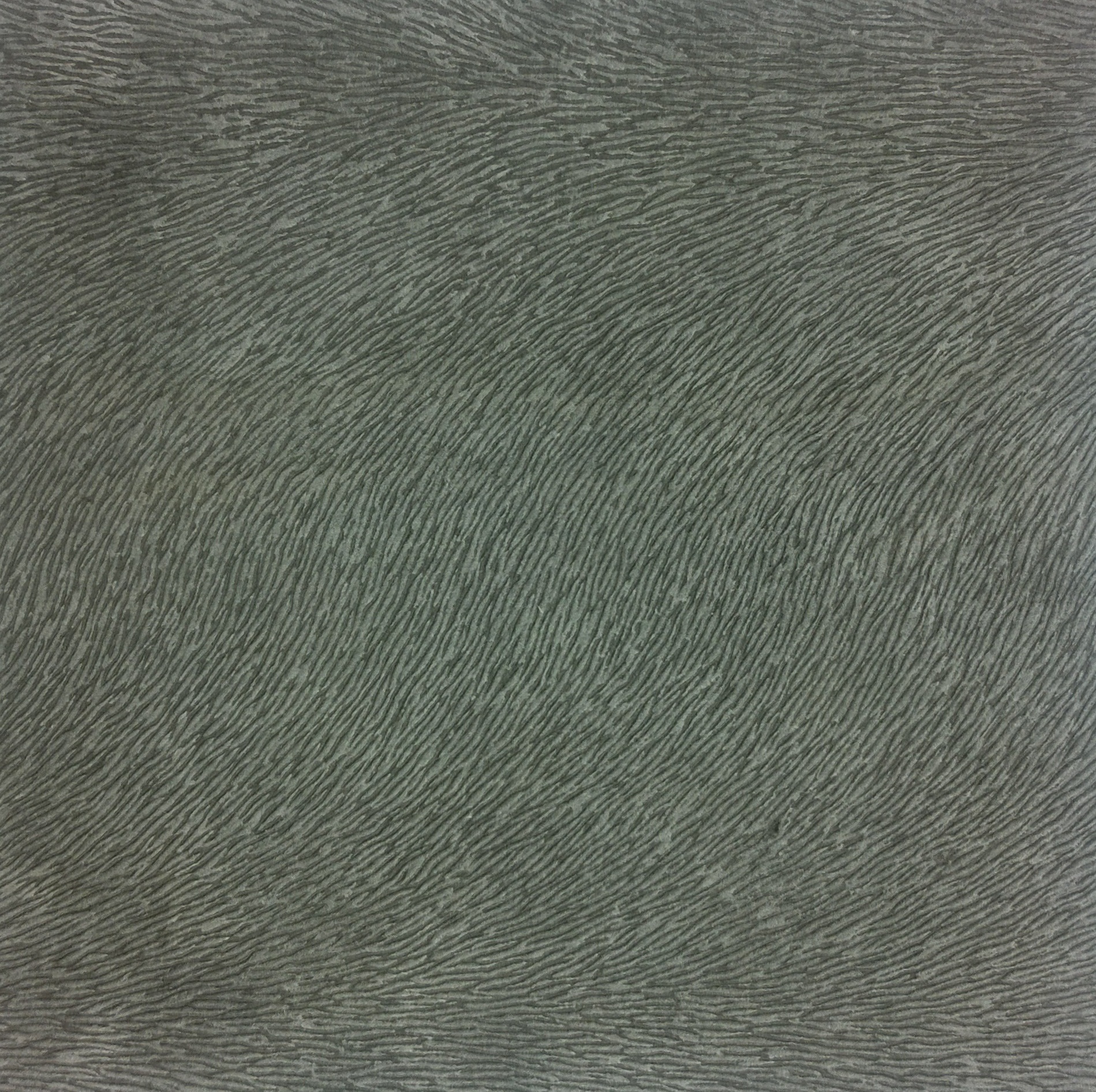

Supplement: Supplementary file 1 — Supplementary Information 2. [file 41598_2023_38929_MOESM1_ESM.zip › 47.jpg]

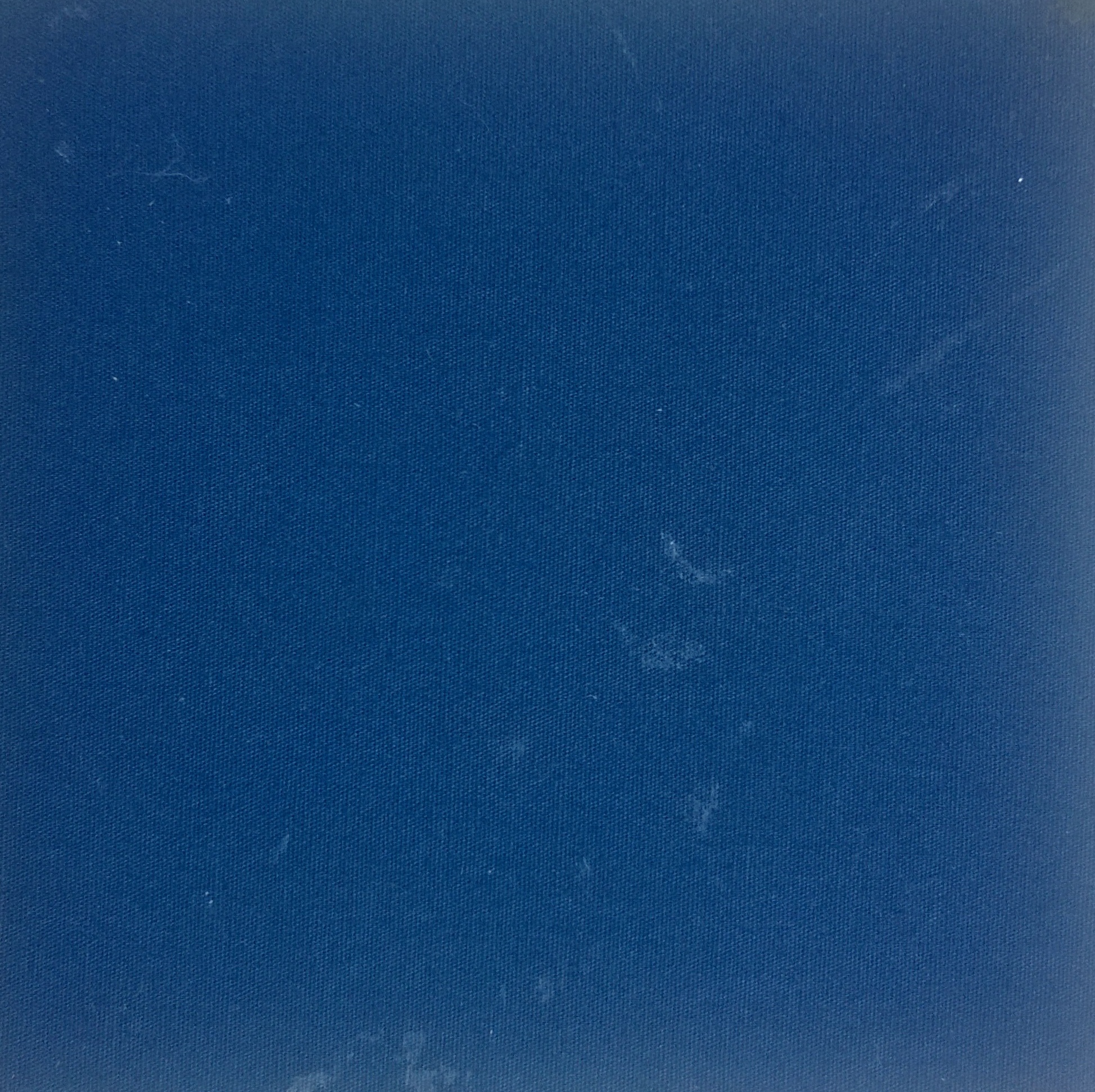

Supplement: Supplementary file 1 — Supplementary Information 2. [file 41598_2023_38929_MOESM1_ESM.zip › 48.jpg]

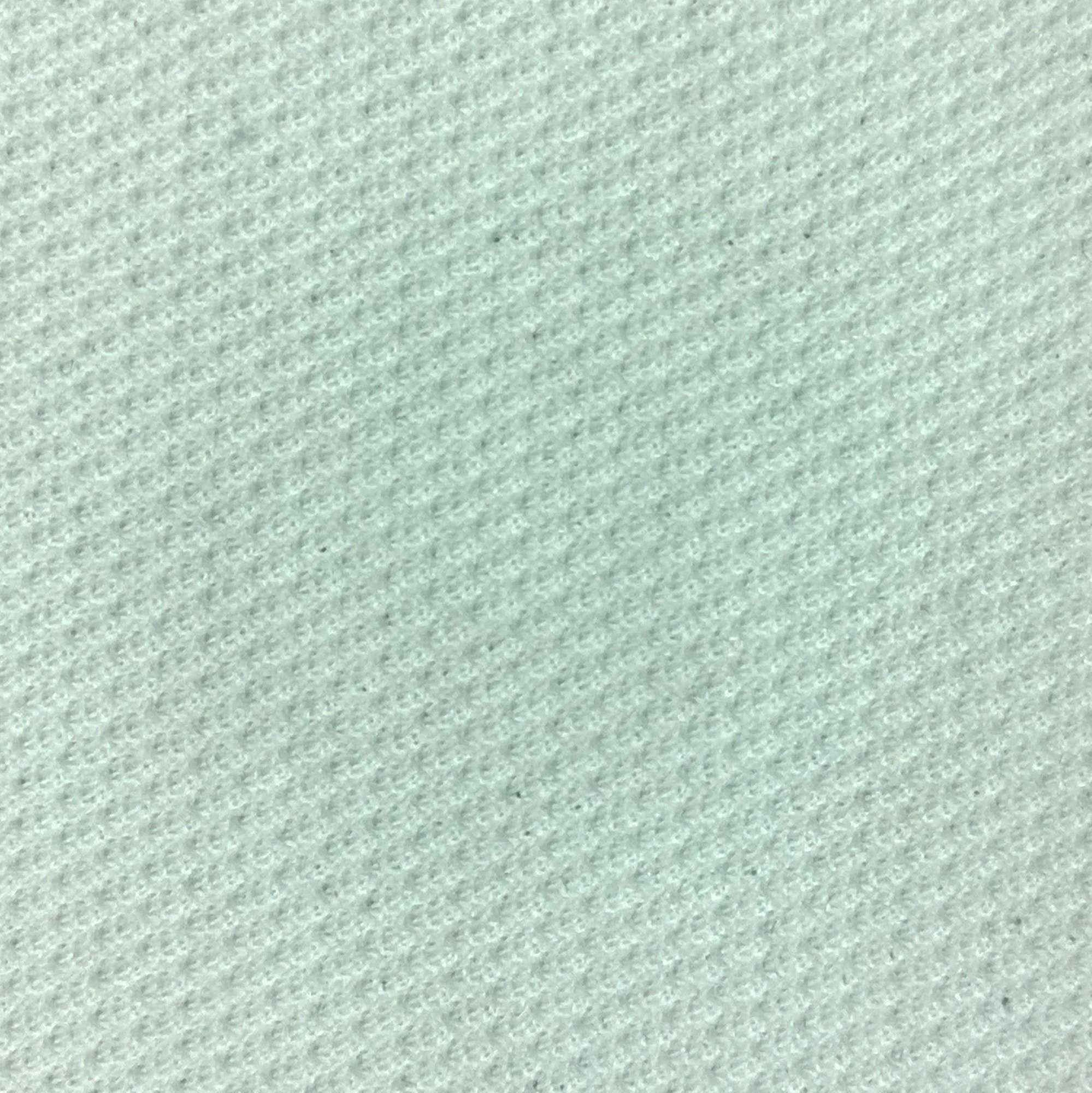

Supplement: Supplementary file 1 — Supplementary Information 2. [file 41598_2023_38929_MOESM1_ESM.zip › 49.jpg]

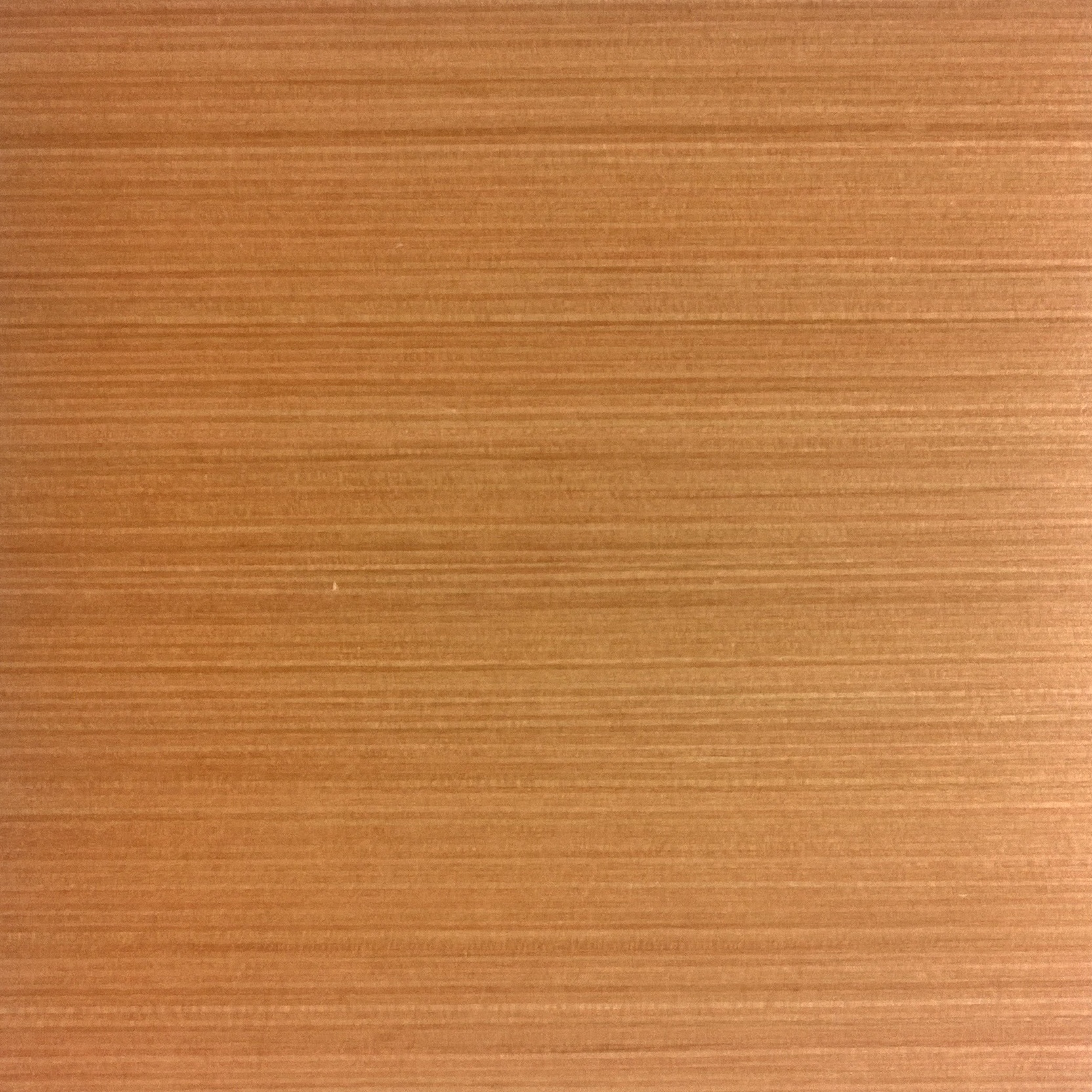

Supplement: Supplementary file 1 — Supplementary Information 2. [file 41598_2023_38929_MOESM1_ESM.zip › 5.jpg]

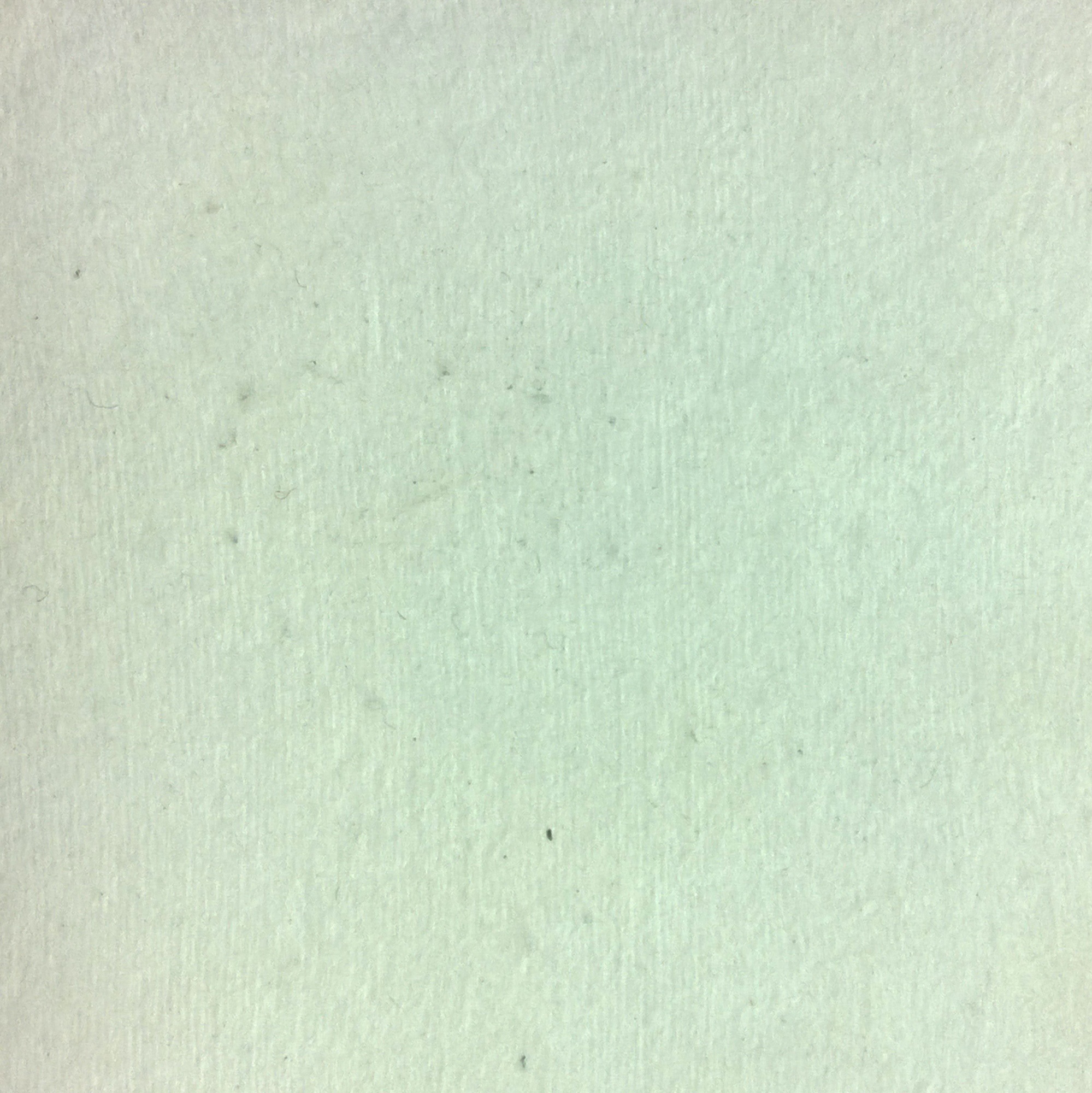

Supplement: Supplementary file 1 — Supplementary Information 2. [file 41598_2023_38929_MOESM1_ESM.zip › 50.jpg]

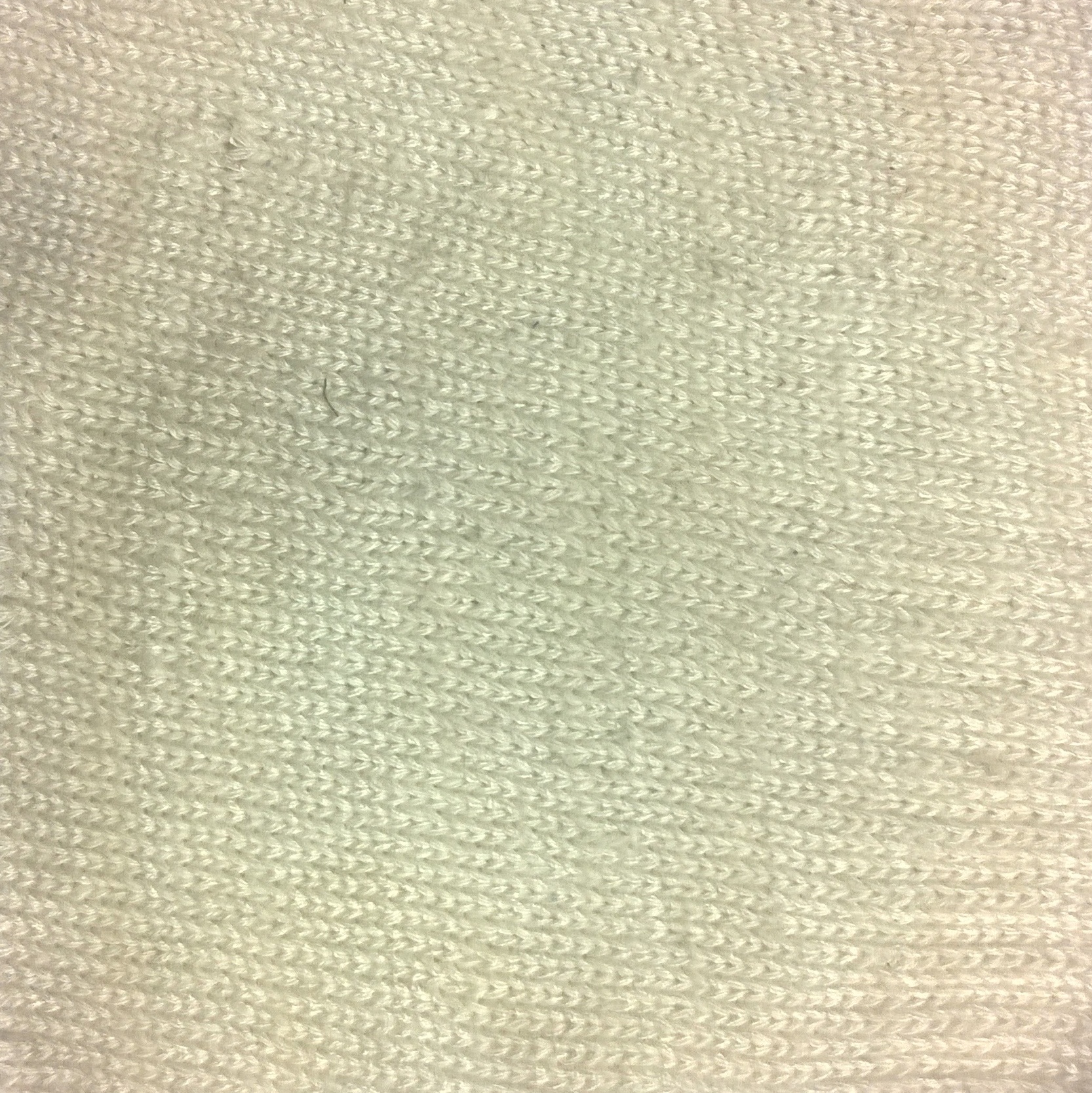

Supplement: Supplementary file 1 — Supplementary Information 2. [file 41598_2023_38929_MOESM1_ESM.zip › 51.jpg]

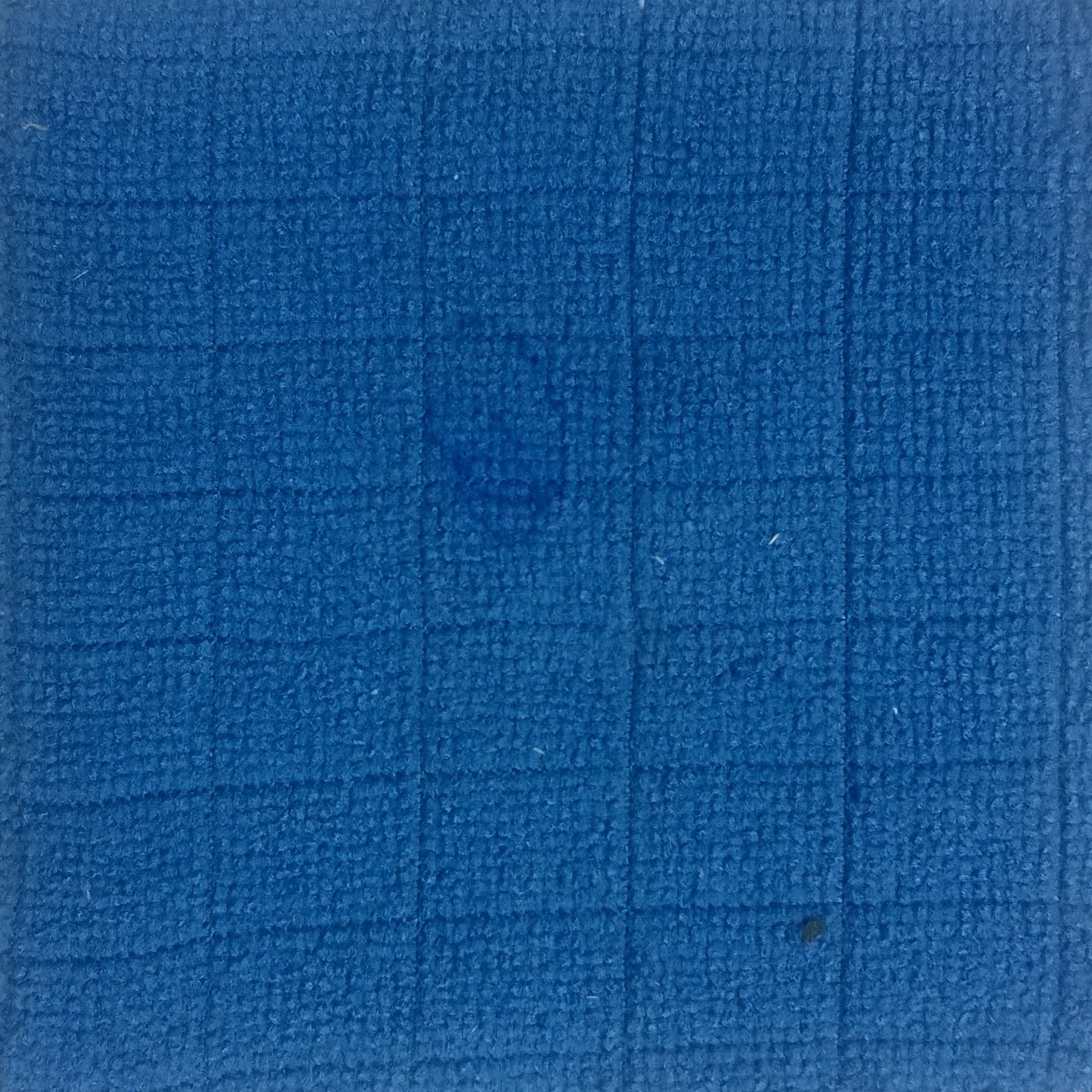

Supplement: Supplementary file 1 — Supplementary Information 2. [file 41598_2023_38929_MOESM1_ESM.zip › 52.jpg]

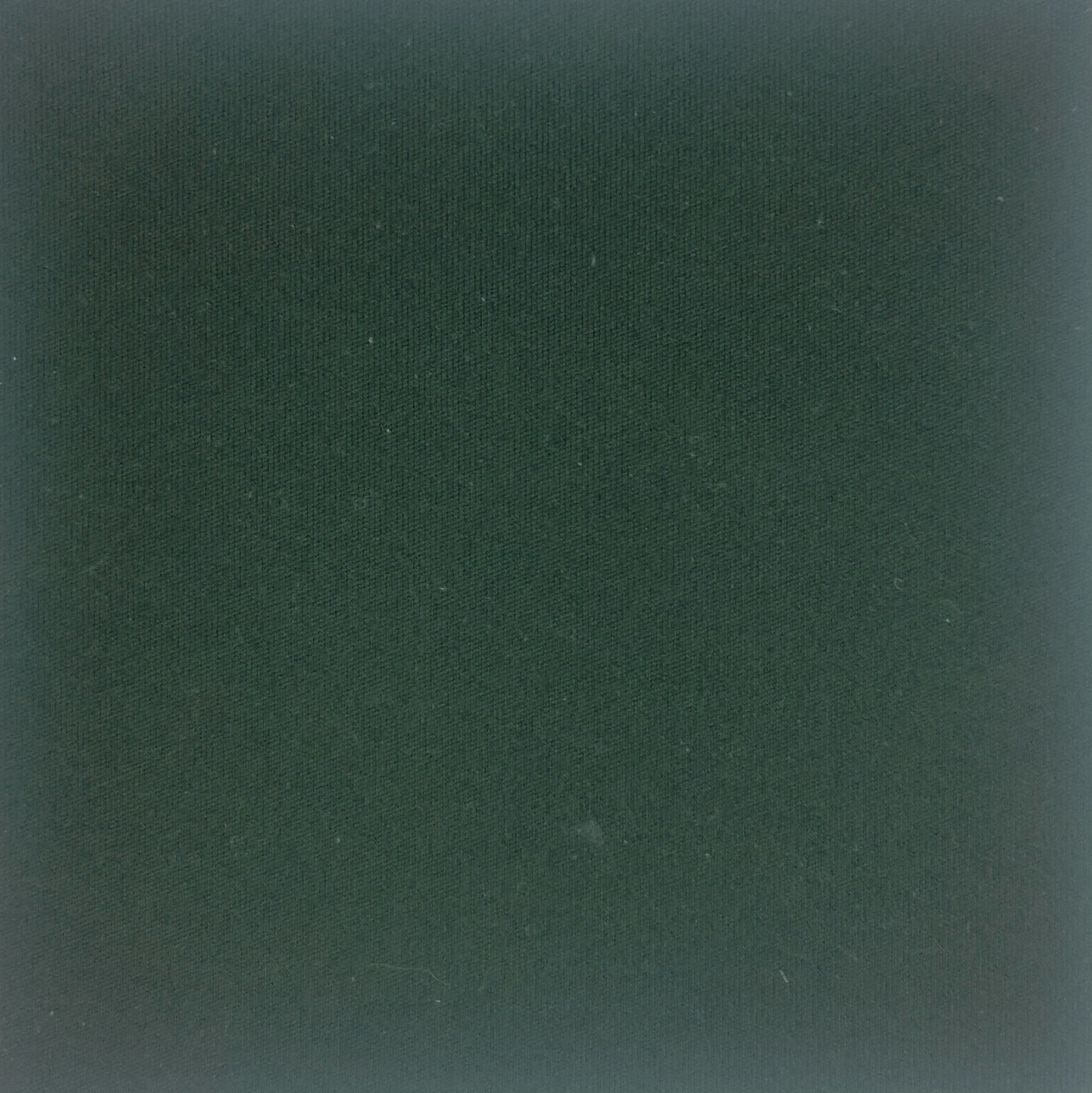

Supplement: Supplementary file 1 — Supplementary Information 2. [file 41598_2023_38929_MOESM1_ESM.zip › 53.jpg]

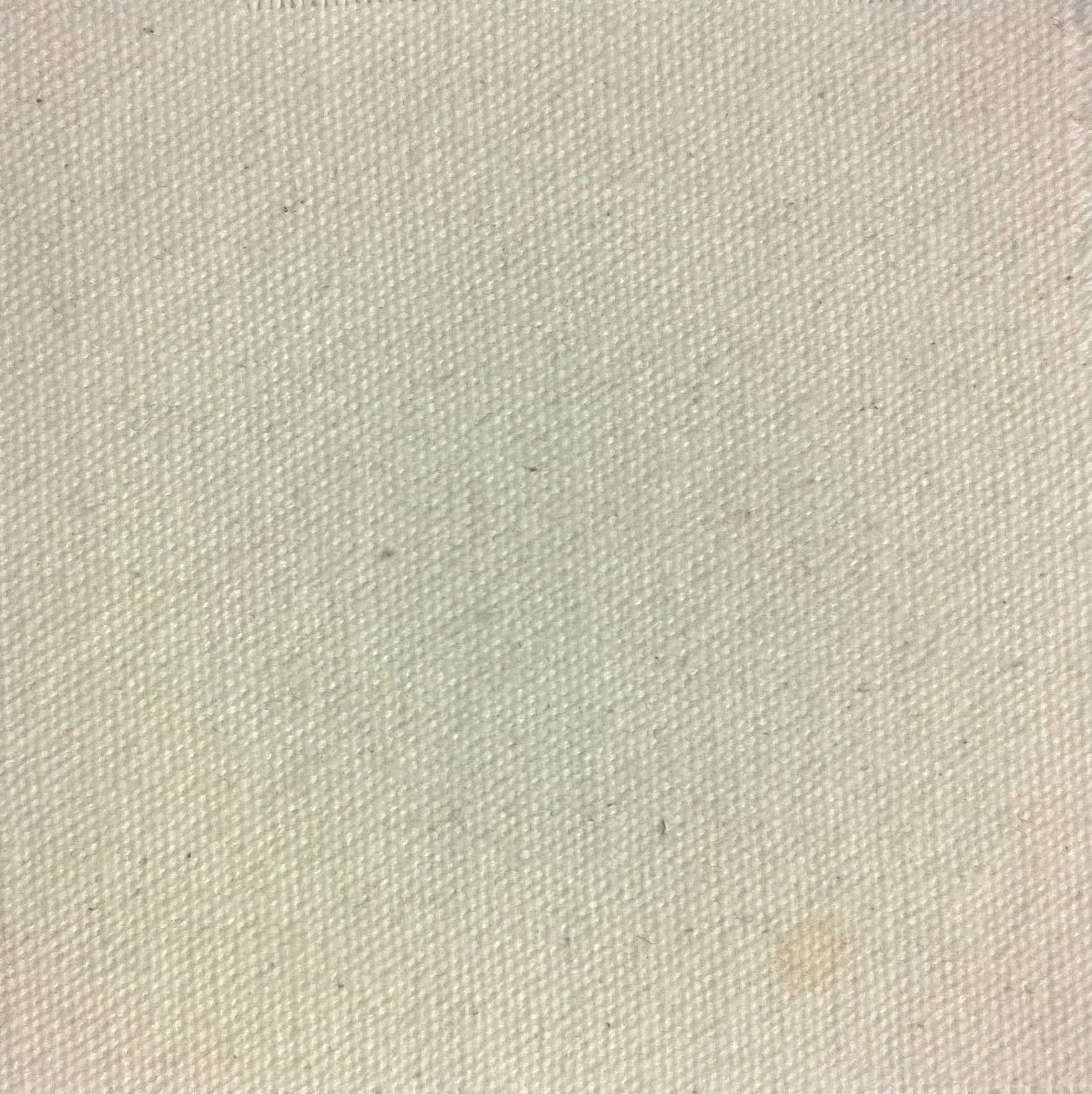

Supplement: Supplementary file 1 — Supplementary Information 2. [file 41598_2023_38929_MOESM1_ESM.zip › 54.jpg]

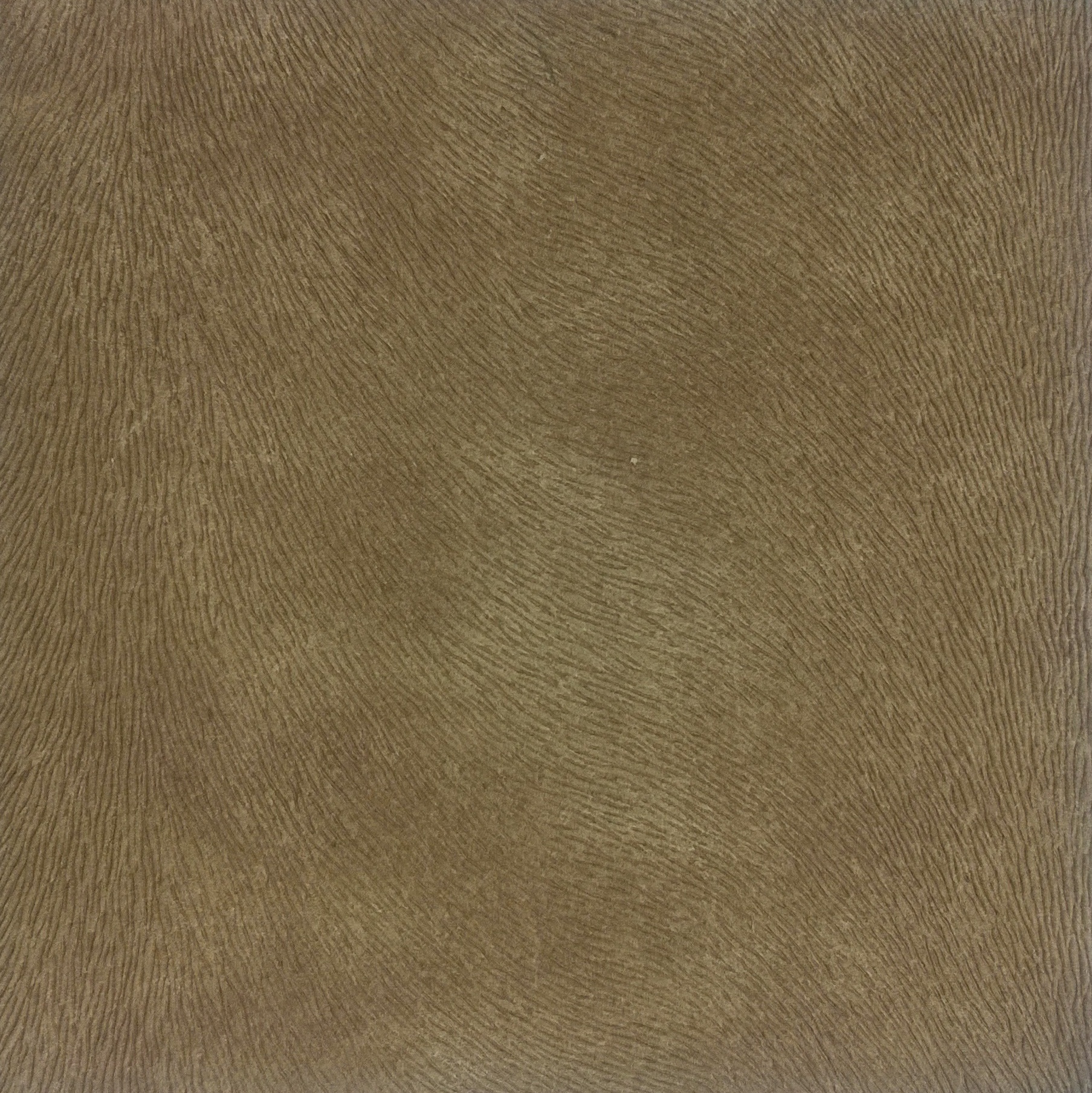

Supplement: Supplementary file 1 — Supplementary Information 2. [file 41598_2023_38929_MOESM1_ESM.zip › 55.jpg]

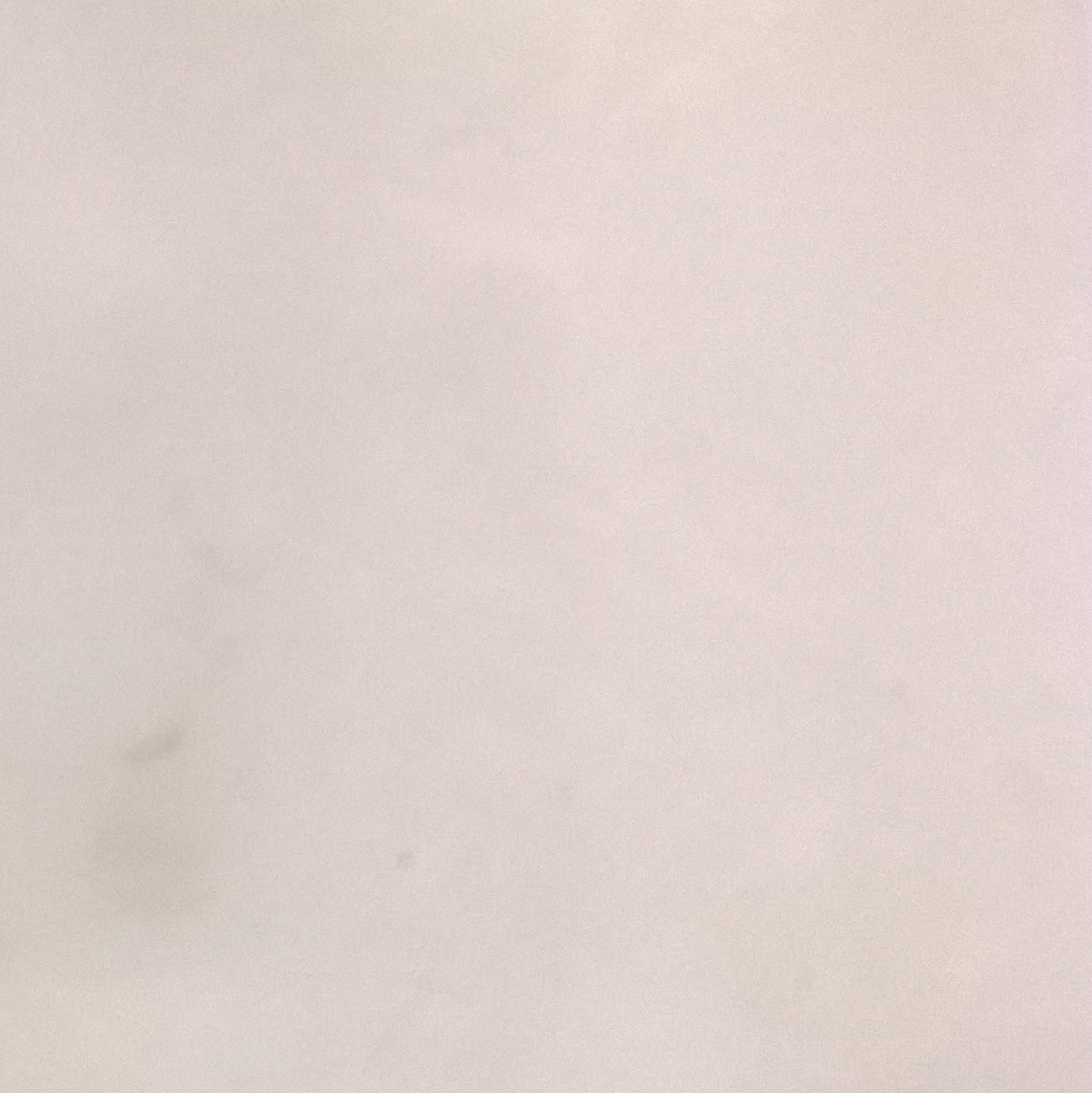

Supplement: Supplementary file 1 — Supplementary Information 2. [file 41598_2023_38929_MOESM1_ESM.zip › 56.jpg]

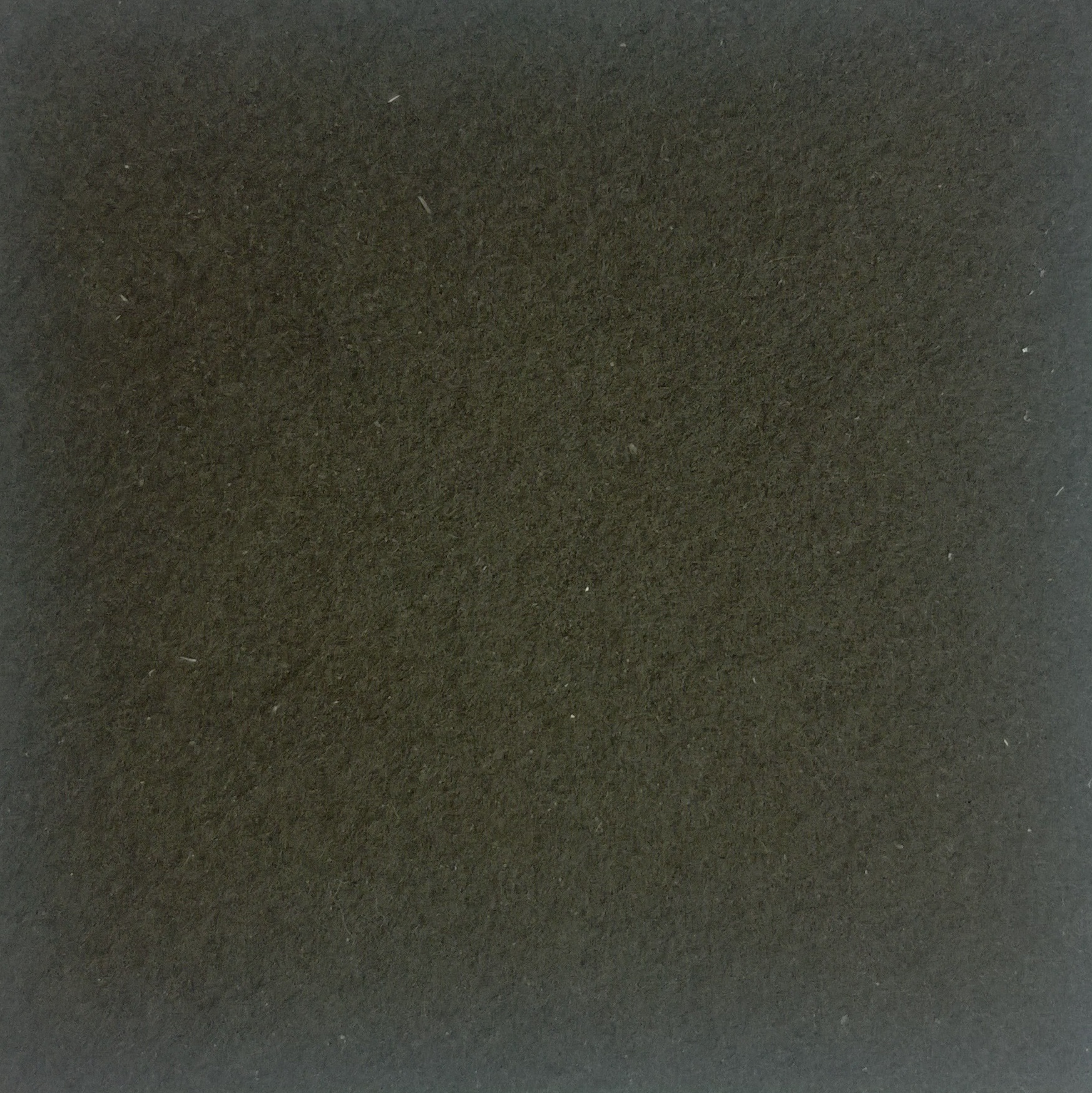

Supplement: Supplementary file 1 — Supplementary Information 2. [file 41598_2023_38929_MOESM1_ESM.zip › 57.jpg]

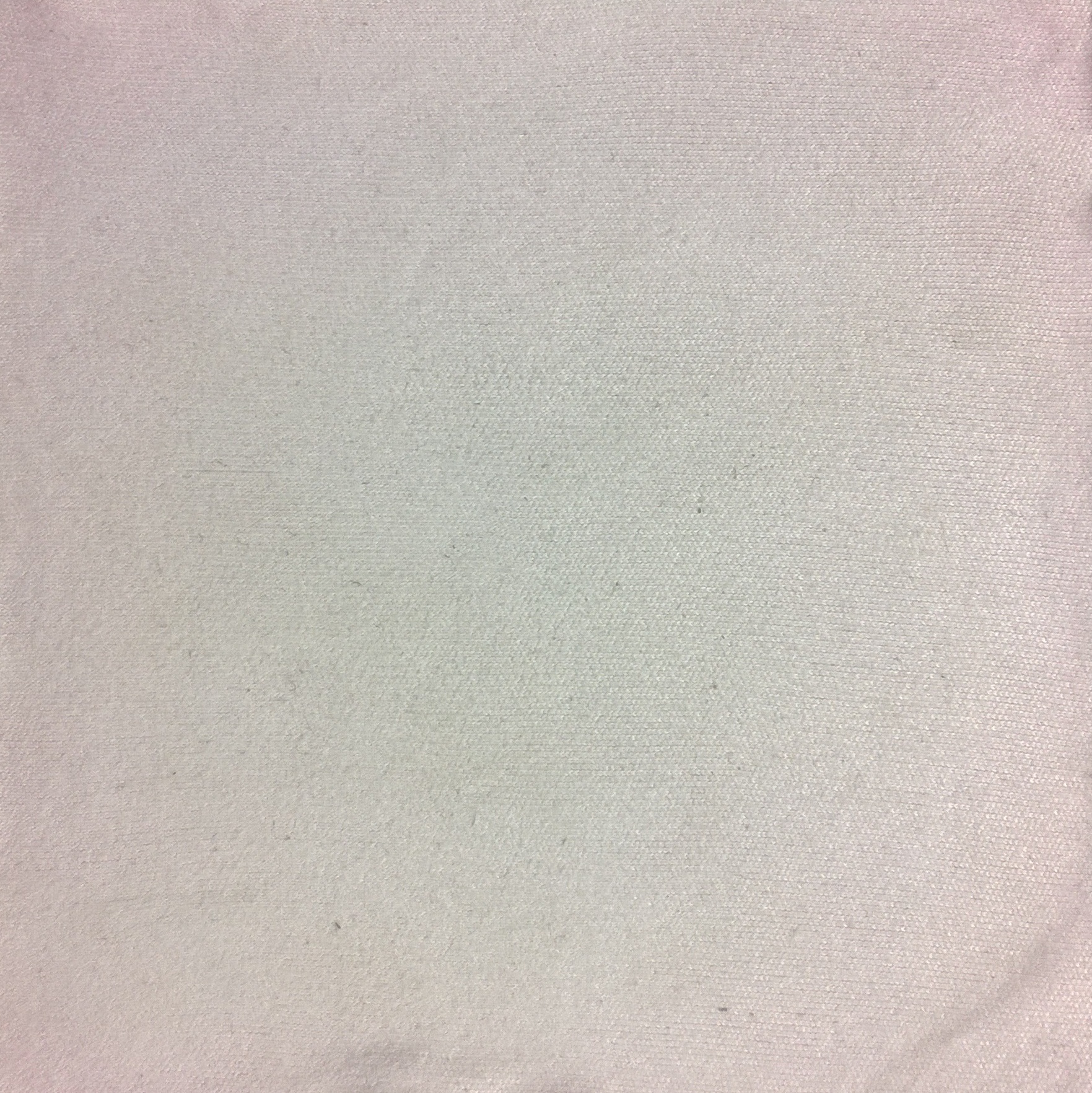

Supplement: Supplementary file 1 — Supplementary Information 2. [file 41598_2023_38929_MOESM1_ESM.zip › 58.jpg]

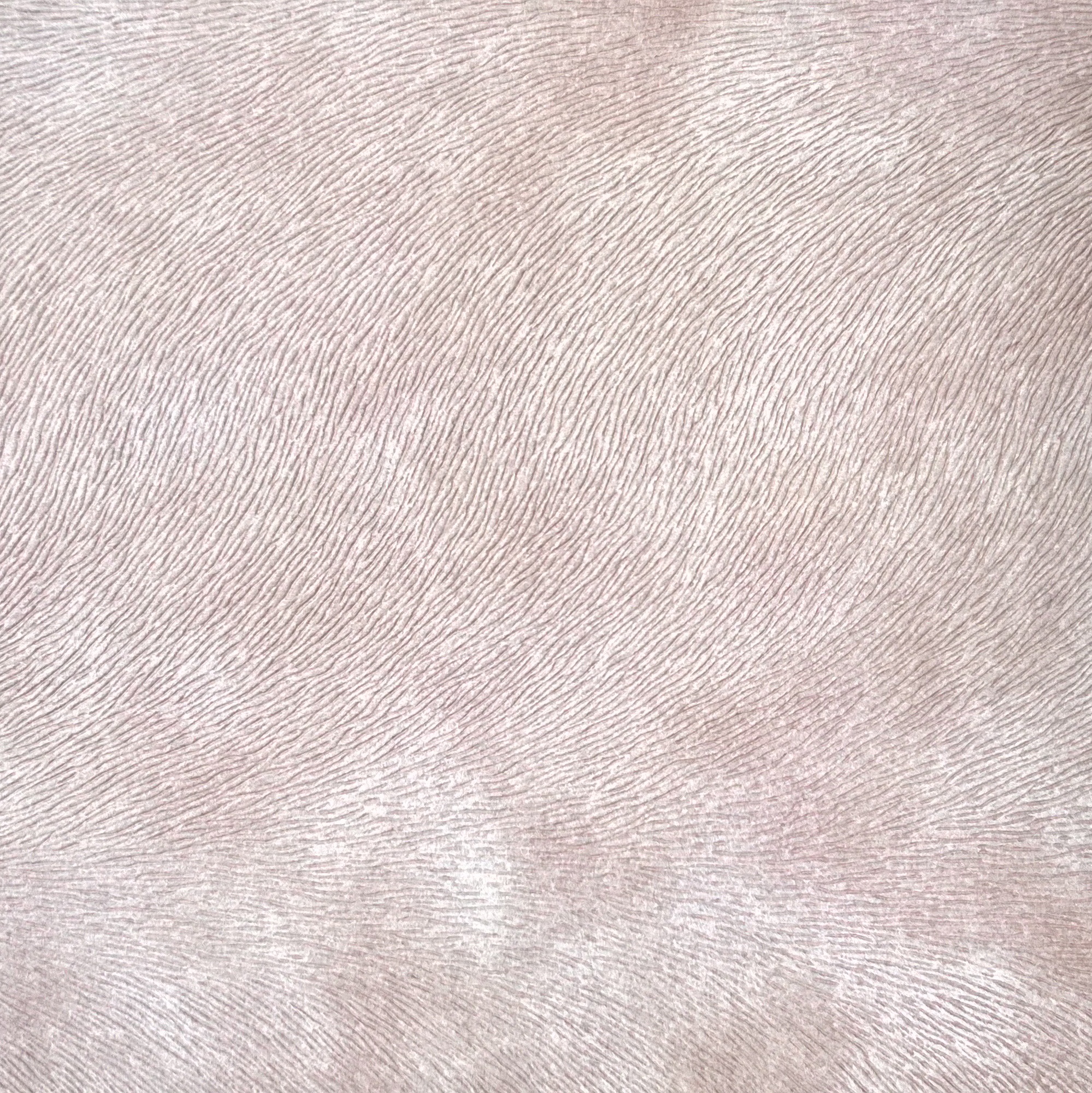

Supplement: Supplementary file 1 — Supplementary Information 2. [file 41598_2023_38929_MOESM1_ESM.zip › 59.jpg]

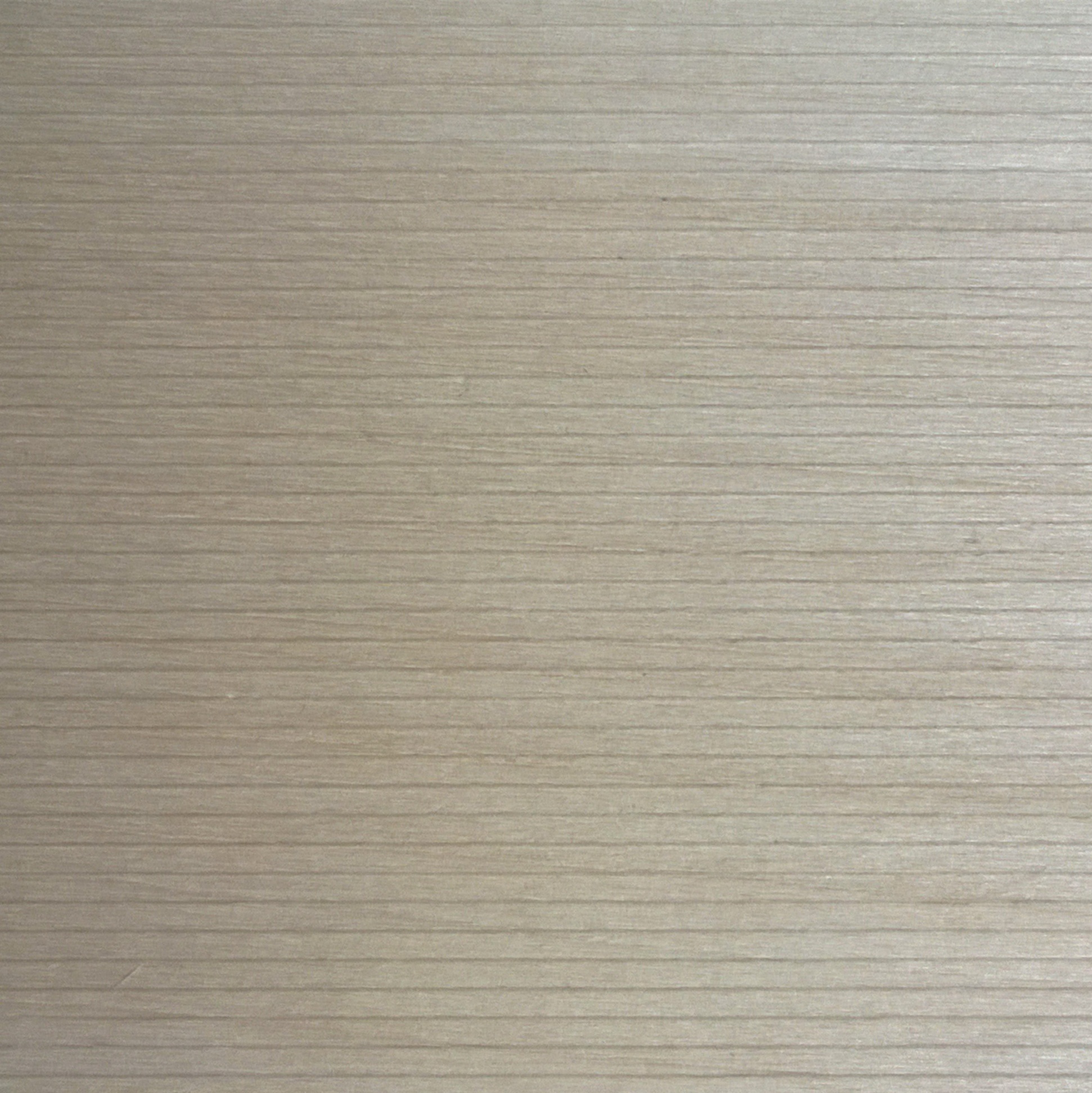

Supplement: Supplementary file 1 — Supplementary Information 2. [file 41598_2023_38929_MOESM1_ESM.zip › 6.jpg]

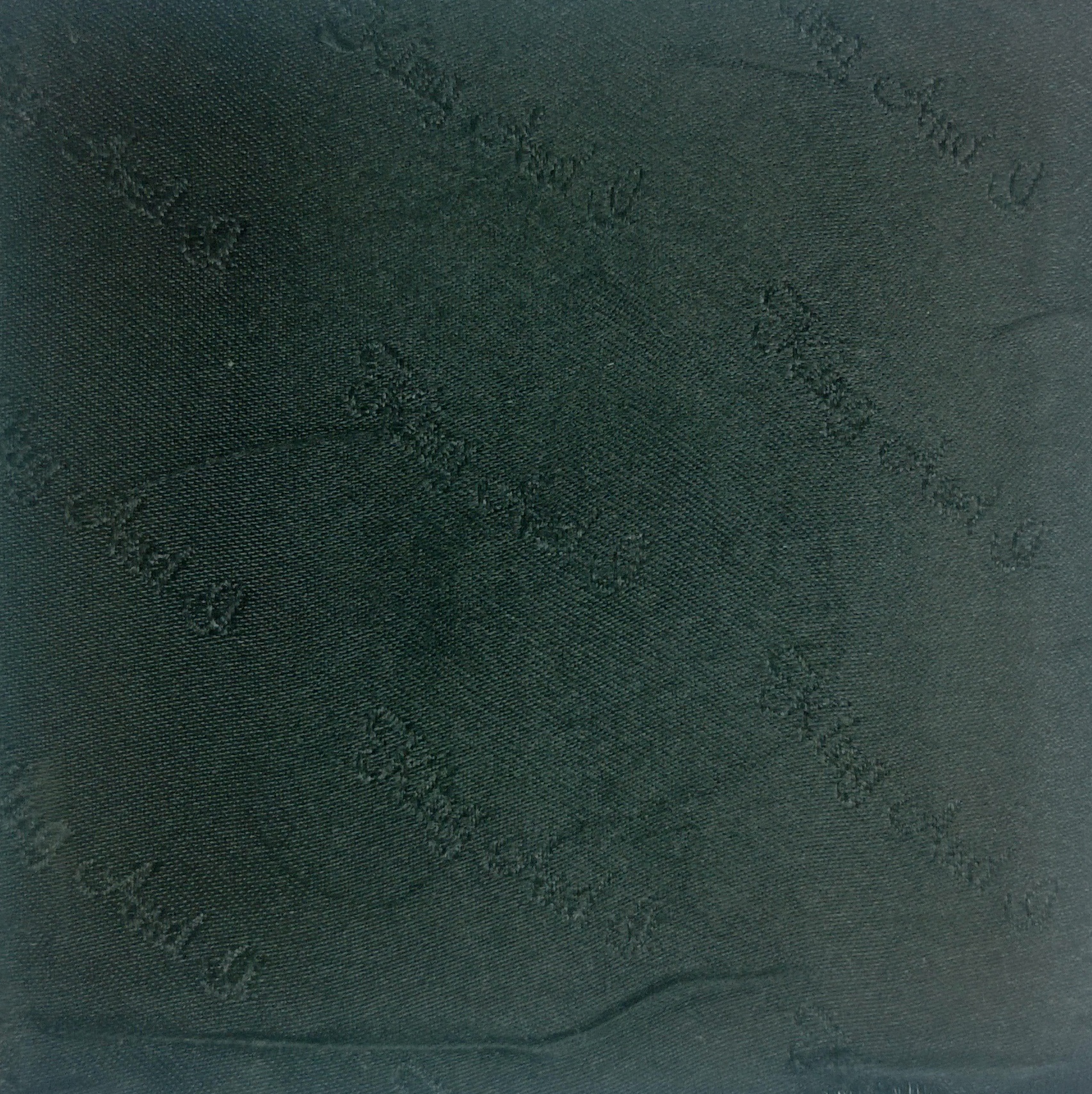

Supplement: Supplementary file 1 — Supplementary Information 2. [file 41598_2023_38929_MOESM1_ESM.zip › 60.jpg]

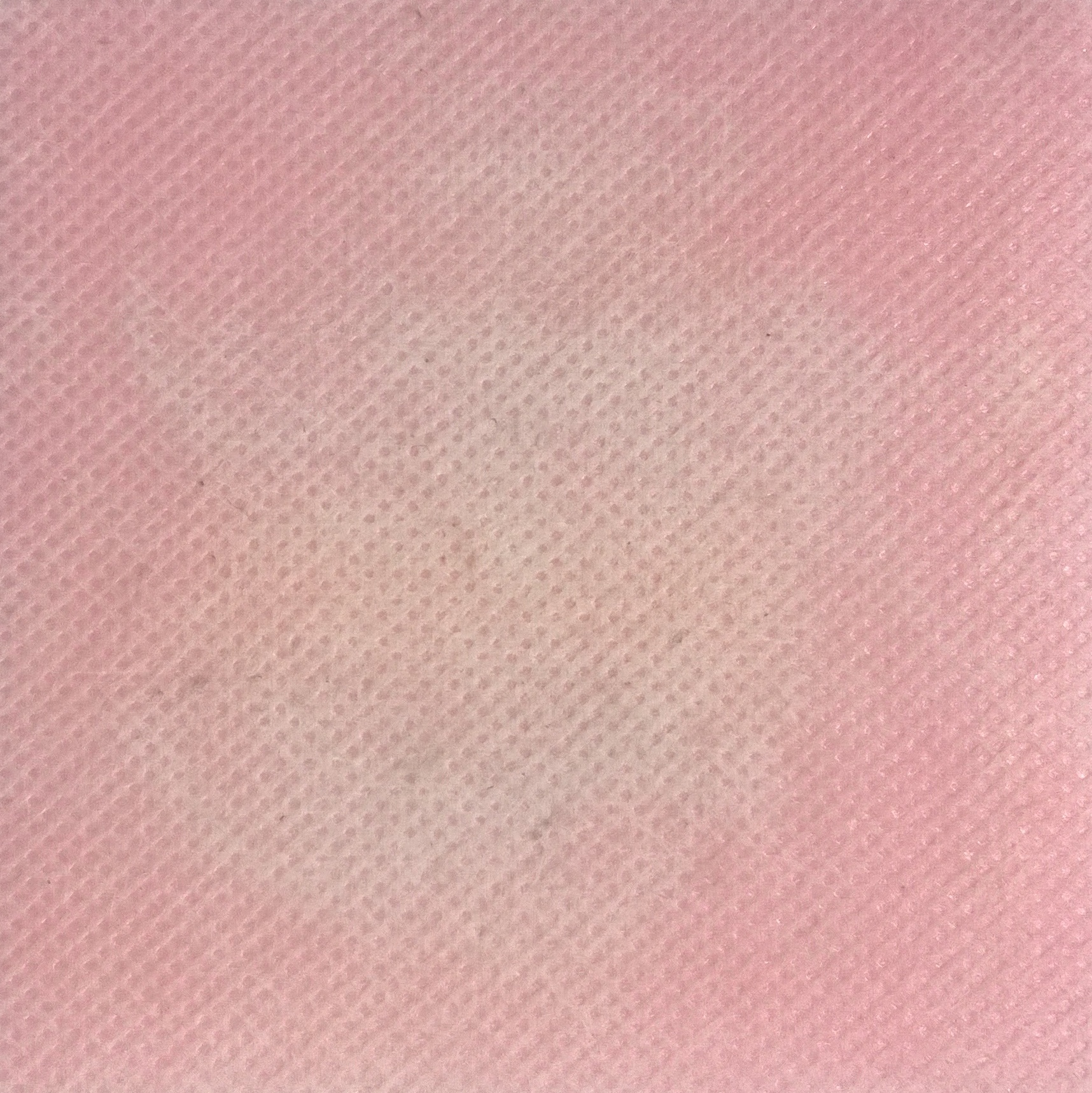

Supplement: Supplementary file 1 — Supplementary Information 2. [file 41598_2023_38929_MOESM1_ESM.zip › 61.jpg]

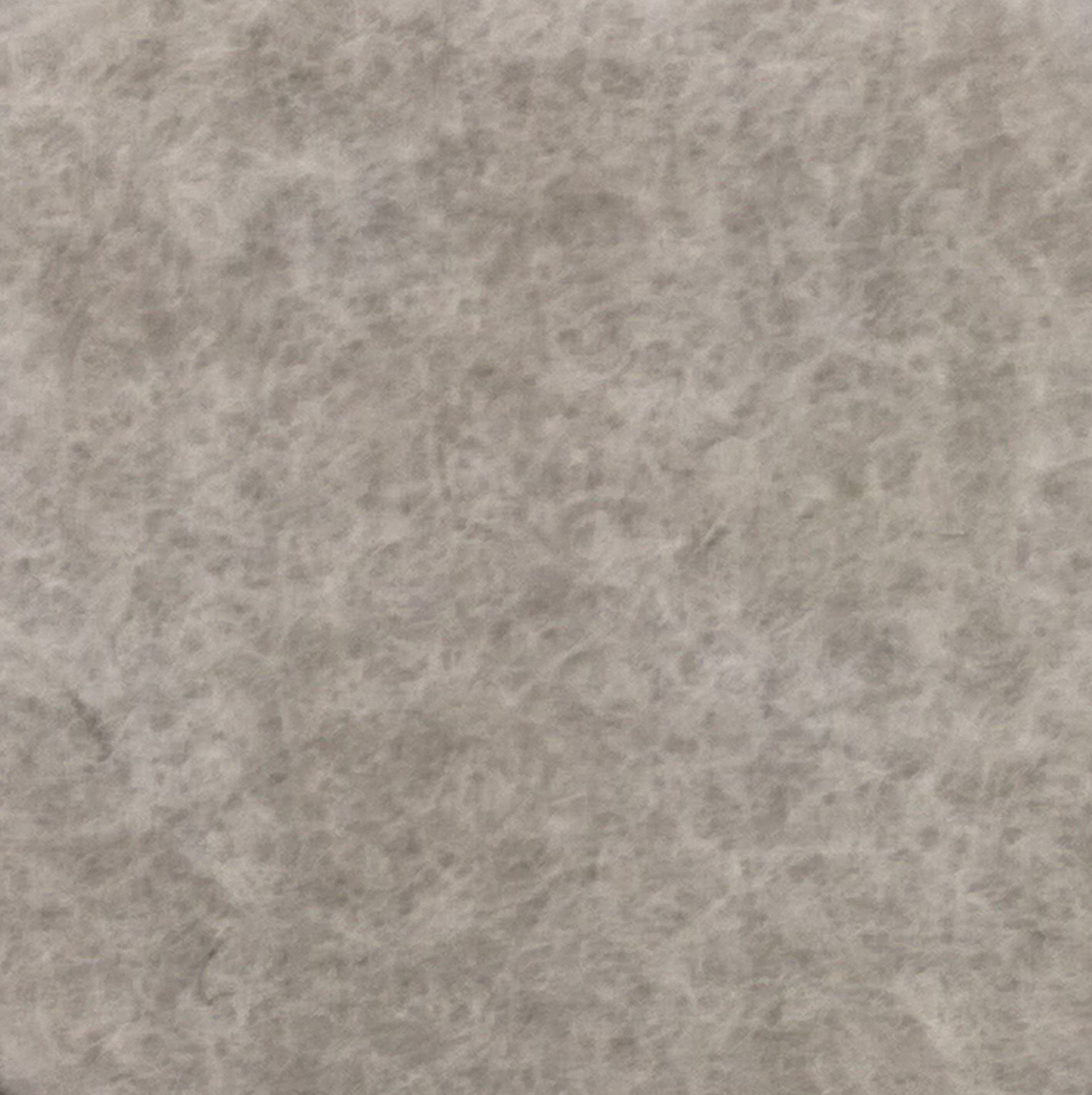

Supplement: Supplementary file 1 — Supplementary Information 2. [file 41598_2023_38929_MOESM1_ESM.zip › 62.jpg]

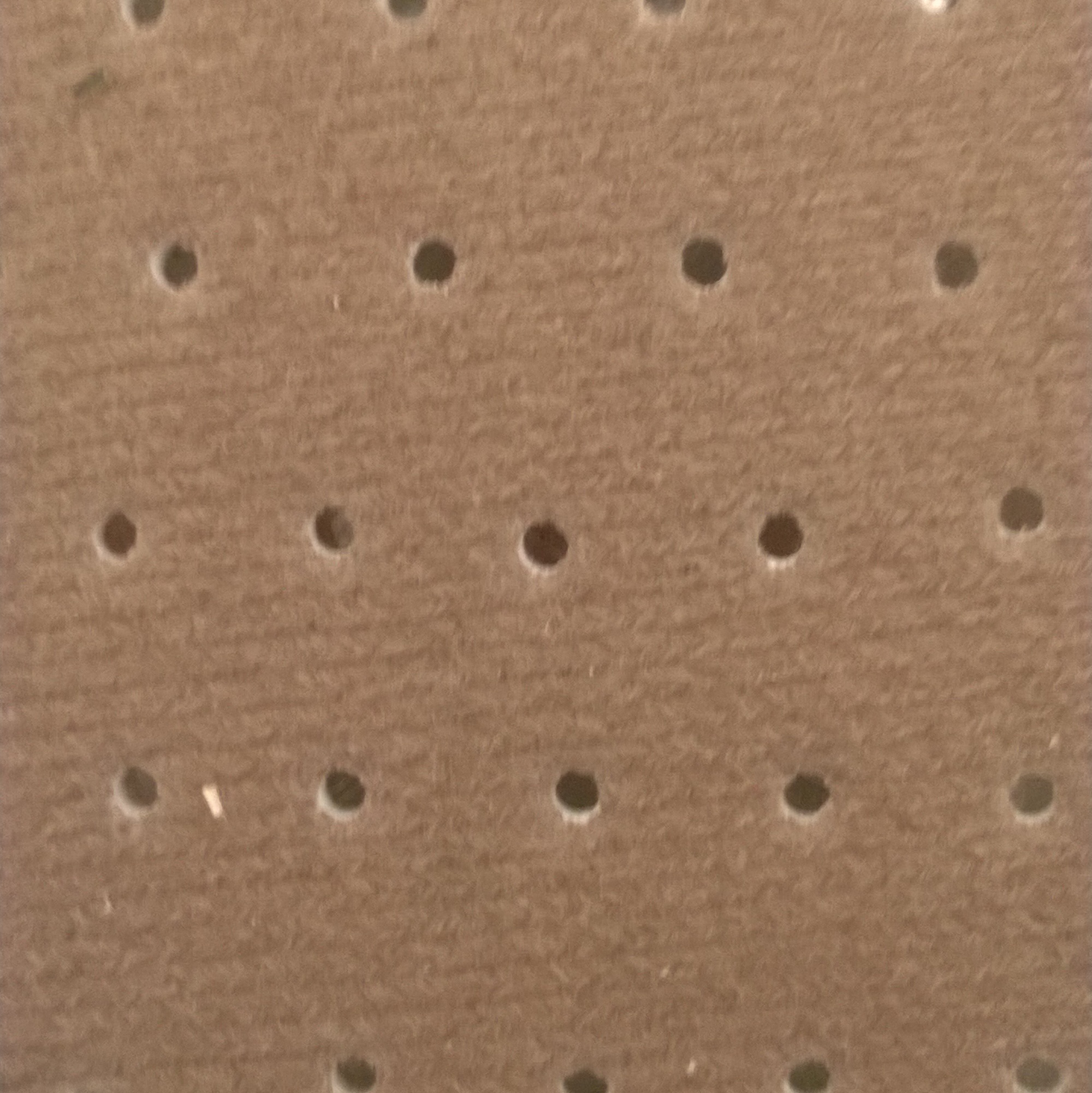

Supplement: Supplementary file 1 — Supplementary Information 2. [file 41598_2023_38929_MOESM1_ESM.zip › 63.jpg]

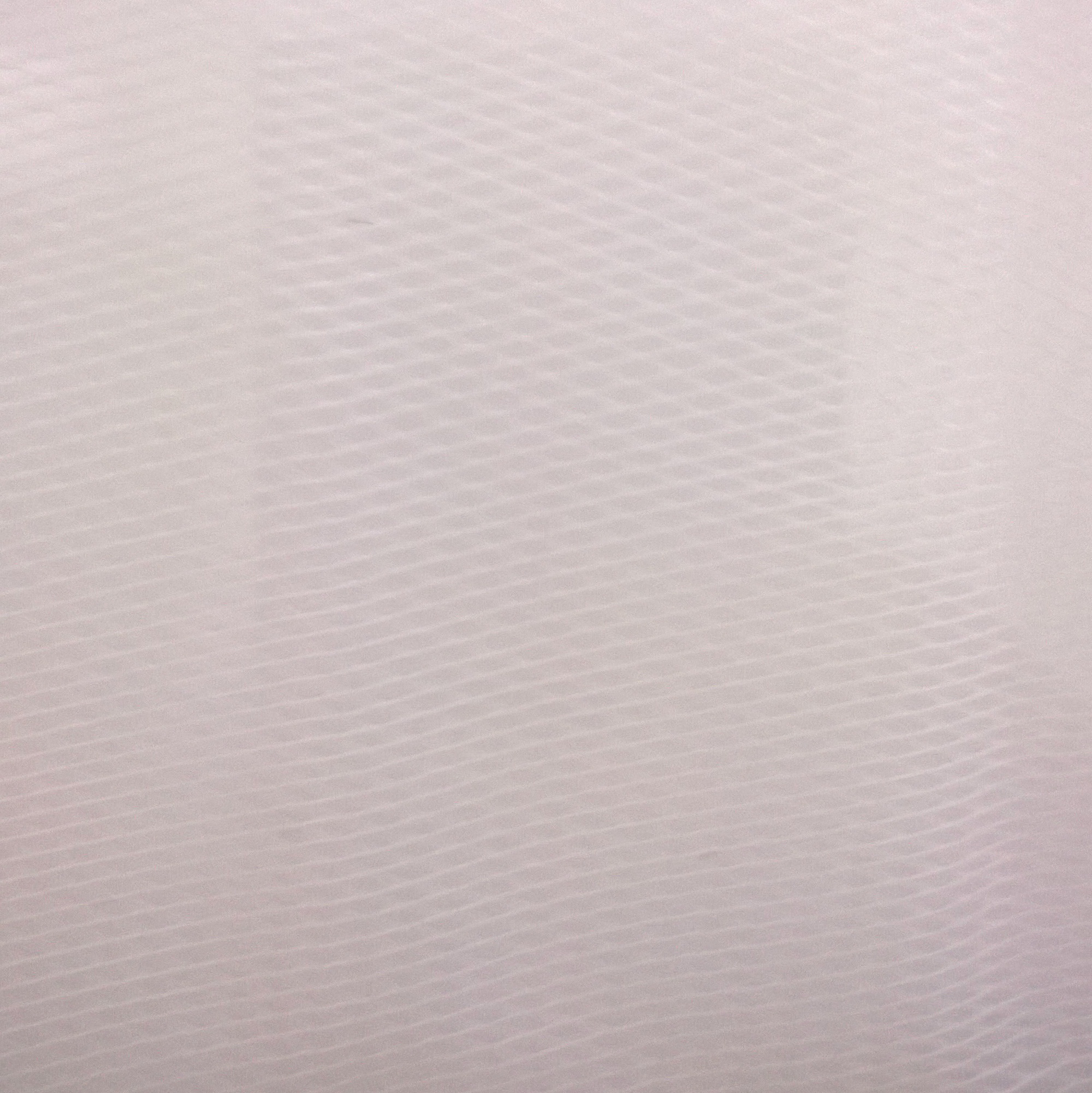

Supplement: Supplementary file 1 — Supplementary Information 2. [file 41598_2023_38929_MOESM1_ESM.zip › 64.jpg]

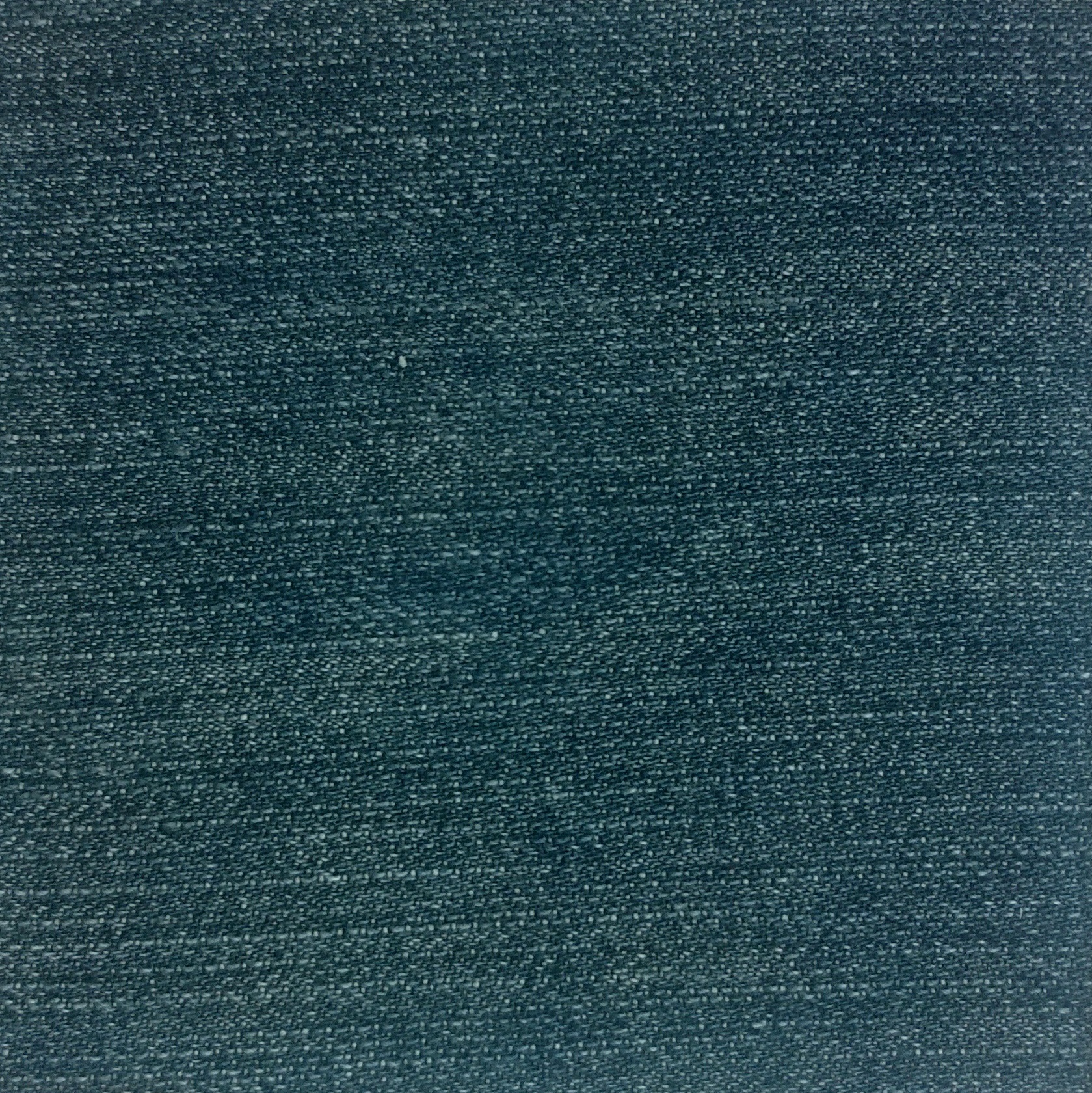

Supplement: Supplementary file 1 — Supplementary Information 2. [file 41598_2023_38929_MOESM1_ESM.zip › 65.jpg]

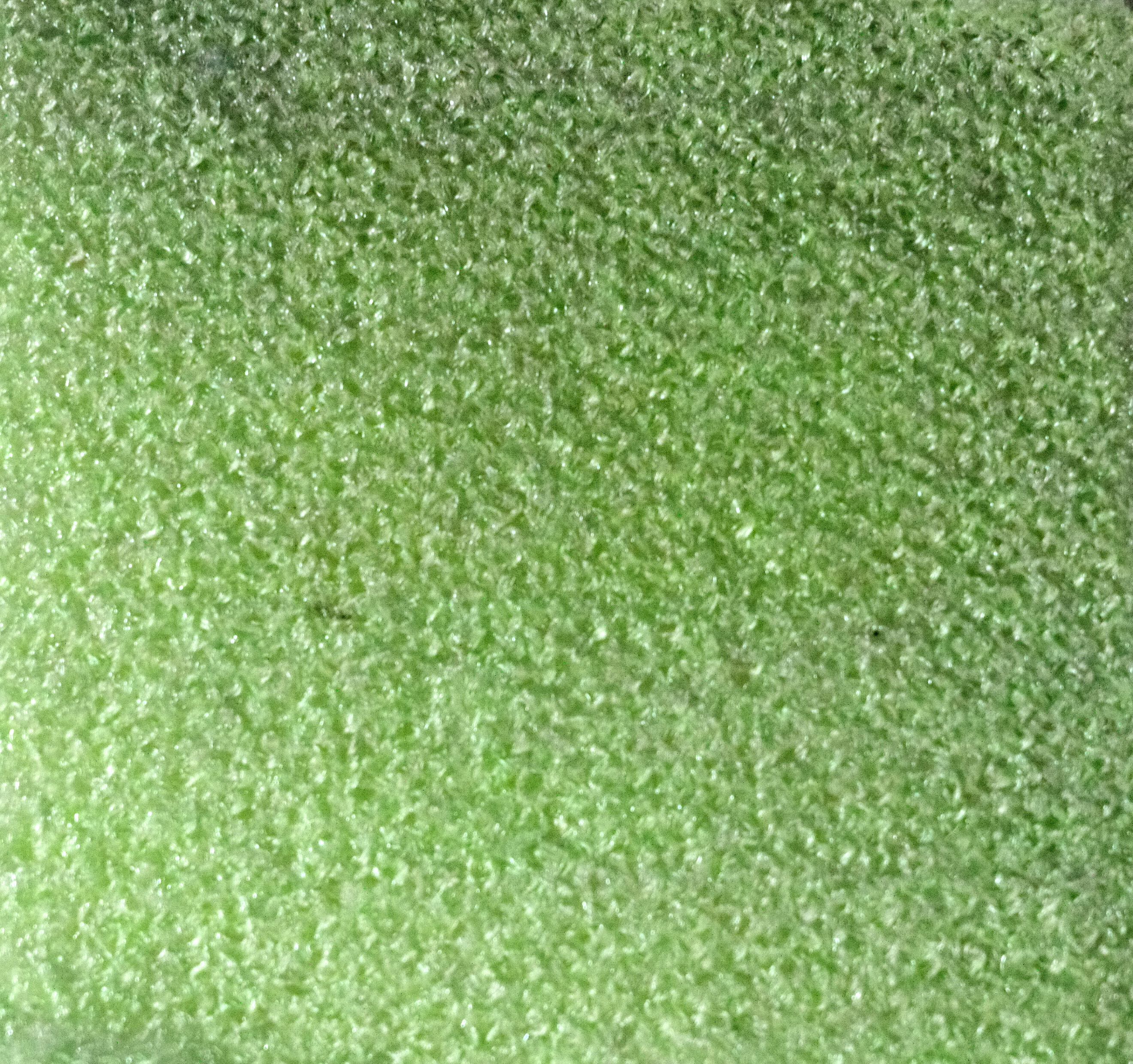

Supplement: Supplementary file 1 — Supplementary Information 2. [file 41598_2023_38929_MOESM1_ESM.zip › 66.JPG]

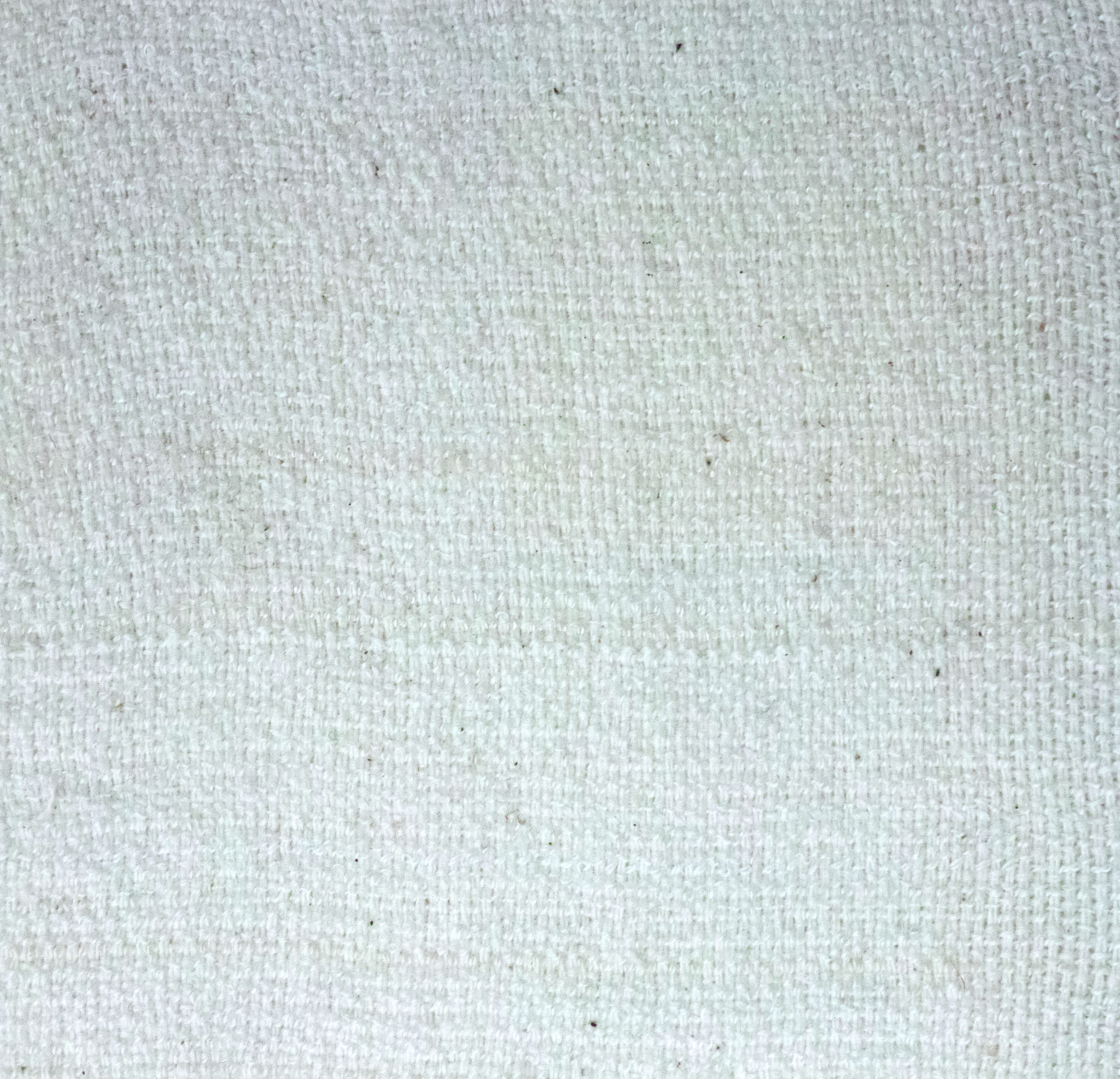

Supplement: Supplementary file 1 — Supplementary Information 2. [file 41598_2023_38929_MOESM1_ESM.zip › 67.JPG]

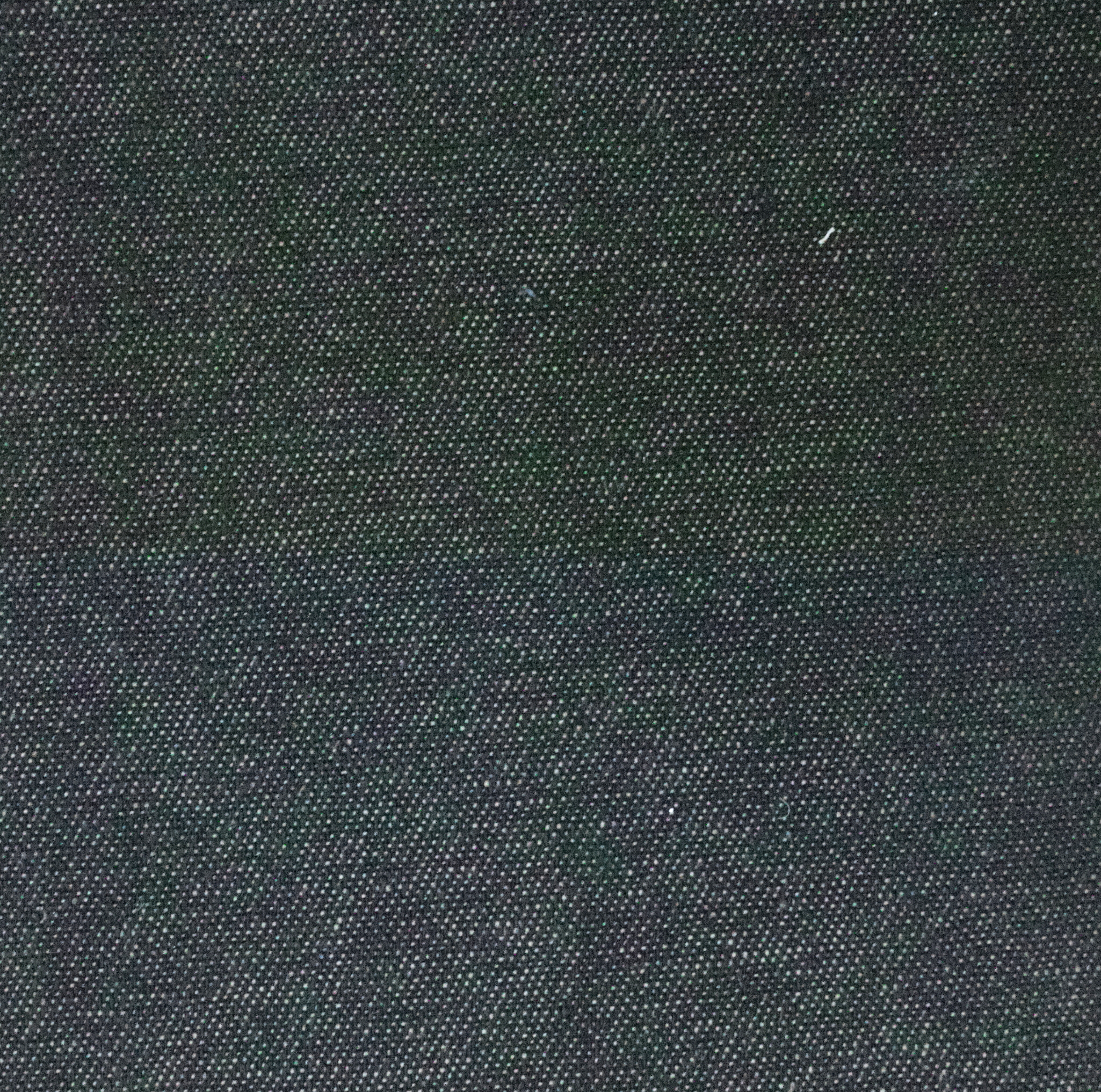

Supplement: Supplementary file 1 — Supplementary Information 2. [file 41598_2023_38929_MOESM1_ESM.zip › 68.JPG]

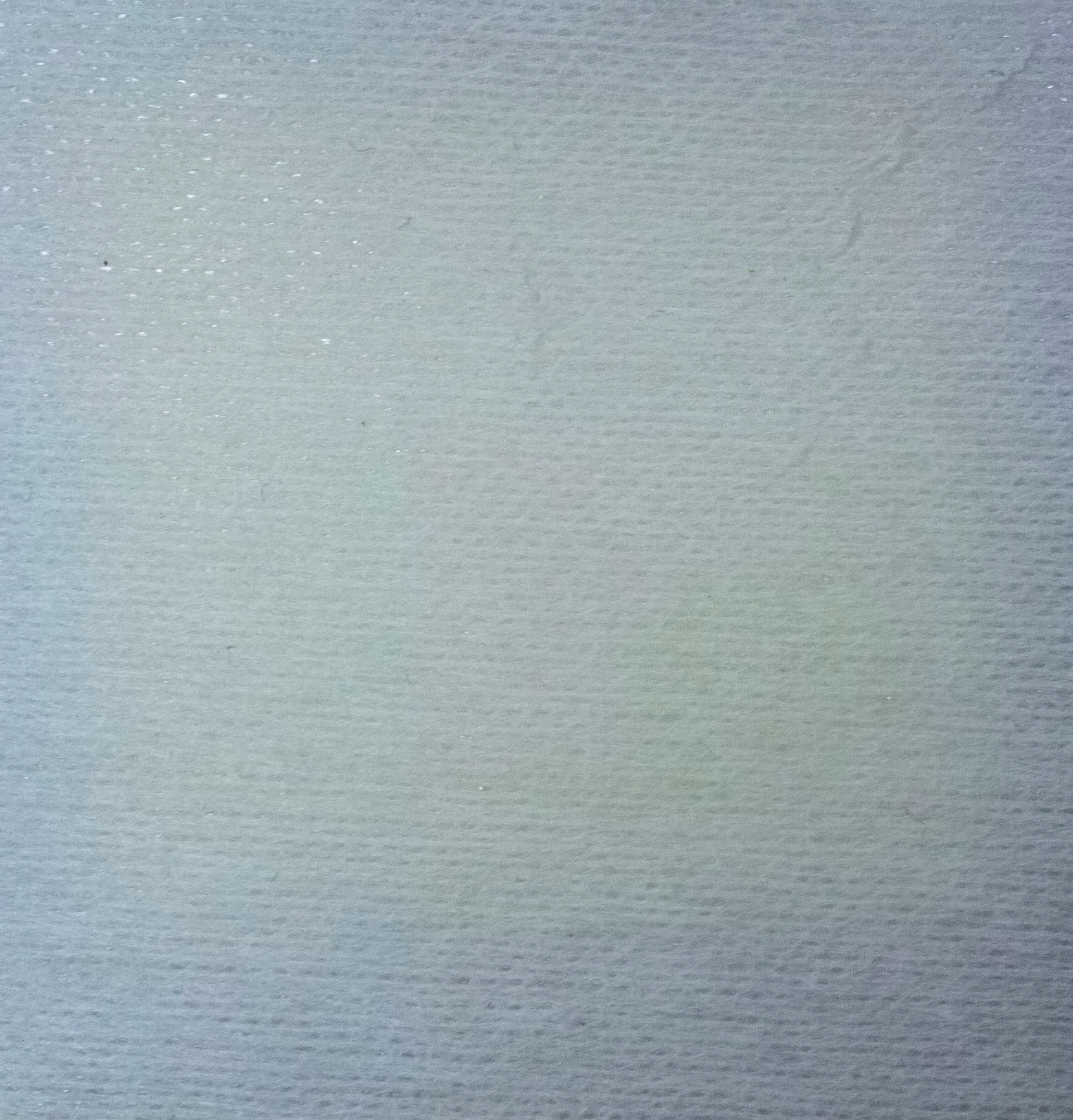

Supplement: Supplementary file 1 — Supplementary Information 2. [file 41598_2023_38929_MOESM1_ESM.zip › 69.JPG]

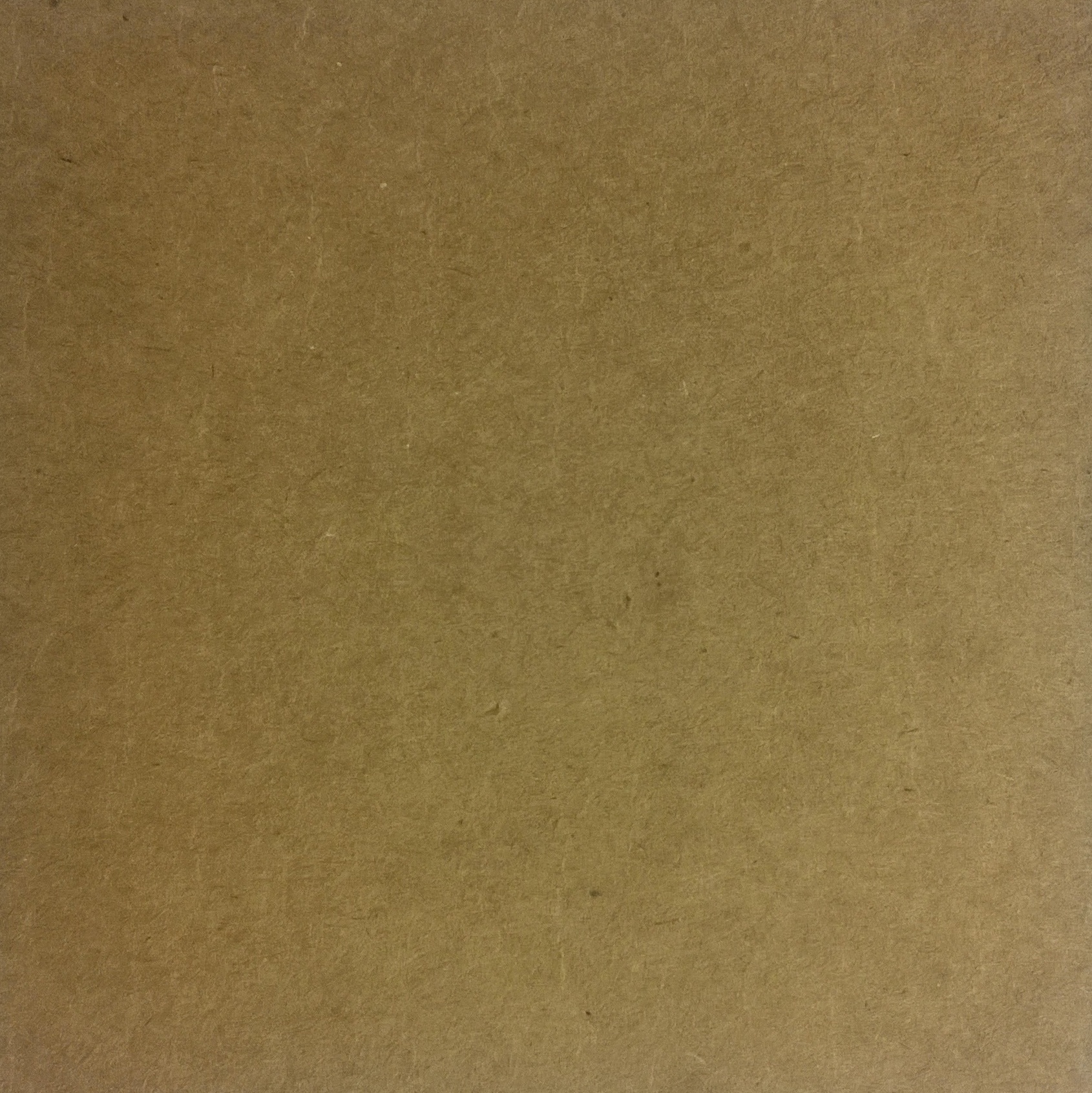

Supplement: Supplementary file 1 — Supplementary Information 2. [file 41598_2023_38929_MOESM1_ESM.zip › 7.jpg]

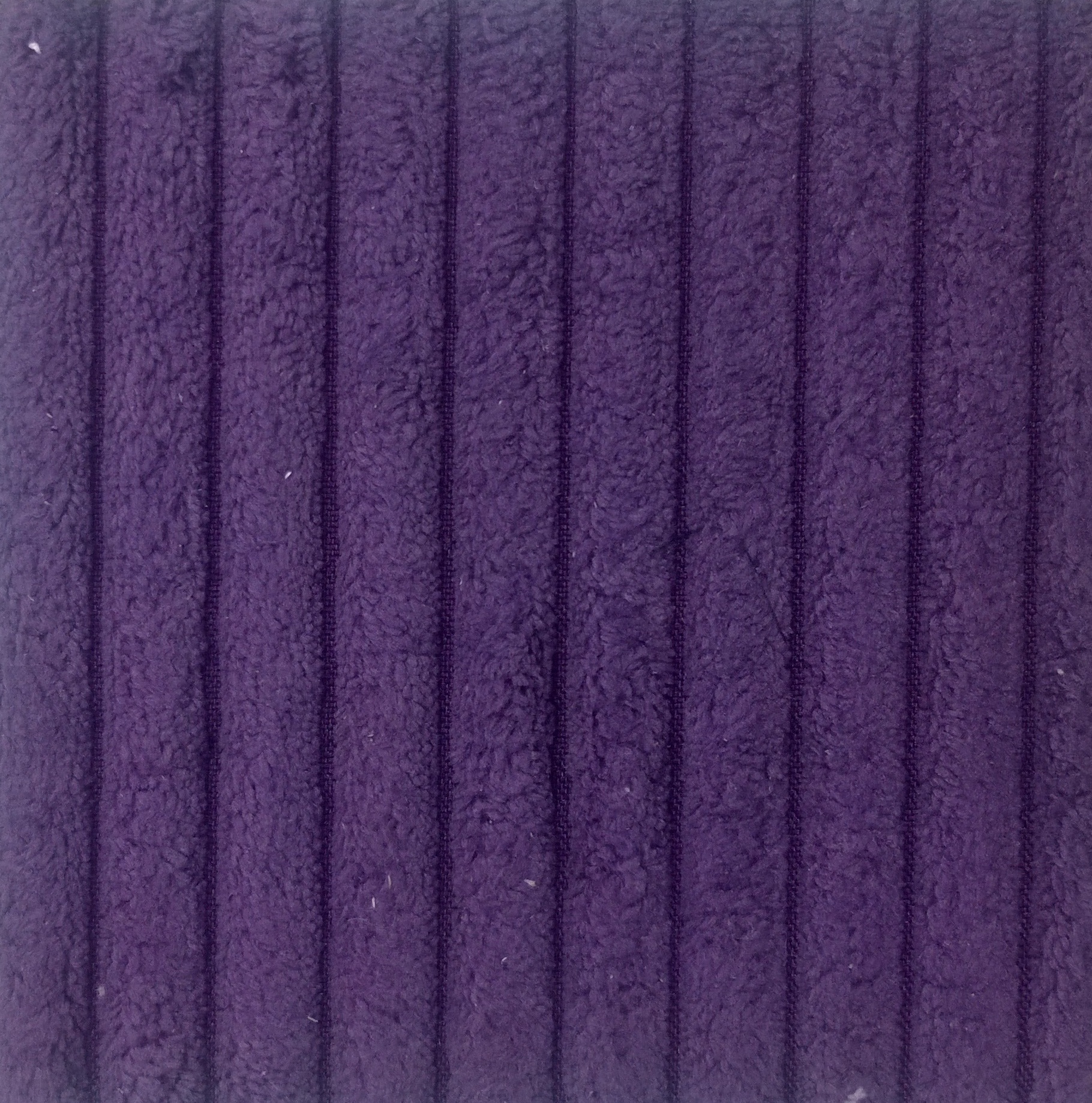

Supplement: Supplementary file 1 — Supplementary Information 2. [file 41598_2023_38929_MOESM1_ESM.zip › 70.jpg]

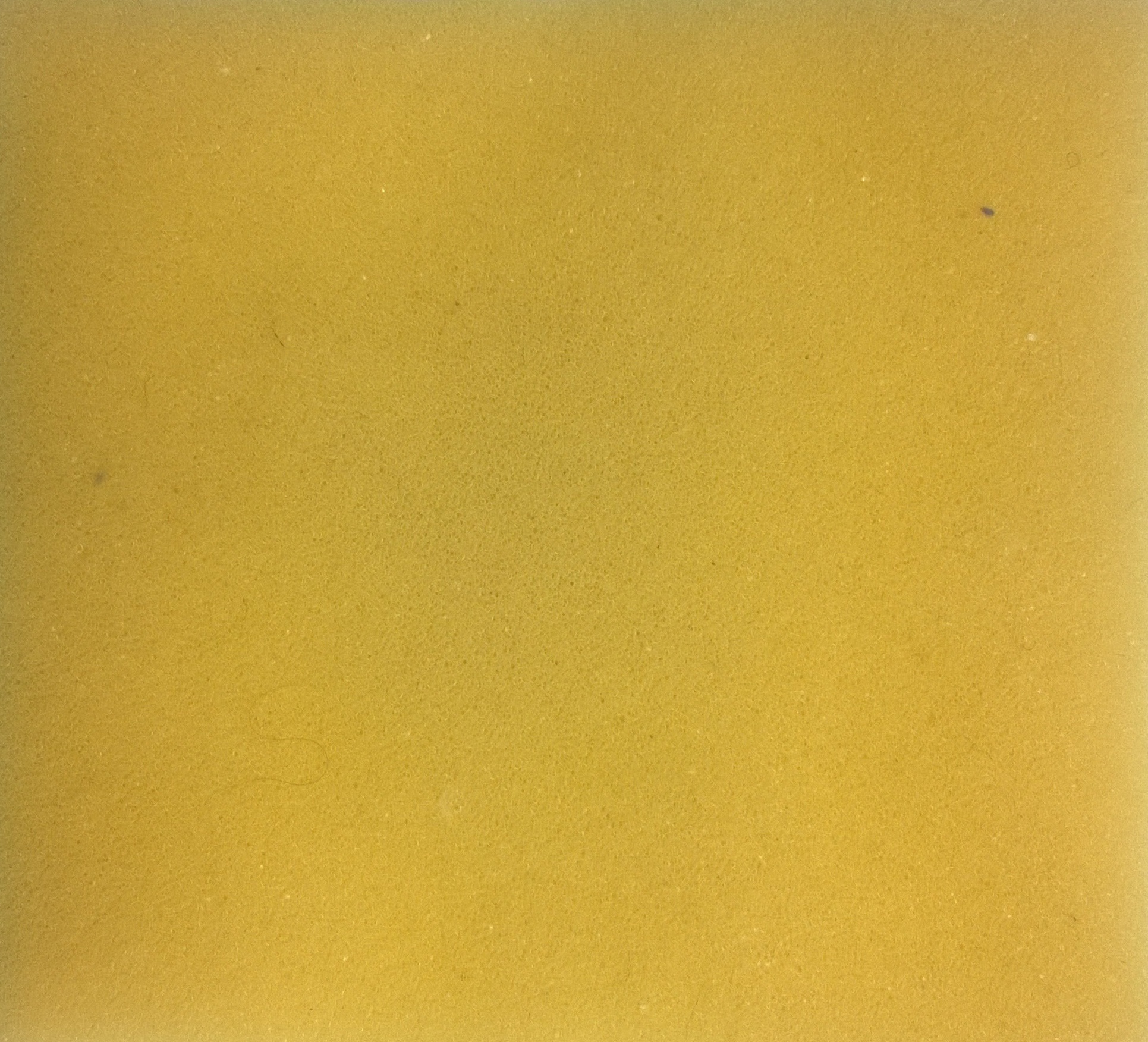

Supplement: Supplementary file 1 — Supplementary Information 2. [file 41598_2023_38929_MOESM1_ESM.zip › 71.jpg]

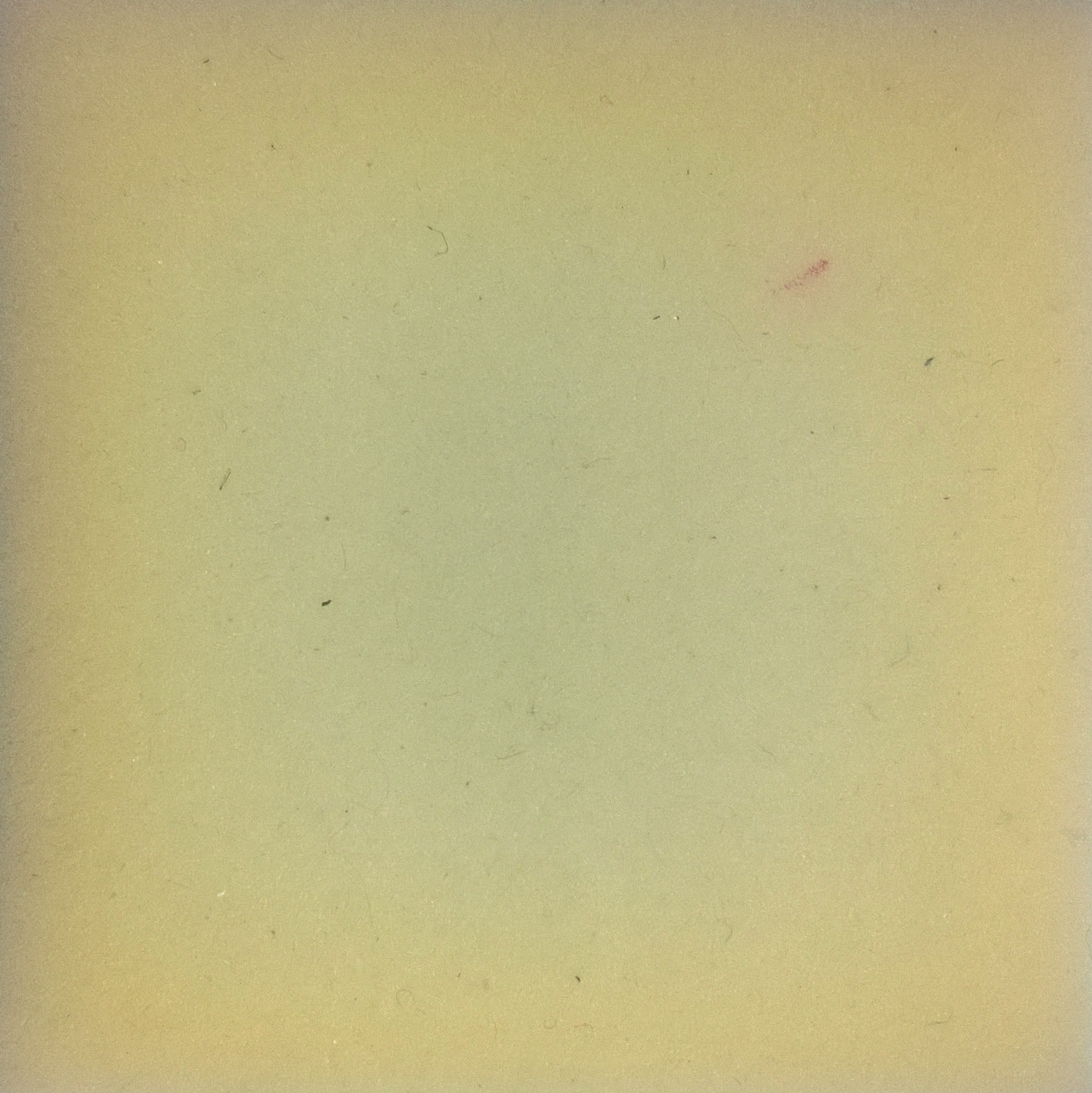

Supplement: Supplementary file 1 — Supplementary Information 2. [file 41598_2023_38929_MOESM1_ESM.zip › 72.jpg]

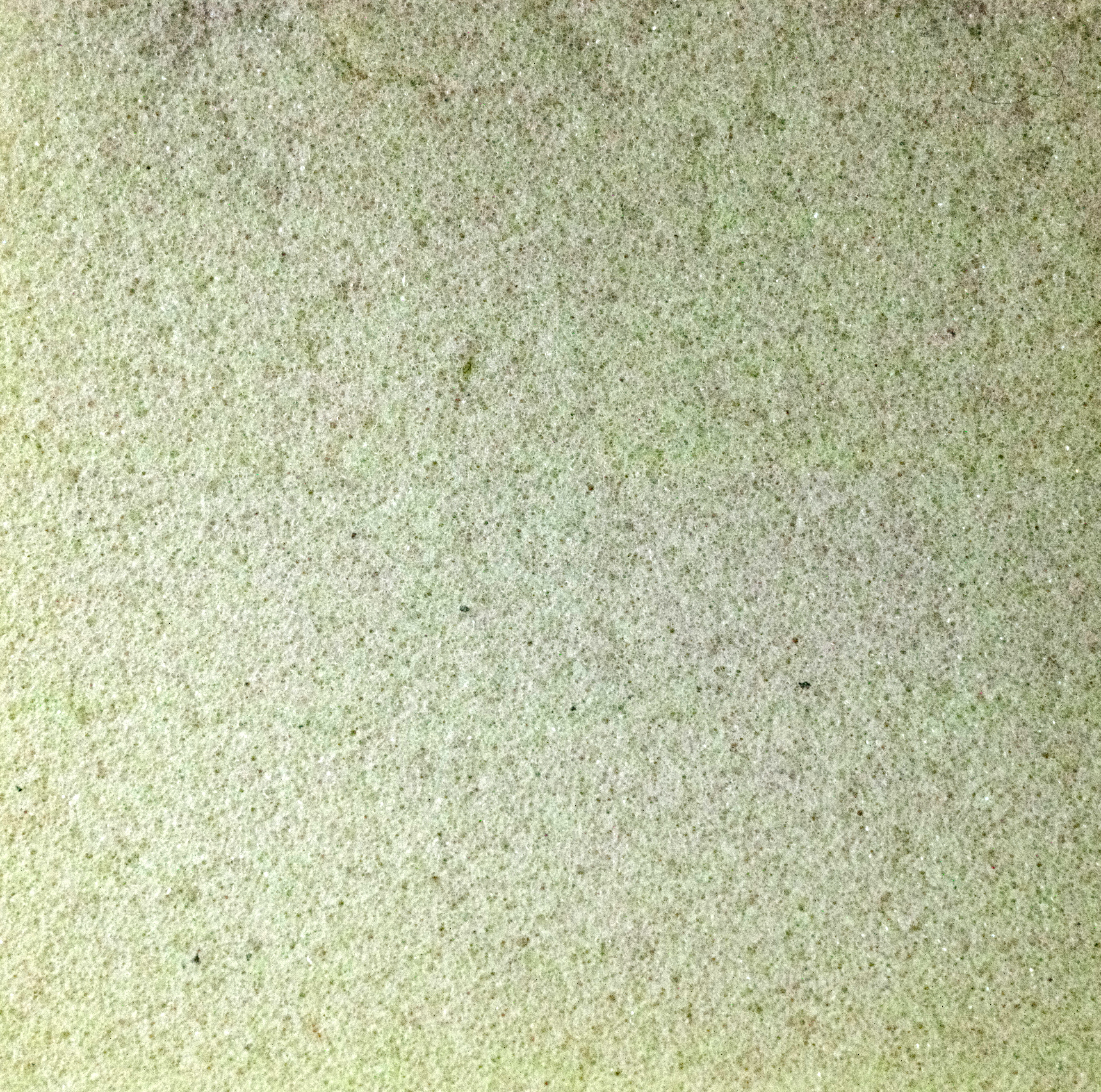

Supplement: Supplementary file 1 — Supplementary Information 2. [file 41598_2023_38929_MOESM1_ESM.zip › 73.JPG]

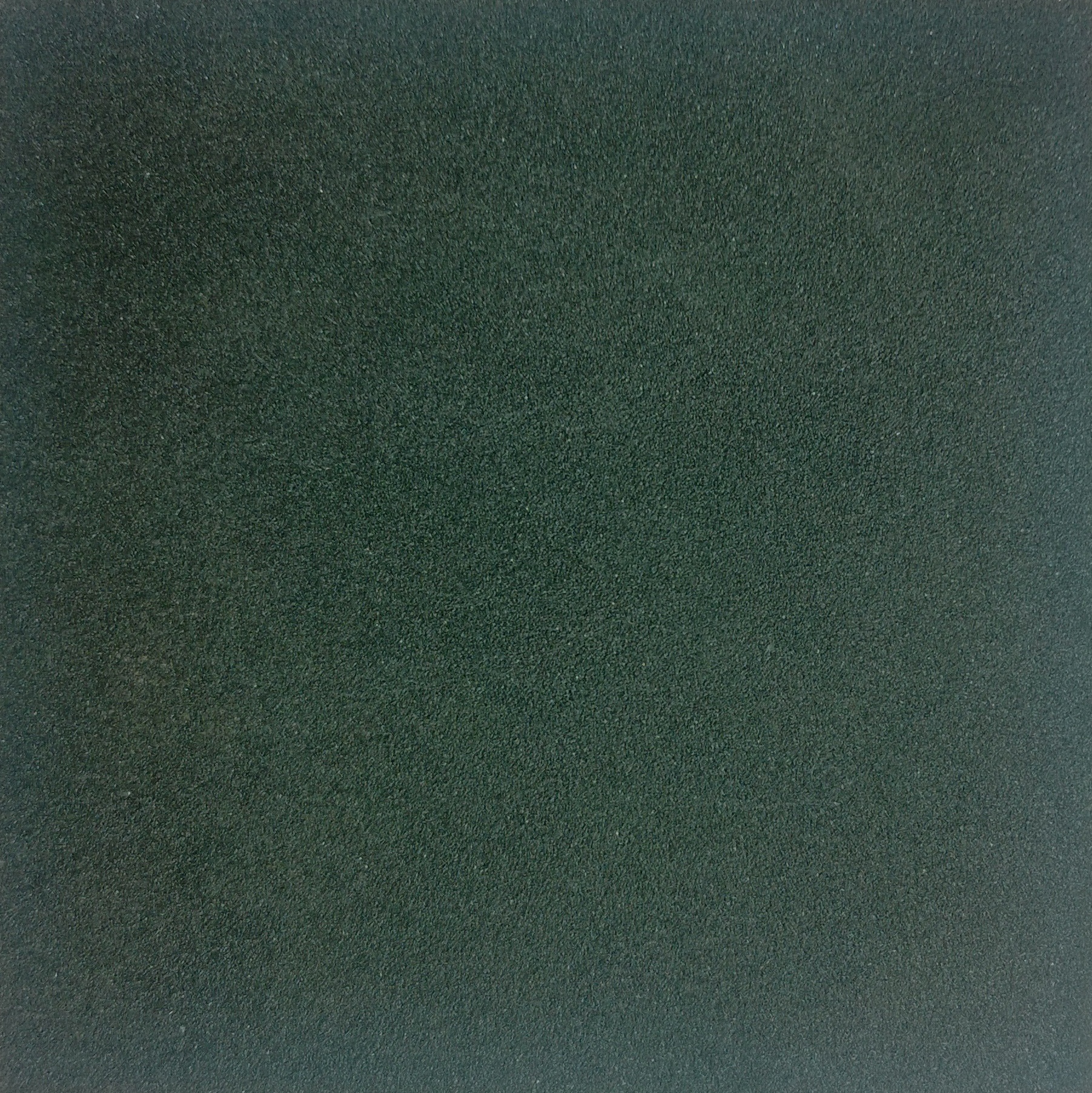

Supplement: Supplementary file 1 — Supplementary Information 2. [file 41598_2023_38929_MOESM1_ESM.zip › 74.jpg]

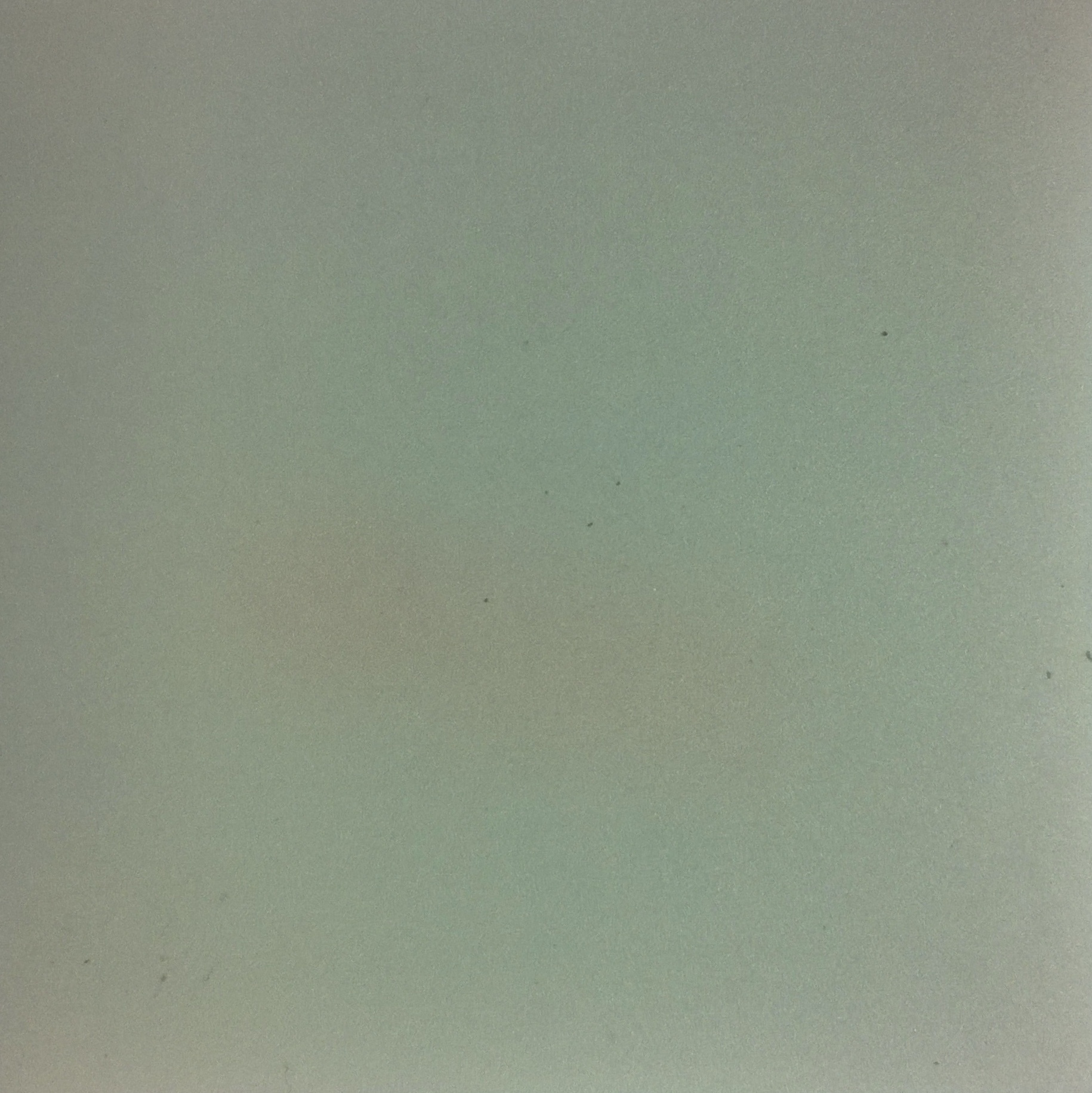

Supplement: Supplementary file 1 — Supplementary Information 2. [file 41598_2023_38929_MOESM1_ESM.zip › 75.jpg]

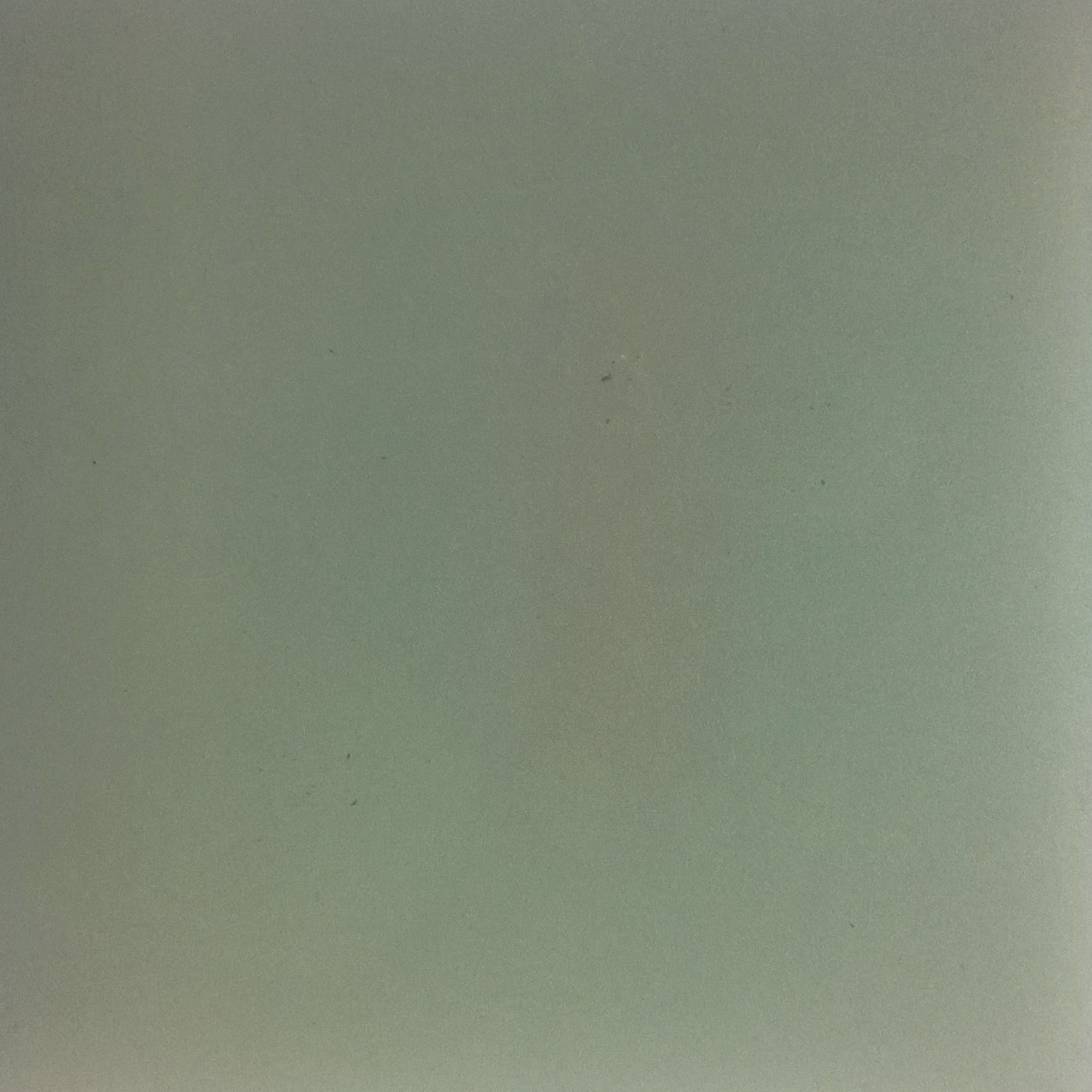

Supplement: Supplementary file 1 — Supplementary Information 2. [file 41598_2023_38929_MOESM1_ESM.zip › 76.jpg]

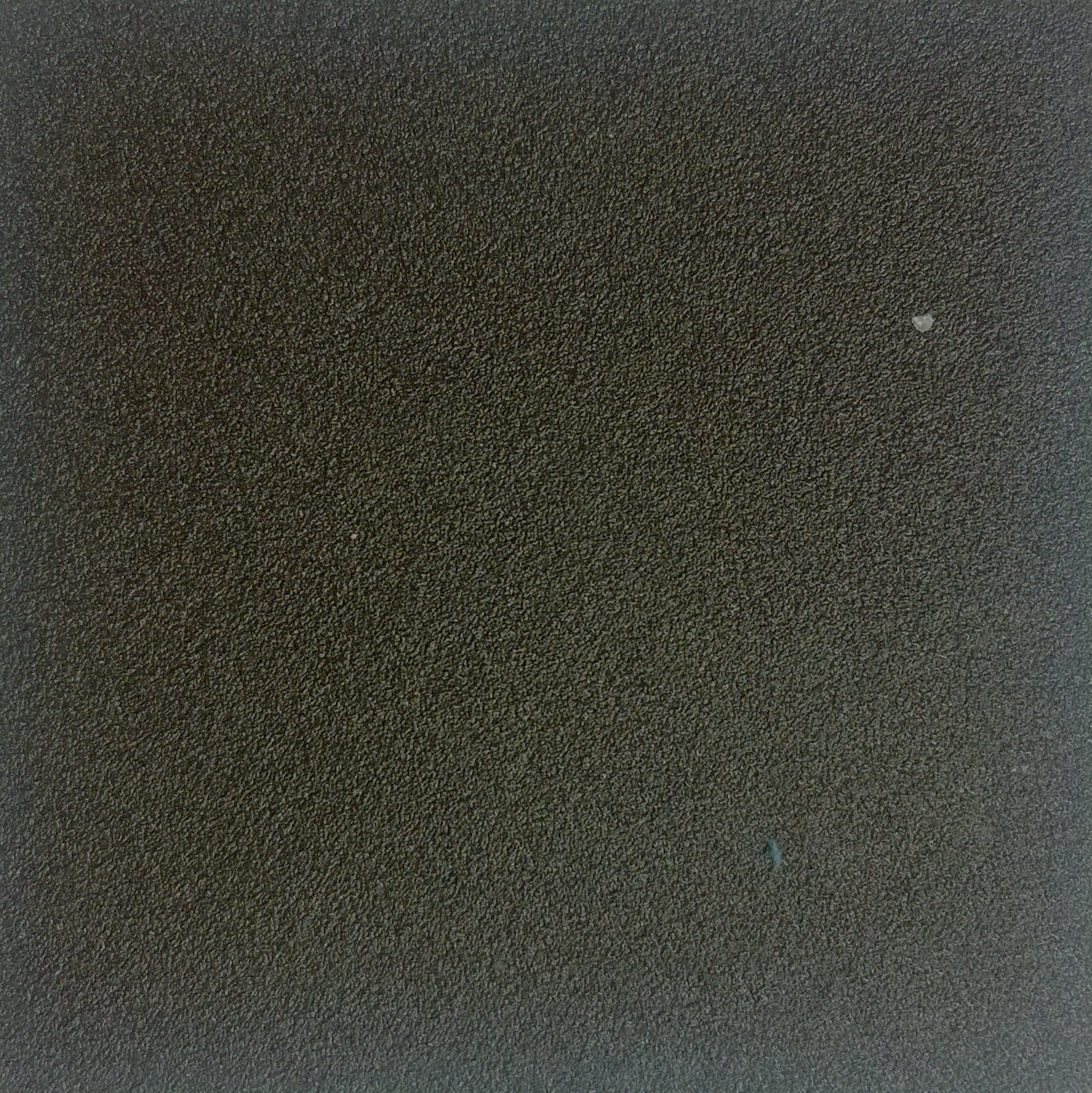

Supplement: Supplementary file 1 — Supplementary Information 2. [file 41598_2023_38929_MOESM1_ESM.zip › 77.jpg]

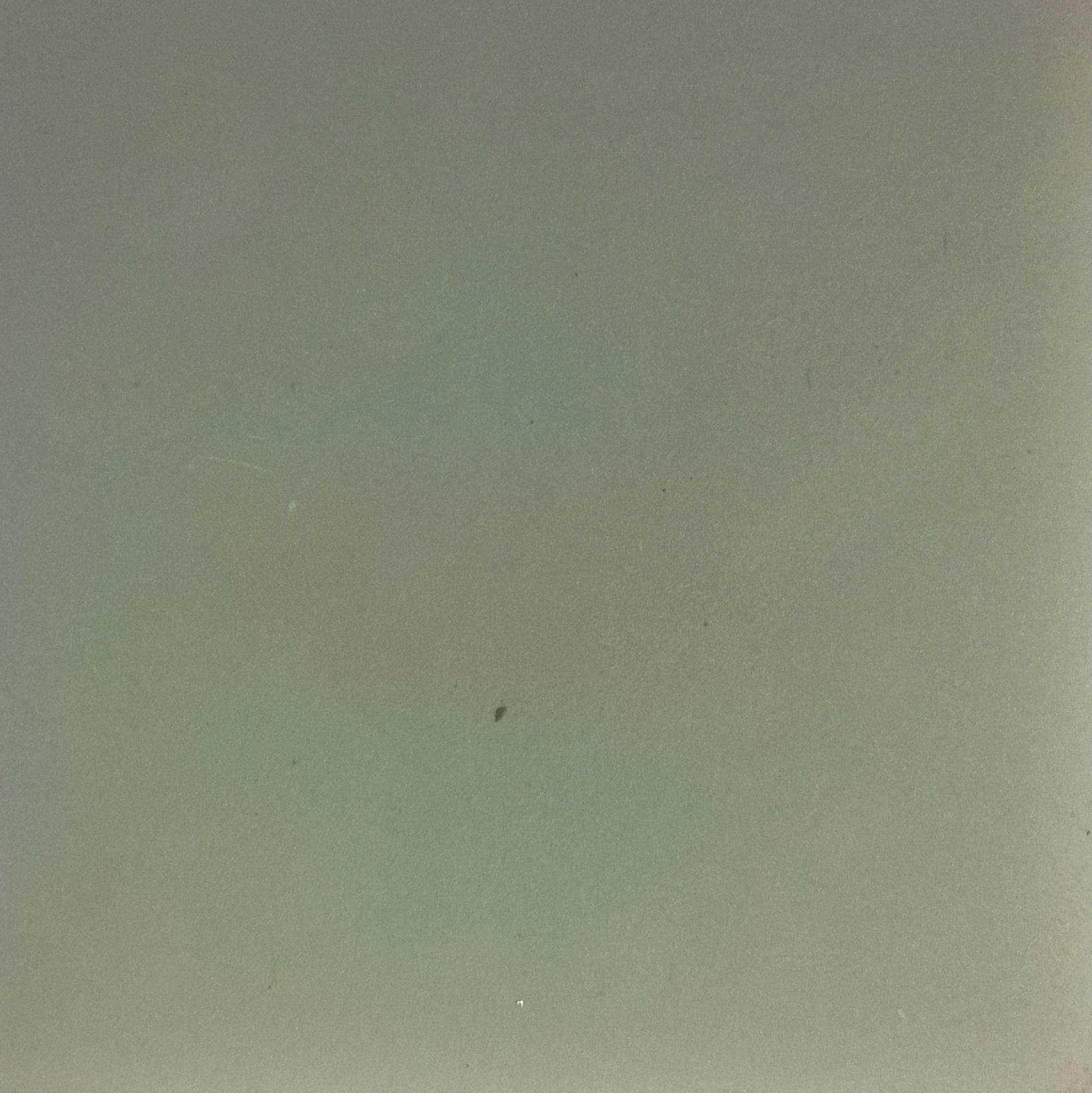

Supplement: Supplementary file 1 — Supplementary Information 2. [file 41598_2023_38929_MOESM1_ESM.zip › 78.jpg]

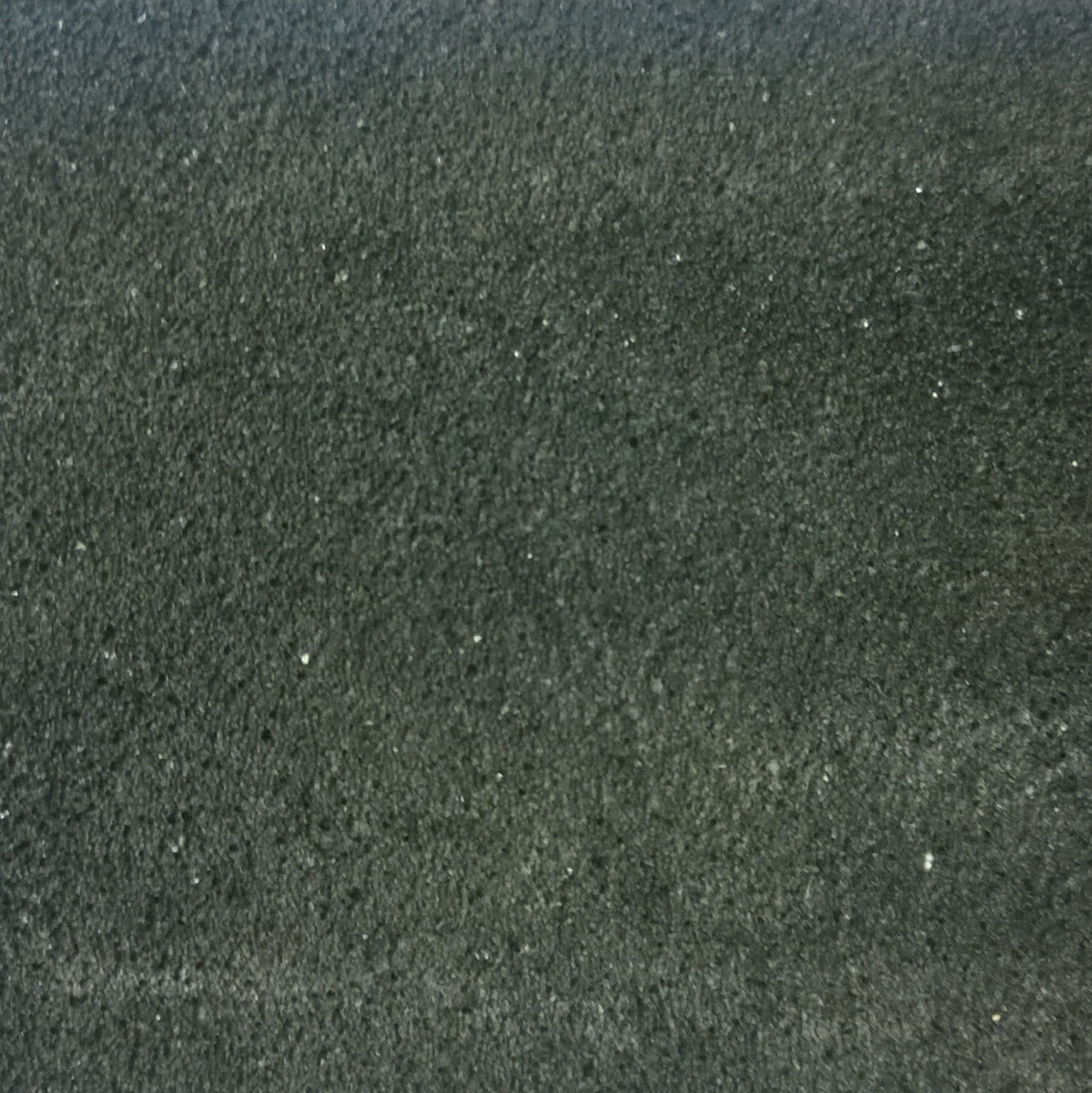

Supplement: Supplementary file 1 — Supplementary Information 2. [file 41598_2023_38929_MOESM1_ESM.zip › 79.jpg]

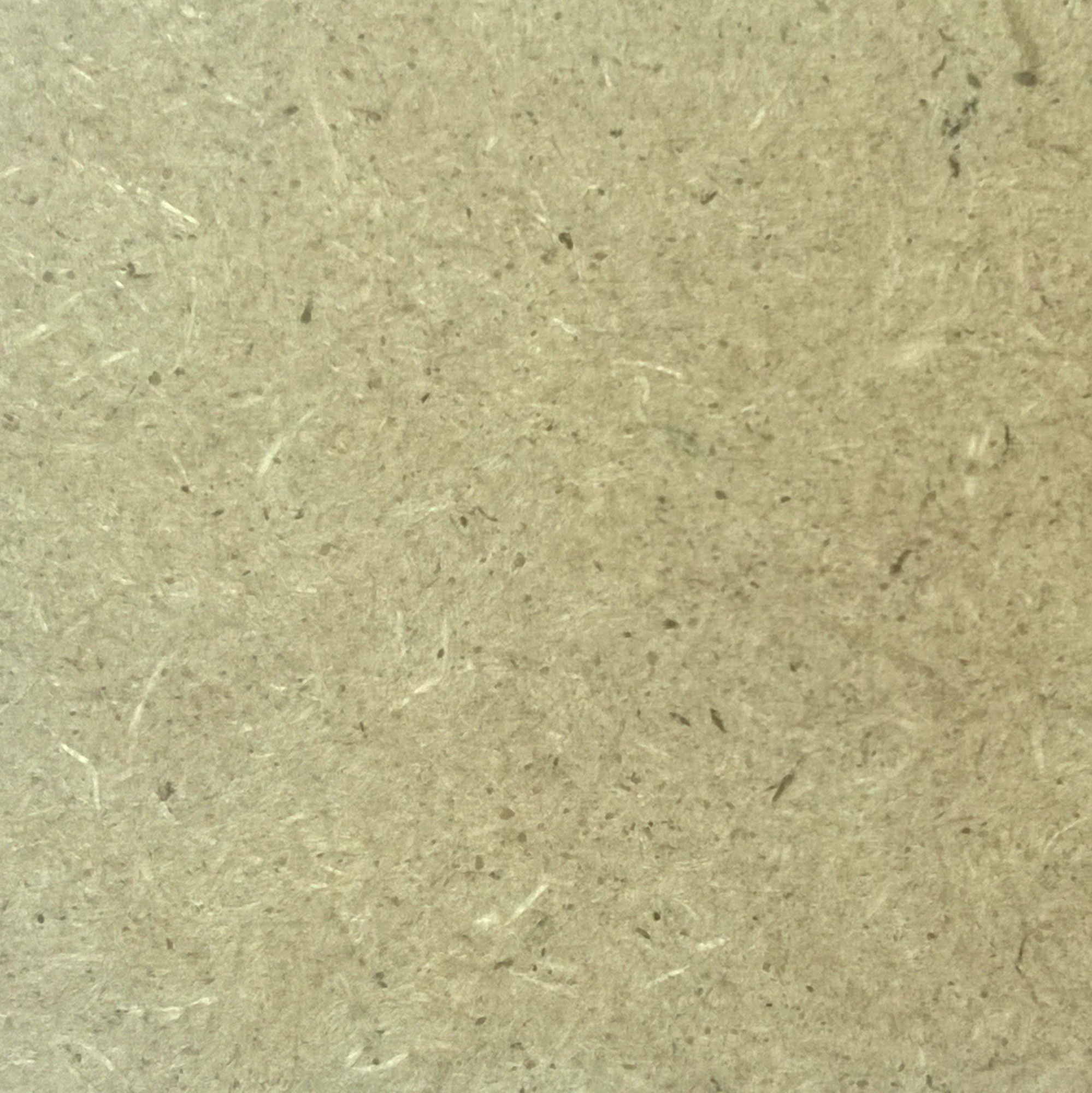

Supplement: Supplementary file 1 — Supplementary Information 2. [file 41598_2023_38929_MOESM1_ESM.zip › 8.jpg]

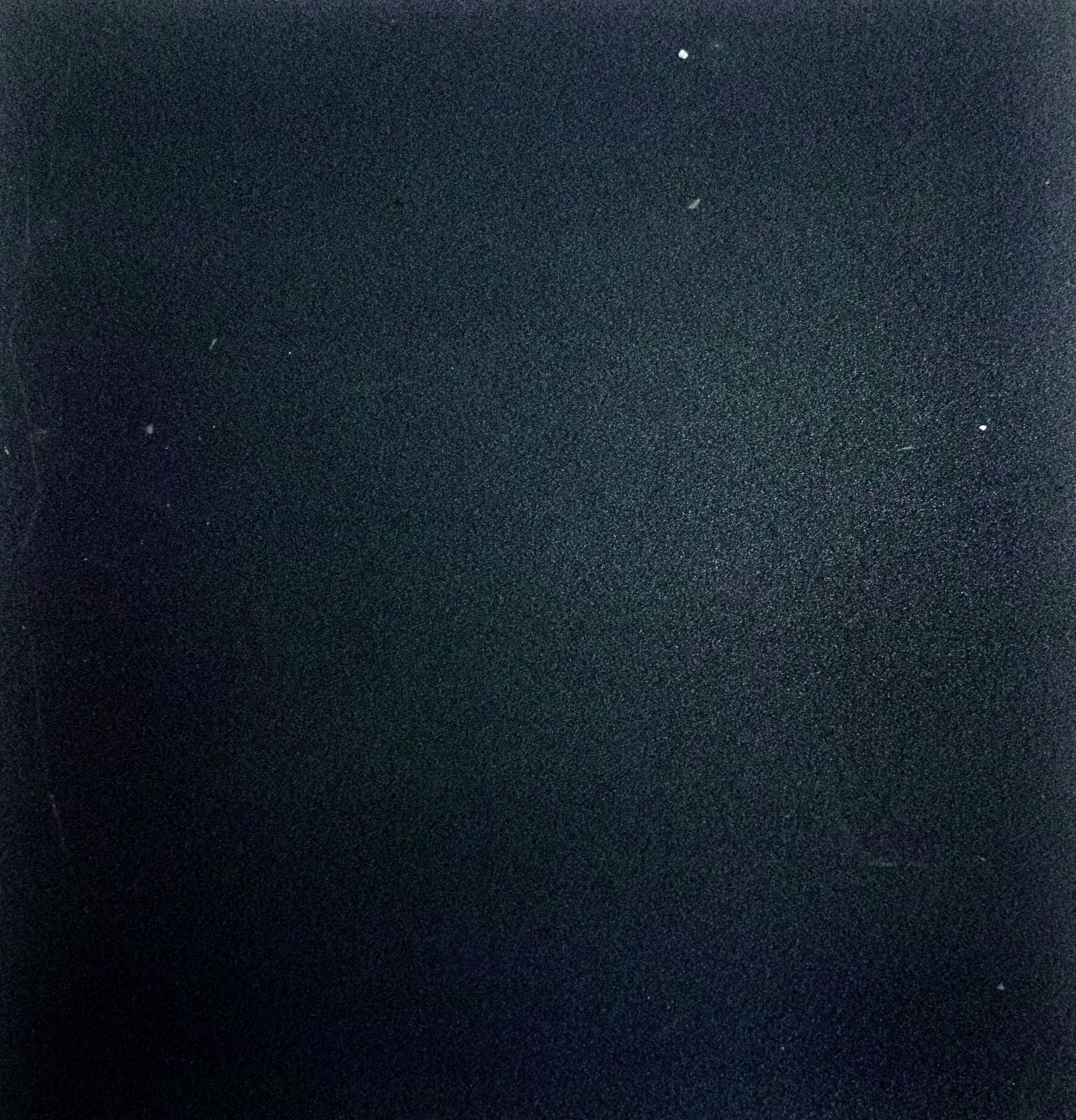

Supplement: Supplementary file 1 — Supplementary Information 2. [file 41598_2023_38929_MOESM1_ESM.zip › 80.JPG]

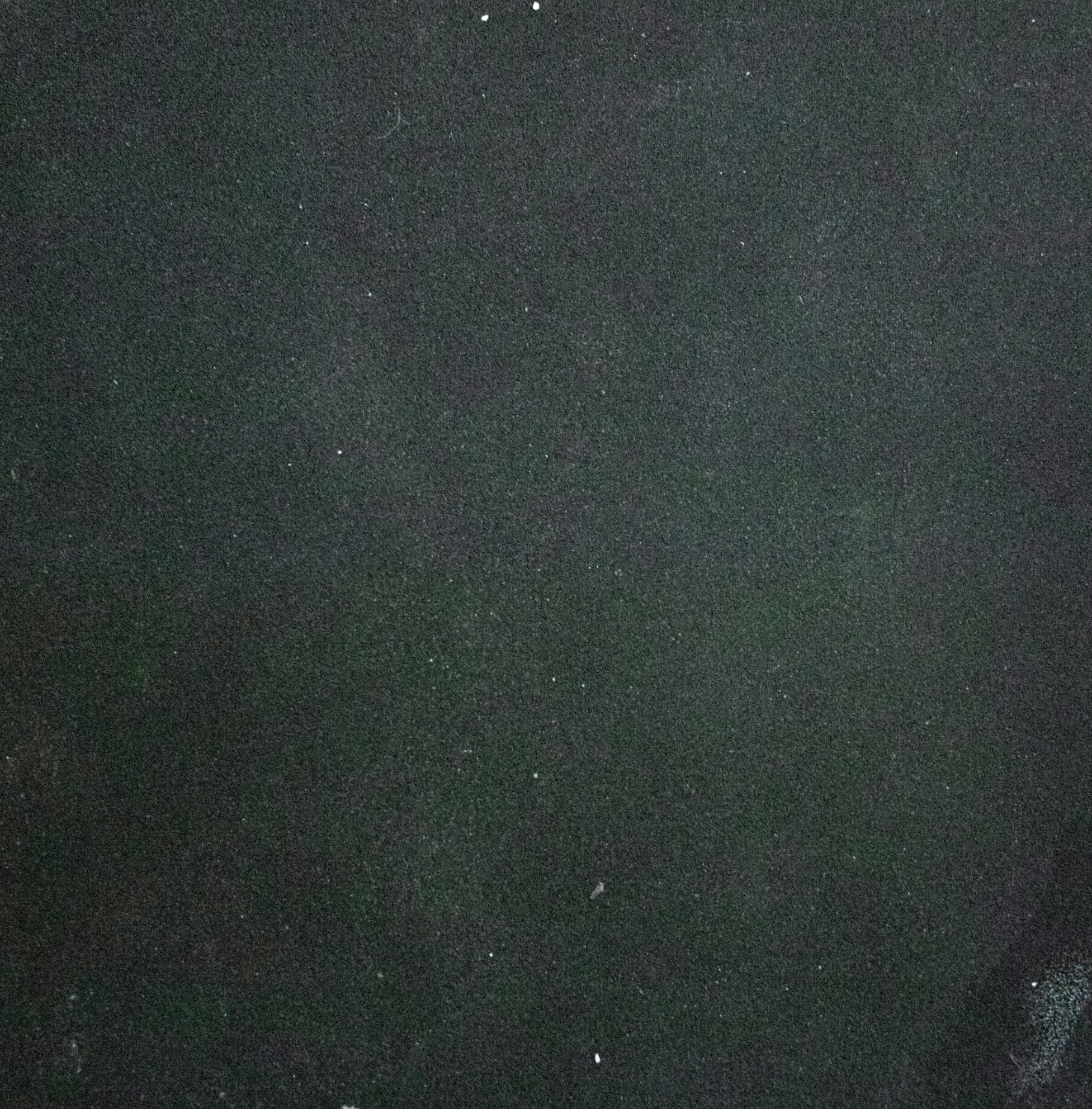

Supplement: Supplementary file 1 — Supplementary Information 2. [file 41598_2023_38929_MOESM1_ESM.zip › 81.JPG]

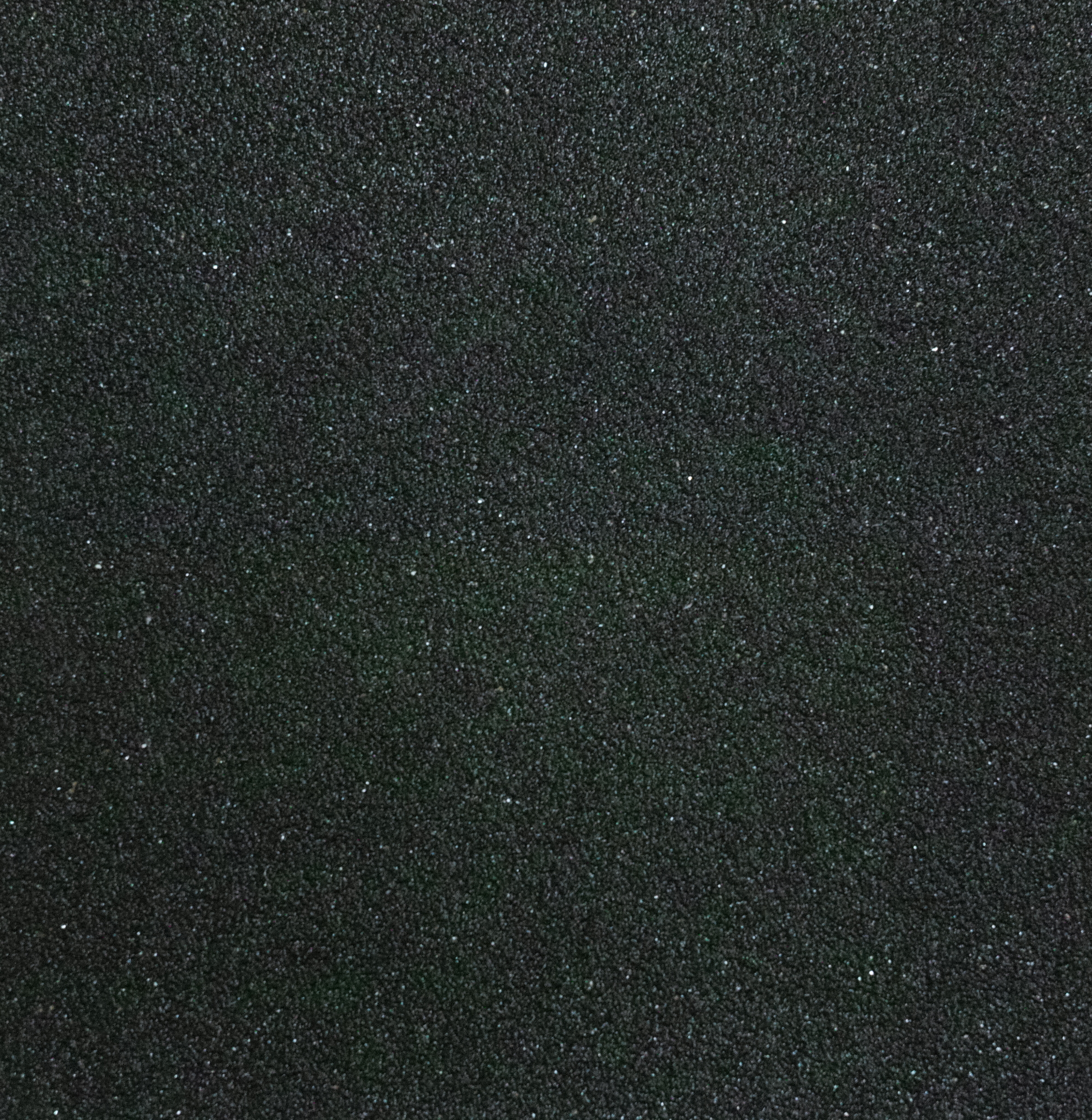

Supplement: Supplementary file 1 — Supplementary Information 2. [file 41598_2023_38929_MOESM1_ESM.zip › 82.JPG]

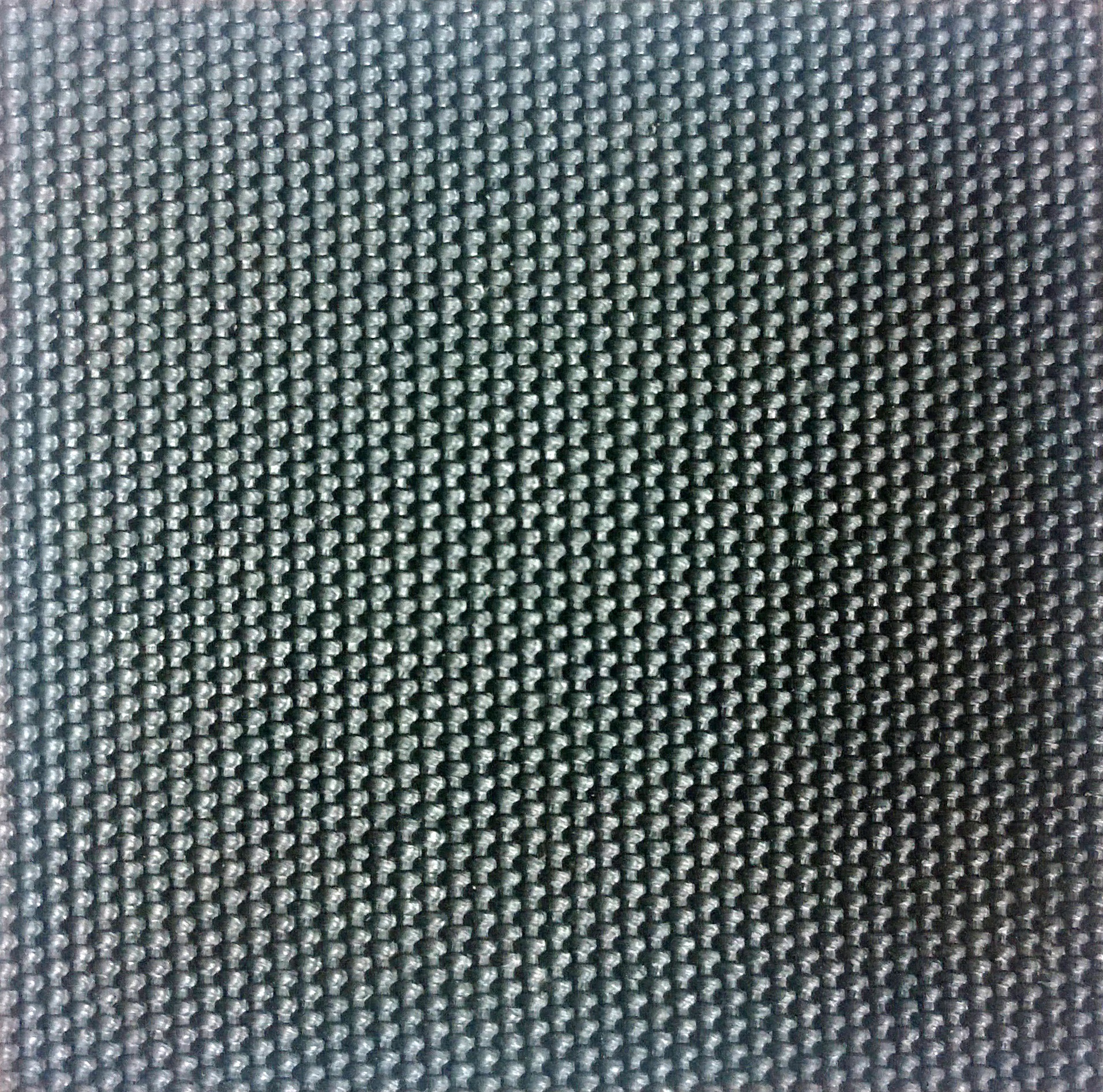

Supplement: Supplementary file 1 — Supplementary Information 2. [file 41598_2023_38929_MOESM1_ESM.zip › 83.jpg]

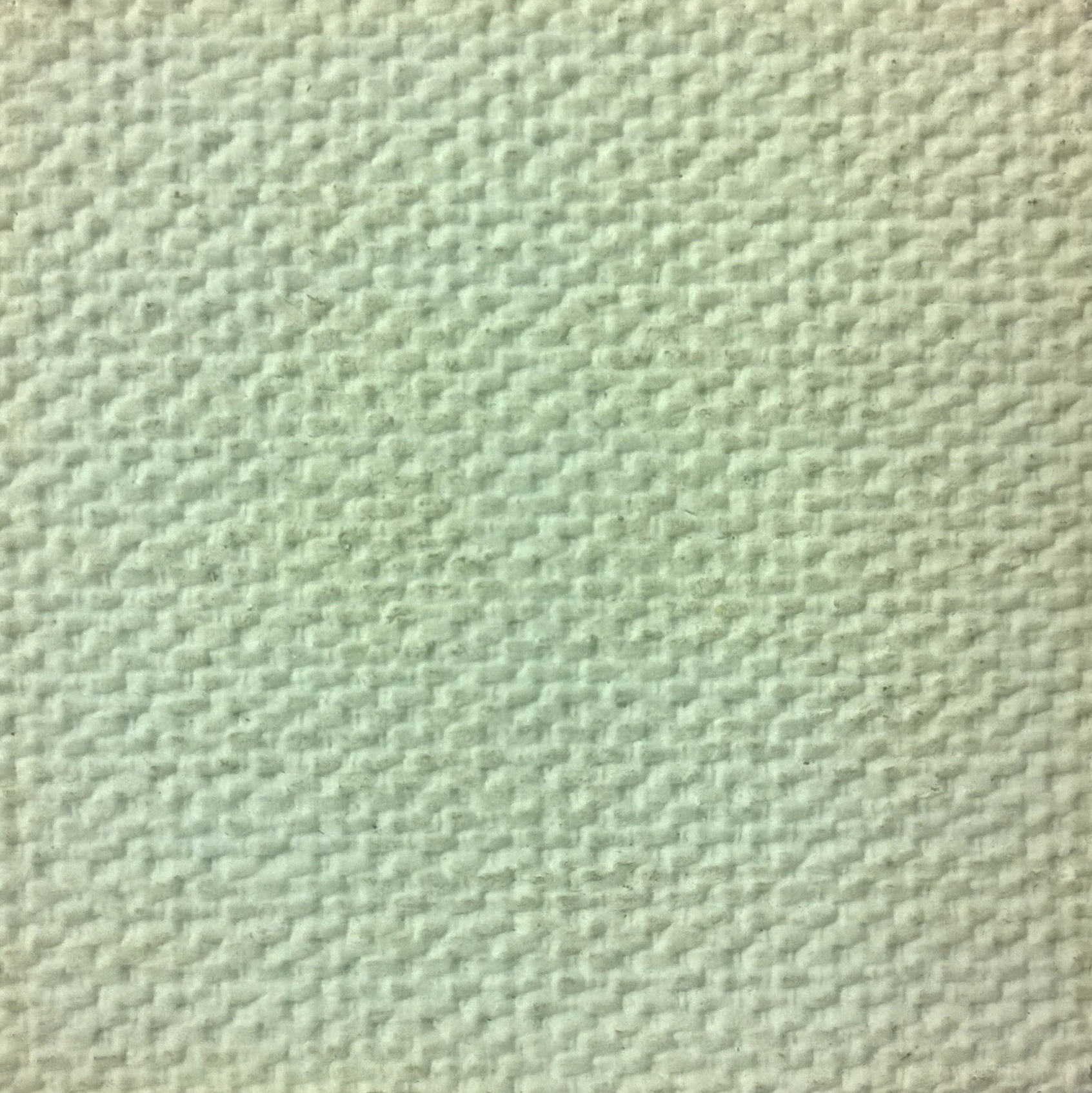

Supplement: Supplementary file 1 — Supplementary Information 2. [file 41598_2023_38929_MOESM1_ESM.zip › 84.jpg]

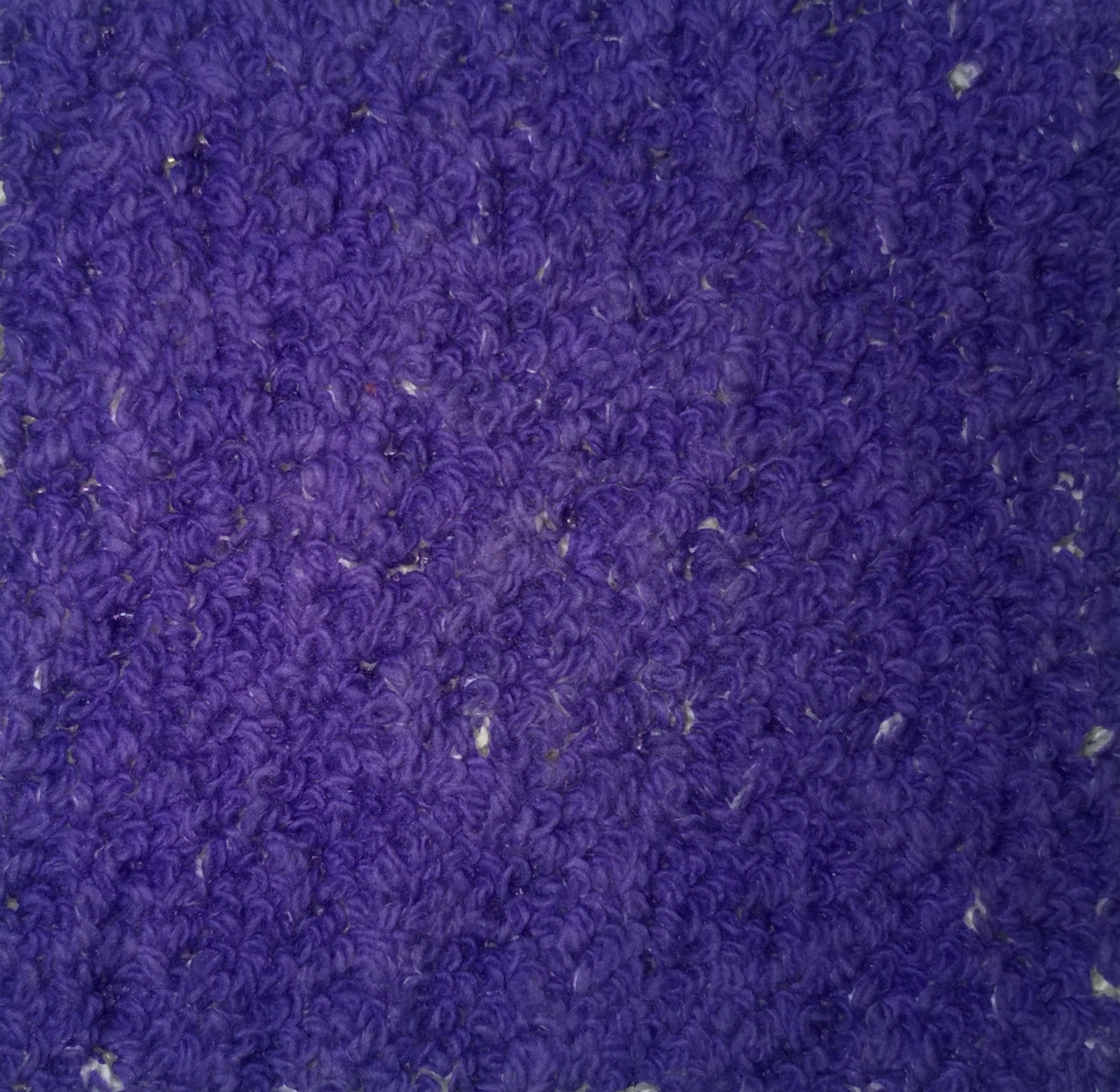

Supplement: Supplementary file 1 — Supplementary Information 2. [file 41598_2023_38929_MOESM1_ESM.zip › 85.jpg]

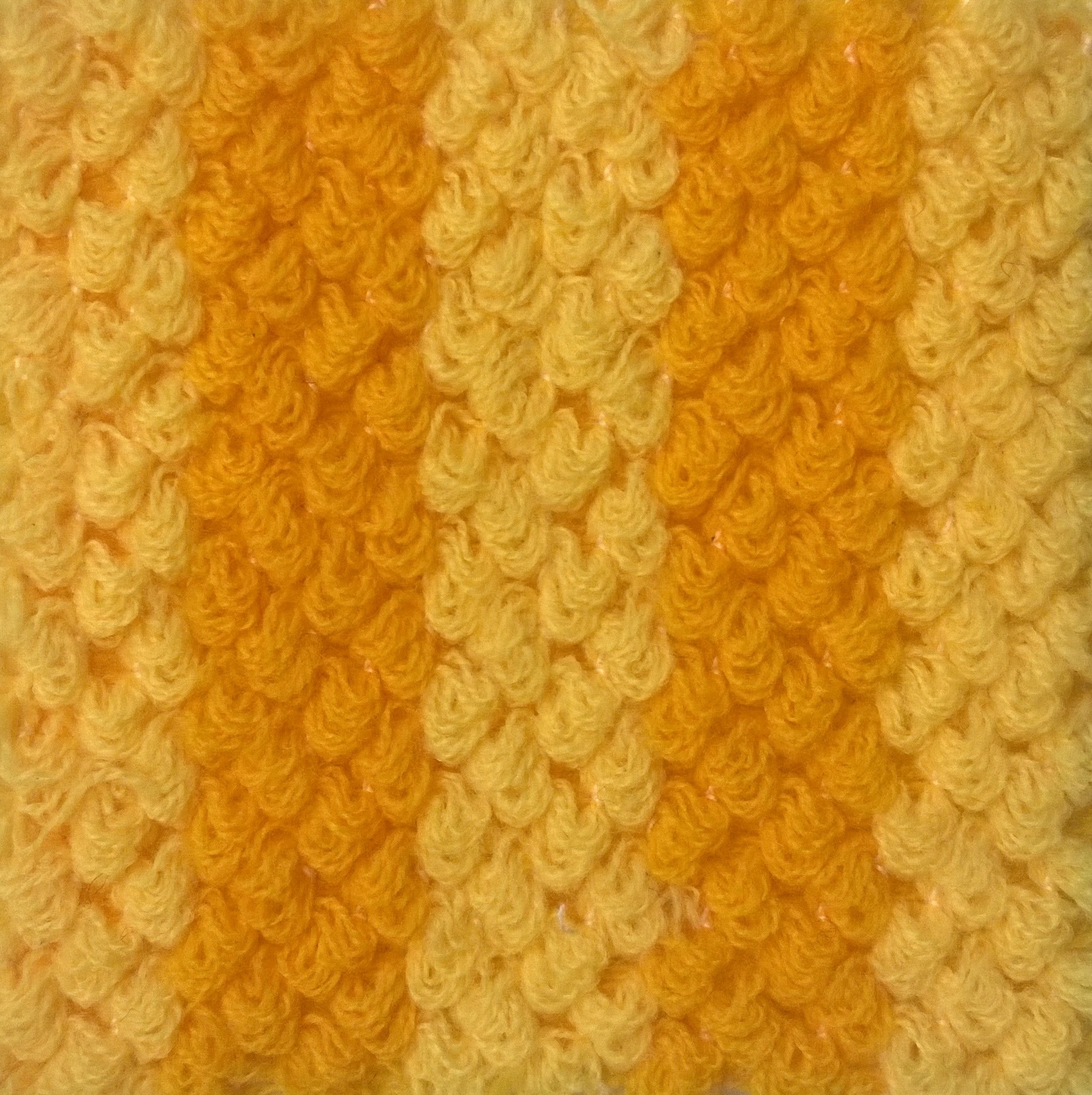

Supplement: Supplementary file 1 — Supplementary Information 2. [file 41598_2023_38929_MOESM1_ESM.zip › 86.jpg]

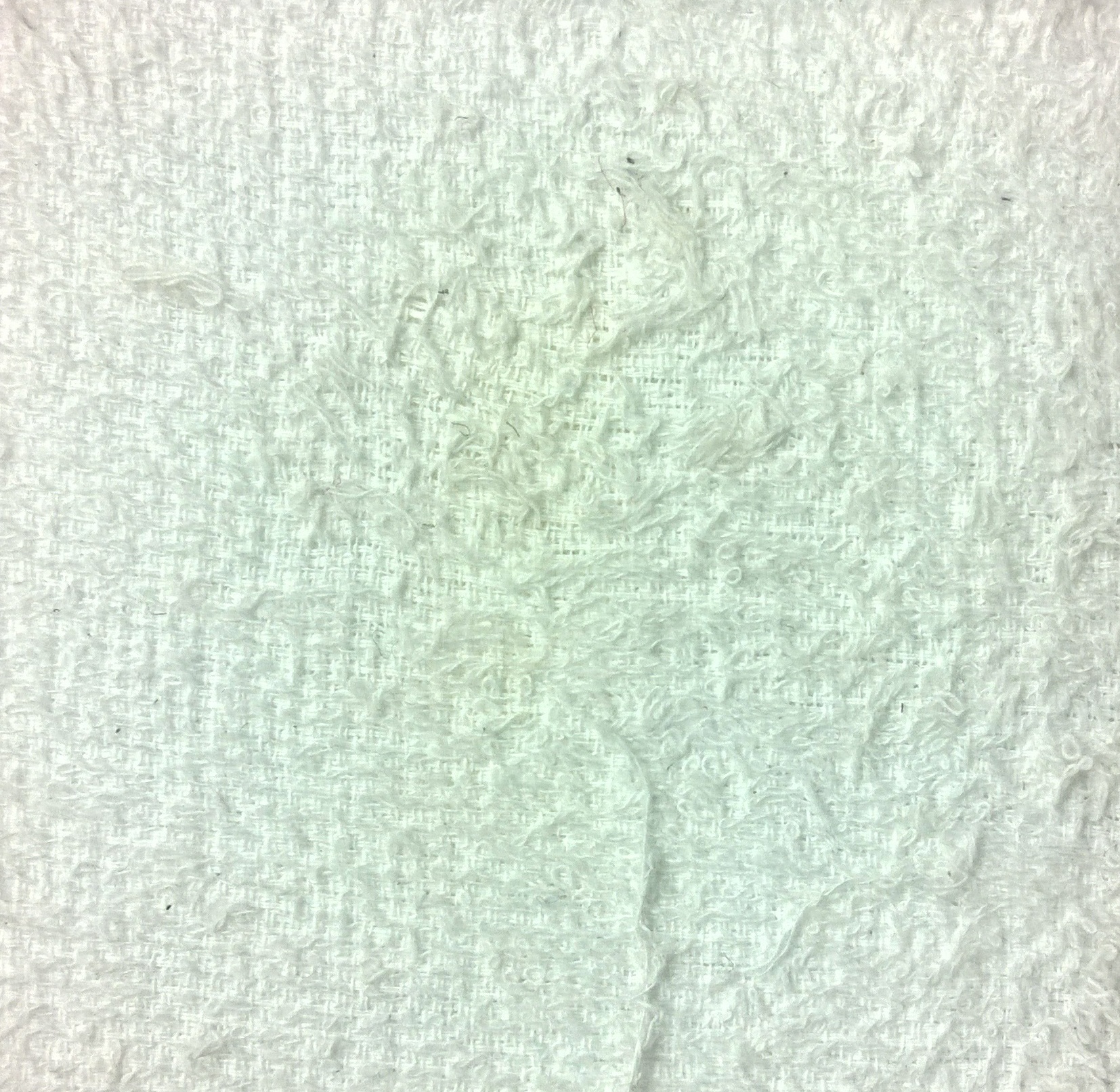

Supplement: Supplementary file 1 — Supplementary Information 2. [file 41598_2023_38929_MOESM1_ESM.zip › 87.jpg]

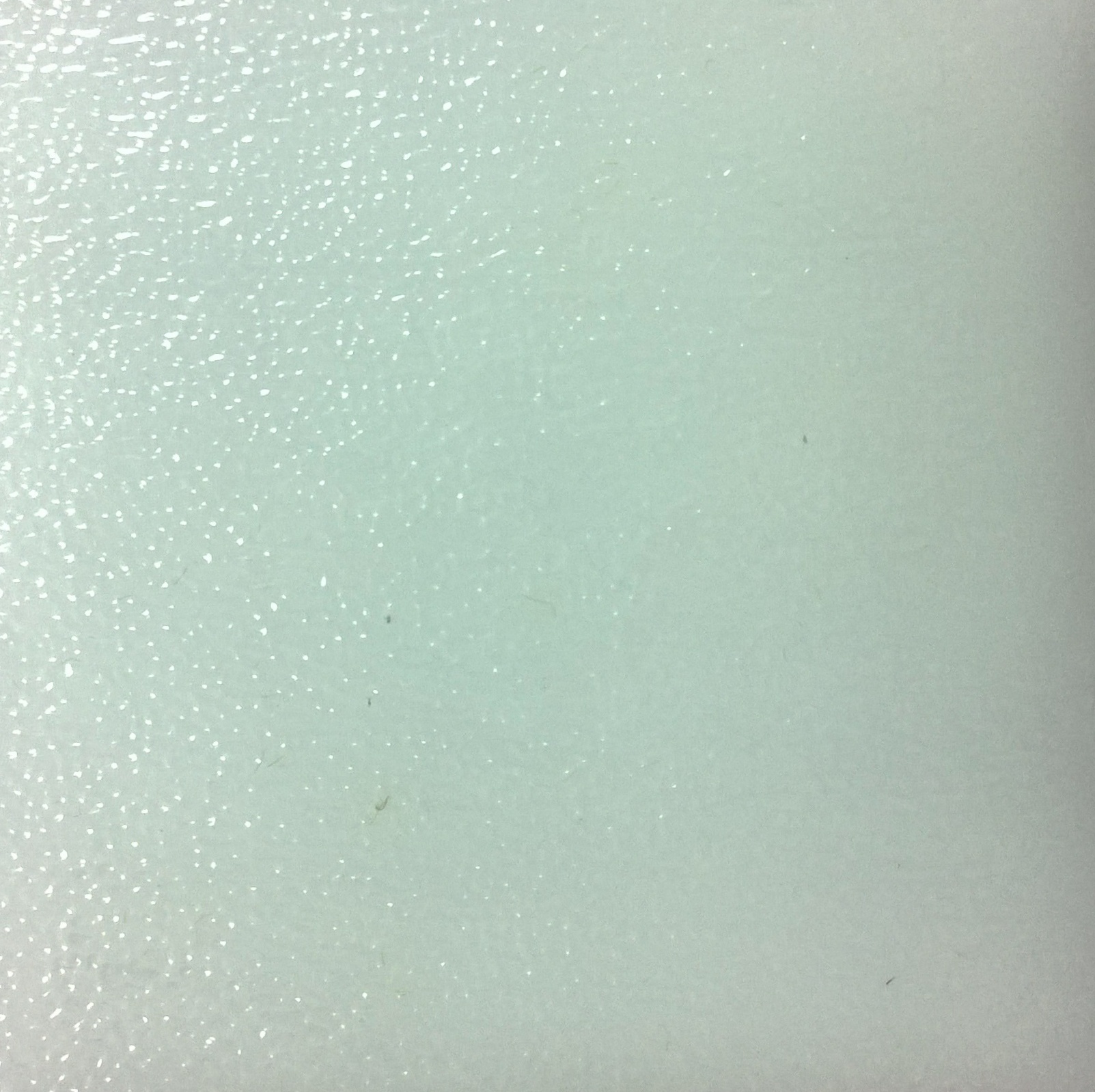

Supplement: Supplementary file 1 — Supplementary Information 2. [file 41598_2023_38929_MOESM1_ESM.zip › 88.jpg]

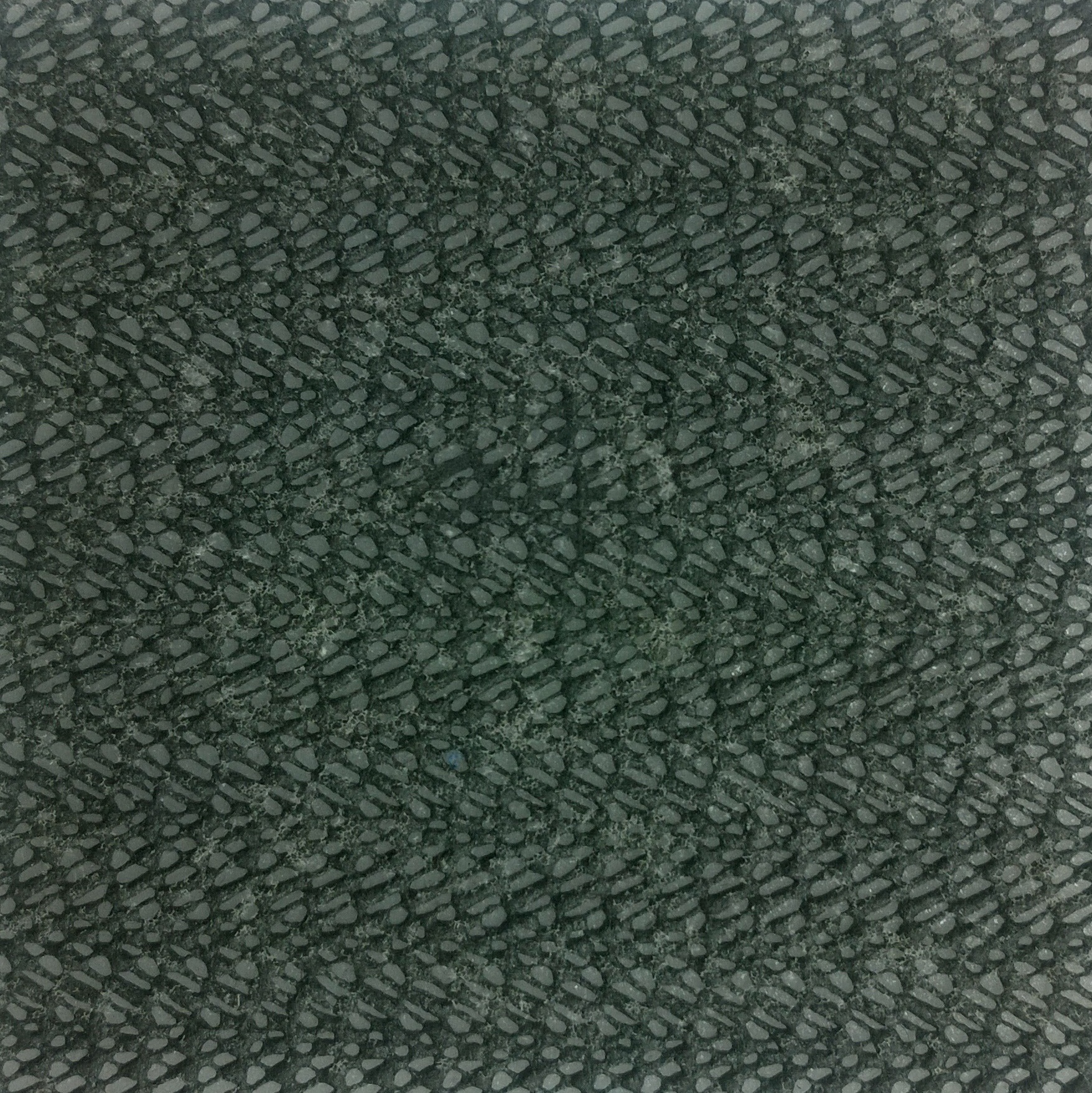

Supplement: Supplementary file 1 — Supplementary Information 2. [file 41598_2023_38929_MOESM1_ESM.zip › 89.jpg]

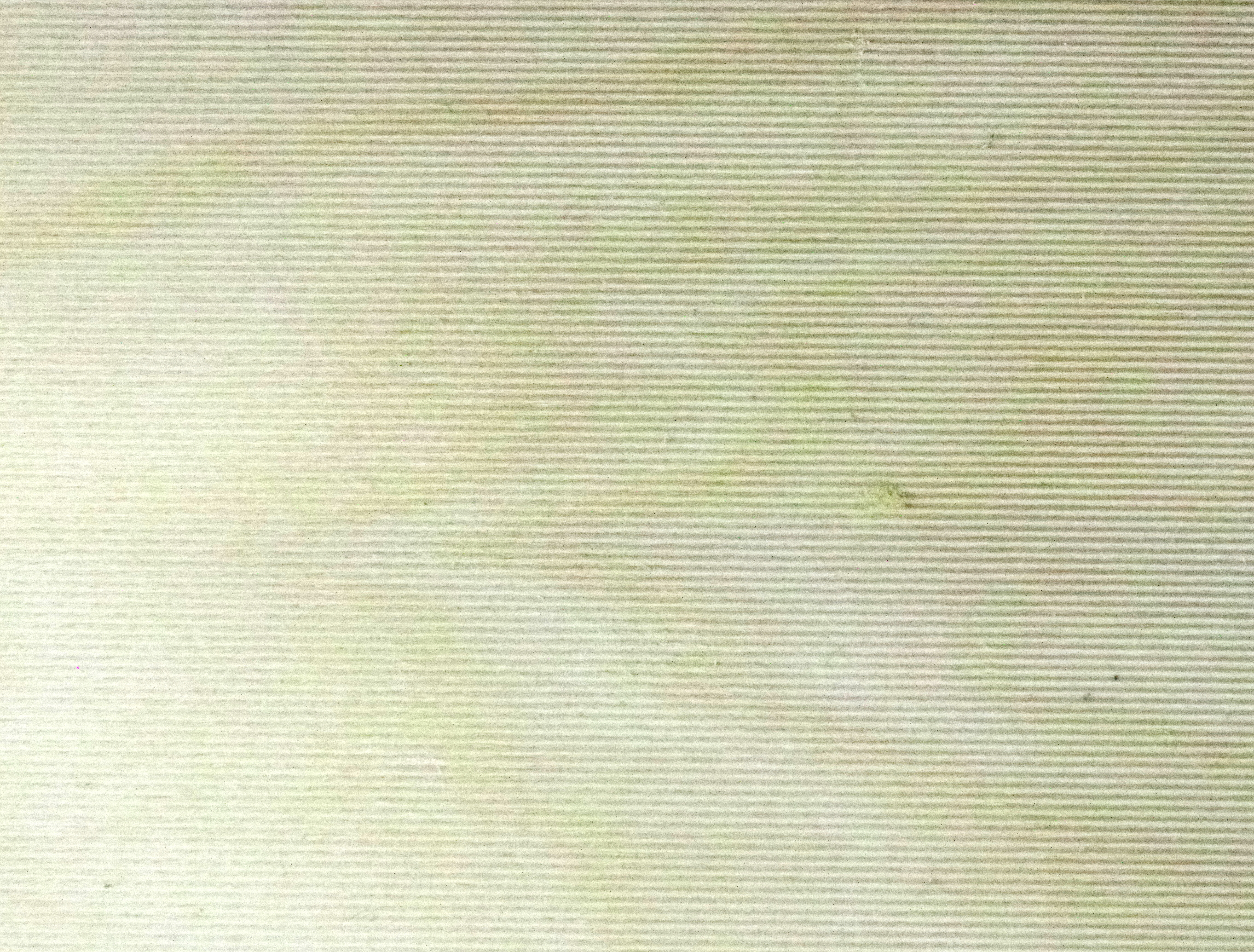

Supplement: Supplementary file 1 — Supplementary Information 2. [file 41598_2023_38929_MOESM1_ESM.zip › 9.JPG]

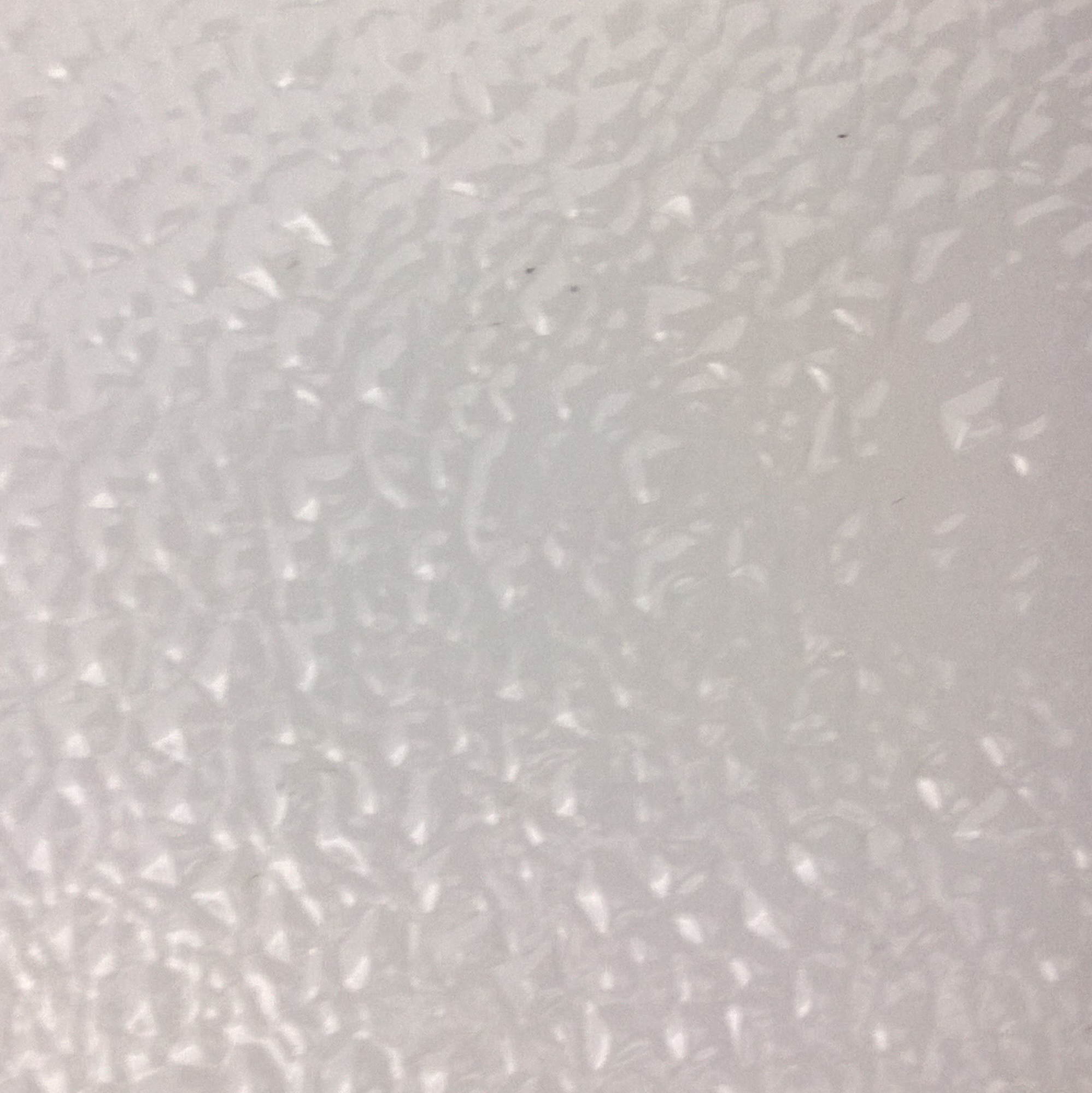

Supplement: Supplementary file 1 — Supplementary Information 2. [file 41598_2023_38929_MOESM1_ESM.zip › 90.jpg]

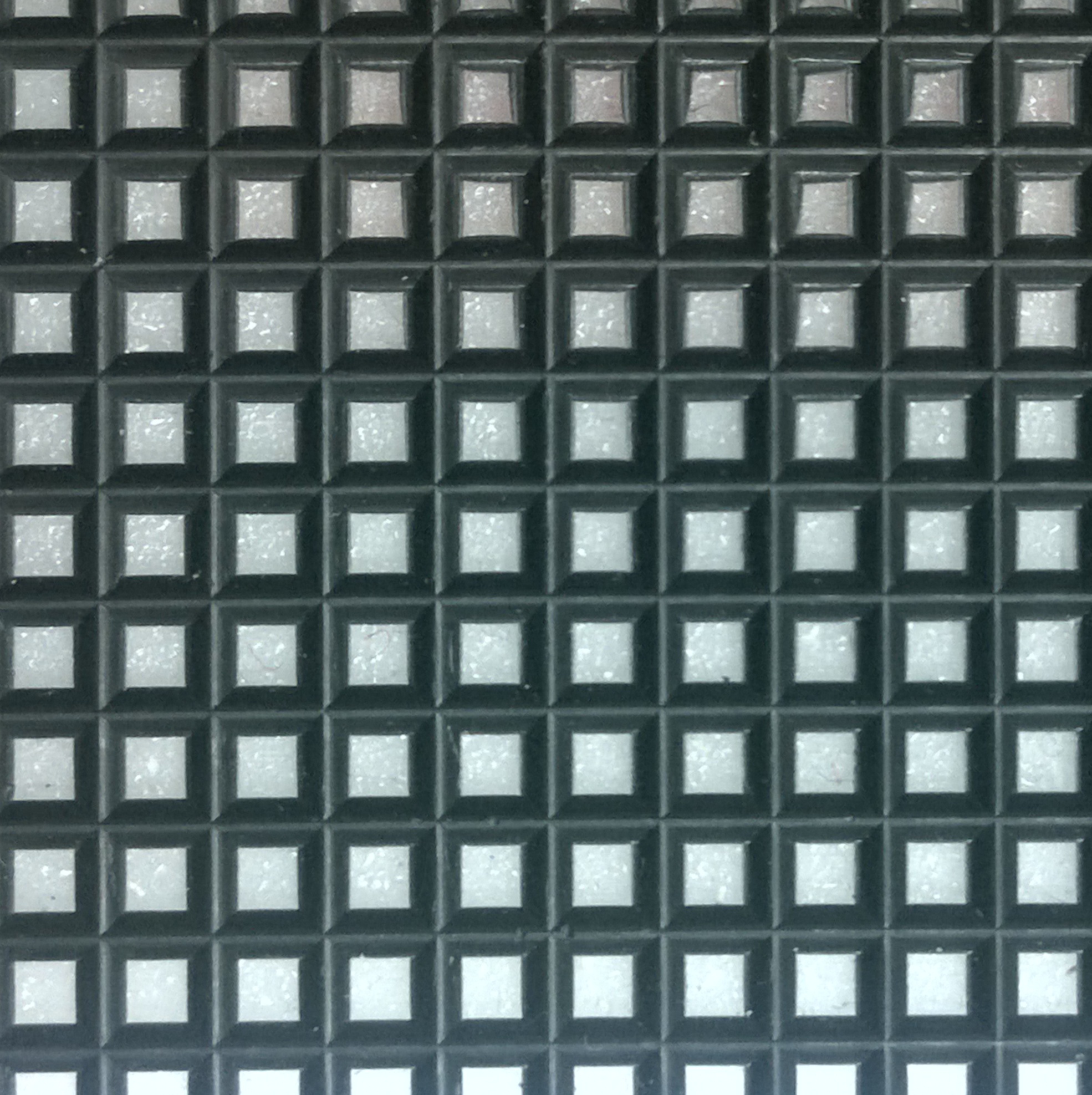

Supplement: Supplementary file 1 — Supplementary Information 2. [file 41598_2023_38929_MOESM1_ESM.zip › 91.jpg]

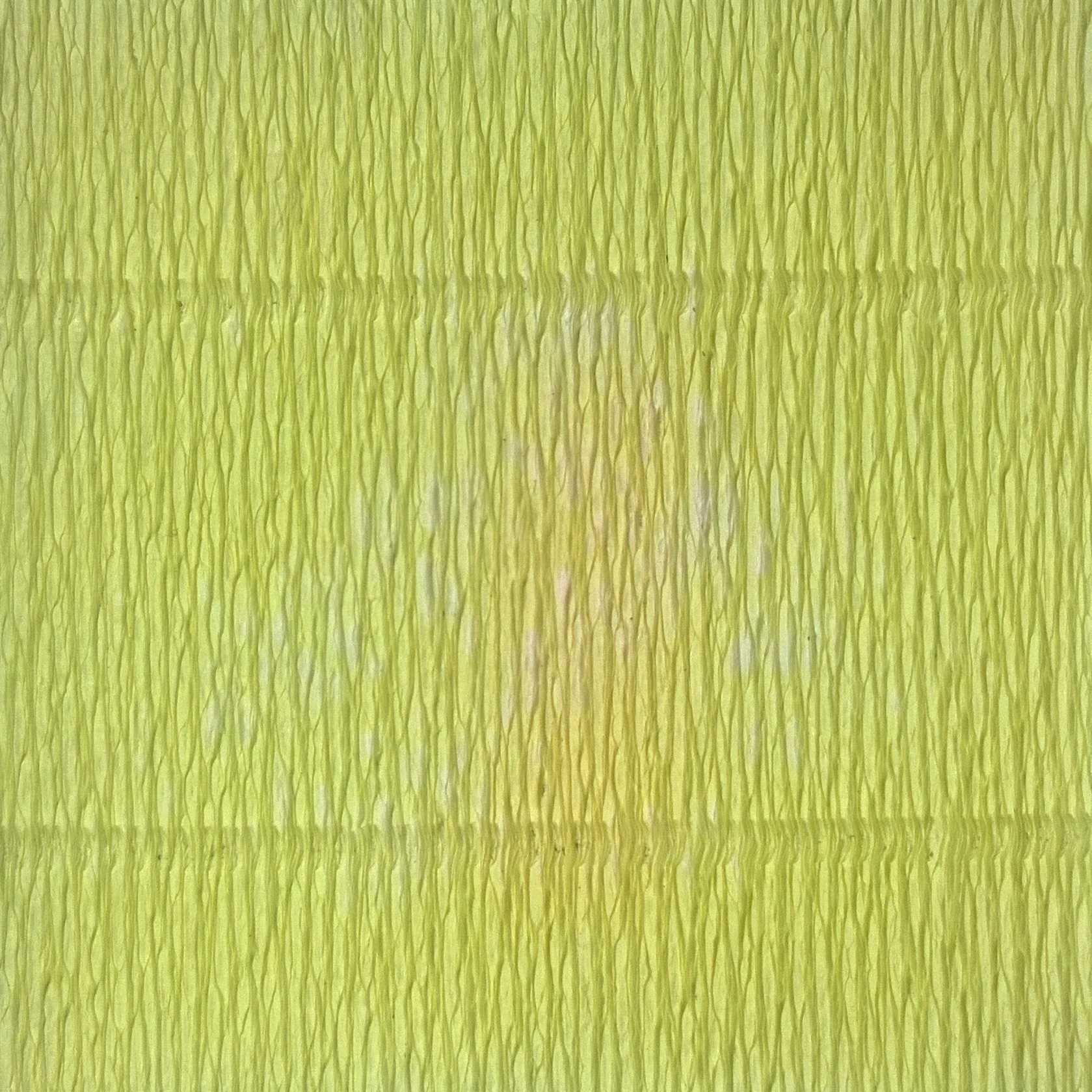

Supplement: Supplementary file 1 — Supplementary Information 2. [file 41598_2023_38929_MOESM1_ESM.zip › 92.jpg]

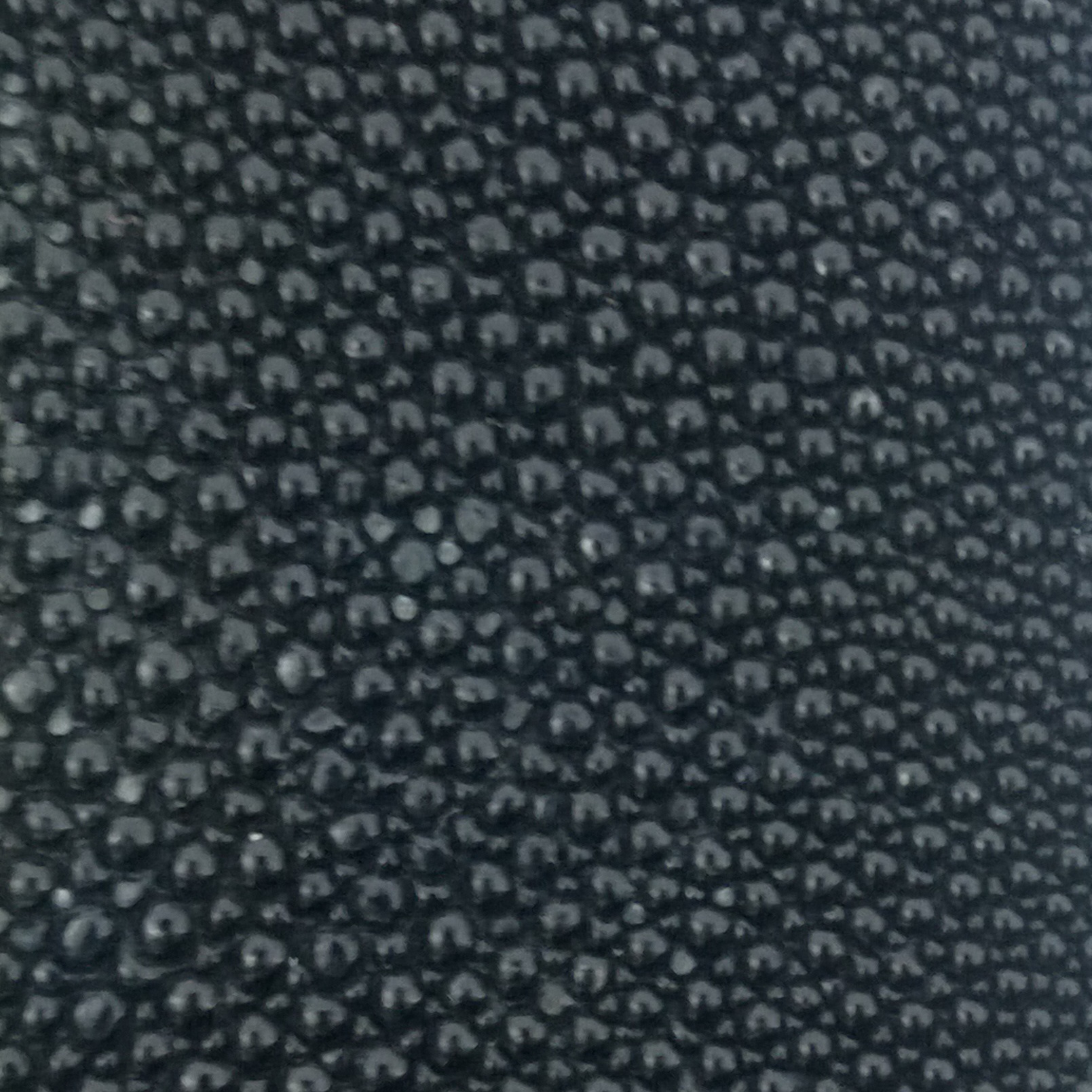

Supplement: Supplementary file 1 — Supplementary Information 2. [file 41598_2023_38929_MOESM1_ESM.zip › 93.jpg]

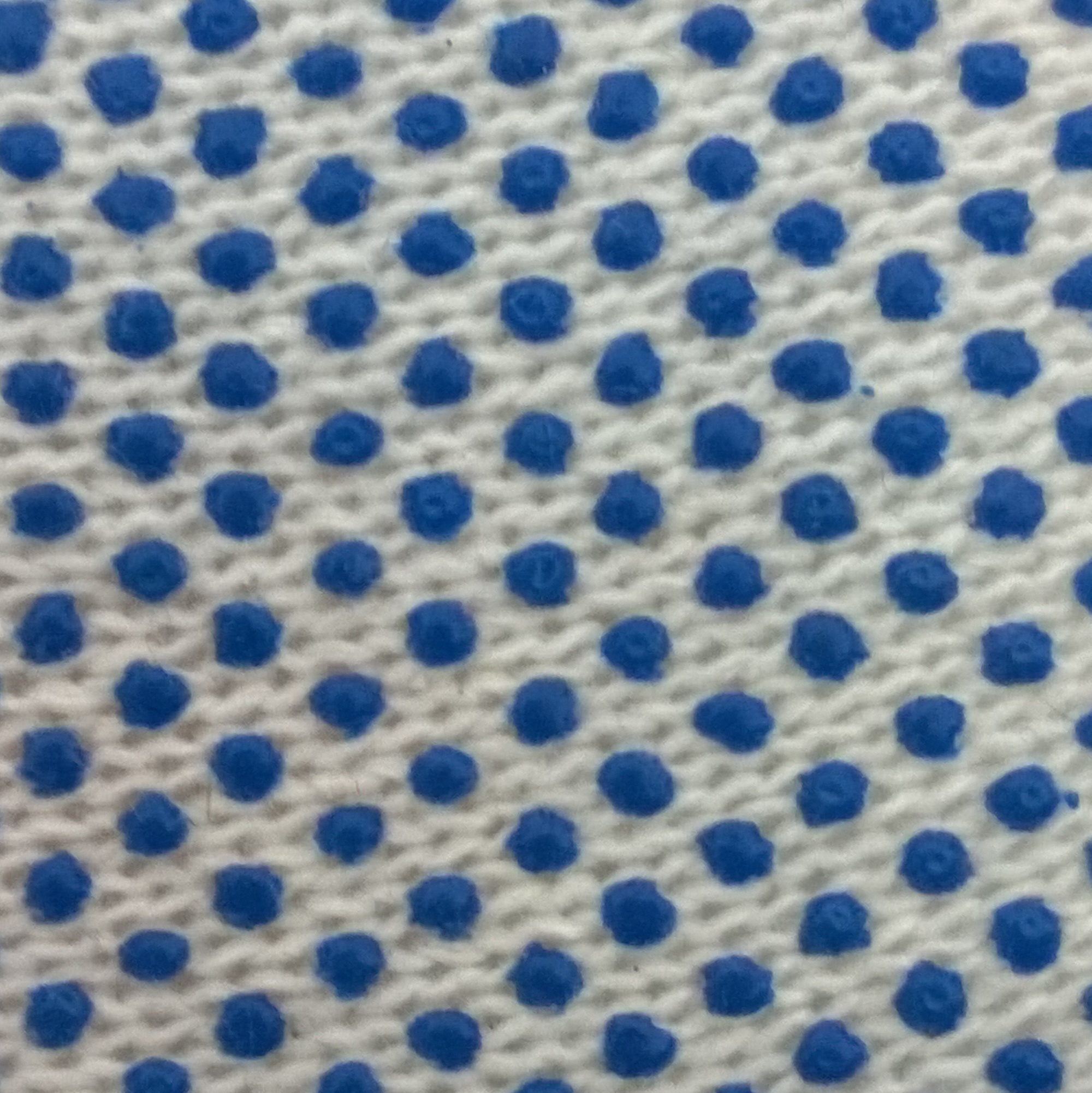

Supplement: Supplementary file 1 — Supplementary Information 2. [file 41598_2023_38929_MOESM1_ESM.zip › 94.jpg]

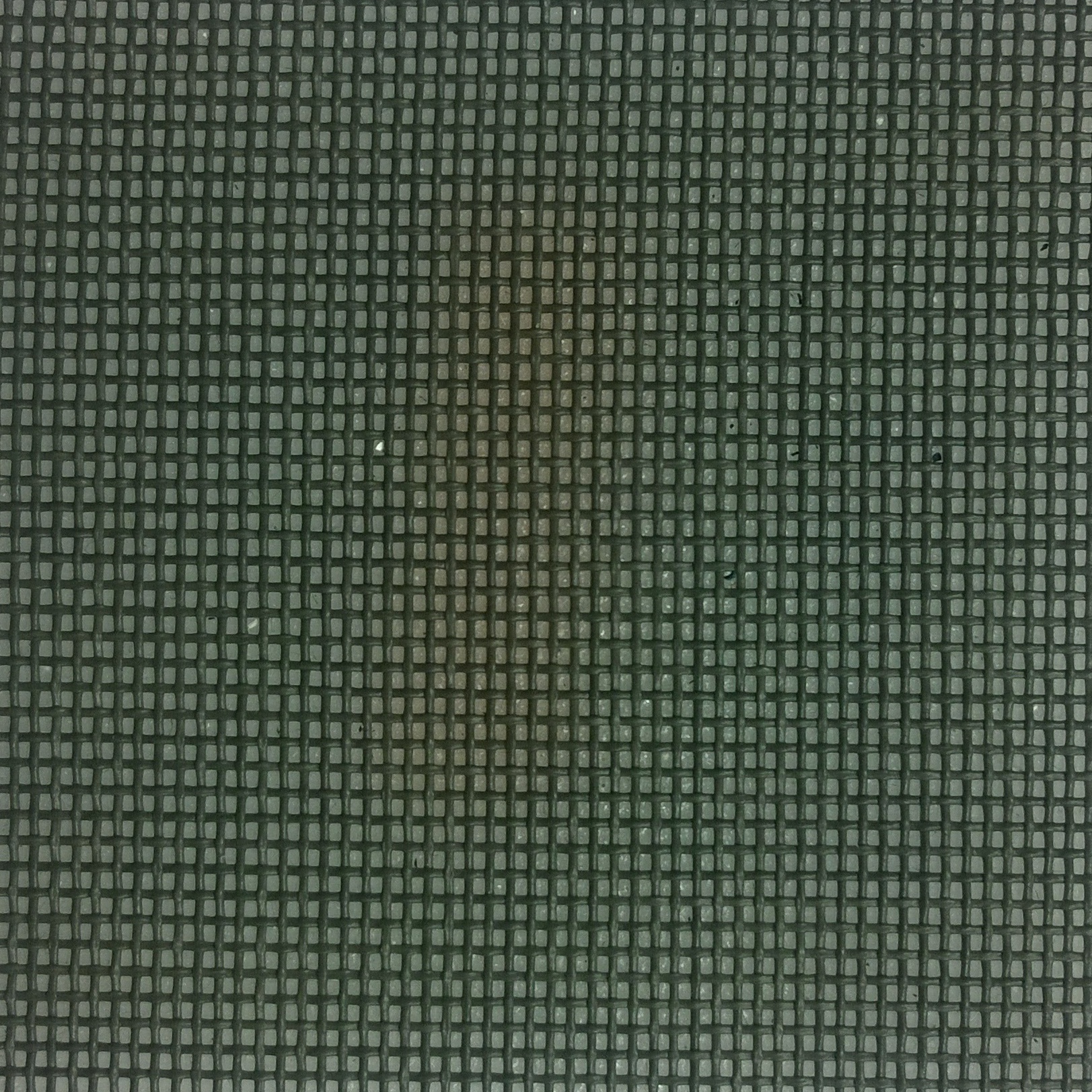

Supplement: Supplementary file 1 — Supplementary Information 2. [file 41598_2023_38929_MOESM1_ESM.zip › 95.jpg]

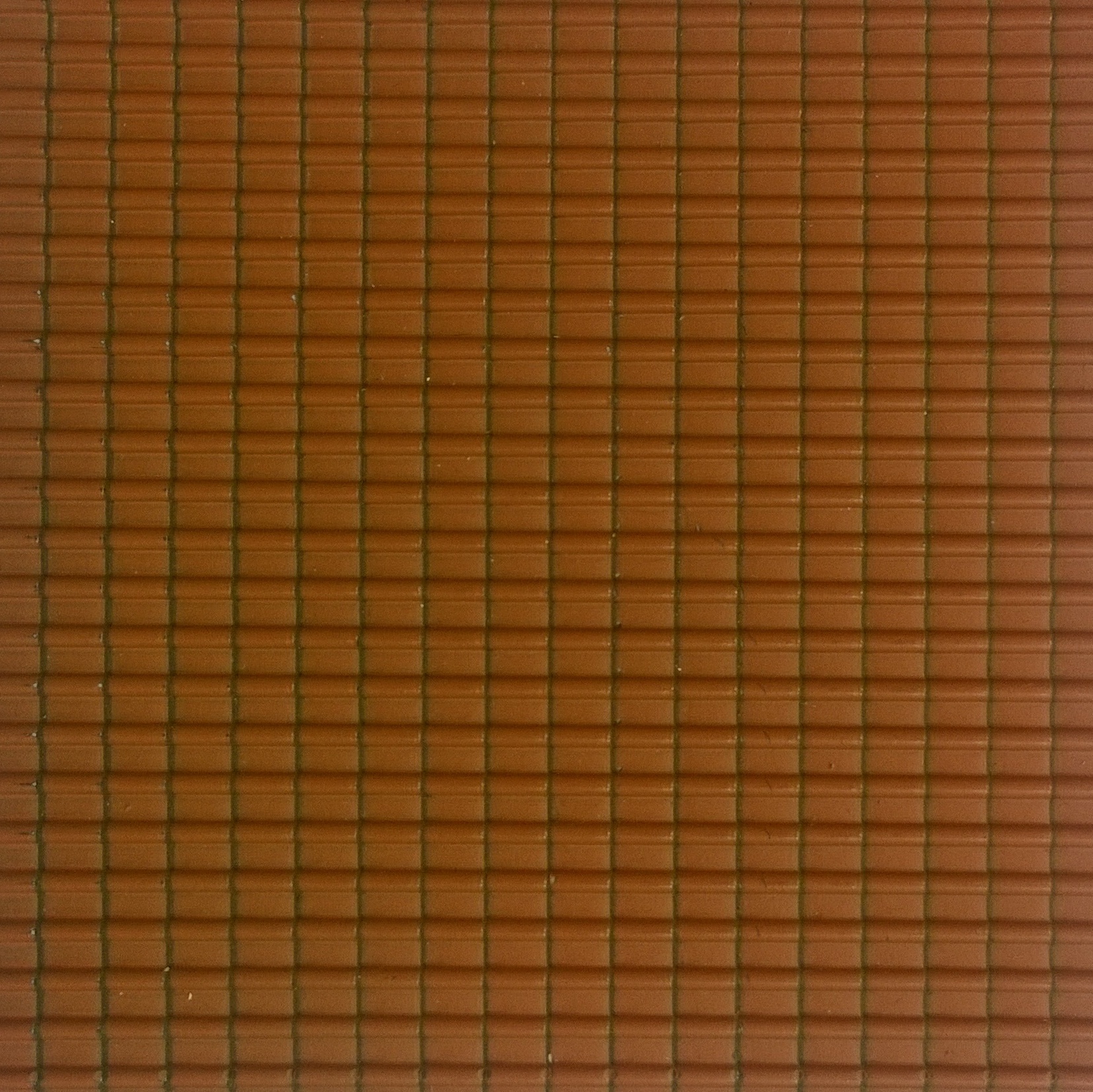

Supplement: Supplementary file 1 — Supplementary Information 2. [file 41598_2023_38929_MOESM1_ESM.zip › 96.jpg]

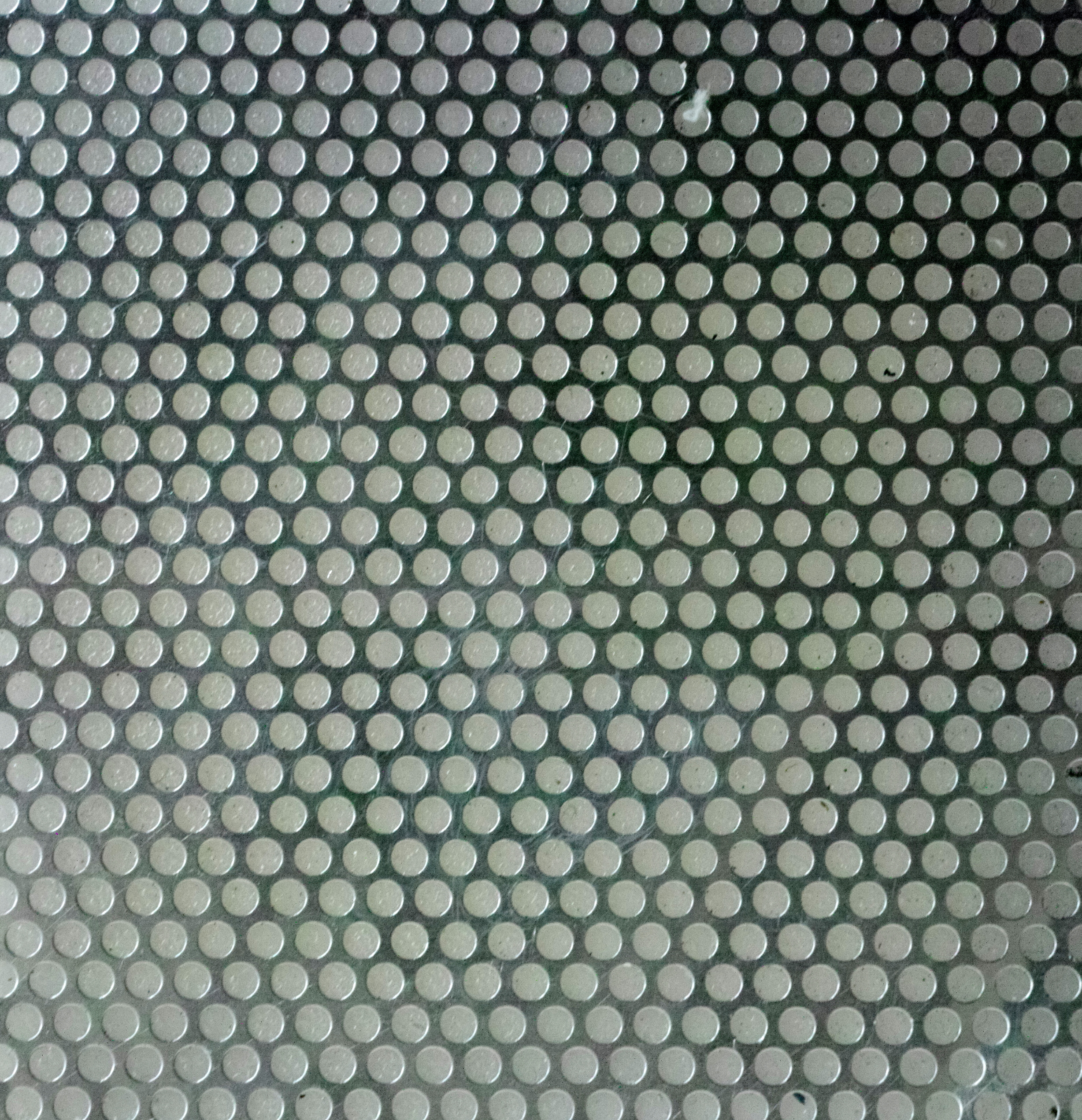

Supplement: Supplementary file 1 — Supplementary Information 2. [file 41598_2023_38929_MOESM1_ESM.zip › 97.JPG]

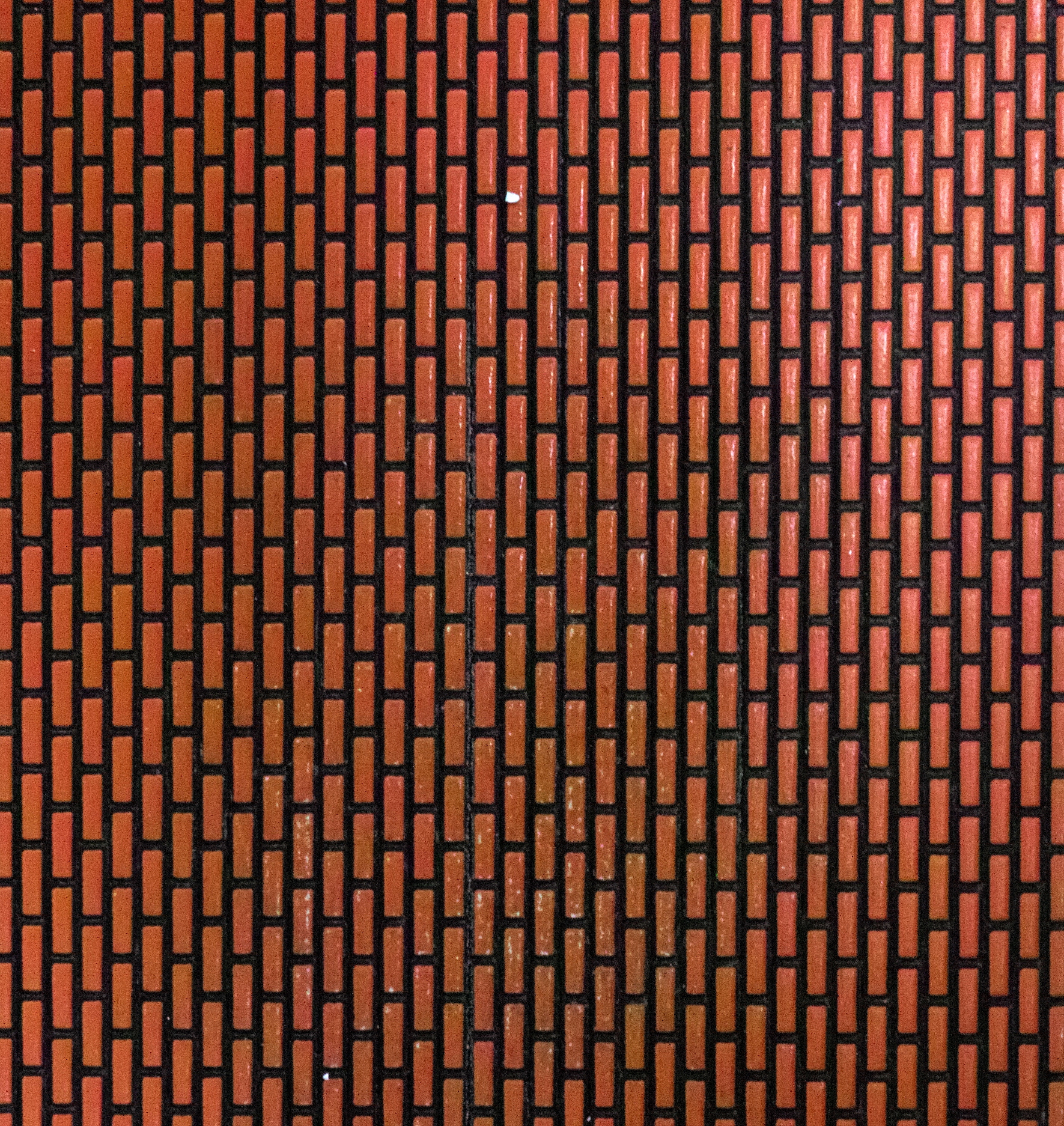

Supplement: Supplementary file 1 — Supplementary Information 2. [file 41598_2023_38929_MOESM1_ESM.zip › 98.JPG]

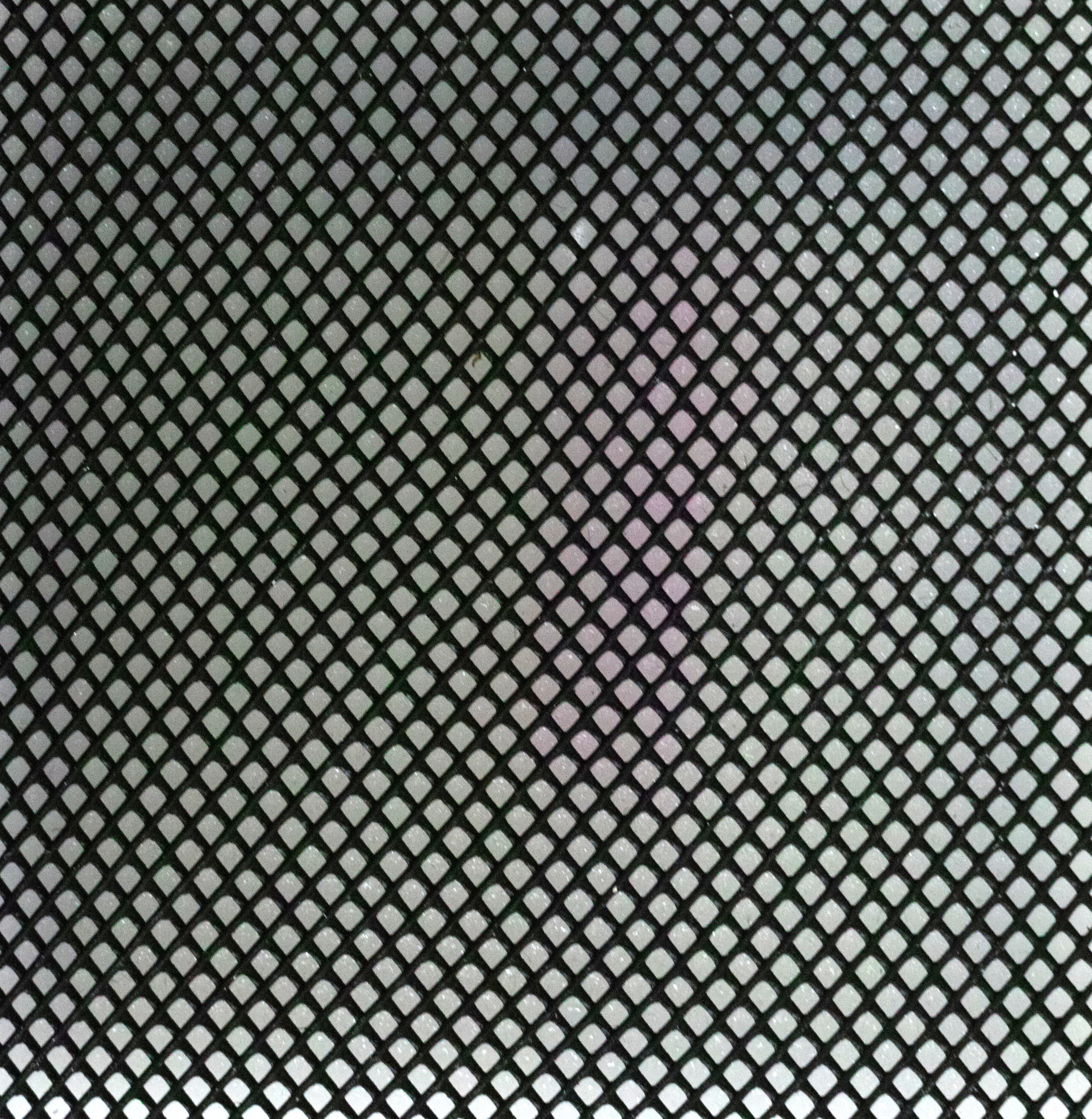

Supplement: Supplementary file 1 — Supplementary Information 2. [file 41598_2023_38929_MOESM1_ESM.zip › 99.JPG]
